# METTL7A-mediated m6A modification of corin reverses bisphosphonates-impaired osteogenic differentiation of orofacial BMSCs

**DOI:** 10.1038/s41368-024-00303-1

**Published:** 2024-05-23

**Authors:** Yizhou Jin, Xiao Han, Yuejun Wang, Zhipeng Fan

**Affiliations:** 1 Beijing Key Laboratory of Tooth Regeneration and Function Reconstruction, Beijing Stomatological Hospital, School of Stomatology, Capital Medical University, Beijing, China; 2 Beijing Laboratory of Oral Health, Capital Medical University, Beijing, China; 3 Research Unit of Tooth Development and Regeneration, Chinese Academy of Medical Sciences, Beijing, China

**Keywords:** Single-molecule biophysics, Gene expression profiling, Mesenchymal stem cells

## Abstract

Bisphosphonate-related osteonecrosis of jaw (BRONJ) is characterized by impaired osteogenic differentiation of orofacial bone marrow stromal cells (BMSCs). Corin has recently been demonstrated to act as a key regulator in bone development and orthopedic disorders. However, the role of corin in BRONJ-related BMSCs dysfunction remains unclarified. A m6A epitranscriptomic microarray study from our group shows that the *CORIN* gene is significantly upregulated and m6A hypermethylated during orofacial BMSCs osteogenic differentiation. Corin knockdown inhibits BMSCs osteogenic differentiation, whereas corin overexpression or soluble corin (sCorin) exerts a promotion effect. Furthermore, corin expression is negatively regulated by bisphosphonates (BPs). Corin overexpression or sCorin reverses BPs-impaired BMSCs differentiation ability. Mechanistically, we find altered expression of phos-ERK in corin knockdown/overexpression BMSCs and BMSCs under sCorin stimulation. PD98059 (a selective ERK inhibitor) blocks the corin-mediated promotion effect. With regard to the high methylation level of corin during osteogenic differentiation, we apply a non-selective m6A methylase inhibitor, Cycloleucine, which also blocks the corin-mediated promotion effect. Furthermore, we demonstrate that METTL7A modulates corin m6A modification and reverses BPs-impaired BMSCs function, indicating that METTL7A regulates corin expression and thus contributes to orofacial BMSCs differentiation ability. To conclude, our study reveals that corin reverses BPs-induced BMSCs dysfunction, and METTL7A-mediated corin m6A modification underlies corin promotion of osteogenic differentiation via the ERK pathway. We hope this brings new insights into future clinical treatments for BRONJ.

## Introduction

Bisphosphonate-related osteonecrosis of jaw (BRONJ) is a class of pathological conditions that mandible bone is exposed to oral environment for more than 8 weeks. BRONJ patients usually have a history of bisphosphonates (BPs) treatment.^1,2^ Attempts in treating BRONJ, such as sequestrectomy or debridement, are reported to induce the bone lesion expanding and the necrotic bone margin is hardly figuring out.^3^ Osteogenesis is important for bone lesion repair, and bone marrow stromal cells (BMSCs) are significant osteoblast progenitors. BMSCs dysfunction may lead to the suppression of bone formation and bone lesion repair.^4–6^ He et al. found that orofacial BMSCs derived from the peripheral and central areas of BRONJ lesions exhibited lower osteogenic ability.^7^ Meanwhile, BPs are reported to induce apoptosis of BMSCs and negatively impact osteogenic differentiation in vitro.^8^ Thus, recovery and promotion orofacial BMSCs osteogenesis function may provide a new strategy for BRONJ treatment.

Corin is a transmembrane serine protease, which is abundantly expressed in heart tissues and is responsible for maintaining normal blood pressure.^9,10^ Besides heart, corin is also identified in bones.^9^ Gene profiling studies found an upregulation of *CORIN* gene in hBMSCs that underwent osteogenic differentiation.^11,12^ Zhou et al. also demonstrated that osteogenic differentiation increased corin expression.^13^ Under physiological and/or pathological conditions, corin is shed from the cell surface and can be detected in human blood.^14,15^ In addition, soluble corin (sCorin) level was dramatically reduced in patients with impaired bone formation, including osteopenia and osteoporosis. This suggests that corin shedding from cells may be an important mechanism to regulate corin function.^16^ These evidences indicate that corin plays a vital role in regulating BMSCs osteogenic differentiation. However, the role of corin in BRONJ-related impairment and the mechanism underlying corin promotion of BMSCs function remain unclarified.

m6A modification is the most abundant epigenetic modification.^17^ The process is regulated by methyltransferases (METTL3, METTL14, WTAP, METTL16, METTL7A, etc.), demethylases (FTO, ALKBH5), and methyl-binding proteins (YTHDFs, YTHDCs, IGF2BPs, HNRNPs, etc.).^18^ Recent advances have shown a close relationship between m6A regulators and osteogenic differentiation. For example, methylases such as METTL3 and METTL14, and demethylase FTO, promoted osteogenic differentiation of apical papilla stem cells (SCAPs), BMSCs and MSCs respectively.^19–21^ METTL7A belongs to the METTL family and exhibits S-adenosyl methionine-dependent methyltransferase activity on RNA.^22^ Initially, METTL7A was considered a key regulator in lipid droplet formation, however, new evidence illustrates that METTL7A promotes osteogenic differentiation of hBMSCs and dental pulp stem cells.^23–25^ The role and key m6A regulator in BRONJ-related orofacial BMSCs impairment remain unclear, which is urgent to explore.

With the development of m6A-epitranscriptomic microarray technology, m6A-modified RNAs have been widely identified and demonstrated to be involved in some orthopedic disorders.^17,26,27^. Applying this new technology may help identify the key factors in regulating osteogenic differentiation, bone formation, and BRONJ-related impairment. Overall, the present study applies m6A-epitranscriptomic microarray technology to elucidate the candidate genes for regulating orofacial BMSCs osteogenic differentiation, and then evaluates the role of corin in BPs-induced BMSCs dysfunction and the underlying mechanism. We demonstrate that increased corin could reverse orofacial BMSCs impairment and potentially enhance the treatment for BRONJ bone lesions.

## Results

### m6A-epitranscriptomic microarray results illustrated the changes in mRNA methylation levels during orofacial BMSCs osteogenic differentiation

**Fig. 1:**
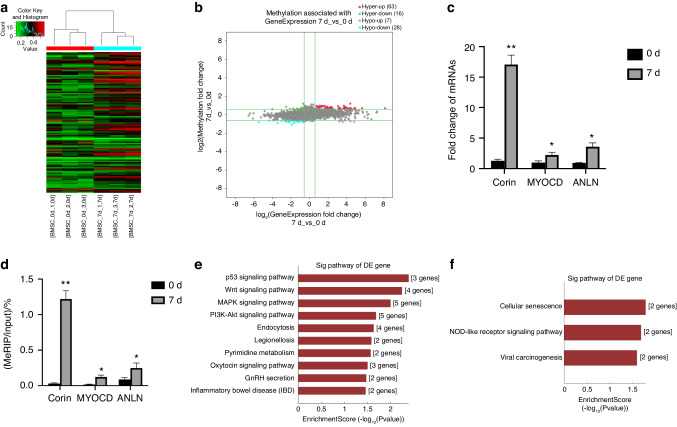
m6A-epitranscriptomic microarray results illustrated the changes in mRNA methylation levels during orofacial BMSCs osteogenic differentiation. **a** Differential m6A-methylated sites in orofacial BMSCs underwent 7-day osteogenic differentiation are displayed in hierarchical clustering. **b** Scatter plot shows differentially methylated genes associated with alterations in gene expression. **c** qRT-PCR analyses of Corin, MYOCD, and ANLN. **d** MeRIP-qPCR results for Corin, MYOCD, and ANLN. **e** KEGG analyses of the differentially expressed m6A hypermethylated genes. **f** KEGG analyses of the differentially expressed m6A hypomethylated genes. Data are expressed as mean ± s.e.m. *P < 0.05, **P < 0.01, 0 d vs. 7 d

To investigate the role of m6A modification in BMSCs differentiation, we utilized m6A-epitranscriptomic microarray technology to compare genes with differential methylation levels between control BMSCs and BMSCs that underwent 7 days of osteogenic differentiation. The hierarchical clustering analysis was conducted based on differentially methylated sites to demonstrate the overall methylation level of BMSCs undergoing 7-day osteogenic differentiation (Fig. 1a). Considering the criteria (fold-change ≥ 1.5, *P* value < 0.05), we identified 4 838 transcripts from 3 675 genes with differentially changed mRNA quantity and 110 transcripts from 108 genes with differentially changed mRNA methylation levels (Supplementary Table 1, 2). The scatter plot analysis revealed differentially methylated genes associated with alterations in gene expression, and there were 63 m6A hypermethylated genes with increased mRNA quantity (Fig. 1b). We then verified and tested the epitranscriptomic microarray results. Among the selected genes, we found that the mRNA quantity and m6A methylation level of the *CORIN* gene were the most significantly upregulated (Fig. 1c, d). Functional analysis was conducted based on “m6A site methylation stoichiometry” to further explore the possible mechanisms underlying orofacial BMSCs osteogenic differentiation. KEGG analysis showed that the top 3 pathways enriched for hypermethylated genes were the p53, Wnt, and MAPK signaling pathways (Fig. 1e). Cellular senescence, NOD-like receptor, and viral carcinogenesis signaling pathway were the main pathways enriched for hypomethylated genes (Fig. 1f).

### Corin promoted orofacial BMSCs osteogenic differentiation in vitro and osteogenesis in vivo

**Fig. 2:**
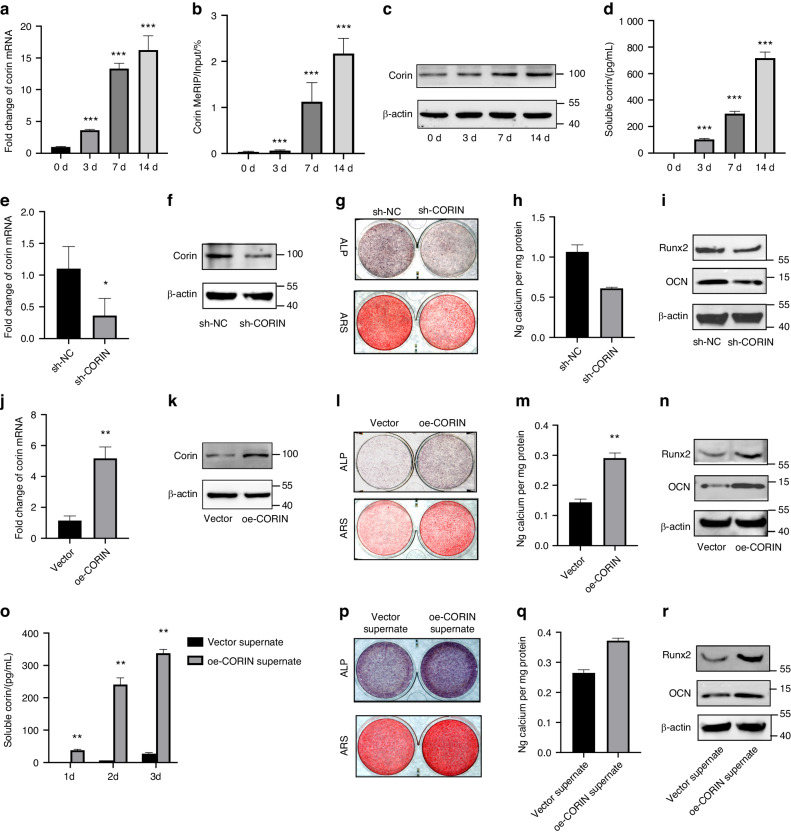
Corin promoted orofacial BMSCs osteogenic differentiation in vitro. **a** qRT-PCR analyses of the corin mRNA expression at 0d, 3d, 7d, 14d after osteogenic differentiation. **b** MeRIP-qPCR results show the m6A modification levels of corin mRNA. **c** Representative immunoblotting shows the protein expression of corin. **d** ELISA results show the sCorin levels at different time points during osteogenic differentiation process. **e**, **f** The mRNA and protein expression of corin from sh-NC/ sh-CORIN Group. **g**, **h** Representative images and quantitative analyses of ALP and ARS staining of BMSCs from sh-NC/ sh-CORIN Group. **i** Representative immunoblotting shows the protein expression of Runx2/OCN from sh-NC/sh-CORIN Group. **j**, **k** The mRNA and protein expression of corin from Vector/oe-CORIN Group. **l**, **m** Representative images and quantitative analyses of ALP and ARS staining of BMSCs from Vector/oe-CORIN Group. **n** Representative immunoblotting shows the protein expression of Runx2/OCN from Vector/oe-CORIN Group. **o** The sCorin levels at different time points from Vector/oe-CORIN Group. **p**, **q** Representative images and quantitative analyses of ALP and ARS staining of BMSCs from Vector/oe-CORIN supernate Group. **r** Representative immunoblotting shows the protein expression of Runx2/OCN from Vector/ oe-CORIN supernate Group. Data are expressed as mean ± s.e.m. *P < 0.05, **P < 0.01, ***P < 0.001, sh-NC or Vector vs. sh-CORIN or oe-CORIN

Consistent with epitranscriptomic microarray results, we found corin mRNA quantity and methylation level were increased in a time-dependent manner during osteogenic differentiation process (Fig. 2a, b). The protein expression level showed a time-dependent increase as well (Fig. 2c). In addition, we collected the osteogenic medium and measured sCorin levels at different time points during the differentiation process. The ELISA-based results illustrated that sCorin levels in the medium supernate were upregulated time-dependently (Fig. 2d). Next, we examined the involvement of corin in regulating orofacial BMSCs osteogenic differentiation. We successfully constructed shRNA-mediated corin knockdown BMSCs (sh-CORIN), as well as corin overexpression BMSCs (oe-CORIN) (Fig. 2e, f, j, k). The ALP and ARS staining, as well as Calcium ion quantification showed much weaker signals in sh-CORIN BMSCs compared to sh-NC BMSCs (Fig. 2g, h), while much stronger signals were observed in oe-CORIN BMSCs compared to Vector BMSCs (Fig. 2l, m). In addition, Western blot results showed lower expression levels of Runx2 and OCN in sh-CORIN BMSCs (Fig. 2i), while higher expression levels were observed in oe-CORIN BMSCs (Fig. 2n). We also investigated whether sCorin promoted osteogenic differentiation. Compared to Vector BMSCs, culture supernate from oe-CORIN BMSCs contained a higher concentration of sCorin (Fig. 2o). Culture supernate (α-MEM + 10% FBS for 3 days) was collected, purified, and mixed with osteogenic medium. ALP/ARS staining, as well as Calcium ion quantification showed much stronger signals in oe-CORIN supernate Group (Fig. 2p, q). Moreover, oe-CORIN supernate Group expressed higher levels of Runx2 and OCN than Vector supernate Group (Fig. 2r). Thus, we demonstrated that corin promoted orofacial BMSCs osteogenic differentiation in vitro.

**Fig. 3:**
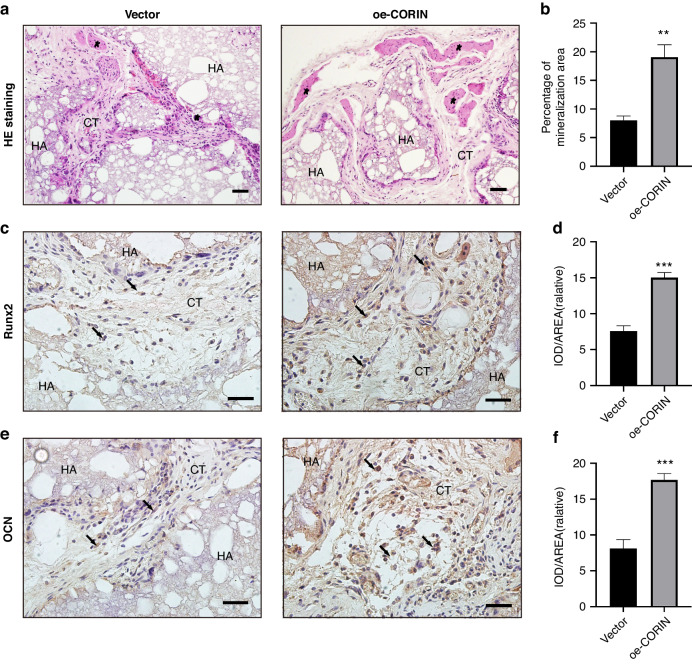
Corin promoted orofacial BMSCs osteogenesis in vivo. **a**, **b** HE staining results show bone-like tissue generation. **c**, **d** Immunohistochemical staining of Runx2 and quantification of its expression. **e**, **f** Immunohistochemical staining of OCN and quantification of its expression. Scale bar: 100 μm. Five-pointed star: bone-like tissue; black arrow: Runx2/OCN-positive BMSCs; HA hydroxyapatite tricalcium carrier, CT connective tissue. Data are expressed as mean ± s.e.m. **P < 0.01, ***P < 0.001, Vector vs. oe-CORIN

In addition, Vector/oe-CORIN BMSCs were mixed with HA/TCP materials and transplanted subcutaneously into the back of mice. The in vivo bone regeneration results illustrated that oe-CORIN BMSCs formed more bone-like tissues (Fig. 3a, b). Immunohistochemical staining and quantitative measurements showed significantly more Runx2/OCN-positive bone matrix in oe-CORIN Group when compared to Vector Group (Fig. 3c–f). Therefore, corin strengthened orofacial BMSCs osteogenesis ability in vivo.

### Corin reversed BPs-impaired orofacial BMSCs osteogenic differentiation in vitro and osteogenesis in vivo

**Fig. 4:**
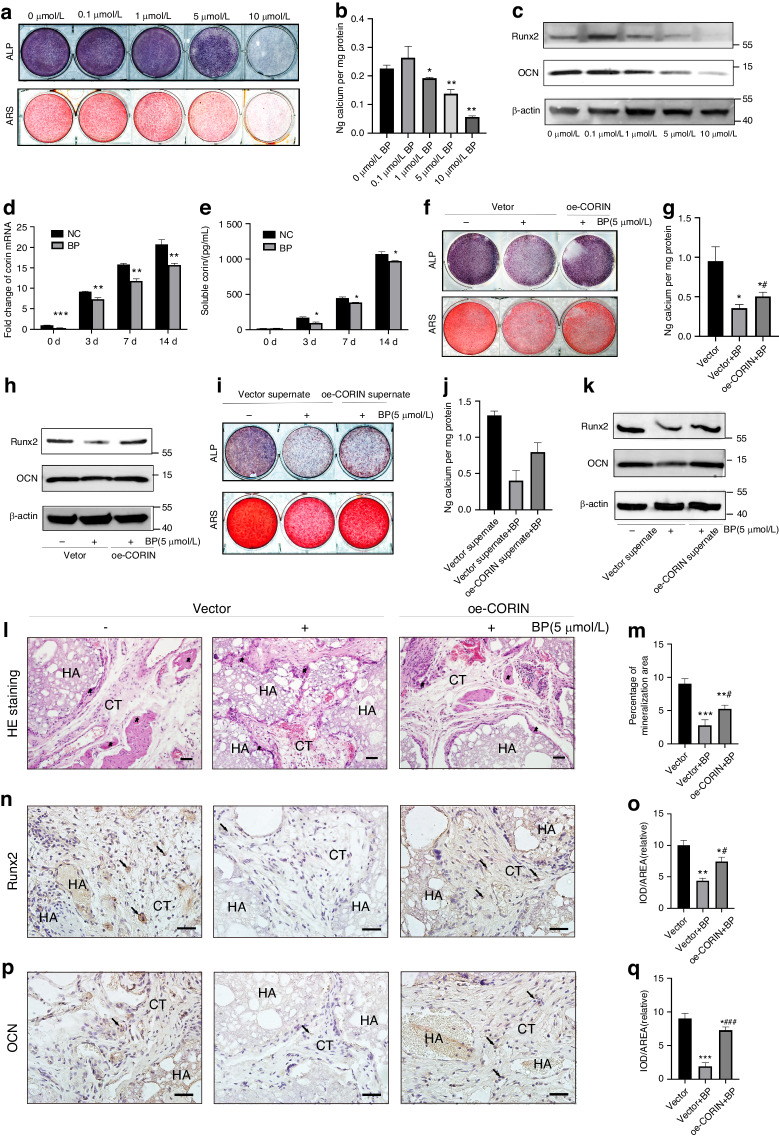
Corin reversed BPs-impaired orofacial BMSCs osteogenic differentiation in vitro and osteogenesis in vivo. **a**, **b** Representative images and quantitative analyses of ALP and ARS staining of BMSCs under dose-dependent zoledronic acid stimulation. **c** Representative immunoblotting shows the protein expression of Runx2/OCN from BMSCs under dose-dependent zoledronic acid stimulation. **d** Pre-treated with 5 µmol/L zoledronic acid for 3 days, qRT-PCR analyses of corin mRNA expression at 0 d, 3 d, 7 d, 14 d after osteogenic differentiation. **e** Pre-treated with 5 µmol/L zoledronic acid for 3 days, ELISA results show the sCorin levels at different time points. **f**, **g** Pre-treated with 5 µmol/L zoledronic acid for 3 days, representative images and quantitative analyses of ALP and ARS staining of BMSCs from Vector/oe-CORIN Group. **h** Representative immunoblotting shows the protein expression of Runx2/OCN from Vector/oe-CORIN Group. **i**, **j** Representative images and quantitative analyses of ALP and ARS staining of BMSCs from Vector/oe-CORIN supernate Group. **k** Representative immunoblotting shows the protein expression of Runx2/OCN from Vector/oe-CORIN supernate Group. **l**, **n**, **p** HE staining results, as well as immunohistochemical staining of Runx2 and OCN. **m**, **o**, **q** Quantitative analyses of HE staining, Runx2 and OCN. Scale bar: 100 μm. Five-pointed star: bone-like tissue; black arrow: Runx2/OCN-positive BMSCs; HA hydroxyapatite tricalcium carrier, CT connective tissue. Data are expressed as mean ± s.e.m. *P < 0.05, **P < 0.01, ***P < 0.001, NC or Vector vs. BP or oe-CORIN. ^#^*P* < 0.05, ^##^*P* < 0.01, ^###^*P* < 0.001, Vector+BP vs oe-CORIN + BP

Among the currently used BPs, zoledronic acid is identified as the most potent nitrogen‐containing BP.^28^ We first detected the impact of zoledronic acid on orofacial BMSCs proliferation and differentiation function. Pre‐treatment with zoledronate (5, 10 μmol/L) for 3 days significantly inhibited BMSCs osteogenic differentiation, as evidenced by weaker signals in ALP/ARS staining and reduced expression levels of Runx2/OCN (Fig. 4a–c). CCK-8 results showed that 10 and 100 μmol/L zoledronic acid had a significantly negative impact on BMSCs proliferation. Therefore we utilized 5 μmol/L zoledronic acid in the subsequent experiments (Supplementary Fig. 1).

We next examined corin expression under zoledronic acid stimulation. We found that the application of zoledronic acid inhibited the upregulation of corin mRNA in orofacial BMSCs undergoing osteogenic differentiation (Fig. 4d). The increase in sCorin levels was also inhibited by zoledronic acid (Fig. 4e). To verify if corin reversed the impaired osteogenic differentiation of BPs-treated BMSCs, we designed a rescue experiment. Pretreatment with zoledronic acid for 3 days resulted in weaker signals in ALP/ARS staining and lower expression levels of Runx2/OCN in the pretreated Vector Group compared to the untreated Vector Group (Fig. 4f–h). However, compared to the pretreated Vector Group, the ALP/ARS staining signals and the Runx2/OCN expression levels in the pretreated oe-CORIN Group were higher, indicating that oe-CORIN BMSCs reversed the negative impact of zoledronic acid (Fig. 4f–h). We also tested whether sCorin alleviated BPs-induced BMSCs dysfunction. Similarly, zoledronic acid pretreated oe-CORIN supernate Group showed stronger signals of ALP /ARS staining and higher expression levels of Runx2/OCN than pretreated Vector supernate Group (Fig. 4i–k). Thus, we demonstrated corin reversed BPs-impaired BMSCs osteogenic differentiation in vitro.

Moreover, compared to the untreated Vector Group, the in vivo bone regeneration results in the pretreated Vector Group showed fewer bone-like tissues, while the pretreated oe-CORIN Group exhibited significantly more bone-like tissues than the pretreated Vector Group (Fig. 4l, m). In addition, immunohistochemical staining of Runx2/OCN in the pretreated oe-CORIN Group was stronger than the pretreated Vector Group (Fig. 4n–q). The above results illustrated that corin alleviated zoledronate-impaired osteogenesis in vivo.

### ERK inhibitor blocked the promotional effect of corin on orofacial BMSCs osteogenic differentiation

**Fig. 5:**
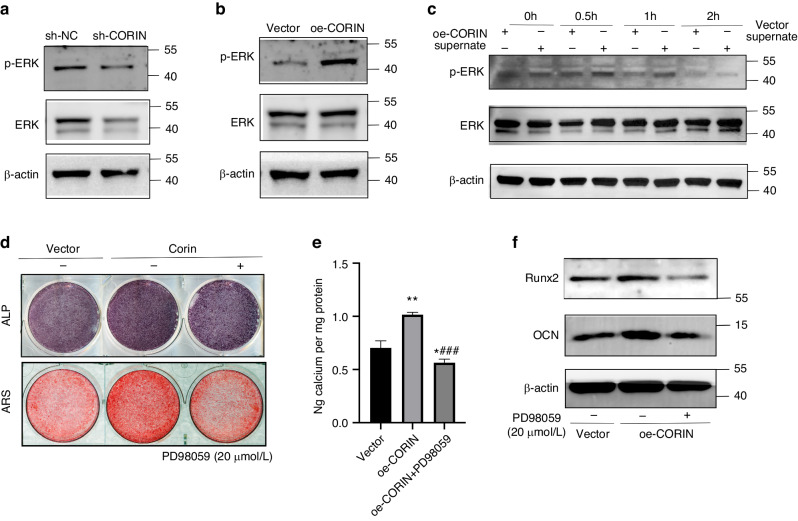
ERK inhibitor blocked corin-mediated promotion of orofacial BMSCs osteogenic differentiation. **a** Representative immunoblotting shows protein expression of p-ERK from sh-NC/ sh-CORIN Group. **b** Protein expression of p-ERK from Vector/oe-CORIN Group. **c** Protein expression of p-ERK from BMSCs under sCorin stimulation. **d**, **e** Treated with 20 µmol/L PD98058, representative images and quantitative analyses of ALP and ARS staining of BMSCs from Vector/oe-CORIN Group. **f** Representative immunoblotting shows the protein expression of Runx2/OCN from Vector/oe-CORIN Group. Data are expressed as mean ± s.e.m. *P < 0.05, **P < 0.01, Vector vs. oe-CORIN. ^###^*P* < 0.001, oe-CORIN vs oe-CORIN + PD98059

According to the KEGG analysis enriched for hypermethylated genes, we detected ERK expression in sh-CORIN and oe-CORIN BMSCs to explore the mechanism underlying corin promoted osteogenic differentiation. We found that the expression of p-ERK was downregulated in sh-CORIN BMSCs, while upregulated in oe-CORIN BMSCs (Fig. 5a, b). In addition, the expression level of p-ERK was increased under sCorin stimulation for 0.5 h and 1 h (Fig. 5c). Furthermore, compared to the untreated oe-CORIN Group, ALP/ARS staining signals and Runx2/OCN expression of were much lower in the PD98059 (a selective ERK inhibitor) treated oe-CORIN Group (Fig. 5d–f), suggesting that ERK inhibitor blocked corin-mediated promotion of orofacial BMSCs osteogenic differentiation.

### METTL7A promoted corin m6A methylation level

**Fig. 6:**
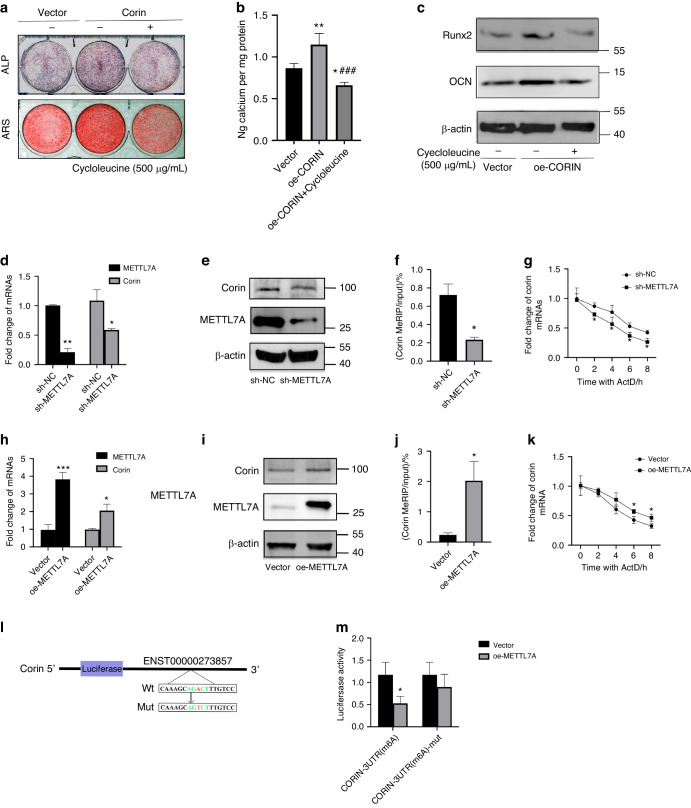
METTL7A promoted corin m6A methylation level. **a**, **b** Treated with 500 µg/mL Cycloleucine, representative images and quantitative analyses of ALP and ARS staining of BMSCs from Vector/oe-CORIN Group. **c** Representative immunoblotting shows the protein expression of Runx2/OCN from Vector/oe-CORIN Group. **d**, **e** qRT-PCR analyses, as well as representative immunoblotting show corin expression from sh-NC/sh-METTL7A Group. **f** MeRIP-qPCR results show m6A modification of corin mRNA from sh-NC/sh-METTL7A Group. **g** Comparation of corin mRNA stability from sh-NC/sh-METTL7A Group. **h**, **i** Corin expression from Vector/oe-METTL7A Group. **j** MeRIP-qPCR results show m6A modification on corin mRNA from Vector/oe-METTL7A Group. **k** Comparation of corin mRNA stability from Vector/oe-METTL7A Group. **l** Construct of Wildtype and mutant 3’UTR CORIN plasmids. **m** METTL7A binding to the CORIN 3′UTR is verified by the dual-luciferase reporter assay. Data are expressed as mean ± s.e.m. *P < 0.05, **P < 0.01, ***P < 0.001, sh-NC or Vector vs. sh-METTL7A or oe-METTL7A. ^###^*P* < 0.001, oe-CORIN vs oe-CORIN+Cycloleucine

With regard to the increased methylation level of corin during osteogenic differentiation, we applied Cycloleucine (a non-selective m6A methylase inhibitor) in the osteogenic differentiation process. We found that ALP/ARS staining and Runx2/OCN expression in the Cycloleucine treated oe-CORIN Group were much lower than the untreated oe-CORIN Group (Fig. 6a–c), suggesting that m6A modification of corin influenced osteogenic differentiation.

To study the molecular mechanism underlying the upregulated corin methylation level, we tested the expression of common methyltransferases during osteogenic differentiation. We found that the mRNA quantity of METTL7A was significantly upregulated (Supplementary Fig. 2). We constructed shRNA-mediated METTL7A knockdown BMSCs (sh-METTL7A), and METTL7A overexpression BMSCs (oe-METTL7A) (Fig. 6d, e, h, i). We found that corin mRNA and protein expression, as well as m6A modification level and mRNA stability were decreased in sh-METTL7A BMSCs (Fig. 6d–g). While in oe-METTL7A BMSCs, corin mRNA quantity, protein expression, m6A modification level, and mRNA stability were increased, suggesting that METTL7A regulated corin expression at transcriptional level (Fig. 6h–k). According to the prediction of m6A recognition sites based on SRAMP, we conducted luciferase reporter and mutagenesis assays (Fig. 6l). Compared to mutant *CORIN* 3’UTR plasmid, co-transfection of METTL7A with wild-type *CORIN* 3’UTR plasmid significantly reduced the luciferase activity, indicating the interaction between METTL7A and *CORIN* 3’UTR (Fig. 6m). Taken together, our data revealed that METTL7A promoted m6A modification of corin on 3’UTR region.

### METTL7A reversed BPs-impaired orofacial BMSCs osteogenic differentiation in vitro and osteogenesis in vivo

**Fig. 7:**
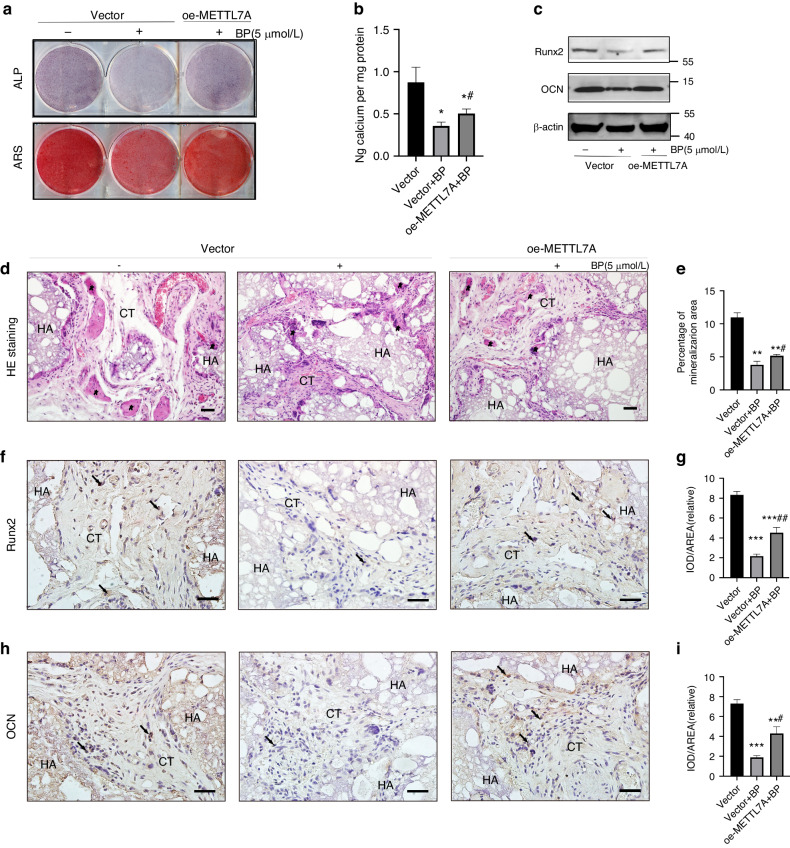
METTL7A reversed BPs-impaired orofacial BMSCs osteogenic differentiation in vitro and osteogenesis in vivo. **a**, **b** Pre-treated with 5 µmol/L zoledronic acid for 3 days, representative images and quantitative analyses of ALP and ARS staining of BMSCs from Vector/oe-METTL7A Group. **c** Representative immunoblotting shows the protein expression of Runx2/OCN from Vector/oe-METTL7A Group. **d**, **e** HE staining results show bone-like tissue generation. **f**, **g** Immunohistochemical staining of Runx2 and quantification of its expression. **h**, **i** Immunohistochemical staining of OCN and quantification of its expression. Scale bar: 100 μm. Five-pointed star: bone-like tissue; black arrow: Runx2/OCN-positive BMSCs; HA hydroxyapatite tricalcium carrier, CT connective tissue. Data are expressed as mean ± s.e.m.; *P < 0.05, **P < 0.01, ***P < 0.001, Vector vs. METTL7A. ^#^*P* < 0.05, ^##^*P* < 0.01, Vector+BP vs. oe-METTL7A + BP

As an m6A regulator, we then verified whether METTL7A reversed BP-induced orofacial BMSCs dysfunction. Pre-treatment with 5 µmol/L zoledronic acid for 3 days, ALP and ARS staining, Calcium ion quantification, as well as Runx2/OCN expression levels in the pretreated Vector Group were much lower compared to the untreated Vector Group. However, ALP/ARS staining signals and Runx2/OCN expression levels in the pretreated oe-METTL7A Group were higher than in the pretreated Vector Group, suggesting that METTL7A reversed BPs-induced inhibition of osteogenic differentiation (Fig. 7a–c). Moreover, in vivo bone regeneration results showed less bone-like tissue formation was observed in the pretreated Vector Group than in the untreated-Vector Group, while more tissue formation in pretreated the oe-METTL7A Group than in the pretreated Vector Group (Fig. 7d, e). In addition, immunohistochemical staining of Runx2/OCN in the pretreated oe-METTL7A Group was stronger than in the pretreated Vector Group (Fig. 7f–i). The above results illustrated that METTL7A reversed zoledronate-impaired osteogenesis in vivo.

## Discussion

BRONJ is a type of medication-related osteonecrosis of the jaw. Considering the negative regulation of zoledronate on orofacial BMSCs and the impaired differentiation ability of BMSCs extracted from BRONJ patients, the recovery and enhancement of BMSCs function become an important part for BRONJ treatment. In order to identify the key regulators that reverse and promote the differentiation ability of BMSCs, m6A-epitranscriptomic microarray technology was utilized. We observed that the altered expression of bone formation-related genes tended to be stable on the seventh day during the osteogenic differentiation course. Therefore, we compared the control BMSCs with BMSCs that underwent 7 days of osteogenic differentiation in an m6A-epitranscriptomic microarray study. We verified and tested the epitranscriptomic microarray results, and found that the mRNA quantity and m6A methylation levels of *CORIN*, *MYOCD*, and *ANLN* genes were upregulated simultaneously. MYOCD is a transcriptional coactivator that mainly regulates the function of cardiac muscle and smooth muscle.^29^ ANLN is an actin-binding protein which primarily regulates cancer cell proliferation.^30^ The upregulation of corin is the most significant among the selected genes. In addition, in this study we demonstrate the role of corin in promoting osteogenic differentiation, which is consistent with Zhou’s findings.^13^

Zoledronic acid is the first-line pharmacologic treatment for osteoporosis and is extensively used in establishing BRONJ animal model and stimulating concerning cells in vitro.^31,32^ Mechanistically, zoledronate plays antiresorptive roles in osteoporosis treatment by inhibiting osteoclast differentiation and inducing osteoclast apoptosis.^33,34^ New evidence has proved the negative regulation of zoledronate on osteoblasts, as well as mesenchymal cells.^35,36^ Interestingly, we notice that the regulation of zoledronate on BMSCs seems double-edged. Low concentration (0.1 μmol/L) of zoledronate slightly promotes BMSCs proliferation and osteogenic differentiation (without statistical significance), while high concentrations (5, 10 μmol/L) of zoledronate inhibited BMSCs proliferation and osteogenic differentiation. Zoledronate at a concentration of 100 μmol/L severely induced BMSCs cell death. To confirm the positive impact of zoledronate on BMSCs, additional research is required. During the osteogenic induction process, we pre-treated BMSCs with 5 μmol/L zoledronate for only 3 days, yet the negative impact persists for an extended period. In nude-mice transplantation experiments, the negative influence of zoledronate lasts for 8 weeks. This long-lasting effect is probably due to the fact that zoledronate is capable of tightly binding to hydroxyapatite once absorbed and can be continuously released from traumatic lesion.^8^

Moreover, we demonstrate for the first time that corin reverses BPs-impaired BMSCs differentiation ability. We also detected sCorin level in BMSCs culture medium and found it was time-dependently upregulated during the osteogenic process. sCorin collected from culture medium of oe-CORIN BMSCs promoted osteogenic differentiation and reversed BPs-induced impairment in vitro. We hypothesize that the administration of sCorin in a BRONJ animal model would promote the repair of bone lesions in vivo. As mentioned above, serum corin levels were decreased in patients with orthopedic disorders, including osteopenia and osteoporosis.^16^ Patients with BRONJ lesions may also experience a decrease in serum corin levels. It is necessary to mention that the upregulation of corin has been proven to accompany with adipogenic and chondrogenic differentiation of BMSCs.^13^ BMSCs extracted from BRONJ lesion sites exhibit reduced adipogenic differentiation ability and osteoclast-inducing capacity. Whether corin is correlated with osteoclast-inducing capacity, or whether corin-mediated promotion of adipogenic and chondrogenic differentiation plays a role in BRONJ treatment, requires further exploration.

During the osteogenic differentiation process, we investigate the role of the high m6A methylation level of corin in regulating BMSCs function. Cycloleucine is a non-metabolizable amino acid that participates in the production of S-adenosyl-L-methionine (a methyl donor).^37^ and inhibits the methylation level of RNA.^38^ We applied Cycloleucine in our study and observed that Cycloleucine blocked corin promotion of osteogenic differentiation, indicating that m6A modification regulates corin function. We tested the expression of METTL3, METTL14, and METTL7A in BMSCs that underwent 7 days of osteogenic differentiation, and found that METTL7A mRNA quantity was upregulated significantly. Then, we demonstrated that knockdown or overexpression of METTL7A influenced the expression, methylation level, as well as mRNA stability of corin, indicating that METTL7A regulates corin expression at the mRNA level. In addition, luciferase reporter assays further demonstrated the interaction between METTL7A and *CORIN* 3’UTR, providing evidence that METTL7A regulates corin m6A modification. As an m6A regulator of corin, METTL7A also reversed BPs-induced BMSCs dysfunction. Targeting METTL7A may provide a new strategy for BRONJ treatment. It is necessary to mention that our study does not exclude the possible role of other m6A regulators in regulating corin and the potential regulatory effect of METTL7A on osteoclasts in the context of BRONJ, which worth further exploring.

To explore the downstream signaling pathway underlying corin promotion of osteogenic differentiation, a KEGG analysis of epitranscriptomic microarray was conducted. According to the pathways enriched for hypermethylated genes, p53, Wnt, and MAPK ranked as the top 3. There were 5 hypermethylated genes belonging to the MAPK pathway, which accounted for the most. MAPK pathway is a potent signaling pathway that modulates various cellular processes, such as cell proliferation, apoptosis, and differentiation. ERK1 or ERK2 are conventional MAPK components.^39^ We tested the expression of phos-ERK and total-ERK in sh-CORIN/oe-CORIN BMSCs, as well as BMSCs under sCorin stimulation. We observed the changes in phos-ERK expression. Moreover, PD98059 (an ERK inhibitor) blocked corin-promoted BMSCs function, indicating that the ERK pathway underlies corin promotion of osteogenic differentiation.

In conclusion, we demonstrate that the overexpression of corin or the application of sCorin reverses BPs-impaired BMSCs osteogenesis. METTL7A promotes corin expression at the mRNA level and reverses BPs-impaired BMSCs function as well. Our study reveals the key role of the METTL7A-corin-ERK pathway in reversing BPs-induced BMSCs dysfunction. It explains the novel mechanism underlying BMSCs osteogenic differentiation under BPs stimulation, aiming to bring new insights into the future treatment for BRONJ.

## Materials and methods

### Cell culture and drug administration

Orofacial BMSCs were isolated from the human mandible bone as previously described.^7^ The tissue was acquired when subjects underwent orthopedic surgery, and the procedure followed the ethics guideline approved by the Ethics Committee of Beijing Stomatology Hospital (Approval number: CMUSH-IRB-KJ-PJ-2023-40). Briefly, the bone tissues were thoroughly rinsed and digested for 30 min. The suspension was collected and seeded in culture medium. The cells were continuously cultured, and passage 3–5 were used in the following study. Zoledronic acid (SML0223, Sigma-Aldrich, US) was added to the culture medium 3 days before inducing osteogenic differentiation. PD98059 (HY-12028, MCE, Shanghai, China) and Cycloleucine (HY-30008, MCE, Shanghai, China) were added to the culture medium 3 days before inducing osteogenic differentiation and continuously supplemented in the osteogenic medium.

### m6A-mRNA&lncRNA epitranscriptomic microarray

m6A-epitranscriptomic microarray and mRNA microarray analyses were conducted by Aksomics Company (Shanghai, China). The procedure was conducted as previously reported.^40^

### m6A MeRIP-quantitative real-time PCR

m6A MeRIP-qPCR was performed as previously reported.^41^ Briefly, extracted RNAs were fragmented into 300 nucleotides by incubating at 94 °C for 2 min. The input sample was preserved. The rest fragmented RNA was incubated with anti-m6A antibody (A19841, ABclonal, Wuhan, China) for 4 h and then incubated with protein A/G magnetic beads for 2 h. The input and the m6A-IP RNA were finally analyzed using qPCR technology. The primer sequences are listed in Supplementary Table 3.

### Construction of plasmids and transfection of virus

*CORIN/METTL7A* short hairpin RNA, full-length *CORIN/METTL7A*, and Wt/Mut *CORIN* 3’UTR-Luc plasmids were constructed by Genechem Company (Shanghai, China). sh-CORIN, 5′-CCTCTCCTCTCAGTTGTCAGAAACA-3′; and sh-METTL7A, 5′-GGTTCACTGTGATATACAACG-3′ plasmids were subcloned into the GV493 lentiviral vector. Human full-length *CORIN* and *METTL7A* gene sequences were subcloned into the GV492 lentiviral vector. Wt/Mut *CORIN* 3’UTR-Luc plasmids were subcloned into the GV272 lentiviral vector. Virus transfection was conducted as previously described.^24^

### RNA extraction and real-time PCR

TRIzol was used to extract total RNA from orofacial BMSCs. Reverse-transcription Kit instructions (R333, Vazyme, Nanjing, China) were followed. GAPDH was used to normalize mRNA levels. The primer sequences are listed in Supplementary Table 3.

### Western blot

Total proteins were extracted as described previously.^27^ The primary antibodies used were as follows: anti-corin antibody (AF2209, R&D, MN. US), anti-ERK antibody (4695 S, CST, MA. US), anti-Phospho-ERK antibody (4370 S, CST, MA. US), anti-METTL7A antibody (67905-1-lg, Proteintech, Wuhan, China) and anti-β-actin antibody (TA-09, ZSGB-BIO, Beijing, China).

### Soluble corin detection and collection

Culture medium was collected at different time points and centrifuged at 1 000 g for 20 min. sCorin in the supernatant was detected following the instructions provided in the human Corin Quantikine ELISA kit (DCRN00, R&D, MN, US). For the sCorin collection, the culture medium (α-MEM + 10%FBS for 3 days) of Vector/oe-CORIN BMSCs was collected and purified using a dialysis tube (UFC803008, Millipore, MA. US).

### Alkaline phosphatase and alizarin red staining detection

Orofacial BMSCs were cultured in osteogenic medium which was changed every 3 days. After 7 days, ALP nodes were stained using an ALP staining kit (C3206, Beyotime, Shanghai, China). After 2 weeks, the mineralization nodes were stained with Alizarin Red.

### Transplantation in nude mice and immunohistochemical staining

Female NU/NU mice (8 weeks old, Vital River Experimental Animal Technique Company, China) were used in this study. The experiments were approved by the Animal Ethics Committee of Beijing Stomatology Hospital (Approval number: KQYY-202307-003). 2 × 10^6^ orofacial BMSCs were mixed with 20 mg of HA/tricalcium phosphate and then transplanted subcutaneously into the back of the mice. After 8 weeks, the samples were harvested, and HE/immunohistochemical staining was performed as described previously.^27^ The primary antibodies used were as follows: anti-Runx2 antibody (1134 R, Bioss, Beijing, China), anti-OCN antibody (4917 R, Bioss, Beijing, China).

### RNA stability assays

Cells were treated with 5 μg/mL Actinomycin D (HY17559, MCE, Shanghai, China) for 0, 2, 4, 6, and 8 h before RNA extraction and qRT-PCR detection.

### Dual-luciferase reporter assays

WT or mutant *CORIN* 3′UTR-Luc reporter plasmids and METTL7A or Vector plasmids were co-transfected into 293 T cells for 48 h. The dual-luciferase reporter assay kit (DL101-01, Vazyme, Nanjing, China) was used to measure the activity.

### Statistics analysis

All data were presented as mean ± SD. Differences between two groups were examined using an independent samples t-test. Differences among more than two groups were examined using a one-way ANOVA test, followed by Tukey’s multiple comparisons test. In all cases, *P* < 0.05 was considered as statistically significant.

### Supplementary information

Supplementary Table 1

Supplementary Table 2

Supplementary Table 3

Supplementary Figure 1

Supplementary Figure 2

## Data Availability

All data used to support the findings of this study are included within the article.
