# Supplementary material for: METTL7A-mediated m6A modification of corin reverses bisphosphonates-impaired osteogenic differentiation of orofacial BMSCs
Source: Int J Oral Sci. 2024 May 23;16:42. doi: 10.1038/s41368-024-00303-1 (PMC11116408; doi:10.1038/s41368-024-00303-1)
Supplement: Supplementary file 1 — Supplementary Table 1 [file 41368_2024_303_MOESM1_ESM.docx]

**Supplementary Table1. m6A-epitranscriptomic microarray results illustrated genes with differentially mRNA quantity**

| **GeneSymbol** | **Transcript ID** | **Regulation** | **Fold Change**  **（log）2** | **P value** |
| --- | --- | --- | --- | --- |
| CLEC10A | ENST00000254868 | up | 8.7943805 | 7.137E-07 |
| DIO3 | ENST00000510508 | up | 7.7273855 | 0.0003378 |
| CXCL6 | ENST00000226317 | up | 7.6131751 | 8.622E-06 |
| CXCL1 | ENST00000395761 | up | 7.4105248 | 3.911E-06 |
| PTBP2 | NM_001300986 | up | 6.4795546 | 4.892E-06 |
| LRRC15 | ENST00000347624 | up | 6.4067901 | 4.287E-05 |
| WISP1 | ENST00000250160 | up | 6.3723218 | 1.238E-05 |
| GPR68 | ENST00000535815 | up | 6.2247766 | 1.371E-06 |
| CATG00000087832.1 | FTMT22200002393 | up | 6.1589416 | 2.57E-06 |
| RERG | ENST00000256953 | up | 6.0695411 | 5.002E-05 |
| PLCB2 | ENST00000557821 | up | 5.987435 | 5.934E-06 |
| CCL8 | ENST00000394620 | up | 5.9083888 | 0.0001047 |
| NUF2 | ENST00000271452 | up | 5.8455984 | 0.0005226 |
| SPC25 | ENST00000282074 | up | 5.6969301 | 1.93E-05 |
| PDE8B | ENST00000346042 | up | 5.6958101 | 1.369E-06 |
| KRTAP2-3 | ENST00000391418 | up | 5.6911633 | 0.0002034 |
| PIP | ENST00000291009 | up | 5.5976447 | 0.0001943 |
| WFDC1 | ENST00000219454 | up | 5.4955582 | 1.668E-05 |
| E2F8 | ENST00000620009 | up | 5.4454349 | 0.0002921 |
| ANGPT2 | ENST00000629816 | up | 5.3985115 | 8.649E-05 |
| MCM10 | ENST00000378714 | up | 5.359766 | 3.031E-05 |
| KIF18B | ENST00000587309 | up | 5.3572497 | 3.274E-05 |
| ASPN | ENST00000375544 | up | 5.3343434 | 0.0008229 |
| NDP | ENST00000642620 | up | 5.1878001 | 0.0004433 |
| ANLN | ENST00000396068 | up | 5.1472373 | 3.848E-05 |
| CKAP2L | ENST00000302450 | up | 5.1383304 | 0.001371 |
| CORIN | ENST00000504584 | up | 5.0800789 | 5.777E-05 |
| BMP6 | ENST00000283147 | up | 5.0278918 | 1.031E-05 |
| BUB1B | ENST00000287598 | up | 4.9958062 | 0.0006442 |
| MYOCD | ENST00000425538 | up | 4.9788386 | 0.0001218 |
| RRM2 | ENST00000304567 | up | 4.9066761 | 6.509E-07 |
| PPP4R4 | ENST00000304338 | up | 4.8669005 | 0.0003989 |
| KCNJ15 | ENST00000328656 | up | 4.8620259 | 0.0001161 |
| TRPA1 | ENST00000262209 | up | 4.8581508 | 0.0001593 |
| IGF2 | ENST00000381395 | up | 4.8396799 | 5.517E-05 |
| CKAP2L | NM_001304361 | up | 4.7810923 | 0.0003648 |
| POU2F2 | ENST00000389341 | up | 4.7695952 | 0.0007556 |
| COL5A3 | ENST00000264828 | up | 4.7542848 | 2.384E-05 |
| VASH2 | ENST00000366966 | up | 4.734621 | 1.801E-05 |
| SAA2 | ENST00000526900 | up | 4.7256694 | 5.565E-05 |
| NKD2 | ENST00000296849 | up | 4.7009492 | 1.708E-05 |
| CSF2 | ENST00000296871 | up | 4.6933618 | 0.000142 |
| HIST1H1B | ENST00000331442 | up | 4.6498261 | 0.0029105 |
| SKA3 | ENST00000400018 | up | 4.6476378 | 3.293E-05 |
| NUF2 | ENST00000367900 | up | 4.6442561 | 0.0002759 |
| ANLN | ENST00000265748 | up | 4.5967572 | 0.0012596 |
| CDC45 | ENST00000263201 | up | 4.576437 | 1.011E-05 |
| DLGAP5 | ENST00000247191 | up | 4.5661276 | 0.0001845 |
| NCAPG | ENST00000251496 | up | 4.5644846 | 5.481E-05 |
| CATG00000052031.1 | MICT00000210435 | up | 4.5595825 | 0.0001664 |
| GTSE1 | ENST00000454366 | up | 4.5291456 | 1.597E-05 |
| OLFM2 | ENST00000264833 | up | 4.5268943 | 0.0002965 |
| TNFAIP3 | ENST00000237289 | up | 4.5146074 | 0.0028496 |
| LEP | ENST00000308868 | up | 4.4935746 | 0.0007413 |
| CORIN | ENST00000273857 | up | 4.4647485 | 0.0011901 |
| FAM111B | ENST00000529618 | up | 4.4290227 | 0.0001265 |
| KRTAP1-1 | ENST00000306271 | up | 4.3841113 | 9.766E-05 |
| KCNB1 | ENST00000371741 | up | 4.3780305 | 0.0017472 |
| IGFBP2 | ENST00000233809 | up | 4.344704 | 0.002347 |
| SLA | ENST00000338087 | up | 4.2993639 | 0.0003071 |
| CXCL5 | ENST00000296027 | up | 4.2628864 | 1.504E-06 |
| MKI67 | ENST00000368654 | up | 4.2434274 | 0.0004019 |
| CCL2 | ENST00000225831 | up | 4.2046073 | 0.0006578 |
| NOG | ENST00000332822 | up | 4.1890452 | 0.0077865 |
| MEOX2 | ENST00000262041 | up | 4.1803441 | 0.0005753 |
| HCK | ENST00000375852 | up | 4.1691979 | 0.0003948 |
| ANGPT2 | ENST00000338312 | up | 4.1511815 | 0.0022202 |
| SAA1 | ENST00000532858 | up | 4.0578816 | 3.348E-05 |
| EXO1 | ENST00000348581 | up | 4.0414094 | 0.0006905 |
| MMP12 | ENST00000571244 | up | 4.0382461 | 0.003934 |
| PRRX2 | ENST00000372469 | up | 4.0315898 | 7.262E-05 |
| ZNF25 | ENST00000302609 | up | 4.0315776 | 0.0029247 |
| RAET1E | ENST00000532335 | up | 4.0234835 | 0.001595 |
| CLEC3B | ENST00000428034 | up | 4.0133112 | 0.0170808 |
| ASPM | ENST00000294732 | up | 4.0046976 | 0.0010989 |
| VCAM1 | ENST00000294728 | up | 3.9596688 | 0.0001847 |
| ZNF25 | NM_001329652 | up | 3.9545572 | 0.0023743 |
| GREM1 | ENST00000622074 | up | 3.9506327 | 0.00051 |
| VNN1 | ENST00000367928 | up | 3.9461093 | 0.0005221 |
| FAM83D | uc002xjf.3 | up | 3.941793 | 0.0008334 |
| RAD51AP1 | ENST00000352618 | up | 3.9327126 | 0.00078 |
| VCAM1 | ENST00000347652 | up | 3.9291301 | 2.787E-05 |
| PRC1 | ENST00000361188 | up | 3.8757078 | 0.0006597 |
| EHF | ENST00000257831 | up | 3.8652207 | 0.0004603 |
| CYP7B1 | ENST00000310193 | up | 3.8370302 | 0.0013331 |
| HCK | ENST00000520553 | up | 3.8310969 | 0.0013605 |
| KRTAP1-5 | ENST00000361883 | up | 3.8211883 | 0.0001814 |
| KIFC1 | ENST00000428849 | up | 3.816672 | 0.0003196 |
| FRZB | ENST00000295113 | up | 3.8027136 | 0.0024497 |
| CMKLR1 | ENST00000412676 | up | 3.8009633 | 0.0002695 |
| SGO1 | ENST00000443724 | up | 3.7991791 | 0.0001945 |
| TNFSF15 | ENST00000374045 | up | 3.7893817 | 0.0003273 |
| CDK1 | ENST00000316629 | up | 3.7442372 | 0.000213 |
| GALNT15 | ENST00000617544 | up | 3.7356439 | 0.0003818 |
| GRIA3 | ENST00000622768 | up | 3.7242915 | 2.316E-05 |
| ACKR4 | ENST00000249887 | up | 3.7187358 | 0.005443 |
| PDZRN3 | ENST00000479530 | up | 3.6981972 | 0.0028077 |
| MCM10 | uc001imc.3 | up | 3.6951604 | 0.0001535 |
| CXCL3 | ENST00000296026 | up | 3.6889707 | 1.202E-05 |
| MGP | ENST00000539261 | up | 3.6880731 | 0.000314 |
| PTPRQ | NM_001145026 | up | 3.6879804 | 0.0021916 |
| WNT10B | ENST00000301061 | up | 3.6661487 | 0.0003413 |
| SAA1 | ENST00000405158 | up | 3.6385464 | 0.0006028 |
| CCIN | ENST00000335119 | up | 3.6384191 | 0.0010116 |
| IL32 | ENST00000382213 | up | 3.6274217 | 0.0011469 |
| PBK | ENST00000301905 | up | 3.5994428 | 0.0022828 |
| STC1 | ENST00000290271 | up | 3.5946667 | 0.0006208 |
| CCL5 | ENST00000605509 | up | 3.5675986 | 0.0002167 |
| TOP2A | ENST00000423485 | up | 3.5628435 | 0.0017332 |
| CLSPN | ENST00000373220 | up | 3.5150099 | 0.0004315 |
| RAD51 | ENST00000267868 | up | 3.4814767 | 0.0071261 |
| ASPM | ENST00000367409 | up | 3.4725625 | 0.0002223 |
| ASPM | ENST00000367409 | up | 3.4725625 | 0.0002223 |
| ERCC6 | ENST00000447839 | up | 3.4692068 | 0.0004984 |
| BUB1 | ENST00000409311 | up | 3.4644645 | 0.0069436 |
| IBA57 | ENST00000366711 | up | 3.4585211 | 8.441E-06 |
| IGFBP2 | ENST00000456764 | up | 3.4582953 | 2.555E-05 |
| SHANK2 | NM_133266 | up | 3.451097 | 0.000416 |
| KIF23 | ENST00000260363 | up | 3.4272738 | 0.0078546 |
| CDCA2 | ENST00000330560 | up | 3.4228131 | 0.0056696 |
| FANCA | ENST00000389301 | up | 3.4209328 | 0.0009182 |
| FAM83D | ENST00000619850 | up | 3.4185577 | 0.0022453 |
| APCDD1 | ENST00000355285 | up | 3.4169729 | 0.0004469 |
| CASC5 | ENST00000399668 | up | 3.4047419 | 0.0023815 |
| INHBB | ENST00000295228 | up | 3.4040486 | 0.0003863 |
| SAA1 | NM_000331 | up | 3.3947752 | 0.0015102 |
| GINS4 | ENST00000276533 | up | 3.3870271 | 0.0041374 |
| SOD2 | ENST00000538183 | up | 3.3661213 | 0.0003245 |
| KCNK6 | ENST00000263372 | up | 3.3485742 | 0.0085249 |
| SLC2A12 | ENST00000275230 | up | 3.341423 | 0.001705 |
| RFTN2 | ENST00000295049 | up | 3.3403476 | 0.0174343 |
| TTK | ENST00000369798 | up | 3.3349929 | 0.0010601 |
| CRTAC1 | ENST00000370597 | up | 3.3278007 | 0.0003422 |
| RAB27B | ENST00000262094 | up | 3.3267018 | 0.0006229 |
| C11orf96 | ENST00000617612 | up | 3.310111 | 0.0009838 |
| NEIL3 | ENST00000264596 | up | 3.2989732 | 0.0003687 |
| GPR183 | ENST00000376414 | up | 3.2940597 | 0.0039 |
| CDKN2C | ENST00000396148 | up | 3.2692082 | 0.01595 |
| HELLS | ENST00000239026 | up | 3.2651605 | 0.0001891 |
| NEK2 | ENST00000366999 | up | 3.2593549 | 0.0004682 |
| CDK1 | ENST00000373809 | up | 3.2539518 | 0.0002562 |
| UBE2C | ENST00000356455 | up | 3.2468121 | 3.43E-05 |
| FGF5 | ENST00000456523 | up | 3.2440311 | 0.0003698 |
| TRPC6 | ENST00000344327 | up | 3.2237037 | 0.001234 |
| ENC1 | ENST00000618628 | up | 3.2225265 | 0.0034672 |
| TK1 | ENST00000301634 | up | 3.2195673 | 9.065E-05 |
| GATSL2 | ENST00000426327 | up | 3.2175217 | 0.0028889 |
| SAA2 | ENST00000414546 | up | 3.2033827 | 1.2E-05 |
| STON1 | ENST00000406226 | up | 3.1929934 | 0.0061938 |
| FBLN2 | ENST00000295760 | up | 3.192849 | 0.0024471 |
| SAPCD2 | ENST00000409687 | up | 3.1919413 | 0.0008587 |
| VCAM1 | ENST00000370119 | up | 3.1717721 | 0.0002155 |
| DPT | ENST00000367817 | up | 3.167888 | 0.0002262 |
| COL3A1 | ENST00000304636 | up | 3.1628841 | 0.0015369 |
| IQGAP3 | ENST00000361170 | up | 3.1564776 | 0.0091802 |
| ZWINT | ENST00000373944 | up | 3.1540586 | 0.0022012 |
| TMEM106A | ENST00000612339 | up | 3.1456646 | 0.0022331 |
| PTGIS | ENST00000244043 | up | 3.1430679 | 0.0005224 |
| OMD | ENST00000375550 | up | 3.1299738 | 0.0026463 |
| FAM43A | ENST00000329759 | up | 3.1296136 | 0.0014534 |
| C3 | ENST00000245907 | up | 3.1183135 | 0.0010571 |
| IGFBP3 | ENST00000613132 | up | 3.1113942 | 0.0006256 |
| PLA2R1 | ENST00000283243 | up | 3.1098565 | 0.0132977 |
| DDX19A | ENST00000302243 | up | 3.0915337 | 0.0028066 |
| MX1 | ENST00000398600 | up | 3.0889653 | 0.0004507 |
| COL10A1 | ENST00000243222 | up | 3.0795709 | 0.0003137 |
| LY6K | ENST00000292430 | up | 3.0775053 | 4.327E-05 |
| ZCCHC5 | ENST00000321110 | up | 3.0692683 | 0.0001078 |
| COL8A1 | ENST00000261037 | up | 3.0640125 | 0.0003808 |
| PDZRN3 | ENST00000492909 | up | 3.0630468 | 0.0067862 |
| TMEM130 | ENST00000339375 | up | 3.0559753 | 0.005973 |
| FAM47A | ENST00000346193 | up | 3.0559749 | 0.0001619 |
| TK1 | ENST00000588734 | up | 3.0498832 | 2.217E-05 |
| KLHL30 | ENST00000409223 | up | 3.0493787 | 0.0076596 |
| MKI67 | ENST00000368653 | up | 3.0400346 | 0.0004704 |
| PRC1 | ENST00000442656 | up | 3.0290784 | 0.0029413 |
| WISP1 | ENST00000220856 | up | 3.0283824 | 0.0035173 |
| SOD2 | ENST00000444946 | up | 3.0271594 | 6.429E-06 |
| COL1A1 | ENST00000225964 | up | 3.0246556 | 0.0007792 |
| SEMA5A | ENST00000382496 | up | 3.0201415 | 0.0033612 |
| SCARA3 | ENST00000337221 | up | 3.0136034 | 0.0059406 |
| ZC3H12A | ENST00000373087 | up | 3.0103446 | 1.718E-05 |
| ST3GAL5 | ENST00000638572 | up | 3.0088227 | 0.0095433 |
| BACE2 | ENST00000347667 | up | 3.0069437 | 0.0005934 |
| SH3D19 | ENST00000514152 | up | 3.0055072 | 0.0014108 |
| CD44 | ENST00000263398 | up | 3.0020753 | 0.0022409 |
| KIF11 | ENST00000260731 | up | 2.9985027 | 0.0006071 |
| ASPN | ENST00000375543 | up | 2.9964674 | 0.0065688 |
| IL32 | ENST00000548652 | up | 2.9894323 | 0.0009088 |
| RNF150 | ENST00000515673 | up | 2.9890452 | 0.0136599 |
| ERCC6 | ENST00000515869 | up | 2.9872972 | 0.0005147 |
| APOBEC3B | ENST00000402182 | up | 2.9857546 | 0.0002869 |
| ITGA8 | ENST00000378076 | up | 2.9856912 | 9.448E-06 |
| B3GALT4 | ENST00000451237 | up | 2.9756209 | 0.0009462 |
| PALLD | ENST00000512127 | up | 2.9737014 | 0.001054 |
| UTY | ENST00000540140 | up | 2.9680437 | 0.0138682 |
| SLA | ENST00000395352 | up | 2.9663825 | 0.0002331 |
| MYBL1 | ENST00000522677 | up | 2.9584544 | 0.0087841 |
| PLCB2 | ENST00000260402 | up | 2.9520184 | 0.0009231 |
| SPAG5 | ENST00000321765 | up | 2.9493589 | 0.0093863 |
| MLLT4 | ENST00000392108 | up | 2.9452501 | 0.0081518 |
| CD9 | ENST00000009180 | up | 2.9302219 | 0.0001637 |
| MXRA5 | ENST00000217939 | up | 2.9294399 | 0.0038072 |
| DEPDC1 | ENST00000370966 | up | 2.9284567 | 0.0070554 |
| AK4 | ENST00000545314 | up | 2.9184386 | 5.781E-05 |
| CENPU | ENST00000281453 | up | 2.9172375 | 0.0003553 |
| AUNIP | ENST00000374298 | up | 2.9105185 | 0.0051598 |
| ARHGEF40 | ENST00000298694 | up | 2.9091135 | 0.0007825 |
| ERCC6 | ENST00000374127 | up | 2.9080297 | 0.0003446 |
| C10orf90 | NM_001350921 | up | 2.9009382 | 0.0030636 |
| CXCL12 | NM_001277990 | up | 2.8927611 | 0.0032442 |
| ERCC6 | ENST00000355832 | up | 2.880823 | 0.0089261 |
| TRAIP | ENST00000331456 | up | 2.8709363 | 0.0011024 |
| SEPT9 | ENST00000423034 | up | 2.8698291 | 0.004256 |
| TUBA1A | ENST00000301071 | up | 2.8648532 | 0.0011712 |
| PREX1 | ENST00000371941 | up | 2.864848 | 0.0023389 |
| SLIT2 | ENST00000504154 | up | 2.8571077 | 0.0145439 |
| HAS3 | ENST00000306560 | up | 2.8495485 | 0.0003669 |
| CENPW | ENST00000368325 | up | 2.8475817 | 6.211E-05 |
| RAMP1 | ENST00000254661 | up | 2.8424111 | 0.0002725 |
| UBE2C | ENST00000243893 | up | 2.8416196 | 0.0060602 |
| PLK1 | ENST00000300093 | up | 2.8408127 | 0.0002823 |
| RGS4 | ENST00000367908 | up | 2.8347375 | 7.841E-05 |
| TFPI2 | ENST00000222543 | up | 2.8314648 | 0.0011243 |
| PAX5 | ENST00000522003 | up | 2.8230328 | 0.0042763 |
| MYO3B | ENST00000408978 | up | 2.8215178 | 0.000953 |
| CTF1 | ENST00000279804 | up | 2.8197939 | 0.0288622 |
| RIT2 | ENST00000326695 | up | 2.8132343 | 0.002387 |
| TFPI2 | NM_001271004 | up | 2.8101289 | 3.145E-05 |
| ATAD5 | ENST00000321990 | up | 2.7987934 | 0.0101375 |
| LMNB1 | ENST00000261366 | up | 2.7977842 | 0.0033589 |
| TBC1D4 | ENST00000377636 | up | 2.7864222 | 0.0100328 |
| GREM2 | ENST00000318160 | up | 2.7681752 | 0.0005204 |
| PDZRN3 | ENST00000462146 | up | 2.7664296 | 0.0022202 |
| CCNB1 | ENST00000256442 | up | 2.7468285 | 0.0011992 |
| EXO1 | ENST00000518483 | up | 2.7452154 | 0.0251994 |
| APPL2 | ENST00000551662 | up | 2.7367074 | 0.0269944 |
| INF2 | ENST00000330634 | up | 2.7311218 | 0.0003607 |
| P2RX2 | ENST00000449132 | up | 2.7308128 | 0.0031884 |
| MYBL1 | ENST00000524176 | up | 2.7302876 | 0.0007006 |
| MOK | ENST00000523231 | up | 2.7297581 | 0.0008043 |
| TLR2 | NM_001318795 | up | 2.7284011 | 0.0013912 |
| TRIOBP | ENST00000646328 | up | 2.7254357 | 0.0012487 |
| ECM2 | ENST00000444490 | up | 2.7252359 | 0.0142419 |
| C14orf180 | ENST00000331952 | up | 2.7205633 | 0.0085986 |
| ARHGAP11A | ENST00000565905 | up | 2.7116398 | 0.0070473 |
| FAM26E | ENST00000368599 | up | 2.7058692 | 0.0156799 |
| PLAU | ENST00000372764 | up | 2.7033785 | 0.0016333 |
| S100A4 | ENST00000354332 | up | 2.690337 | 4.491E-05 |
| SLC7A6 | ENST00000566454 | up | 2.681991 | 0.0038295 |
| TUBA3C | ENST00000400113 | up | 2.6743158 | 0.0006371 |
| APOBEC3B | ENST00000407298 | up | 2.6674658 | 0.0186799 |
| CKAP2 | ENST00000378034 | up | 2.6637763 | 0.0045153 |
| NCEH1 | NM_001146277 | up | 2.6607976 | 0.0191145 |
| CSPG4 | ENST00000308508 | up | 2.6601973 | 0.0012894 |
| ADAMTS12 | ENST00000504830 | up | 2.6520429 | 0.0078253 |
| RALGDS | ENST00000393160 | up | 2.6519468 | 0.0031381 |
| REP15 | ENST00000310791 | up | 2.6492278 | 0.0068027 |
| NPTX2 | ENST00000265634 | up | 2.6308765 | 0.0010759 |
| TM4SF1 | ENST00000305366 | up | 2.6271846 | 0.0018562 |
| UBE2C | ENST00000405520 | up | 2.6241148 | 0.002704 |
| CCNB1 | NM_001354845 | up | 2.6234523 | 0.0021829 |
| CATG00000030168.1 | ENCT00000169304 | up | 2.6222588 | 8.95E-05 |
| SLC1A3 | ENST00000613445 | up | 2.6151101 | 0.0047029 |
| CDK6 | ENST00000424848 | up | 2.6141977 | 0.0002964 |
| KDR | ENST00000645273 | up | 2.6124939 | 0.0023784 |
| SOD2 | ENST00000367054 | up | 2.6036631 | 0.0011882 |
| HCK | ENST00000538448 | up | 2.6020548 | 0.0012055 |
| IL16 | ENST00000394660 | up | 2.5932393 | 0.0042162 |
| PRRT2 | ENST00000358758 | up | 2.5851634 | 0.0021811 |
| MMP7 | ENST00000260227 | up | 2.5836742 | 0.0073038 |
| HSPB3 | ENST00000302005 | up | 2.5836508 | 0.0012527 |
| FSTL1 | ENST00000295633 | up | 2.5824214 | 0.0035478 |
| FAM64A | ENST00000250056 | up | 2.5781889 | 0.0002717 |
| CDC6 | ENST00000209728 | up | 2.5739106 | 0.0040285 |
| NAV2 | ENST00000533917 | up | 2.5735853 | 0.0017355 |
| CRABP2 | ENST00000368222 | up | 2.5657225 | 0.0075452 |
| ERCC6 | NM_001346440 | up | 2.5643247 | 0.0019986 |
| KCNK2 | ENST00000391895 | up | 2.5641841 | 0.0005013 |
| CENPF | ENST00000366955 | up | 2.5591263 | 0.0021491 |
| DST | ENST00000312431 | up | 2.553727 | 0.0115273 |
| HMGA2 | ENST00000393578 | up | 2.5465228 | 0.0296512 |
| PF4V1 | ENST00000226524 | up | 2.544084 | 0.0149248 |
| TAGLN | ENST00000532870 | up | 2.5426161 | 0.0015564 |
| GALNT5 | NM_001329868 | up | 2.536458 | 0.0006083 |
| C13orf45 | ENST00000318245 | up | 2.5338704 | 0.0467913 |
| ATL1 | NM_181598 | up | 2.5336714 | 0.0095773 |
| NUSAP1 | ENST00000414849 | up | 2.5303165 | 0.0035264 |
| SEC14L2 | ENST00000615189 | up | 2.5237714 | 0.0003308 |
| LMCD1 | ENST00000157600 | up | 2.5223587 | 0.0165741 |
| CACNA1G | ENST00000503485 | up | 2.5132968 | 0.0040623 |
| GPR39 | ENST00000329321 | up | 2.5102778 | 0.0001629 |
| RACGAP1 | ENST00000427314 | up | 2.5060587 | 0.0011312 |
| FLI1 | ENST00000527786 | up | 2.5016943 | 0.0008742 |
| MT4 | ENST00000219162 | up | 2.4995319 | 0.0014392 |
| BUD13 | ENST00000260210 | up | 2.4984434 | 0.0004342 |
| SLC50A1 | ENST00000622581 | up | 2.4976393 | 0.0133621 |
| FAM105A | ENST00000274217 | up | 2.4958935 | 0.0048082 |
| BMP1 | ENST00000306349 | up | 2.4937309 | 0.0002264 |
| CLEC3B | ENST00000296130 | up | 2.492937 | 0.001328 |
| DCN | ENST00000547568 | up | 2.4901153 | 0.0044251 |
| AUNIP | ENST00000538789 | up | 2.4857364 | 0.0084009 |
| ARRB1 | ENST00000360025 | up | 2.4853692 | 0.0066057 |
| MCM5 | ENST00000216122 | up | 2.4840562 | 0.0020965 |
| POU2F2 | ENST00000529067 | up | 2.4837796 | 0.0031346 |
| KIF4A | uc010nkw.3 | up | 2.4814813 | 0.0031293 |
| LMCD1 | ENST00000454244 | up | 2.4792037 | 0.0006818 |
| NCEH1 | ENST00000543711 | up | 2.478441 | 0.0133347 |
| PLAT | uc010lxf.1 | up | 2.4760756 | 0.001512 |
| VDR | ENST00000229022 | up | 2.4731905 | 0.0031844 |
| C20orf82 | ENST00000262487 | up | 2.464946 | 0.0132973 |
| MGLL | ENST00000453507 | up | 2.4590278 | 0.0011163 |
| RSPO1 | ENST00000612451 | up | 2.4545544 | 0.0019762 |
| TPX2 | ENST00000300403 | up | 2.4543319 | 0.0001176 |
| THBS1 | ENST00000260356 | up | 2.4499049 | 8.207E-05 |
| L1CAM | ENST00000361981 | up | 2.4469283 | 0.0317696 |
| C11orf91 | ENST00000379011 | up | 2.446538 | 0.0046197 |
| PPFIA2 | NM_001220475 | up | 2.4441758 | 0.0031771 |
| ENC1 | ENST00000302351 | up | 2.4435711 | 0.0074459 |
| CELF5 | ENST00000541430 | up | 2.4333922 | 0.0265407 |
| GALNT5 | ENST00000259056 | up | 2.4209029 | 0.0020349 |
| CENPE | ENST00000380026 | up | 2.4139745 | 0.0007054 |
| SOD2 | ENST00000337404 | up | 2.4100706 | 2.684E-06 |
| SGO1 | NM_001012409 | up | 2.4090769 | 0.0005007 |
| C21orf33 | ENST00000646873 | up | 2.4071434 | 0.0062561 |
| SPC24 | ENST00000592540 | up | 2.4066908 | 0.0200627 |
| KIRREL3 | ENST00000525144 | up | 2.4062862 | 0.002398 |
| KDELR3 | ENST00000409006 | up | 2.4050988 | 0.0001549 |
| FZD1 | ENST00000287934 | up | 2.4023881 | 0.002084 |
| CCNA2 | ENST00000274026 | up | 2.3983015 | 0.0293325 |
| SERPINE1 | ENST00000223095 | up | 2.3932467 | 0.0007484 |
| GALNT15 | ENST00000437509 | up | 2.3794543 | 0.0011321 |
| MMP1 | ENST00000315274 | up | 2.3749903 | 0.0001176 |
| FKBP1B | ENST00000380986 | up | 2.3748379 | 0.004729 |
| NUSAP1 | ENST00000560177 | up | 2.374081 | 0.0005333 |
| NDC80 | ENST00000261597 | up | 2.371696 | 0.0024302 |
| THBD | ENST00000377103 | up | 2.3647861 | 0.000694 |
| NAB1 | ENST00000409581 | up | 2.3603927 | 0.0009203 |
| MEOX1 | ENST00000318579 | up | 2.3588931 | 0.036917 |
| ALDH16A1 | ENST00000293350 | up | 2.3381604 | 0.0001166 |
| KCNMA1 | ENST00000639489 | up | 2.3364165 | 0.0133281 |
| JPH2 | ENST00000342272 | up | 2.3329613 | 0.0229393 |
| CHTF18 | ENST00000262315 | up | 2.3305619 | 0.0008955 |
| BLID | ENST00000560104 | up | 2.3285194 | 0.0034312 |
| FAM167A | ENST00000284486 | up | 2.3272002 | 0.000983 |
| AQP1 | ENST00000311813 | up | 2.3184324 | 0.0253381 |
| NUSAP1 | ENST00000559596 | up | 2.3131168 | 0.0001378 |
| AKAP12 | ENST00000402676 | up | 2.3104953 | 0.0043502 |
| ARHGAP11A | ENST00000361627 | up | 2.3079028 | 0.0012223 |
| HMMR | ENST00000358715 | up | 2.3038586 | 0.0210997 |
| OLFML2A | ENST00000288815 | up | 2.2995563 | 0.0378257 |
| CCDC85B | ENST00000312579 | up | 2.2920117 | 0.0117212 |
| GREM1 | ENST00000560830 | up | 2.2853753 | 0.0011412 |
| TMEM171 | ENST00000287773 | up | 2.2852667 | 0.0072404 |
| PIK3IP1 | ENST00000441972 | up | 2.2847227 | 0.0023253 |
| KIAA1217 | ENST00000396446 | up | 2.278946 | 0.0032284 |
| CBR3 | ENST00000290354 | up | 2.2774864 | 0.0018123 |
| CATG00000040062.1 | MICT00000004709 | up | 2.2681812 | 0.0021039 |
| IBSP | ENST00000226284 | up | 2.2623025 | 0.0052641 |
| SPRY1 | ENST00000610581 | up | 2.2536873 | 0.0032395 |
| SPECC1 | ENST00000395527 | up | 2.2500527 | 0.0018973 |
| EPB41L2 | ENST00000392427 | up | 2.2492294 | 0.0005223 |
| CD44 | ENST00000278386 | up | 2.2488991 | 0.0005364 |
| AKAP12 | ENST00000354675 | up | 2.2477783 | 0.0013681 |
| MTRNR2L5 | ENST00000512524 | up | 2.2456905 | 0.0266302 |
| CATG00000039349.1 | MICT00000176154 | up | 2.2448606 | 0.040672 |
| TCF4 | ENST00000561992 | up | 2.2361399 | 0.0150425 |
| OAS1 | ENST00000551241 | up | 2.2359516 | 0.0038882 |
| KDELR3 | ENST00000216014 | up | 2.231494 | 0.0031296 |
| HFE | ENST00000461397 | up | 2.227456 | 0.0010444 |
| KDM6A | ENST00000377967 | up | 2.2261579 | 0.0050593 |
| CNR1 | ENST00000468898 | up | 2.2234099 | 0.019859 |
| ANG | ENST00000336811 | up | 2.2219486 | 0.008668 |
| SLIT2 | NM_001289135 | up | 2.2211937 | 0.004766 |
| TM4SF1 | ENST00000472441 | up | 2.2053823 | 0.0164041 |
| CTHRC1 | ENST00000330295 | up | 2.2051052 | 0.0002968 |
| AMPD3 | ENST00000396553 | up | 2.1974887 | 0.0003854 |
| DCN | ENST00000420120 | up | 2.1937464 | 0.0001427 |
| NOTCH3 | ENST00000263388 | up | 2.1878587 | 0.0011883 |
| GATA6 | ENST00000269216 | up | 2.1853163 | 0.0324801 |
| IKBIP | ENST00000299157 | up | 2.1844057 | 0.0013257 |
| CEMIP | ENST00000220244 | up | 2.1803944 | 0.0283822 |
| PCLAF | ENST00000380258 | up | 2.1788434 | 0.0126879 |
| CENPM | ENST00000215980 | up | 2.1779095 | 0.0010443 |
| BEST4 | ENST00000372207 | up | 2.1748145 | 0.0497003 |
| DSP | NM_001319034 | up | 2.1737057 | 0.0070395 |
| SEC14L2 | ENST00000402592 | up | 2.170326 | 0.010455 |
| CCNH | ENST00000504878 | up | 2.1568672 | 0.0008554 |
| SGO1 | uc021wtx.1 | up | 2.1531172 | 0.0143709 |
| ZKSCAN3 | ENST00000341464 | up | 2.143769 | 0.0213283 |
| CATG00000018128.1 | FTMT25000007364 | up | 2.1418894 | 0.0179492 |
| PLCB3 | ENST00000540288 | up | 2.1410528 | 0.0144918 |
| HELLS | ENST00000348459 | up | 2.1402349 | 0.0008332 |
| LRFN1 | ENST00000248668 | up | 2.1380807 | 0.04226 |
| CCR1 | ENST00000296140 | up | 2.1320638 | 0.0134012 |
| TRIM9 | ENST00000360392 | up | 2.1222711 | 0.002101 |
| CH25H | ENST00000371852 | up | 2.1176102 | 0.0141301 |
| KIAA1217 | ENST00000376454 | up | 2.1171964 | 0.0020702 |
| CCND2 | ENST00000261254 | up | 2.1048083 | 0.0025794 |
| IGFBP7 | ENST00000514062 | up | 2.1047033 | 0.0290118 |
| KCTD8 | ENST00000360029 | up | 2.0921853 | 0.0003301 |
| PLEKHN1 | ENST00000379410 | up | 2.0920783 | 0.0165335 |
| FGGY | ENST00000303721 | up | 2.0909283 | 0.0003164 |
| FOXM1 | ENST00000361953 | up | 2.0861246 | 0.0366389 |
| COL7A1 | ENST00000328333 | up | 2.0779339 | 0.0032061 |
| TBC1D8B | ENST00000310452 | up | 2.0778489 | 0.0195548 |
| P4HA3 | ENST00000427714 | up | 2.0763759 | 0.0013919 |
| NAV2 | ENST00000349880 | up | 2.0744186 | 0.0005162 |
| SPATA13 | ENST00000424834 | up | 2.0733698 | 0.0057692 |
| LPCAT1 | ENST00000283415 | up | 2.0611 | 0.0410671 |
| LCK | ENST00000333070 | up | 2.0609354 | 0.0314906 |
| RAB15 | ENST00000533601 | up | 2.0581604 | 0.0008718 |
| CCBE1 | ENST00000439986 | up | 2.052361 | 0.0009115 |
| LDHA | ENST00000422447 | up | 2.0485647 | 0.000274 |
| CFD | ENST00000592860 | up | 2.046355 | 0.0006236 |
| PPP1R3G | ENST00000405617 | up | 2.0447202 | 0.0173671 |
| IRAK2 | ENST00000256458 | up | 2.0444718 | 0.0023274 |
| LRRN3 | ENST00000451085 | up | 2.0428202 | 0.0003649 |
| CATG00000050714.1 | FTMT20600011012 | up | 2.0406309 | 0.0353794 |
| PFKFB4 | ENST00000232375 | up | 2.0398192 | 0.0031313 |
| AMPD3 | ENST00000444303 | up | 2.0355602 | 0.0050598 |
| SHANK1 | NM_016148 | up | 2.0346483 | 0.0122137 |
| C6orf132 | ENST00000341865 | up | 2.0345557 | 0.013017 |
| DLGAP5 | ENST00000395425 | up | 2.0337945 | 0.0196275 |
| LIMS2 | ENST00000324938 | up | 2.0224615 | 0.0026846 |
| KIF4B | ENST00000435029 | up | 2.020878 | 0.0081308 |
| POC1A | ENST00000296484 | up | 2.0201941 | 0.0029699 |
| GPM6B | NM_001318729 | up | 2.0176537 | 0.0007028 |
| TNS1 | ENST00000430930 | up | 2.0073477 | 0.0197729 |
| SLC41A2 | ENST00000258538 | up | 2.0055251 | 0.0037422 |
| LMOD1 | ENST00000367288 | up | 1.9980161 | 0.0018323 |
| MOK | ENST00000517966 | up | 1.9957193 | 0.0005276 |
| GRK5 | ENST00000392870 | up | 1.9866543 | 0.0049943 |
| TBCK | ENST00000394706 | up | 1.9835734 | 0.0105804 |
| ANGPTL4 | ENST00000594875 | up | 1.9831334 | 0.0066864 |
| EPB41L2 | NM_001350302 | up | 1.9824962 | 0.0025299 |
| TBXA2R | ENST00000375190 | up | 1.9801843 | 0.0044315 |
| LDHA | ENST00000396222 | up | 1.9800634 | 0.0015762 |
| ARHGAP24 | ENST00000395183 | up | 1.9766016 | 0.0339704 |
| GALNT1 | ENST00000269195 | up | 1.9708519 | 0.0068404 |
| FGD4 | ENST00000427716 | up | 1.9661425 | 0.0032639 |
| TLR2 | ENST00000642580 | up | 1.9612403 | 0.0015115 |
| APAF1 | ENST00000552268 | up | 1.9551688 | 0.0187596 |
| OAS1 | ENST00000202917 | up | 1.945547 | 0.0016977 |
| DIRC1 | ENST00000308100 | up | 1.9415387 | 0.0047684 |
| IL6 | uc011jyq.1 | up | 1.9410878 | 0.0099385 |
| BID | ENST00000399767 | up | 1.9400217 | 0.001033 |
| NCEH1 | ENST00000475381 | up | 1.9383361 | 0.0042415 |
| MCM3 | ENST00000616552 | up | 1.9378841 | 0.0244423 |
| EPB41L2 | ENST00000337057 | up | 1.932612 | 0.0019872 |
| KIAA1524 | ENST00000295746 | up | 1.9314777 | 0.04192 |
| IKBKE | ENST00000581977 | up | 1.9298951 | 0.0020516 |
| MAFK | ENST00000343242 | up | 1.9287423 | 0.005186 |
| OPN3 | ENST00000366554 | up | 1.9247791 | 0.0041588 |
| SORCS2 | ENST00000507866 | up | 1.9246016 | 0.026774 |
| LDHA | ENST00000227157 | up | 1.921757 | 9.438E-05 |
| CYB561A3 | ENST00000447532 | up | 1.919613 | 0.0010813 |
| PDZRN3 | ENST00000263666 | up | 1.9178731 | 0.007801 |
| OGDHL | NM_001347822 | up | 1.9174878 | 0.0218405 |
| SGCD | NM_172244 | up | 1.9165398 | 0.0010488 |
| CD248 | ENST00000311330 | up | 1.9158746 | 0.0037047 |
| COL13A1 | ENST00000645393 | up | 1.9153748 | 0.0210452 |
| FOSL1 | ENST00000448083 | up | 1.9130635 | 0.0074648 |
| ABCA13 | ENST00000435803 | up | 1.9094194 | 0.0039915 |
| SPRY1 | ENST00000339241 | up | 1.9086413 | 0.0114611 |
| UACA | ENST00000322954 | up | 1.9077475 | 0.0155471 |
| OASL | ENST00000620239 | up | 1.9063339 | 0.0114995 |
| DUSP6 | ENST00000308385 | up | 1.9050605 | 0.0039465 |
| PF4 | ENST00000296029 | up | 1.904019 | 0.0067765 |
| DIAPH3 | ENST00000400320 | up | 1.9026424 | 0.0172243 |
| AMN1 | ENST00000537562 | up | 1.9020989 | 0.0189693 |
| ABCA6 | ENST00000284425 | up | 1.900032 | 0.0171521 |
| MAP2K3 | ENST00000361818 | up | 1.8826259 | 0.0134355 |
| NEGR1 | ENST00000357731 | up | 1.8793334 | 0.0171547 |
| ANK1 | ENST00000348036 | up | 1.867652 | 0.0041137 |
| KCNJ15 | ENST00000612702 | up | 1.8671711 | 0.0066784 |
| HIST1H1C | ENST00000343677 | up | 1.8658858 | 0.0004007 |
| SLC7A7 | ENST00000285850 | up | 1.8640747 | 0.0192686 |
| STEAP4 | ENST00000380079 | up | 1.8591129 | 0.0049286 |
| SH3D19 | ENST00000409252 | up | 1.8577481 | 0.0005218 |
| S100A4 | ENST00000368715 | up | 1.8560567 | 0.0071842 |
| ADAM12 | NM_001288973 | up | 1.8524407 | 0.0022497 |
| HCK | ENST00000518730 | up | 1.845752 | 0.0129783 |
| EPB41L2 | ENST00000445890 | up | 1.8446624 | 0.0222354 |
| STMN1 | ENST00000455785 | up | 1.8432165 | 0.0005977 |
| ECM2 | NM_001197295 | up | 1.8398071 | 0.0206619 |
| HELLS | NM_001289073 | up | 1.8394756 | 0.0014121 |
| PRRT4 | ENST00000446477 | up | 1.8330377 | 0.0107521 |
| IGFBP5 | ENST00000233813 | up | 1.8297804 | 0.0038305 |
| TROAP | ENST00000257909 | up | 1.8290937 | 0.0022847 |
| BID | ENST00000551952 | up | 1.828297 | 0.0125329 |
| CD44 | ENST00000442151 | up | 1.8281236 | 0.0007192 |
| NPTXR | ENST00000333039 | up | 1.8270434 | 0.0059932 |
| EVA1C | ENST00000382699 | up | 1.8257721 | 0.0013787 |
| HMGA2 | ENST00000541363 | up | 1.825223 | 0.0012858 |
| NAV2 | NM_182964 | up | 1.8140753 | 0.001891 |
| BACE2 | ENST00000328735 | up | 1.8118913 | 0.0051141 |
| CPSF6 | ENST00000435070 | up | 1.8068452 | 0.0008442 |
| LMCD1 | ENST00000397386 | up | 1.8063473 | 0.000154 |
| CATG00000044530.1 | MICT00000198512 | up | 1.8036552 | 0.0492327 |
| BIRC3 | ENST00000263464 | up | 1.8030855 | 0.0189403 |
| PSMC3IP | ENST00000253789 | up | 1.8015294 | 0.0093487 |
| CATG00000016372.1 | MICT00000099780 | up | 1.7996939 | 0.0343062 |
| FOXM1 | ENST00000627656 | up | 1.7897168 | 0.0137604 |
| ADAMTS7 | ENST00000388820 | up | 1.7891367 | 0.0248815 |
| OAS2 | ENST00000342315 | up | 1.7858963 | 0.0024322 |
| ARNTL2 | ENST00000311001 | up | 1.7831399 | 0.0234411 |
| ZWILCH | ENST00000446801 | up | 1.7751566 | 0.0011719 |
| SYTL4 | ENST00000263033 | up | 1.7737413 | 0.0002382 |
| ECT2 | NM_001349097 | up | 1.7706703 | 0.0005564 |
| FAM114A1 | NM_001350632 | up | 1.7706063 | 0.0001296 |
| ARHGAP11A | ENST00000543522 | up | 1.7681573 | 0.0162286 |
| FMNL3 | ENST00000335154 | up | 1.7655945 | 0.0131513 |
| TLR6 | ENST00000436693 | up | 1.7654805 | 0.00442 |
| MAD2L1 | ENST00000296509 | up | 1.7643528 | 0.0148304 |
| SOD3 | ENST00000382120 | up | 1.760586 | 0.0034438 |
| IKBIP | ENST00000393042 | up | 1.7577133 | 0.0127863 |
| SPRY1 | ENST00000622283 | up | 1.7559523 | 0.0100154 |
| MGLL | ENST00000398104 | up | 1.7556463 | 0.0172407 |
| WFS1 | ENST00000503569 | up | 1.7548303 | 0.0282352 |
| DIAPH3 | ENST00000400324 | up | 1.7522474 | 0.0012554 |
| LKAAEAR1 | ENST00000302096 | up | 1.7487822 | 0.0009474 |
| AX747246 | uc001hxp.1 | up | 1.7487427 | 0.0150133 |
| LRRN3 | ENST00000422987 | up | 1.7483802 | 0.0273423 |
| SGCD | ENST00000337851 | up | 1.748328 | 0.0139181 |
| JCHAIN | ENST00000543780 | up | 1.7475808 | 0.0260133 |
| CTSS | ENST00000448301 | up | 1.7429394 | 0.0221882 |
| NFE2L3 | ENST00000056233 | up | 1.7413793 | 0.0021805 |
| ZBTB16 | ENST00000392996 | up | 1.7307487 | 0.002691 |
| FXYD1 | ENST00000351325 | up | 1.7306125 | 0.0064905 |
| IKBIP | ENST00000342502 | up | 1.7300057 | 0.0070089 |
| TGFB1 | ENST00000221930 | up | 1.7290847 | 0.0020591 |
| PRELP | NM_201348 | up | 1.7273554 | 0.0346993 |
| CGREF1 | ENST00000402394 | up | 1.7265751 | 0.0072556 |
| PALLD | ENST00000505667 | up | 1.7263744 | 0.0072733 |
| PTGER2 | ENST00000245457 | up | 1.722276 | 0.0005472 |
| LTBP2 | ENST00000261978 | up | 1.7211186 | 0.0020106 |
| TACC1 | NM_001352778 | up | 1.7176969 | 0.0388576 |
| CYP7B1 | NM_001324112 | up | 1.7170441 | 0.009302 |
| ELK3 | ENST00000228741 | up | 1.714326 | 0.0009639 |
| INCENP | ENST00000394818 | up | 1.713234 | 0.0013447 |
| LRRN3 | ENST00000308478 | up | 1.7124223 | 0.0023466 |
| MX2 | ENST00000330714 | up | 1.712387 | 0.0133599 |
| GJC1 | ENST00000426548 | up | 1.7119928 | 0.0115334 |
| XXbac-BPG116M5.17 | ENST00000456570 | up | 1.7108468 | 0.0496079 |
| BID | ENST00000317361 | up | 1.7076812 | 0.0038468 |
| ADM | ENST00000528655 | up | 1.7038763 | 0.0012845 |
| RP1-34B20.21 | ENST00000635200 | up | 1.7025236 | 0.0001725 |
| SIT1 | ENST00000259608 | up | 1.7010853 | 0.0103484 |
| HOXC6 | ENST00000243108 | up | 1.6975933 | 0.0063334 |
| CHRNB1 | ENST00000306071 | up | 1.696537 | 0.0349325 |
| VOPP1 | ENST00000418904 | up | 1.6955889 | 0.0371399 |
| CATG00000001354.1 | ENCT00000063592 | up | 1.6947057 | 0.0002091 |
| CCNA2 | ENST00000618014 | up | 1.6925814 | 0.0018289 |
| ISG15 | ENST00000379389 | up | 1.6918945 | 0.001352 |
| TRAF3IP2 | ENST00000340026 | up | 1.6850777 | 0.0026969 |
| SLIT2 | ENST00000503823 | up | 1.6738053 | 0.009093 |
| STMN1 | ENST00000426559 | up | 1.6714825 | 0.0003165 |
| TMEM44 | ENST00000473092 | up | 1.6710522 | 0.0314085 |
| STX1A | ENST00000222812 | up | 1.6627567 | 0.000639 |
| FOXC2 | ENST00000320354 | up | 1.6607298 | 0.0053904 |
| INSC | ENST00000528567 | up | 1.6549968 | 0.0063042 |
| SLC7A6 | ENST00000219343 | up | 1.6536304 | 0.004718 |
| MGAT1 | ENST00000333055 | up | 1.6460747 | 0.0063212 |
| DSN1 | ENST00000373734 | up | 1.645511 | 0.004818 |
| EHBP1L1 | ENST00000309295 | up | 1.6449172 | 0.0132324 |
| MT1E | ENST00000306061 | up | 1.6373965 | 0.0242136 |
| PRR5 | ENST00000336985 | up | 1.6360959 | 0.0498464 |
| CENPW | ENST00000368326 | up | 1.6346566 | 0.0128645 |
| TBC1D4 | ENST00000377625 | up | 1.6339657 | 0.0002948 |
| SEC14L2 | ENST00000617837 | up | 1.6320336 | 0.0004638 |
| LPAR1 | NM_001351404 | up | 1.6299322 | 0.0024112 |
| YPEL3 | ENST00000398838 | up | 1.628238 | 0.0345847 |
| ITGBL1 | ENST00000545560 | up | 1.625814 | 0.0011421 |
| PINK1 | ENST00000321556 | up | 1.6211386 | 0.0003646 |
| MMP19 | ENST00000409200 | up | 1.6197741 | 0.0376569 |
| PSMC3IP | ENST00000393795 | up | 1.6172276 | 0.0029699 |
| SYNGR3 | ENST00000248121 | up | 1.6171455 | 2.389E-06 |
| DENND3 | ENST00000519811 | up | 1.6136393 | 0.0015776 |
| SLC22A4 | ENST00000200652 | up | 1.611293 | 0.0029963 |
| STEAP2 | ENST00000394629 | up | 1.6077621 | 0.0207532 |
| IFI6 | ENST00000362020 | up | 1.6031211 | 0.0104437 |
| IL27RA | ENST00000263379 | up | 1.6024962 | 0.0051279 |
| IFI6 | ENST00000361157 | up | 1.6008967 | 0.000307 |
| WWC1 | ENST00000521089 | up | 1.5968632 | 0.000779 |
| CDT1 | ENST00000301019 | up | 1.5959893 | 0.0007481 |
| GPM6B | ENST00000356942 | up | 1.5957652 | 0.0002734 |
| AC187652.1 | ENST00000599994 | up | 1.5933457 | 0.0058825 |
| MGLL | ENST00000265052 | up | 1.5904675 | 0.0038636 |
| IGFBP7 | ENST00000295666 | up | 1.5895052 | 0.0038743 |
| SAAL1 | ENST00000524803 | up | 1.5886245 | 0.0160481 |
| NAV2 | ENST00000360655 | up | 1.5856247 | 0.0135777 |
| PITPNC1 | ENST00000299954 | up | 1.5798264 | 0.0025611 |
| PDE7B | ENST00000308191 | up | 1.5788783 | 0.03454 |
| TRIM26 | uc010jry.3 | up | 1.5788733 | 0.0117451 |
| MLKL | ENST00000306247 | up | 1.5761271 | 0.0078184 |
| STMN1 | ENST00000399728 | up | 1.5730294 | 0.0216455 |
| MB21D2 | ENST00000392452 | up | 1.5724002 | 0.008968 |
| RIN3 | ENST00000556418 | up | 1.571583 | 0.01633 |
| ITGBL1 | ENST00000376162 | up | 1.5615666 | 0.0043694 |
| ADAM12 | ENST00000368679 | up | 1.5558292 | 0.0006416 |
| BID | ENST00000622694 | up | 1.5540462 | 0.0033952 |
| EDN1 | NM_001168319 | up | 1.5530836 | 0.0264202 |
| SGO2 | NM_001160033 | up | 1.5514353 | 0.0109566 |
| C15orf52 | ENST00000559313 | up | 1.5492629 | 0.0008509 |
| COL2A1 | ENST00000337299 | up | 1.5467198 | 0.024594 |
| ABCA1 | ENST00000374736 | up | 1.5437997 | 0.0086943 |
| ITGBL1 | ENST00000376180 | up | 1.540803 | 0.0019045 |
| APCDD1L | NM_001304787 | up | 1.5372132 | 0.0102997 |
| BID | ENST00000399765 | up | 1.5363552 | 0.0013881 |
| SLC16A6 | ENST00000580666 | up | 1.5336493 | 0.0006948 |
| GPR176 | ENST00000543580 | up | 1.5325661 | 0.0023761 |
| E2F1 | ENST00000343380 | up | 1.5278258 | 0.0303558 |
| ADAM12 | ENST00000368676 | up | 1.5271221 | 0.0040963 |
| HECW2 | ENST00000409111 | up | 1.5256886 | 0.0193786 |
| RBM15B | ENST00000563281 | up | 1.5246026 | 0.0316723 |
| PLAUR | ENST00000601723 | up | 1.5218714 | 0.000564 |
| APAF1 | ENST00000357310 | up | 1.5215196 | 0.0078006 |
| COL5A2 | ENST00000374866 | up | 1.5205722 | 0.0023101 |
| SLC39A7 | ENST00000374677 | up | 1.5188003 | 0.008459 |
| SPDL1 | NM_001329642 | up | 1.5161175 | 0.0154437 |
| ATP13A2 | ENST00000452699 | up | 1.5125705 | 0.0058236 |
| TNNC2 | ENST00000372557 | up | 1.5106937 | 0.0293463 |
| C9orf3 | ENST00000375315 | up | 1.510622 | 0.0004395 |
| SYNC | ENST00000409190 | up | 1.5102491 | 0.006126 |
| PLAUR | ENST00000340093 | up | 1.5046837 | 0.0103702 |
| SEC24D | ENST00000280551 | up | 1.5043936 | 0.0045076 |
| MT1M | ENST00000379818 | up | 1.5043849 | 0.0117767 |
| ACTL10 | ENST00000330271 | up | 1.5039108 | 0.001445 |
| EML1 | ENST00000334192 | up | 1.5027838 | 0.010676 |
| FANCI | ENST00000310775 | up | 1.4983819 | 0.0320067 |
| CAMK2G | ENST00000322635 | up | 1.4964371 | 0.0156721 |
| SGMS2 | ENST00000394684 | up | 1.4957578 | 0.0255503 |
| STAMBPL1 | ENST00000371926 | up | 1.4948336 | 0.0044624 |
| SLC39A7 | NM_001288777 | up | 1.4945907 | 0.0049084 |
| CDC25A | ENST00000351231 | up | 1.4923211 | 0.0070733 |
| NAB1 | ENST00000434473 | up | 1.4905118 | 0.0012124 |
| CST6 | ENST00000312134 | up | 1.4830011 | 0.0181581 |
| CALHM2 | uc001kxd.1 | up | 1.4828758 | 0.0119138 |
| CCDC142 | uc002slp.2 | up | 1.4824085 | 0.0241347 |
| GPR176 | ENST00000561100 | up | 1.4772066 | 0.0073394 |
| DCN | ENST00000052754 | up | 1.476602 | 0.0012024 |
| TMEM100 | ENST00000424486 | up | 1.4753724 | 0.0074988 |
| TMED3 | ENST00000536821 | up | 1.4715075 | 0.0127642 |
| CCDC28B | ENST00000421922 | up | 1.4693522 | 0.0117996 |
| SERPINE1 | uc003uxu.2 | up | 1.4686042 | 0.0040594 |
| C15orf62 | ENST00000344320 | up | 1.4679087 | 0.019161 |
| HMGA2 | ENST00000393577 | up | 1.4663731 | 0.0082505 |
| KIF20B | ENST00000371728 | up | 1.4630161 | 0.0172608 |
| CSHL1 | ENST00000259003 | up | 1.462328 | 0.0355266 |
| CATG00000005989.1 | MICT00000062561 | up | 1.4598287 | 0.0292396 |
| CD44 | ENST00000428726 | up | 1.4594009 | 0.0035806 |
| APOBEC3B | ENST00000333467 | up | 1.4588592 | 0.0103321 |
| SGO2 | ENST00000357799 | up | 1.4588249 | 0.0111016 |
| THY1 | ENST00000528522 | up | 1.4536913 | 0.0027538 |
| SMTN | ENST00000358743 | up | 1.4536252 | 0.0003918 |
| LMO7 | ENST00000465261 | up | 1.4527321 | 0.0221746 |
| IL6R | ENST00000368485 | up | 1.4512733 | 0.0275104 |
| PRR5 | ENST00000617066 | up | 1.4461416 | 0.0126861 |
| EID3 | ENST00000527879 | up | 1.4399789 | 0.0054643 |
| PGM2L1 | ENST00000298198 | up | 1.4383264 | 0.0066007 |
| APCDD1L | ENST00000371149 | up | 1.4356689 | 0.001932 |
| PRR15 | NM_001329996 | up | 1.4351264 | 0.0059397 |
| DDAH1 | ENST00000284031 | up | 1.433449 | 0.0151211 |
| NPDC1 | ENST00000371601 | up | 1.4330826 | 0.016578 |
| LOXL4 | ENST00000260702 | up | 1.4287505 | 0.0038765 |
| DUSP4 | ENST00000240100 | up | 1.4280449 | 0.0093097 |
| STEAP2 | ENST00000394621 | up | 1.4271912 | 0.0219516 |
| IFI44 | uc010orr.1 | up | 1.4235711 | 0.0143737 |
| APOLD1 | ENST00000326765 | up | 1.4173402 | 0.000863 |
| CDK2 | ENST00000266970 | up | 1.4158968 | 0.0056693 |
| NTM | ENST00000425719 | up | 1.415215 | 0.0018577 |
| CDKN3 | ENST00000556102 | up | 1.4119115 | 0.0016735 |
| CCDC107 | ENST00000378409 | up | 1.4116482 | 0.0006735 |
| DKK3 | NM_001018057 | up | 1.4108502 | 0.0046021 |
| ANXA2 | ENST00000396024 | up | 1.4065898 | 0.019873 |
| GALNT2 | ENST00000366672 | up | 1.4036214 | 0.0071768 |
| CD44 | ENST00000434472 | up | 1.3980578 | 0.0018784 |
| PITPNC1 | ENST00000580974 | up | 1.3952167 | 0.0120951 |
| RP11-507K12.1 | ENST00000637807 | up | 1.394848 | 0.0141541 |
| TMEM87B | ENST00000283206 | up | 1.3925137 | 0.0289951 |
| CATG00000110054.1 | HBMT00001496451 | up | 1.3910006 | 0.0012876 |
| STARD13 | NM_001243476 | up | 1.3898724 | 0.0237319 |
| LIG1 | ENST00000263274 | up | 1.3866264 | 0.0128785 |
| FGD4 | ENST00000546442 | up | 1.385022 | 0.0048846 |
| AKAP12 | ENST00000253332 | up | 1.3817405 | 0.0101103 |
| TRAF2 | ENST00000247668 | up | 1.3815718 | 0.0281903 |
| PML | ENST00000565898 | up | 1.3814125 | 0.0295796 |
| SMTN | ENST00000347557 | up | 1.3786364 | 5.258E-05 |
| CDKN3 | ENST00000335183 | up | 1.3784793 | 0.0041349 |
| SRPX2 | ENST00000373004 | up | 1.3771249 | 0.0023708 |
| TUBB | NM_001293216 | up | 1.3771092 | 0.002672 |
| ITGA11 | ENST00000315757 | up | 1.3734173 | 0.0282937 |
| POLA2 | ENST00000265465 | up | 1.3722018 | 0.0304035 |
| AC037459.4 | ENST00000450780 | up | 1.3718547 | 0.0069807 |
| CDC25A | ENST00000302506 | up | 1.3716307 | 0.0125026 |
| SAMD9 | ENST00000620985 | up | 1.3712832 | 0.0240194 |
| GYS1 | ENST00000263276 | up | 1.3712573 | 0.0448027 |
| CRMP1 | ENST00000397890 | up | 1.3670736 | 0.0313947 |
| ALDH18A1 | NM_001323415 | up | 1.36225 | 0.0111945 |
| PHACTR2 | ENST00000440869 | up | 1.3598968 | 0.0075161 |
| P3H1 | ENST00000397054 | up | 1.3586434 | 0.0024211 |
| SH3D19 | ENST00000409598 | up | 1.3544349 | 0.0136531 |
| LPAR1 | ENST00000374431 | up | 1.3535633 | 0.013142 |
| HIST2H2BE | ENST00000369155 | up | 1.3526932 | 0.0357574 |
| DSP | ENST00000379802 | up | 1.3500492 | 0.0257329 |
| TDP1 | ENST00000393454 | up | 1.3473842 | 0.0064804 |
| S1PR2 | ENST00000646641 | up | 1.3463656 | 0.0165983 |
| IFI6 | ENST00000339145 | up | 1.3461211 | 0.0106569 |
| ITGAV | ENST00000374907 | up | 1.3449139 | 0.0030351 |
| TBC1D8B | ENST00000357242 | up | 1.3429928 | 0.0028837 |
| TPST2 | ENST00000338754 | up | 1.3427527 | 0.0039209 |
| MMP3 | ENST00000299855 | up | 1.3395253 | 0.0061362 |
| TUBB | ENST00000396389 | up | 1.3362337 | 4.17E-05 |
| BORA | ENST00000390667 | up | 1.3332372 | 0.0083324 |
| SLC39A7 | ENST00000374675 | up | 1.332045 | 0.0021609 |
| CASP10 | ENST00000360132 | up | 1.3309678 | 0.0021367 |
| ITGB5 | NM_001354765 | up | 1.3308229 | 0.0011117 |
| AHNAK | ENST00000378024 | up | 1.327749 | 0.0067424 |
| CLCF1 | ENST00000312438 | up | 1.3257894 | 0.0211216 |
| DOCK10 | ENST00000258390 | up | 1.3249354 | 0.032386 |
| APPL2 | ENST00000258530 | up | 1.3239853 | 0.0025815 |
| TUBB | ENST00000330914 | up | 1.3214322 | 0.0041384 |
| RP4-541C22.8 | ENST00000534312 | up | 1.320401 | 0.0016381 |
| MT1HL1 | ENST00000464121 | up | 1.3137935 | 0.0343185 |
| TXNDC15 | ENST00000358387 | up | 1.312565 | 0.0075263 |
| NIPAL3 | ENST00000374399 | up | 1.3119161 | 0.0271801 |
| DOK4 | ENST00000340099 | up | 1.3106102 | 0.0103789 |
| JUN | ENST00000371222 | up | 1.3085083 | 0.002386 |
| SLC16A3 | ENST00000619321 | up | 1.3084155 | 0.0398471 |
| EIF2AK1 | NM_001134335 | up | 1.3082538 | 0.010987 |
| TRIOBP | ENST00000407319 | up | 1.3066611 | 0.0136453 |
| CD44 | ENST00000352818 | up | 1.3054108 | 0.0007472 |
| IFNAR1 | ENST00000270139 | up | 1.3049216 | 0.0161319 |
| KIAA1217 | ENST00000307544 | up | 1.3030619 | 0.004454 |
| ANK1 | ENST00000347528 | up | 1.3011632 | 0.0081251 |
| SFRP2 | ENST00000274063 | up | 1.3003529 | 0.0159085 |
| CNGA3 | ENST00000436404 | up | 1.299501 | 0.0108145 |
| NTM | NM_001352002 | up | 1.2978781 | 0.0002677 |
| DBN1 | ENST00000309007 | up | 1.2967786 | 0.0090531 |
| TRAF3IP2 | ENST00000368735 | up | 1.2952477 | 0.0069733 |
| TSPO2 | ENST00000373161 | up | 1.2951465 | 0.0324659 |
| NEXN | ENST00000334785 | up | 1.2946407 | 0.006639 |
| MYD88 | ENST00000495303 | up | 1.2937757 | 0.0037059 |
| LEPR | ENST00000349533 | up | 1.2912581 | 0.012068 |
| CATG00000080562.1 | ENCT00000360106 | up | 1.288181 | 0.0046293 |
| PBX3 | ENST00000373489 | up | 1.285966 | 0.0119059 |
| WDR76 | ENST00000263795 | up | 1.2856276 | 0.0040394 |
| CD59 | ENST00000426650 | up | 1.2846104 | 0.0251858 |
| XXYLT1 | ENST00000429994 | up | 1.283201 | 0.0153519 |
| CARD6 | ENST00000254691 | up | 1.2825069 | 0.0138431 |
| SYNC | ENST00000373484 | up | 1.2806375 | 0.0057703 |
| FST | ENST00000396947 | up | 1.2804911 | 0.0064945 |
| SYNJ2 | ENST00000355585 | up | 1.2773253 | 0.002878 |
| NIPAL2 | ENST00000430223 | up | 1.2754203 | 0.0302718 |
| PI4KB | ENST00000368872 | up | 1.2682474 | 0.0257817 |
| SLC5A3 | ENST00000381151 | up | 1.2672186 | 0.0480795 |
| GBP2 | ENST00000370466 | up | 1.267022 | 0.0120255 |
| AOX1 | ENST00000374700 | up | 1.264653 | 0.0139828 |
| GMFG | ENST00000601387 | up | 1.2645111 | 0.0305691 |
| AHNAK | ENST00000257247 | up | 1.2639344 | 0.0182915 |
| KIF23 | ENST00000352331 | up | 1.2630471 | 0.0136421 |
| MX1 | ENST00000619682 | up | 1.2598449 | 0.0389135 |
| CAV1 | ENST00000614113 | up | 1.258522 | 0.0174305 |
| SLC43A2 | NM_001321364 | up | 1.2577641 | 0.012428 |
| STEAP1B | ENST00000404369 | up | 1.2574194 | 0.0045121 |
| IRF3 | ENST00000601291 | up | 1.2566144 | 0.00648 |
| C12orf75 | ENST00000443585 | up | 1.2506333 | 0.0008528 |
| CTGF | ENST00000367976 | up | 1.248065 | 0.0002347 |
| C19orf12 | ENST00000392276 | up | 1.2475269 | 0.0114747 |
| CRYAB | ENST00000616970 | up | 1.2465205 | 0.0148193 |
| FOSL1 | ENST00000312562 | up | 1.2464179 | 0.0006377 |
| MAGI3 | ENST00000307546 | up | 1.2461167 | 0.024343 |
| TNFAIP6 | ENST00000243347 | up | 1.2457359 | 0.000898 |
| CYB561 | ENST00000360793 | up | 1.2448859 | 0.0008777 |
| COL4A1 | ENST00000375820 | up | 1.2446162 | 0.0064435 |
| HMGA2 | ENST00000354636 | up | 1.2443237 | 0.0008198 |
| CDKN3 | ENST00000541304 | up | 1.2433283 | 0.0200586 |
| ZBTB47 | ENST00000232974 | up | 1.2431802 | 0.0243356 |
| WTAP | ENST00000337387 | up | 1.2429611 | 0.0049953 |
| ATL1 | ENST00000358385 | up | 1.2391544 | 0.017002 |
| DYNC1I1 | ENST00000359388 | up | 1.2360204 | 0.0245847 |
| C17orf62 | ENST00000434650 | up | 1.2338455 | 0.0006101 |
| CATG00000076265.1 | ENCT00000345803 | up | 1.2329413 | 0.0233222 |
| LHFP | ENST00000379589 | up | 1.2314063 | 0.008657 |
| SEMA4C | ENST00000305476 | up | 1.2294668 | 0.0466707 |
| SEMA4C | ENST00000305476 | up | 1.2294668 | 0.0466707 |
| PITPNC1 | ENST00000581322 | up | 1.2235086 | 0.0048867 |
| LURAP1 | ENST00000371980 | up | 1.2224783 | 0.004805 |
| KIFC3 | NM_001318711 | up | 1.2221602 | 0.0370908 |
| MND1 | ENST00000622785 | up | 1.2221306 | 0.0021552 |
| PYCRL | ENST00000220966 | up | 1.2185651 | 0.0037058 |
| KIAA1644 | ENST00000381176 | up | 1.2173063 | 0.0012787 |
| FOXF2 | ENST00000645481 | up | 1.2169544 | 0.0263625 |
| SHISA2 | ENST00000319420 | up | 1.2167454 | 0.0067087 |
| TOX | ENST00000361421 | up | 1.2162063 | 0.0380545 |
| TLCD1 | ENST00000292090 | up | 1.2158434 | 0.0014054 |
| CCDC107 | ENST00000426546 | up | 1.2137807 | 0.0030841 |
| LMO7 | ENST00000377534 | up | 1.2133261 | 0.0056326 |
| LEPR | ENST00000371059 | up | 1.2114068 | 0.0023539 |
| KDR | ENST00000263923 | up | 1.2087874 | 0.0469409 |
| TMPO | ENST00000556029 | up | 1.2078817 | 0.02469 |
| PRR16 | ENST00000407149 | up | 1.2076251 | 0.0036577 |
| STK17A | ENST00000319357 | up | 1.2046523 | 0.0158093 |
| WNT5A | ENST00000264634 | up | 1.2042659 | 0.0009169 |
| HMGN3 | ENST00000620514 | up | 1.204027 | 0.0059972 |
| BCOR | ENST00000378444 | up | 1.2036588 | 0.0200781 |
| RECK | NM_001316345 | up | 1.20008 | 0.0144907 |
| FOXF2 | ENST00000259806 | up | 1.1991711 | 0.0433767 |
| FGFRL1 | ENST00000510644 | up | 1.1973111 | 0.0109352 |
| KRTAP2-2 | ENST00000398477 | up | 1.1953787 | 0.0128988 |
| ANPEP | ENST00000300060 | up | 1.19488 | 0.0083353 |
| DIP2A | ENST00000457905 | up | 1.1945483 | 0.0263669 |
| SMTN | ENST00000333137 | up | 1.19446 | 0.010148 |
| COL5A1 | ENST00000618395 | up | 1.1927359 | 0.0175949 |
| TUBB6 | ENST00000317702 | up | 1.1919292 | 0.0409922 |
| CKAP2 | ENST00000378037 | up | 1.191476 | 0.0491642 |
| HSPB2 | ENST00000304298 | up | 1.1867318 | 0.0003846 |
| TRAF3IP2 | ENST00000368761 | up | 1.1843855 | 0.0370757 |
| KIR3DL2 | uc002qhl.4 | up | 1.1837804 | 0.0112813 |
| LASP1 | ENST00000433206 | up | 1.1833571 | 0.0037009 |
| GALNT1 | uc002kyz.4 | up | 1.179633 | 0.0065659 |
| EML1 | ENST00000262233 | up | 1.179357 | 0.0048408 |
| CASP7 | ENST00000621345 | up | 1.1774693 | 0.0404268 |
| KIF4A | ENST00000374403 | up | 1.1742626 | 0.0167825 |
| SPARCL1 | ENST00000503414 | up | 1.173529 | 0.0047591 |
| CPEB4 | ENST00000522336 | up | 1.1734667 | 0.0112522 |
| OGDHL | ENST00000374103 | up | 1.1699796 | 0.0300803 |
| GSN | ENST00000394353 | up | 1.169466 | 0.0246491 |
| BID | ENST00000399774 | up | 1.1682293 | 0.0043451 |
| SEC14L2 | ENST00000405717 | up | 1.1675077 | 0.0104329 |
| AC004381.6 | ENST00000261377 | up | 1.1674739 | 0.0117532 |
| ZSWIM4 | ENST00000254323 | up | 1.1665193 | 0.0139464 |
| DLL1 | ENST00000366756 | up | 1.1663345 | 0.0457385 |
| ITGB5 | ENST00000296181 | up | 1.1647999 | 0.0146492 |
| ABHD14A | ENST00000458031 | up | 1.1647788 | 0.0094843 |
| KCNE5 | ENST00000372101 | up | 1.1627812 | 0.0197296 |
| SGCB | ENST00000381431 | up | 1.1626879 | 0.0103136 |
| IQCD | ENST00000416617 | up | 1.1603498 | 0.002747 |
| PHF19 | ENST00000419155 | up | 1.1575855 | 0.0242732 |
| KCTD12 | ENST00000377474 | up | 1.1566424 | 0.0257484 |
| CATG00000057034.1 | FTMT28100003988 | up | 1.1560641 | 0.0129732 |
| MAPKBP1 | ENST00000456763 | up | 1.1514661 | 0.0245597 |
| TMEM189 | ENST00000371652 | up | 1.1510347 | 0.0184874 |
| SIL1 | ENST00000394817 | up | 1.146433 | 0.0220264 |
| PRRC1 | ENST00000442138 | up | 1.1443836 | 0.0102825 |
| TUBB6 | ENST00000590103 | up | 1.1432079 | 0.0254157 |
| TBCK | ENST00000361687 | up | 1.1427798 | 0.0083687 |
| TNIP1 | ENST00000524280 | up | 1.1426547 | 0.0145787 |
| NAB1 | ENST00000337386 | up | 1.1412907 | 0.0219781 |
| SMURF2 | ENST00000262435 | up | 1.1385307 | 0.0283859 |
| LPAR1 | ENST00000358883 | up | 1.1376512 | 0.0134999 |
| LEPR | ENST00000616738 | up | 1.1356537 | 0.0126766 |
| ABR | ENST00000291107 | up | 1.1328034 | 0.0481318 |
| PTGS1 | ENST00000614910 | up | 1.1320404 | 0.005496 |
| ZBTB42 | ENST00000342537 | up | 1.1311878 | 0.0255556 |
| TUBB4B | ENST00000340384 | up | 1.1299772 | 0.0022771 |
| JAG1 | ENST00000254958 | up | 1.1299442 | 0.0130974 |
| HMGN3 | NM_001201362 | up | 1.1282791 | 0.0113378 |
| FEN1 | ENST00000305885 | up | 1.127251 | 0.0222276 |
| CATG00000072318.1 | HBMT00001081968 | up | 1.1268029 | 0.012702 |
| CHST7 | ENST00000276055 | up | 1.1224721 | 0.0235744 |
| CATG00000068640.1 | ENCT00000319213 | up | 1.1213221 | 0.0221204 |
| LUM | ENST00000266718 | up | 1.1204734 | 0.0144255 |
| CAMK2N1 | ENST00000375078 | up | 1.1199594 | 0.0002682 |
| KCNMA1 | NM_001322839 | up | 1.1187935 | 0.0314318 |
| C2orf88 | ENST00000340623 | up | 1.1182163 | 0.0397658 |
| SH3D19 | ENST00000304527 | up | 1.1179977 | 0.0091492 |
| TRIP13 | ENST00000166345 | up | 1.1179493 | 0.0094284 |
| STARD13 | ENST00000336934 | up | 1.1170324 | 0.0388737 |
| CYR61 | ENST00000451137 | up | 1.1168939 | 0.0095497 |
| CASP8 | ENST00000392258 | up | 1.1155052 | 0.0303471 |
| VOPP1 | ENST00000285279 | up | 1.1149094 | 0.0077008 |
| TIMP1 | ENST00000218388 | up | 1.1131049 | 0.010177 |
| ADAD2 | ENST00000268624 | up | 1.1106432 | 0.0463266 |
| KIAA1217 | ENST00000376452 | up | 1.1091728 | 0.0419981 |
| CATG00000038184.1 | ENCT00000200335 | up | 1.1084306 | 0.0109611 |
| RNF183 | ENST00000478815 | up | 1.1076882 | 0.0036605 |
| CCDC190 | ENST00000367912 | up | 1.1072325 | 0.0259135 |
| ALDH1A3 | ENST00000329841 | up | 1.0944425 | 0.0079685 |
| CD59 | ENST00000415002 | up | 1.0941702 | 0.0071478 |
| ARHGAP11A | ENST00000567348 | up | 1.092965 | 0.0406731 |
| RASA3 | ENST00000334062 | up | 1.0920693 | 0.0090563 |
| CALU | ENST00000542996 | up | 1.0917136 | 0.0210461 |
| HERC6 | ENST00000380265 | up | 1.0914729 | 0.0330889 |
| ABI3BP | ENST00000471714 | up | 1.0885662 | 0.0069614 |
| OAS1 | ENST00000445409 | up | 1.0876242 | 0.0033817 |
| CATG00000101027.1 | HBMT00001402195 | up | 1.0874092 | 0.0274882 |
| RPS14 | ENST00000312037 | up | 1.0870691 | 0.0421157 |
| KANK2 | NM_001329451 | up | 1.0867748 | 0.0146624 |
| ADAMTS1 | ENST00000284984 | up | 1.0867036 | 0.0118594 |
| TRNP1 | ENST00000522111 | up | 1.0854537 | 0.0040183 |
| XXYLT1 | ENST00000310380 | up | 1.085368 | 0.0246232 |
| SLC16A3 | ENST00000392339 | up | 1.0850175 | 0.0463291 |
| UBTF | ENST00000436088 | up | 1.084593 | 0.0075376 |
| SPIN4 | ENST00000374884 | up | 1.0820188 | 0.0420385 |
| MBOAT7 | ENST00000391754 | up | 1.0815623 | 0.0270164 |
| ATP8B2 | ENST00000368489 | up | 1.0811873 | 0.021064 |
| DOCK4 | ENST00000437633 | up | 1.0808261 | 0.0154861 |
| DNMT3B | ENST00000443239 | up | 1.0804723 | 0.0393158 |
| HIPK2 | ENST00000428878 | up | 1.0793919 | 0.0084439 |
| FANCM | ENST00000556036 | up | 1.0789307 | 0.0471736 |
| CENPN | ENST00000393335 | up | 1.0778006 | 0.0177771 |
| DYNC1LI2 | ENST00000443351 | up | 1.0764011 | 0.0078286 |
| TIAM1 | ENST00000286827 | up | 1.0721719 | 0.0376023 |
| NFKBIA | ENST00000216797 | up | 1.0698952 | 0.0389719 |
| OLFM1 | ENST00000252854 | up | 1.0694753 | 0.0446193 |
| RRM1 | ENST00000300738 | up | 1.0694035 | 0.02118 |
| ITSN1 | ENST00000399352 | up | 1.0688131 | 0.0189761 |
| CKAP2 | ENST00000258607 | up | 1.06526 | 0.0155578 |
| EHF | ENST00000450654 | up | 1.0641661 | 0.017102 |
| TMEM263 | ENST00000548125 | up | 1.0619634 | 0.0041289 |
| DIP2A | ENST00000435722 | up | 1.0603864 | 0.019909 |
| PMP22 | ENST00000644020 | up | 1.0582558 | 0.0216882 |
| MIER2 | ENST00000264819 | up | 1.0570516 | 0.0225197 |
| LSM11 | ENST00000286307 | up | 1.0567622 | 0.038875 |
| NME4 | ENST00000621774 | up | 1.056087 | 0.0432182 |
| ATP8B1 | ENST00000283684 | up | 1.0539559 | 0.0402045 |
| WTAP | ENST00000614346 | up | 1.0507839 | 0.0090287 |
| TRABD2A | ENST00000409520 | up | 1.0507283 | 0.0040087 |
| SYNDIG1L | ENST00000554823 | up | 1.0505906 | 0.0207188 |
| CD44 | ENST00000433892 | up | 1.0500041 | 0.0010789 |
| CSAR2 | ENST00000595464 | up | 1.0477499 | 0.0119711 |
| SAMD4A | ENST00000251091 | up | 1.0441306 | 0.0257704 |
| TRIM7 | ENST00000334421 | up | 1.0432577 | 0.0007248 |
| GPX8 | ENST00000503787 | up | 1.0430421 | 0.0034733 |
| SOWAHD | ENST00000343905 | up | 1.0428732 | 0.0118758 |
| IRAK3 | ENST00000457197 | up | 1.0420866 | 0.0176869 |
| MYD88 | ENST00000424893 | up | 1.0377525 | 0.0111623 |
| FAM92B | ENST00000539556 | up | 1.0372369 | 0.0447454 |
| PRRC1 | ENST00000512635 | up | 1.0370115 | 0.0409567 |
| CD59 | ENST00000351554 | up | 1.0363671 | 0.0246926 |
| SPTAN1 | ENST00000372739 | up | 1.0356742 | 0.0191988 |
| CATG00000031483.1 | FTMT26800002387 | up | 1.0335526 | 0.0361945 |
| BTN3A3 | ENST00000490254 | up | 1.0327473 | 0.0053442 |
| ARNTL2 | ENST00000544915 | up | 1.0322346 | 0.0189152 |
| SPTLC2 | ENST00000216484 | up | 1.0316486 | 0.0251309 |
| GNG11 | ENST00000248564 | up | 1.0314939 | 0.0241334 |
| FKBPL | ENST00000375156 | up | 1.0310452 | 0.0227677 |
| TSHZ3 | ENST00000240587 | up | 1.0307954 | 0.0470559 |
| ZBTB12 | ENST00000375527 | up | 1.0298708 | 0.0269236 |
| GPR150 | ENST00000380007 | up | 1.0286786 | 0.0312571 |
| SLC44A1 | ENST00000374723 | up | 1.0266511 | 0.0053362 |
| YWHAH | ENST00000248975 | up | 1.0242989 | 0.0460061 |
| P4HA1 | ENST00000440381 | up | 1.0240918 | 0.0150816 |
| AP3B1 | ENST00000255194 | up | 1.0214643 | 0.0162155 |
| FKBP11 | ENST00000552878 | up | 1.0204301 | 0.0070052 |
| HIST1H4A | ENST00000617569 | up | 1.0200706 | 0.0238712 |
| YWHAH | ENST00000397492 | up | 1.0172366 | 0.0396615 |
| SLC16A3 | ENST00000582743 | up | 1.0153823 | 0.0202771 |
| CENPN | ENST00000305850 | up | 1.0138427 | 0.0309855 |
| SMC1A | ENST00000322213 | up | 1.0096318 | 0.010125 |
| BTN3A2 | ENST00000527422 | up | 1.0095129 | 0.0127724 |
| TM4SF19 | NM_001204897 | up | 1.0046162 | 0.0344409 |
| C16orf59 | uc031qur.1 | up | 1.004605 | 0.0476118 |
| BICD1 | ENST00000548411 | up | 1.0024765 | 0.0081698 |
| ELAC2 | ENST00000426905 | up | 0.9986884 | 0.0025051 |
| CAMKMT | ENST00000378494 | up | 0.9982981 | 0.0296471 |
| RDX | ENST00000405097 | up | 0.99639 | 0.0184098 |
| TMEM95 | ENST00000576060 | up | 0.9950166 | 0.0009902 |
| PLOD2 | ENST00000360060 | up | 0.9938875 | 0.0150194 |
| VOPP1 | ENST00000428648 | up | 0.9933572 | 0.0065543 |
| CRYAB | ENST00000533280 | up | 0.9930944 | 0.0030987 |
| PFN1 | ENST00000225655 | up | 0.9925895 | 0.0047952 |
| RAB3D | ENST00000222120 | up | 0.9920971 | 0.0326599 |
| CCDC107 | ENST00000327351 | up | 0.9920583 | 0.0004471 |
| KCNK1 | ENST00000366621 | up | 0.9901539 | 0.0133303 |
| GMEB1 | ENST00000294409 | up | 0.9862113 | 0.0195299 |
| EGR1 | ENST00000239938 | up | 0.9855928 | 0.0361476 |
| MOXD1 | ENST00000367963 | up | 0.9837628 | 0.0129613 |
| AMOTL1 | ENST00000317829 | up | 0.983205 | 0.023903 |
| BTN3A3 | ENST00000244519 | up | 0.9829594 | 0.004947 |
| FOSL1 | NM_001300856 | up | 0.9823979 | 0.0017343 |
| TYMS | ENST00000323274 | up | 0.9820825 | 0.006816 |
| TUBG1 | ENST00000251413 | up | 0.9817996 | 0.047457 |
| POU3F3 | ENST00000361360 | up | 0.9797269 | 0.043083 |
| ARNTL2 | ENST00000261178 | up | 0.9785014 | 0.0225233 |
| KCNMA1 | ENST00000639406 | up | 0.9783085 | 0.0062702 |
| WTAP | ENST00000631126 | up | 0.9764729 | 0.0104017 |
| TMPO | ENST00000393053 | up | 0.9745087 | 0.01809 |
| CATG00000000695.1 | FTMT23800005389 | up | 0.9740946 | 0.0373177 |
| MME | ENST00000492661 | up | 0.9733517 | 0.047811 |
| FUOM | ENST00000278025 | up | 0.9731781 | 0.0098733 |
| GPX8 | ENST00000515370 | up | 0.9722286 | 0.032548 |
| ITGA5 | ENST00000293379 | up | 0.9719253 | 0.0179676 |
| SOCS1 | ENST00000332029 | up | 0.9709238 | 0.0226359 |
| PLOD2 | ENST00000282903 | up | 0.9699508 | 0.0033507 |
| SMAD3 | ENST00000327367 | up | 0.9692831 | 0.0147313 |
| ALDH18A1 | NM_001323413 | up | 0.968715 | 0.0179695 |
| DTYMK | ENST00000305784 | up | 0.9664167 | 0.0058836 |
| RGS3 | ENST00000342620 | up | 0.9653989 | 0.0227496 |
| ARRDC2 | ENST00000222250 | up | 0.9644343 | 0.0133939 |
| PNP | ENST00000361505 | up | 0.9620473 | 0.0265443 |
| CACNB2 | ENST00000645287 | up | 0.9611454 | 0.00222 |
| PNMA2 | ENST00000522362 | up | 0.9591428 | 0.0339342 |
| TMSB10 | ENST00000233143 | up | 0.9590273 | 0.0120563 |
| TMPO | ENST00000343315 | up | 0.95331 | 0.0256852 |
| CTXN1 | ENST00000318978 | up | 0.952316 | 0.005224 |
| PODNL1 | ENST00000645954 | up | 0.9510647 | 0.0137842 |
| MAML2 | ENST00000524717 | up | 0.9479476 | 0.0303122 |
| KBTBD13 | ENST00000432196 | up | 0.9470355 | 0.0149639 |
| CDK2AP1 | ENST00000535979 | up | 0.945728 | 0.0044402 |
| CHAF1A | ENST00000301280 | up | 0.9454733 | 0.0123294 |
| ARNT2 | ENST00000303329 | up | 0.9441725 | 0.0437869 |
| PRKD3 | ENST00000234179 | up | 0.9439589 | 0.0263172 |
| ATP13A2 | ENST00000326735 | up | 0.9437192 | 0.0004824 |
| ABCC10 | ENST00000372530 | up | 0.9425161 | 0.0463532 |
| CATG00000034676.1 | HBMT00000637906 | up | 0.9412436 | 0.0291402 |
| CKS1B | ENST00000308987 | up | 0.9403364 | 0.0104655 |
| STIM2 | ENST00000467087 | up | 0.9394481 | 0.0128317 |
| GK5 | ENST00000392993 | up | 0.9375012 | 0.0299206 |
| SMAD7 | ENST00000589634 | up | 0.9366671 | 0.0085395 |
| RNF145 | ENST00000519865 | up | 0.9353507 | 0.0357695 |
| AK5 | ENST00000354567 | up | 0.9352634 | 0.0127948 |
| SEMA6D | ENST00000558014 | up | 0.9342103 | 0.0450727 |
| TMEM184B | ENST00000633438 | up | 0.9327659 | 0.0297632 |
| A4GALT | ENST00000642412 | up | 0.9293696 | 0.0108716 |
| EFNA3 | ENST00000368408 | up | 0.9292547 | 0.0280665 |
| ARL6IP5 | ENST00000273258 | up | 0.9288502 | 0.0316526 |
| BTN3A1 | ENST00000414912 | up | 0.9278026 | 0.0215098 |
| TMEM151A | ENST00000327259 | up | 0.9275188 | 0.0059695 |
| RP11-134F2.8 | ENST00000418776 | up | 0.9263448 | 0.0071048 |
| P4HA1 | ENST00000307116 | up | 0.9257481 | 0.0224981 |
| GFPT2 | ENST00000253778 | up | 0.9252105 | 0.0050343 |
| NES | ENST00000368223 | up | 0.923603 | 0.0303623 |
| PRIM1 | ENST00000338193 | up | 0.9232578 | 0.0264796 |
| BTN3A3 | ENST00000361232 | up | 0.9230433 | 0.0235265 |
| RNF144B | ENST00000259939 | up | 0.9218723 | 0.0031758 |
| S100A16 | ENST00000368706 | up | 0.9212534 | 0.0068649 |
| SOCS5 | ENST00000394861 | up | 0.9194397 | 0.0169518 |
| DOCK5 | ENST00000410074 | up | 0.9183521 | 0.029784 |
| SLC30A6 | ENST00000435660 | up | 0.9169297 | 0.0037148 |
| ZFP36L1 | ENST00000439696 | up | 0.915565 | 0.0323837 |
| RALGPS2 | ENST00000367635 | up | 0.9135336 | 0.006198 |
| TMEM160 | ENST00000253047 | up | 0.9112678 | 0.0203925 |
| BDKRB2 | NM_000623 | up | 0.9076421 | 0.0286744 |
| STEAP1 | ENST00000297205 | up | 0.9034875 | 0.0031328 |
| RP11-566K11.2 | ENST00000556922 | up | 0.9030946 | 0.0175216 |
| ELOVL3 | ENST00000370005 | up | 0.9021011 | 0.0177882 |
| HIST1H1E | ENST00000304218 | up | 0.9009266 | 0.0017771 |
| CLIC5 | ENST00000544153 | up | 0.9007307 | 0.0188686 |
| DBNL | ENST00000494774 | up | 0.9005583 | 0.0041359 |
| ARNTL2 | ENST00000546179 | up | 0.9001233 | 0.0443105 |
| FAM109B | ENST00000321753 | up | 0.8987191 | 0.0296622 |
| SLC16A6 | ENST00000327268 | up | 0.8984057 | 0.0238155 |
| FAM3C | NM_001040020 | up | 0.8964703 | 0.0244263 |
| ANP32E | ENST00000616917 | up | 0.8963866 | 0.0250959 |
| CLMP | ENST00000448775 | up | 0.8942268 | 0.0089619 |
| CCDC18 | ENST00000401026 | up | 0.8931777 | 0.030966 |
| BICD1 | NM_001354187 | up | 0.89126 | 0.0201575 |
| APOL3 | ENST00000424878 | up | 0.890882 | 0.0035066 |
| BRI3BP | ENST00000341446 | up | 0.8904281 | 0.020754 |
| CREB3L1 | ENST00000621158 | up | 0.8885104 | 0.0170629 |
| PLCB3 | ENST00000325234 | up | 0.8881807 | 0.008072 |
| PRMT2 | ENST00000458387 | up | 0.8856015 | 0.0011261 |
| MAGEC3 | ENST00000298296 | up | 0.8841897 | 0.0369441 |
| SEC24A | ENST00000398844 | up | 0.8832863 | 0.0448249 |
| CALU | uc022alg.1 | up | 0.882924 | 0.0056754 |
| SHANK2 | ENST00000338508 | up | 0.8828856 | 0.011451 |
| PLPP5 | ENST00000419686 | up | 0.8824716 | 0.0019538 |
| RPTOR | ENST00000570891 | up | 0.8818065 | 0.0449094 |
| ARSJ | ENST00000315366 | up | 0.8793836 | 0.0444994 |
| GDF6 | ENST00000287020 | up | 0.8787932 | 0.0283132 |
| RND3 | ENST00000263895 | up | 0.878267 | 0.0381546 |
| PPP1R1B | ENST00000394265 | up | 0.8747275 | 0.0248246 |
| TMEM243 | ENST00000257637 | up | 0.874191 | 0.0396697 |
| MELK | ENST00000541717 | up | 0.874037 | 0.0163525 |
| RFC2 | NM_001278791 | up | 0.8714152 | 0.0269053 |
| CCDC80 | ENST00000206423 | up | 0.8694298 | 0.0463507 |
| HIRIP3 | ENST00000279392 | up | 0.8686788 | 0.0094864 |
| AQP7 | ENST00000624075 | up | 0.8669584 | 0.0007962 |
| SIX1 | ENST00000247182 | up | 0.865821 | 0.033156 |
| RAB34 | ENST00000636154 | up | 0.8648246 | 0.0495018 |
| KIAA1217 | ENST00000458595 | up | 0.8646047 | 0.0204147 |
| OBSL1 | ENST00000373873 | up | 0.8636685 | 0.0059361 |
| UBAP2L | ENST00000271877 | up | 0.8607959 | 0.0340255 |
| SMARCA1 | ENST00000371121 | up | 0.8590958 | 0.019226 |
| JADE2 | ENST00000395003 | up | 0.858881 | 0.0312971 |
| CHPF2 | ENST00000035307 | up | 0.8578356 | 0.0293433 |
| PHTF2 | ENST00000307305 | up | 0.8561215 | 0.0356723 |
| MCM6 | ENST00000264156 | up | 0.8557527 | 0.0131505 |
| DIAPH1 | ENST00000389054 | up | 0.8541956 | 0.0495021 |
| OLFM1 | ENST00000277415 | up | 0.8534041 | 0.0257316 |
| OSTC | ENST00000613215 | up | 0.8531762 | 0.0001237 |
| RAET1G | ENST00000367360 | up | 0.8525123 | 0.0268752 |
| RHOG | ENST00000351018 | up | 0.8482335 | 0.0267079 |
| XG | ENST00000644266 | up | 0.8457114 | 0.0007556 |
| HERC4 | ENST00000395198 | up | 0.8446923 | 0.042845 |
| PRKCD | ENST00000394729 | up | 0.8441601 | 0.0166021 |
| PLD4 | ENST00000392593 | up | 0.8419603 | 0.0257067 |
| CTA-384D8.28 | ENST00000543927 | up | 0.8390274 | 0.0476301 |
| PHLDB1 | ENST00000600882 | up | 0.8381557 | 0.046916 |
| FAM63B | ENST00000450403 | up | 0.8367448 | 0.0198372 |
| EME1 | ENST00000338165 | up | 0.8319361 | 0.0049277 |
| SLC17A9 | ENST00000370351 | up | 0.8278968 | 0.0148963 |
| MASTL | ENST00000375946 | up | 0.8254856 | 0.0011653 |
| CATG00000038552.1 | HBMT00000700340 | up | 0.8254445 | 0.0148272 |
| FBLN1 | ENST00000442170 | up | 0.8244196 | 0.0404845 |
| SLC9A7 | ENST00000616978 | up | 0.8195746 | 0.0297059 |
| DICER1 | ENST00000343455 | up | 0.8179736 | 0.0449189 |
| CATG00000107322.1 | ENCT00000451616 | up | 0.8171987 | 0.0218355 |
| ABCC4 | ENST00000629385 | up | 0.8169956 | 0.005036 |
| CALU | ENST00000449187 | up | 0.8157457 | 0.0192592 |
| POLR3D | ENST00000397802 | up | 0.8150869 | 0.0249704 |
| YEATS2 | ENST00000305135 | up | 0.8146271 | 0.0253892 |
| C22orf29 | ENST00000407472 | up | 0.8142118 | 0.037669 |
| SORT1 | ENST00000256637 | up | 0.813064 | 0.0060375 |
| C1orf198 | ENST00000366663 | up | 0.81225 | 0.032881 |
| ZC3H7B | ENST00000352645 | up | 0.8076438 | 0.0377074 |
| RIOX1 | ENST00000304061 | up | 0.8060781 | 0.0269004 |
| KNSTRN | ENST00000448395 | up | 0.8058286 | 0.0308503 |
| PKM | ENST00000389093 | up | 0.8048542 | 0.0222018 |
| FKBP9 | ENST00000538336 | up | 0.8015047 | 0.0318457 |
| USP53 | ENST00000274030 | up | 0.7986265 | 0.0144479 |
| SCFD2 | ENST00000401642 | up | 0.7977456 | 0.045983 |
| RGMB | ENST00000308234 | up | 0.7969982 | 0.012968 |
| TNNI2 | ENST00000381905 | up | 0.7964571 | 0.0497843 |
| HIF1A | ENST00000539097 | up | 0.7945358 | 0.0176227 |
| KCTD3 | ENST00000259154 | up | 0.7942229 | 0.0442247 |
| CRYGS | ENST00000392499 | up | 0.7931347 | 0.0308989 |
| USP18 | ENST00000215794 | up | 0.7926935 | 0.0009205 |
| RRP7A | ENST00000323013 | up | 0.7916644 | 0.0463711 |
| DAP | ENST00000230895 | up | 0.7906641 | 0.0067788 |
| RCN3 | ENST00000270645 | up | 0.7877838 | 0.0111017 |
| FAM134C | ENST00000309428 | up | 0.7865045 | 0.0318459 |
| MAPRE2 | ENST00000300249 | up | 0.7863997 | 0.0378362 |
| SLC2A6 | ENST00000371897 | up | 0.7856476 | 0.0404732 |
| PACSIN3 | ENST00000298838 | up | 0.7854438 | 0.0215059 |
| PDLIM4 | ENST00000379018 | up | 0.7851368 | 0.0423215 |
| ATP2B4 | ENST00000367218 | up | 0.7850431 | 0.0077157 |
| GUCY1B3 | ENST00000264424 | up | 0.783633 | 0.0376456 |
| PDLIM5 | ENST00000318007 | up | 0.7803429 | 0.0155077 |
| ADD3 | NM_001320594 | up | 0.7770407 | 0.0475017 |
| SPTAN1 | ENST00000358161 | up | 0.7766241 | 0.0307466 |
| ARHGEF28 | uc003kcy.1 | up | 0.774364 | 0.0386 |
| NOTCH2 | ENST00000256646 | up | 0.7732553 | 0.0392015 |
| USP53 | NM_019050 | up | 0.7705653 | 0.0366253 |
| SQRDL | ENST00000260324 | up | 0.7693589 | 0.0108887 |
| RND3 | ENST00000375734 | up | 0.7691303 | 0.0289719 |
| TBX3 | ENST00000349155 | up | 0.7681996 | 0.0419206 |
| AGAP3 | ENST00000335367 | up | 0.7642672 | 0.0294726 |
| SFXN3 | ENST00000393459 | up | 0.7631559 | 0.0476975 |
| ALDH18A1 | ENST00000371221 | up | 0.7626231 | 0.031511 |
| MRPS6 | ENST00000399312 | up | 0.7619085 | 0.0368396 |
| AP4M1 | ENST00000359593 | up | 0.7613781 | 0.0074088 |
| DYNC1I2 | ENST00000397119 | up | 0.7576442 | 0.0184453 |
| RAB26 | ENST00000210187 | up | 0.7558908 | 0.0484324 |
| BOK | ENST00000318407 | up | 0.7552487 | 0.0134839 |
| TWIST2 | NM_001271893 | up | 0.755063 | 0.0112176 |
| GNB1 | ENST00000378609 | up | 0.7538191 | 0.0101495 |
| SMARCA1 | ENST00000371123 | up | 0.7534942 | 0.0200777 |
| SPATA6L | ENST00000475086 | up | 0.7533634 | 0.0454371 |
| STEAP1B | ENST00000406890 | up | 0.7507401 | 0.0087894 |
| C2orf81 | ENST00000290390 | up | 0.7506227 | 0.0004284 |
| CATG00000057022.1 | HBMT00000928308 | up | 0.7500673 | 0.0202521 |
| KRT34 | ENST00000394001 | up | 0.7478631 | 0.0260166 |
| C8orf86 | ENST00000437935 | up | 0.7477765 | 0.0135352 |
| RHOB | ENST00000272233 | up | 0.7465956 | 0.046032 |
| ENAH | ENST00000366844 | up | 0.7461518 | 0.0322008 |
| AIM1 | ENST00000369066 | up | 0.7421998 | 0.045559 |
| DYNC1I2 | ENST00000508530 | up | 0.7373711 | 0.0099782 |
| SCYL2 | ENST00000360820 | up | 0.7312293 | 0.044992 |
| TMED3 | ENST00000424155 | up | 0.7302699 | 0.0097509 |
| HTRA3 | ENST00000382512 | up | 0.7302047 | 0.0272914 |
| KCNMA1 | ENST00000638991 | up | 0.7301833 | 0.0472938 |
| AGPAT6 | ENST00000396987 | up | 0.7298762 | 0.0278674 |
| PDCD4 | NM_001199492 | up | 0.7277657 | 0.0091518 |
| NDRG1 | ENST00000323851 | up | 0.7241492 | 0.0291582 |
| SEC23A | ENST00000537403 | up | 0.7237236 | 0.0451441 |
| NT5E | ENST00000257770 | up | 0.7232612 | 0.015755 |
| SIPA1 | ENST00000534313 | up | 0.7230614 | 0.0248616 |
| PRMT2 | ENST00000440086 | up | 0.7229472 | 0.0020624 |
| BICC1 | ENST00000373886 | up | 0.7225585 | 0.0241506 |
| ERBB2 | ENST00000269571 | up | 0.71963 | 0.0163518 |
| CAMK2G | ENST00000322680 | up | 0.7192162 | 0.0193194 |
| DYNC1LI2 | NM_001323955 | up | 0.7169529 | 0.0220336 |
| ANGPTL5 | ENST00000334289 | up | 0.7160498 | 0.0054217 |
| HGSNAT | ENST00000379644 | up | 0.715884 | 0.0275154 |
| GAS6 | ENST00000327773 | up | 0.7150833 | 0.0019313 |
| KIAA1328 | ENST00000280020 | up | 0.7148317 | 0.0487939 |
| LOX | ENST00000231004 | up | 0.7129136 | 0.0399637 |
| ZC3H13 | NM_001330566 | up | 0.712528 | 0.0220172 |
| ACSF3 | ENST00000614302 | up | 0.711404 | 0.0019359 |
| SEPT7 | ENST00000435235 | up | 0.7111663 | 0.0308753 |
| PRRC1 | ENST00000296666 | up | 0.708632 | 0.0245958 |
| TFPI | ENST00000409676 | up | 0.7074395 | 0.0414182 |
| BAG3 | ENST00000369085 | up | 0.6986527 | 0.0399584 |
| STEAP3 | ENST00000393110 | up | 0.6984771 | 0.0096388 |
| ZNF503 | ENST00000372524 | up | 0.69536 | 0.0187917 |
| MAGEH1 | ENST00000342972 | up | 0.6933679 | 0.0295486 |
| SUPT5H | ENST00000359191 | up | 0.6921494 | 0.0214898 |
| RPS14 | ENST00000401695 | up | 0.6908723 | 0.0005432 |
| NIPAL3 | ENST00000339255 | up | 0.6881941 | 0.0268027 |
| PGM3 | ENST00000512866 | up | 0.6865476 | 0.0091497 |
| CITED4 | ENST00000372638 | up | 0.6862303 | 0.0267645 |
| KANK1 | ENST00000382293 | up | 0.6822861 | 0.0461268 |
| PAGR1 | ENST00000320330 | up | 0.6800444 | 0.0087912 |
| HIST1H2AK | ENST00000618958 | up | 0.678712 | 0.0030539 |
| CDHR3 | ENST00000478080 | up | 0.6786481 | 0.0289275 |
| SETD7 | ENST00000404104 | up | 0.6779327 | 0.0148857 |
| FN1 | ENST00000426059 | up | 0.6773494 | 0.0149154 |
| PTOV1 | ENST00000600603 | up | 0.6747588 | 0.0310054 |
| GPR161 | NM_001349635 | up | 0.6733177 | 0.007068 |
| SVIL | NM_001323600 | up | 0.6722845 | 0.0446747 |
| VCPKMT | ENST00000395860 | up | 0.6709162 | 0.041118 |
| ITSN1 | ENST00000381291 | up | 0.6696113 | 0.021805 |
| GNG12 | ENST00000370982 | up | 0.6653969 | 0.0182817 |
| KPNB1 | ENST00000290158 | up | 0.6643321 | 0.0246149 |
| CALU | ENST00000535011 | up | 0.6641245 | 0.04594 |
| SH3BGRL3 | ENST00000270792 | up | 0.663618 | 0.0018219 |
| WEE1 | ENST00000450114 | up | 0.6635663 | 0.026419 |
| PFKFB3 | ENST00000360521 | up | 0.6611456 | 0.0148624 |
| SUFU | ENST00000369902 | up | 0.653828 | 0.0442123 |
| CUL7 | ENST00000265348 | up | 0.6523311 | 0.0227703 |
| MVP | ENST00000395353 | up | 0.6508975 | 0.0180854 |
| ABCC4 | ENST00000646439 | up | 0.6493856 | 0.0499585 |
| RNF141 | ENST00000265981 | up | 0.6479093 | 0.003179 |
| ZEB2 | ENST00000636471 | up | 0.6472523 | 0.0140259 |
| SSH1 | ENST00000551165 | up | 0.6454541 | 0.0289975 |
| SLC16A3 | ENST00000617373 | up | 0.645307 | 0.0480439 |
| SMOC1 | ENST00000361956 | up | 0.6446121 | 0.0155217 |
| HNRNPUL1 | ENST00000593587 | up | 0.6444589 | 0.0221841 |
| FBLN7 | ENST00000331203 | up | 0.6441003 | 0.0319258 |
| ANO7 | ENST00000274979 | up | 0.6425118 | 0.0397797 |
| MAN1A1 | ENST00000368468 | up | 0.641273 | 0.0205786 |
| DTYMK | NM_001165031 | up | 0.6411867 | 0.0122485 |
| PRKAG2 | uc003wkl.2 | up | 0.6400321 | 0.0482717 |
| FKBP7 | ENST00000434643 | up | 0.6391198 | 0.0217603 |
| HFE | ENST00000397022 | up | 0.6385819 | 0.0425347 |
| IL1R1 | ENST00000409929 | up | 0.6364265 | 0.010446 |
| WDR47 | ENST00000369962 | up | 0.6296363 | 0.0344746 |
| TOMM40L | ENST00000545897 | up | 0.6277068 | 0.0262298 |
| RNASEH2A | ENST00000221486 | up | 0.6255491 | 0.0354073 |
| AMT | ENST00000458307 | up | 0.6253273 | 0.0455398 |
| ARHGEF19 | ENST00000270747 | up | 0.6215964 | 0.0071486 |
| PPT1 | ENST00000642050 | up | 0.6207061 | 0.0334973 |
| SNX21 | ENST00000491381 | up | 0.6204389 | 0.0201563 |
| UBE2T | ENST00000646651 | up | 0.6201393 | 0.0166626 |
| EIF4E2 | ENST00000409322 | up | 0.6140326 | 0.0036992 |
| ITPRIPL2 | ENST00000381440 | up | 0.6137881 | 0.0113989 |
| SORT1 | ENST00000538502 | up | 0.6117716 | 0.0468402 |
| NSD2 | ENST00000514045 | up | 0.6113197 | 0.0026166 |
| BARD1 | ENST00000260947 | up | 0.6065637 | 0.036777 |
| IL11RA | ENST00000318041 | up | 0.6065449 | 0.0245462 |
| CAST | ENST00000395812 | up | 0.6059991 | 0.007812 |
| EIF4E2 | ENST00000409394 | up | 0.6029568 | 0.0033174 |
| UAP1 | NM_001324117 | up | 0.602646 | 0.0484465 |
| ANP32E | ENST00000583931 | up | 0.6011119 | 0.0046298 |
| RAD21 | ENST00000297338 | up | 0.5992617 | 0.0188405 |
| GATSL3 | ENST00000407689 | up | 0.5945497 | 0.0286027 |
| DSEL | ENST00000310045 | up | 0.5908068 | 0.0386383 |
| CCND3 | ENST00000616010 | up | 0.5865724 | 0.0258945 |
| CFLAR | ENST00000479953 | up | 0.5862858 | 0.0292574 |
| MARVELD1 | ENST00000285605 | up | 0.5855957 | 0.0444226 |
| BTG1 | ENST00000256015 | down | -0.5849744 | 0.0014666 |
| SAT1 | ENST00000379251 | down | -0.5850314 | 0.0078196 |
| BSG | NM_001322243 | down | -0.5855301 | 0.0353814 |
| PARL | ENST00000639100 | down | -0.5860855 | 0.0061029 |
| TMEM14C | uc010joq.2 | down | -0.586378 | 0.0411971 |
| CATG00000058112.1 | HBMT00000946641 | down | -0.5866207 | 0.0278708 |
| FLNB | ENST00000295956 | down | -0.5867528 | 0.0327094 |
| PLBD2 | ENST00000280800 | down | -0.5870694 | 0.0239236 |
| HERPUD1 | ENST00000300302 | down | -0.5876241 | 0.0018339 |
| FAM133B | ENST00000438306 | down | -0.587978 | 0.0155186 |
| KCNE4 | ENST00000281830 | down | -0.5879836 | 0.0477299 |
| SLC9A5 | ENST00000299798 | down | -0.5885846 | 0.0049824 |
| NDUFB8 | ENST00000370322 | down | -0.5886437 | 0.0497235 |
| GGCT | ENST00000005374 | down | -0.5888501 | 0.0219755 |
| FAM188A | ENST00000418767 | down | -0.5890254 | 0.004821 |
| LZIC | NM_001316974 | down | -0.5891378 | 0.0044024 |
| SP140L | ENST00000243810 | down | -0.5892509 | 0.0320106 |
| ADAM15 | ENST00000356955 | down | -0.589965 | 0.008373 |
| NDUFB3 | ENST00000433898 | down | -0.5901048 | 0.045577 |
| HMBS | ENST00000392841 | down | -0.5912593 | 0.0231769 |
| ANKHD1 | ENST00000616482 | down | -0.5921088 | 0.0041925 |
| ZNF711 | ENST00000360700 | down | -0.5924007 | 0.0424838 |
| KHSRP | ENST00000398148 | down | -0.5926326 | 0.019428 |
| URI1 | ENST00000360605 | down | -0.5927898 | 0.021575 |
| C14orf119 | ENST00000319074 | down | -0.5929757 | 0.0119875 |
| MIPEP | ENST00000382172 | down | -0.5933919 | 0.034888 |
| RASGEF1A | ENST00000395809 | down | -0.5939211 | 0.0420539 |
| EAF1 | ENST00000396842 | down | -0.5940869 | 0.0231917 |
| EMC3 | NM_018447 | down | -0.5943378 | 0.0024328 |
| TTC26 | ENST00000478836 | down | -0.5945323 | 0.0479567 |
| MAGEF1 | ENST00000317897 | down | -0.594735 | 0.0186221 |
| WBP2 | ENST00000433525 | down | -0.5947416 | 0.0239022 |
| MCAT | ENST00000290429 | down | -0.5952858 | 0.0048906 |
| PHYH | ENST00000263038 | down | -0.5955088 | 0.0200896 |
| HSPBP1 | ENST00000255631 | down | -0.5956119 | 0.034933 |
| ZNF185 | ENST00000318504 | down | -0.5959551 | 0.0335594 |
| RHOF | ENST00000267205 | down | -0.5963126 | 0.0477391 |
| MORC2 | ENST00000397641 | down | -0.5965186 | 0.0248078 |
| PHEX | ENST00000379374 | down | -0.596724 | 0.0375397 |
| LSM14A | ENST00000544216 | down | -0.5970216 | 0.0352827 |
| RPL31 | ENST00000409733 | down | -0.5977434 | 0.0409965 |
| TRAPPC4 | ENST00000528230 | down | -0.5981193 | 0.0059465 |
| RWDD1 | ENST00000487832 | down | -0.5981709 | 0.0355299 |
| KTN1 | ENST00000395308 | down | -0.5983823 | 0.0432118 |
| KDM5C | ENST00000404049 | down | -0.5984161 | 0.0496205 |
| STK32C | NM_001318878 | down | -0.5988857 | 0.009436 |
| NCSTN | NM_001290186 | down | -0.5992897 | 0.0356686 |
| EPSTI1 | ENST00000313640 | down | -0.5993253 | 0.0077307 |
| TNRC6B | ENST00000402203 | down | -0.5994357 | 0.0002199 |
| PTPN6 | ENST00000456013 | down | -0.5996423 | 0.0373723 |
| FAM60A | ENST00000337682 | down | -0.6005223 | 0.0130791 |
| HOXD11 | ENST00000249504 | down | -0.6020299 | 0.0364636 |
| LAMP1 | ENST00000332556 | down | -0.6020623 | 0.0306854 |
| CATG00000107372.1 | ENCT00000451796 | down | -0.6022894 | 0.0247427 |
| ZFP90 | ENST00000611381 | down | -0.6024106 | 0.010472 |
| PRPSAP1 | ENST00000324684 | down | -0.6026251 | 0.03341 |
| TMA16 | ENST00000358572 | down | -0.6033069 | 0.0390955 |
| MRPS17 | ENST00000285298 | down | -0.6033874 | 0.0131786 |
| CORO7-PAM16 | ENST00000572467 | down | -0.6035414 | 0.0352846 |
| NDUFS2 | ENST00000392179 | down | -0.6036512 | 0.0130613 |
| TIAF1 | ENST00000359450 | down | -0.6042256 | 0.0030073 |
| CLNS1A | ENST00000525064 | down | -0.6044141 | 0.0036588 |
| ACSS3 | ENST00000261206 | down | -0.6044472 | 0.0336863 |
| LILRA2 | ENST00000391737 | down | -0.6047279 | 0.0104351 |
| IRS1 | ENST00000305123 | down | -0.6057319 | 0.0454956 |
| CAMK2D | ENST00000296402 | down | -0.6063501 | 0.0258883 |
| RPP21 | ENST00000442966 | down | -0.6065454 | 0.0325997 |
| MAPT | ENST00000415613 | down | -0.6066788 | 0.0439914 |
| VPS28 | ENST00000292510 | down | -0.6067732 | 0.0337984 |
| TMCO1 | ENST00000612311 | down | -0.6069563 | 0.0256058 |
| DESI1 | ENST00000263256 | down | -0.6074925 | 0.0234166 |
| ROR1 | ENST00000371079 | down | -0.6082567 | 0.0495082 |
| MRPS27 | ENST00000513900 | down | -0.6084743 | 0.0384859 |
| BLMH | ENST00000261714 | down | -0.6086655 | 0.0118599 |
| CTSK | ENST00000271651 | down | -0.608764 | 0.0301147 |
| RPL26 | ENST00000582556 | down | -0.6089259 | 0.0002808 |
| RPS9 | NM_001321704 | down | -0.6093016 | 0.0059854 |
| RNPS1 | ENST00000567147 | down | -0.6094343 | 0.0441731 |
| LSM1 | ENST00000520755 | down | -0.6098005 | 0.0447976 |
| LY96 | ENST00000284818 | down | -0.6101335 | 0.0051196 |
| FUT7 | ENST00000314412 | down | -0.6101415 | 0.0277299 |
| TMX2 | NM_001347898 | down | -0.6103742 | 0.0327383 |
| ZNF841 | NM_001352298 | down | -0.6104041 | 0.0356059 |
| MRPL23 | ENST00000397298 | down | -0.6104426 | 0.0123457 |
| ZNF506 | ENST00000443905 | down | -0.6111708 | 0.032561 |
| SEC14L1 | ENST00000392476 | down | -0.6111816 | 0.0363947 |
| DCAKD | ENST00000588499 | down | -0.61177 | 0.0393536 |
| KCNAB2 | ENST00000378092 | down | -0.6118927 | 0.000631 |
| CDV3 | ENST00000431519 | down | -0.6119636 | 0.0187099 |
| COPS3 | NM_001316357 | down | -0.6124068 | 0.0070929 |
| CATG00000013838.1 | MICT00000090075 | down | -0.6126857 | 0.0103772 |
| MORN3 | ENST00000355329 | down | -0.6134617 | 0.0408614 |
| BLOC1S1 | ENST00000547076 | down | -0.6135237 | 0.0285916 |
| CATG00000006002.1 | FTMT24200003673 | down | -0.6138666 | 0.0386816 |
| SET | ENST00000372688 | down | -0.6139057 | 0.0253761 |
| PDE4B | ENST00000329654 | down | -0.6142502 | 0.0263866 |
| COX11 | ENST00000571584 | down | -0.6143055 | 0.009479 |
| SMG6 | ENST00000263073 | down | -0.6150831 | 0.000701 |
| EARS2 | ENST00000563232 | down | -0.6152582 | 0.0454423 |
| TSPAN3 | ENST00000424443 | down | -0.6159974 | 0.0459308 |
| SLC39A11 | ENST00000255559 | down | -0.6161129 | 0.0144419 |
| PLOD3 | ENST00000223127 | down | -0.6165914 | 0.0092363 |
| SAE1 | ENST00000392776 | down | -0.6167215 | 0.0420566 |
| PDLIM2 | ENST00000308354 | down | -0.6167967 | 0.0487733 |
| CCP110 | ENST00000381396 | down | -0.6171503 | 0.0455896 |
| GEMIN8 | NM_001042479 | down | -0.6173634 | 0.0072199 |
| SLC12A8 | ENST00000393469 | down | -0.6174429 | 0.0238415 |
| DDX54 | ENST00000314045 | down | -0.6175236 | 0.046394 |
| ZBTB22 | ENST00000418724 | down | -0.6175685 | 0.0270925 |
| PRKCSH | ENST00000592741 | down | -0.617579 | 0.0397796 |
| TAF11 | ENST00000361288 | down | -0.6177194 | 0.0094045 |
| PRDX3 | NM_001302272 | down | -0.6183064 | 0.0163606 |
| PPP1R7 | ENST00000234038 | down | -0.6184933 | 0.0071587 |
| MARC2 | NM_001317338 | down | -0.6193908 | 0.0119665 |
| ARG2 | ENST00000261783 | down | -0.6194128 | 0.0058679 |
| HRAS | ENST00000451590 | down | -0.6196157 | 0.0278599 |
| HAUS4 | ENST00000206474 | down | -0.6196528 | 0.0053239 |
| ATP6V1G1 | ENST00000374050 | down | -0.6199246 | 0.0111651 |
| ZSCAN26 | ENST00000623276 | down | -0.6199429 | 0.0266435 |
| RTN4 | ENST00000317610 | down | -0.6200101 | 0.0167523 |
| TLE4 | ENST00000376552 | down | -0.620357 | 0.0171268 |
| CAMK2D | ENST00000514328 | down | -0.6203801 | 0.0141535 |
| LIG3 | ENST00000262327 | down | -0.6203829 | 0.0006037 |
| PHC2 | ENST00000257118 | down | -0.621242 | 0.0101184 |
| CNN2 | ENST00000263097 | down | -0.6215254 | 0.0385621 |
| CTCF | ENST00000646076 | down | -0.6219324 | 0.0152062 |
| RPL38 | ENST00000439590 | down | -0.6224982 | 0.01704 |
| ALG11 | ENST00000521508 | down | -0.622794 | 0.0381868 |
| RASGRP2 | ENST00000377494 | down | -0.6230031 | 0.0179619 |
| MRPS10 | ENST00000053468 | down | -0.6231761 | 0.0146733 |
| SH2D3A | ENST00000245908 | down | -0.6233531 | 0.0494391 |
| MRPS18C | ENST00000505719 | down | -0.6234348 | 0.0001166 |
| LMX1A | ENST00000342310 | down | -0.6237335 | 0.0199157 |
| RP11-216L13.17 | uc011meg.1 | down | -0.6239037 | 0.0383937 |
| FAM161B | ENST00000286544 | down | -0.6240619 | 0.0327014 |
| EMC7 | ENST00000256545 | down | -0.6244076 | 0.0235748 |
| DCAF11 | ENST00000446197 | down | -0.6246243 | 0.01201 |
| ARFGAP2 | ENST00000524782 | down | -0.6248377 | 0.0120688 |
| VPS11 | ENST00000621676 | down | -0.6250073 | 0.0146915 |
| PDHX | ENST00000430469 | down | -0.6252745 | 0.0142188 |
| C19orf12 | ENST00000592153 | down | -0.6254969 | 0.015422 |
| COMMD7 | ENST00000278980 | down | -0.6257835 | 0.004728 |
| TK2 | ENST00000569718 | down | -0.6261769 | 0.0090249 |
| NUDCD2 | ENST00000302764 | down | -0.6262316 | 0.0210482 |
| ELOVL6 | ENST00000394607 | down | -0.62655 | 0.0367103 |
| CTD-2135J3.2 | ENST00000519088 | down | -0.6270223 | 0.0105798 |
| AFAP1L1 | ENST00000515000 | down | -0.6274423 | 0.027944 |
| DNAJB11 | ENST00000265028 | down | -0.6275124 | 0.0046768 |
| SSRP1 | ENST00000278412 | down | -0.6276459 | 0.0275365 |
| APRT | ENST00000426324 | down | -0.6279729 | 0.0094986 |
| MRPS18A | ENST00000372133 | down | -0.6283796 | 0.0019815 |
| OSGIN1 | ENST00000343939 | down | -0.6287046 | 0.0339655 |
| PI4KB | ENST00000529142 | down | -0.6287608 | 0.0391372 |
| TBRG1 | ENST00000441174 | down | -0.628988 | 0.0272877 |
| RBM39 | NM_001323422 | down | -0.6290972 | 0.0137484 |
| RRP1B | ENST00000340648 | down | -0.6292206 | 0.0402562 |
| PTRH1 | ENST00000543175 | down | -0.6295548 | 0.032505 |
| DDX59 | NM_001349800 | down | -0.6296127 | 0.0491356 |
| PQBP1 | ENST00000396763 | down | -0.6302951 | 0.0478717 |
| MDM4 | ENST00000614459 | down | -0.6305293 | 0.0258398 |
| MTFR1 | ENST00000458689 | down | -0.6310017 | 0.0352148 |
| NUS1 | ENST00000368494 | down | -0.6316296 | 0.0387134 |
| C11orf73 | ENST00000618164 | down | -0.6316697 | 0.0130764 |
| B9D1 | ENST00000395616 | down | -0.6318439 | 0.0303599 |
| CDK11A | ENST00000356200 | down | -0.6318489 | 0.0210724 |
| PRPF38B | ENST00000370021 | down | -0.6323083 | 0.047655 |
| FECH | ENST00000262093 | down | -0.6327099 | 0.0151478 |
| PDSS1 | ENST00000376215 | down | -0.632864 | 0.0453834 |
| HIST1H2BB | ENST00000615966 | down | -0.6331236 | 0.0464256 |
| SLC25A39 | NM_001321241 | down | -0.6331966 | 0.0444118 |
| MBD2 | ENST00000256429 | down | -0.6333419 | 0.015647 |
| GSS | ENST00000646735 | down | -0.6333506 | 0.014136 |
| ZNF764 | ENST00000252797 | down | -0.6334703 | 0.0178584 |
| SEMA6B | ENST00000586582 | down | -0.633665 | 0.004253 |
| HPCAL4 | ENST00000617690 | down | -0.6337838 | 0.0384564 |
| RPS16 | ENST00000251453 | down | -0.6341756 | 0.0221225 |
| SEC61B | ENST00000223641 | down | -0.6343501 | 0.0281784 |
| TMED4 | ENST00000289577 | down | -0.6346313 | 0.0113452 |
| HSPE1 | ENST00000233893 | down | -0.6351317 | 0.012136 |
| SNRNP25 | ENST00000383018 | down | -0.6353409 | 0.0040466 |
| BEX3 | ENST00000372645 | down | -0.6355738 | 0.0016375 |
| MRPL54 | ENST00000330133 | down | -0.6358691 | 0.0028092 |
| DIABLO | ENST00000353548 | down | -0.6361956 | 0.0254317 |
| FAM133B | ENST00000427372 | down | -0.6362416 | 0.004027 |
| CDV3 | ENST00000511392 | down | -0.6364798 | 0.0147633 |
| MDFIC | ENST00000448022 | down | -0.6365736 | 0.0348484 |
| TCEAL9 | ENST00000372656 | down | -0.6368663 | 0.0034533 |
| TIMM23B | NM_001290118 | down | -0.6368962 | 0.013588 |
| NAE1 | ENST00000394074 | down | -0.6369123 | 0.0358958 |
| ZMYND11 | ENST00000397962 | down | -0.6377728 | 0.0225304 |
| SCML1 | ENST00000380045 | down | -0.6379647 | 0.0191071 |
| IL6ST | ENST00000381294 | down | -0.6382728 | 0.0142059 |
| CCNI | ENST00000237654 | down | -0.6386455 | 0.0252612 |
| IFT20 | ENST00000579419 | down | -0.6393422 | 0.009812 |
| CLDN9 | ENST00000445369 | down | -0.63947 | 0.0485854 |
| MBIP | ENST00000318473 | down | -0.6398406 | 0.0043423 |
| DPH7 | ENST00000277540 | down | -0.6398457 | 0.0107267 |
| AVPR2 | NM_001146151 | down | -0.639865 | 0.0352616 |
| CNN1 | ENST00000252456 | down | -0.6400213 | 0.0438063 |
| MESDC2 | ENST00000561312 | down | -0.6404961 | 0.0097197 |
| RPA2 | ENST00000373912 | down | -0.640669 | 0.0093347 |
| TNRC6B | ENST00000301923 | down | -0.6408377 | 0.0365613 |
| LAMP2 | ENST00000200639 | down | -0.6410871 | 0.000543 |
| ATP5G3 | ENST00000284727 | down | -0.6427755 | 0.0043773 |
| LLGL2 | ENST00000392550 | down | -0.6430763 | 0.015486 |
| MTX1 | ENST00000316721 | down | -0.6435382 | 0.0039626 |
| THUMPD3 | ENST00000345094 | down | -0.6435646 | 0.0364128 |
| MED17 | ENST00000251871 | down | -0.6448582 | 0.042377 |
| KLRD1 | ENST00000381908 | down | -0.6463087 | 0.0109704 |
| BCL11A | ENST00000642384 | down | -0.6463911 | 0.0273433 |
| GIGYF2 | ENST00000629305 | down | -0.6464282 | 0.0273137 |
| MRPL43 | ENST00000318325 | down | -0.6467105 | 0.0025534 |
| PREB | ENST00000406567 | down | -0.6476894 | 0.0007729 |
| SCN11A | ENST00000302328 | down | -0.6477183 | 0.0154014 |
| TMEM14C | ENST00000229563 | down | -0.6477657 | 0.0399997 |
| DERL1 | ENST00000519018 | down | -0.6479313 | 0.0139521 |
| SAP30BP | ENST00000583536 | down | -0.648409 | 0.034948 |
| USP19 | ENST00000398888 | down | -0.6490547 | 0.0185003 |
| RCE1 | ENST00000309657 | down | -0.6498483 | 0.015117 |
| XAF1 | ENST00000361842 | down | -0.6499132 | 0.0299784 |
| PPP1CC | ENST00000335007 | down | -0.6501168 | 0.0100247 |
| ZNF575 | ENST00000458714 | down | -0.6501286 | 0.0495658 |
| LAMP2 | ENST00000371335 | down | -0.6501868 | 0.0462988 |
| ARGLU1 | ENST00000400198 | down | -0.6502196 | 0.0396812 |
| COMMD5 | ENST00000305103 | down | -0.6506523 | 0.0142871 |
| PHB | ENST00000614445 | down | -0.6508253 | 0.0221287 |
| DAP3 | NM_001199849 | down | -0.6509783 | 0.001738 |
| PARP2 | ENST00000527915 | down | -0.6512525 | 0.0107792 |
| ZNF254 | ENST00000613065 | down | -0.6512861 | 0.0219763 |
| NPHP1 | ENST00000355301 | down | -0.6514366 | 0.0451311 |
| BTC | ENST00000395743 | down | -0.652038 | 0.0284227 |
| CABP2 | ENST00000636477 | down | -0.6525625 | 0.038451 |
| ATP5J2 | ENST00000359832 | down | -0.6526358 | 0.0311908 |
| PARD3 | ENST00000374788 | down | -0.6528344 | 0.0164176 |
| SURF6 | ENST00000372022 | down | -0.6530347 | 0.0156925 |
| DNAJC13 | NM_001329126 | down | -0.653046 | 0.0401759 |
| CDK2AP2 | ENST00000301488 | down | -0.6533877 | 0.0304454 |
| AUH | ENST00000303617 | down | -0.6533917 | 0.034954 |
| C20orf100 | ENST00000372999 | down | -0.6536482 | 0.0380272 |
| RAD23A | ENST00000586534 | down | -0.6537996 | 0.0253118 |
| VBP1 | ENST00000286428 | down | -0.6541856 | 0.0450275 |
| VBP1 | ENST00000286428 | down | -0.6541856 | 0.0450275 |
| C9orf69 | ENST00000561457 | down | -0.6546598 | 0.0424641 |
| HDHD2 | ENST00000300605 | down | -0.6547497 | 0.0458385 |
| ACBD6 | ENST00000367595 | down | -0.6547892 | 0.047725 |
| GMPR2 | ENST00000557854 | down | -0.6549677 | 0.0092116 |
| APLP2 | NM_001328686 | down | -0.6551719 | 0.0209594 |
| PDPK1 | ENST00000441549 | down | -0.6554556 | 0.0148747 |
| ZNF397 | ENST00000261333 | down | -0.6557432 | 0.0201019 |
| CDC42EP1 | ENST00000249014 | down | -0.6559569 | 0.0493947 |
| RPL7A | ENST00000323345 | down | -0.6559804 | 0.0499685 |
| PCBP2 | ENST00000359462 | down | -0.6561147 | 0.0350001 |
| SIPA1 | ENST00000394224 | down | -0.6562476 | 0.0038192 |
| ITPKC | ENST00000263370 | down | -0.6564103 | 0.0096859 |
| B4GALT3 | ENST00000319769 | down | -0.6564618 | 0.0272235 |
| KXD1 | ENST00000539106 | down | -0.6567825 | 0.0457135 |
| HSD17B1 | ENST00000225929 | down | -0.6570807 | 0.0404841 |
| ZCCHC8 | ENST00000633063 | down | -0.6576633 | 0.0069699 |
| ZNF107 | ENST00000613690 | down | -0.6576988 | 0.0052311 |
| C1orf131 | ENST00000366649 | down | -0.6579451 | 0.033993 |
| LRP1 | ENST00000243077 | down | -0.6579659 | 0.0220832 |
| MYCBP | ENST00000397572 | down | -0.6585073 | 0.0240016 |
| DPH3 | ENST00000383775 | down | -0.6585073 | 0.023082 |
| IFRD1 | ENST00000005558 | down | -0.6588805 | 0.0136249 |
| RBAK-RBAKDN | ENST00000396904 | down | -0.6600665 | 0.027757 |
| TMED4 | ENST00000457408 | down | -0.6605595 | 0.035636 |
| ZNHIT3 | NM_001281433 | down | -0.6606148 | 0.0094405 |
| NAE1 | ENST00000359087 | down | -0.6606157 | 0.0170879 |
| PRPF38B | ENST00000370025 | down | -0.6608212 | 0.0063484 |
| C14orf179 | ENST00000314067 | down | -0.6611638 | 0.0245268 |
| C17orf80 | ENST00000255557 | down | -0.6614244 | 0.0421925 |
| GET4 | ENST00000265857 | down | -0.6614598 | 0.0153567 |
| PPP4R3B | ENST00000616288 | down | -0.6617167 | 0.0422028 |
| DNAJC2 | ENST00000249270 | down | -0.6621581 | 0.0278873 |
| GPER1 | ENST00000397088 | down | -0.6626759 | 0.0110574 |
| CYHR1 | ENST00000424149 | down | -0.6629808 | 0.0141999 |
| TMEM143 | ENST00000293261 | down | -0.663327 | 0.0249842 |
| SUMF1 | ENST00000405420 | down | -0.6634506 | 0.0217127 |
| NSMCE2 | NM_001349486 | down | -0.6643673 | 0.0213709 |
| RPP38 | ENST00000378197 | down | -0.6645423 | 0.0147118 |
| SLC39A3 | ENST00000269740 | down | -0.6646018 | 0.0274517 |
| ZMYND11 | ENST00000381591 | down | -0.664911 | 0.0463839 |
| APLP2 | ENST00000528499 | down | -0.6652979 | 0.0372516 |
| PHRF1 | ENST00000413872 | down | -0.6654332 | 0.000534 |
| PARD3 | ENST00000340077 | down | -0.6659583 | 0.0255983 |
| CD320 | ENST00000537716 | down | -0.6664141 | 0.0011044 |
| HIGD2A | ENST00000274787 | down | -0.6666493 | 0.0427015 |
| REPS1 | ENST00000367663 | down | -0.6666892 | 0.0209604 |
| COPG2 | NM_012133 | down | -0.6671297 | 0.0174372 |
| BECN1 | ENST00000438274 | down | -0.6671694 | 0.0180324 |
| ZNF2 | ENST00000617923 | down | -0.6674941 | 0.0312833 |
| RUNX1 | ENST00000437180 | down | -0.667747 | 0.0099138 |
| USP3 | ENST00000540797 | down | -0.6681389 | 0.0242403 |
| FZD9 | ENST00000344575 | down | -0.668189 | 0.046699 |
| SMIM7 | ENST00000627144 | down | -0.6685511 | 0.0204667 |
| ADAD2 | ENST00000315906 | down | -0.6685623 | 0.0370132 |
| ADAR | ENST00000368474 | down | -0.6689988 | 0.0439414 |
| SYVN1 | ENST00000294256 | down | -0.6690115 | 0.0203673 |
| HAX1 | ENST00000457918 | down | -0.6690352 | 0.0314627 |
| POLR1E | ENST00000377798 | down | -0.6692158 | 0.0203164 |
| PPARA | ENST00000262735 | down | -0.6693876 | 0.0436843 |
| MSL3 | ENST00000337339 | down | -0.6699505 | 0.0120476 |
| UCHL5 | ENST00000367449 | down | -0.6711237 | 0.0469618 |
| TTC14 | ENST00000382584 | down | -0.6712278 | 0.0142629 |
| TPGS2 | ENST00000591906 | down | -0.6713167 | 0.0285745 |
| C19orf54 | ENST00000378313 | down | -0.671426 | 0.0393474 |
| UBE2K | ENST00000261427 | down | -0.6714838 | 0.0076156 |
| C12orf4 | NM_001346156 | down | -0.671968 | 0.0245154 |
| SLC36A1 | ENST00000243389 | down | -0.6720606 | 0.0376621 |
| POLR1D | ENST00000302979 | down | -0.6724371 | 0.0087714 |
| TRAPPC4 | ENST00000533632 | down | -0.6724791 | 0.0131059 |
| LCE1C | ENST00000607093 | down | -0.6724925 | 0.013981 |
| HGF | ENST00000457544 | down | -0.6725605 | 0.0363557 |
| CPT1C | ENST00000598293 | down | -0.6727824 | 0.0030839 |
| WRN | ENST00000298139 | down | -0.6730095 | 0.039819 |
| NUDT6 | ENST00000339154 | down | -0.6730279 | 0.0498699 |
| RUNX3 | ENST00000399916 | down | -0.6735306 | 0.0160454 |
| CAMK2D | ENST00000394524 | down | -0.673567 | 0.033026 |
| ZKSCAN8 | ENST00000457389 | down | -0.6736342 | 0.0345695 |
| UBXN8 | ENST00000615729 | down | -0.6738109 | 0.0259554 |
| PROCR | ENST00000216968 | down | -0.6747162 | 0.0150774 |
| STOML2 | ENST00000356493 | down | -0.6748758 | 0.0334991 |
| CATG00000082420.1 | MICT00000296176 | down | -0.6749319 | 0.0294994 |
| ZNF195 | ENST00000528410 | down | -0.67527 | 0.0098429 |
| ARMC7 | ENST00000584947 | down | -0.6760722 | 0.014753 |
| C1orf123 | NM_001304760 | down | -0.676954 | 0.031167 |
| SRSF4 | ENST00000373795 | down | -0.6769972 | 0.0273159 |
| SNAP91 | ENST00000521485 | down | -0.6770698 | 0.0231894 |
| RWDD4 | ENST00000326397 | down | -0.6771011 | 0.0252199 |
| HYAL1 | ENST00000395144 | down | -0.6771381 | 0.0051685 |
| SLC5A6 | ENST00000310574 | down | -0.6773102 | 0.0213928 |
| MDM4 | ENST00000391947 | down | -0.6776368 | 0.0277472 |
| ZNF205 | ENST00000620094 | down | -0.6776974 | 0.0295726 |
| HADHA | ENST00000380649 | down | -0.6780737 | 0.0236425 |
| NFU1 | ENST00000394305 | down | -0.6781597 | 0.0108868 |
| SHARPIN | ENST00000398712 | down | -0.678726 | 0.0301268 |
| FAAP20 | ENST00000378546 | down | -0.6790628 | 0.0382356 |
| GPN1 | ENST00000407583 | down | -0.6794954 | 0.003064 |
| SETD2 | ENST00000409792 | down | -0.6797504 | 0.026982 |
| C21orf2 | ENST00000339818 | down | -0.6798889 | 0.0296218 |
| TNRC6A | ENST00000315183 | down | -0.6799728 | 0.0422733 |
| PRMT5 | ENST00000216350 | down | -0.6800556 | 0.0311873 |
| UNC45A | NM_001323619 | down | -0.6804254 | 0.0299612 |
| NDUFS6 | ENST00000274137 | down | -0.6805029 | 0.0125524 |
| NKIRAS1 | ENST00000425478 | down | -0.6805861 | 0.0191774 |
| NDUFA12 | ENST00000547986 | down | -0.6806335 | 0.0005999 |
| GTDC1 | ENST00000344850 | down | -0.6806397 | 0.0298812 |
| C1orf122 | ENST00000373042 | down | -0.6808901 | 0.0070355 |
| CGGBP1 | ENST00000398392 | down | -0.6814481 | 0.0285711 |
| ZNF331 | ENST00000511154 | down | -0.6815278 | 0.0041507 |
| CHMP3 | ENST00000409225 | down | -0.6818179 | 0.0205911 |
| ATG14 | ENST00000247178 | down | -0.6820146 | 0.0135872 |
| SET | ENST00000372692 | down | -0.6820849 | 0.0444228 |
| ASCC2 | ENST00000307790 | down | -0.6821801 | 0.0199809 |
| FOXP1 | ENST00000614176 | down | -0.6823425 | 0.0384805 |
| SH3RF2 | ENST00000511217 | down | -0.6824378 | 0.0428187 |
| TMEM126B | ENST00000358867 | down | -0.6832797 | 0.0049006 |
| SIL1 | ENST00000265195 | down | -0.6834814 | 0.0464426 |
| TTLL1 | ENST00000266254 | down | -0.6836153 | 0.0096877 |
| MRPL15 | ENST00000260102 | down | -0.6836341 | 0.0163364 |
| RP4-614O4.5 | ENST00000424358 | down | -0.6836429 | 0.0006902 |
| SUPT20H | ENST00000350612 | down | -0.6839147 | 0.0104264 |
| DDOST | ENST00000415136 | down | -0.6844517 | 0.0347926 |
| TRIM17 | ENST00000295033 | down | -0.6846077 | 0.0045551 |
| CCDC130 | ENST00000585844 | down | -0.6846494 | 0.0251788 |
| LDLRAD2 | ENST00000344642 | down | -0.6852268 | 0.0193506 |
| DCP1B | ENST00000535873 | down | -0.6853042 | 0.0429144 |
| PANX2 | ENST00000395842 | down | -0.6858267 | 0.0439838 |
| ATE1 | ENST00000224652 | down | -0.6858456 | 0.0016497 |
| HSF2 | ENST00000452194 | down | -0.6859005 | 0.0422968 |
| SDF2L1 | ENST00000248958 | down | -0.686007 | 0.0317769 |
| C12orf43 | ENST00000537817 | down | -0.6861825 | 0.0317072 |
| TBC1D22B | ENST00000373491 | down | -0.6868636 | 0.0211309 |
| DOCK6 | ENST00000294618 | down | -0.6870172 | 0.0430579 |
| YAE1D1 | ENST00000223273 | down | -0.6881036 | 0.0002496 |
| RCN2 | ENST00000394885 | down | -0.6884897 | 0.036858 |
| UPP1 | ENST00000331803 | down | -0.6885697 | 0.0494589 |
| NOX5 | ENST00000530406 | down | -0.6889879 | 0.0279825 |
| TSNARE1 | ENST00000519651 | down | -0.6892062 | 0.020015 |
| RDH11 | ENST00000428130 | down | -0.6893906 | 0.0076246 |
| DHPS | ENST00000351660 | down | -0.6898076 | 0.0048743 |
| BRPF3 | ENST00000357641 | down | -0.6901949 | 0.0038827 |
| DHRS11 | ENST00000618403 | down | -0.6903621 | 0.0354302 |
| COPZ1 | ENST00000262061 | down | -0.6906129 | 0.017438 |
| ZNF358 | ENST00000597229 | down | -0.6907933 | 0.0458001 |
| PLEKHB2 | ENST00000409612 | down | -0.6908223 | 0.0108001 |
| ASIP | ENST00000568305 | down | -0.6923479 | 0.0427384 |
| ZNF93 | ENST00000343769 | down | -0.692348 | 0.0353606 |
| GATA2 | ENST00000487848 | down | -0.6924472 | 0.0055687 |
| PSMB9 | uc011dqj.2 | down | -0.6925449 | 0.0035304 |
| ETFDH | ENST00000511912 | down | -0.6926441 | 0.0096157 |
| ATG3 | ENST00000283290 | down | -0.692763 | 0.0182111 |
| ZNF460 | ENST00000360338 | down | -0.692867 | 0.0404353 |
| NUP37 | ENST00000552283 | down | -0.6931432 | 0.0018319 |
| KIAA1683 | ENST00000600359 | down | -0.6931619 | 0.0155859 |
| SNRPD1 | NM_001291916 | down | -0.693365 | 0.0353105 |
| CCNG1 | ENST00000340828 | down | -0.6937521 | 0.0072781 |
| PPP3CC | ENST00000397775 | down | -0.6938756 | 0.0355759 |
| PCTP | ENST00000573500 | down | -0.6941125 | 0.0378958 |
| AC040977.1 | ENST00000593646 | down | -0.6943795 | 0.0066897 |
| TNPO3 | ENST00000265388 | down | -0.6948546 | 0.0290633 |
| MFAP2 | NM_001135247 | down | -0.6954784 | 0.0240424 |
| C3orf33 | ENST00000340171 | down | -0.695726 | 0.0287409 |
| VPS13A | ENST00000357409 | down | -0.6960193 | 0.0048299 |
| TMUB2 | ENST00000538716 | down | -0.6960273 | 0.0411727 |
| YWHAZ | ENST00000395951 | down | -0.6965142 | 0.0206289 |
| CDK5RAP1 | ENST00000339269 | down | -0.6976799 | 0.0140239 |
| RPRD2 | ENST00000401000 | down | -0.6980981 | 0.0476396 |
| IKZF3 | ENST00000467757 | down | -0.6986057 | 0.04813 |
| DDB2 | ENST00000378600 | down | -0.6988083 | 0.0443635 |
| CPT1A | ENST00000376618 | down | -0.6988129 | 0.0011774 |
| CTCF | ENST00000264010 | down | -0.6996245 | 0.0466744 |
| AX747988 | uc001ayv.2 | down | -0.6998089 | 0.0492182 |
| FAM45A | ENST00000361432 | down | -0.7004762 | 0.0133548 |
| ALPK2 | ENST00000361673 | down | -0.7009193 | 0.0469963 |
| C18orf21 | ENST00000333234 | down | -0.7013343 | 0.0485873 |
| PROSER2 | ENST00000277570 | down | -0.7015032 | 0.0465966 |
| TKT | ENST00000423516 | down | -0.7017098 | 0.0128111 |
| HMG20A | ENST00000336216 | down | -0.7022567 | 0.002281 |
| DTNA | ENST00000595022 | down | -0.7030025 | 0.0202692 |
| VCAN | ENST00000502527 | down | -0.7034777 | 0.0383338 |
| RHOT1 | ENST00000394692 | down | -0.7034842 | 0.0278506 |
| SLC12A8 | uc003ehw.4 | down | -0.7036878 | 0.0342046 |
| CATG00000060081.1 | HBMT00000973041 | down | -0.703891 | 0.0290183 |
| SHC1 | ENST00000368453 | down | -0.7039507 | 0.0087721 |
| APOM | ENST00000375920 | down | -0.7039619 | 0.0081845 |
| EXOSC5 | ENST00000221233 | down | -0.7042347 | 0.0401859 |
| IBTK | ENST00000510291 | down | -0.7042451 | 0.0286513 |
| ANKHD1-EIF4EBP3 | ENST00000532219 | down | -0.7043938 | 0.0223542 |
| CD209 | ENST00000204801 | down | -0.7049025 | 0.0424792 |
| MYO10 | ENST00000274203 | down | -0.7052653 | 0.0275093 |
| CXorf56 | ENST00000536133 | down | -0.706347 | 0.0352092 |
| HPCAL1 | ENST00000307845 | down | -0.7066908 | 0.0370666 |
| CCBL2 | ENST00000370491 | down | -0.7074422 | 0.0296383 |
| ACACA | ENST00000616317 | down | -0.7074544 | 0.0261437 |
| ARHGAP36 | ENST00000370921 | down | -0.7079884 | 0.0239988 |
| CACNB4 | ENST00000201943 | down | -0.7080186 | 0.0058649 |
| SCAMP4 | ENST00000316097 | down | -0.7080319 | 0.0112023 |
| PCGF2 | ENST00000620225 | down | -0.7081084 | 0.0146639 |
| ERVK3-1 | ENST00000610038 | down | -0.7082055 | 0.026132 |
| SAP30 | ENST00000296504 | down | -0.7084842 | 0.0373964 |
| CEP250 | NM_001318219 | down | -0.7089645 | 0.0131363 |
| PAQR6 | ENST00000613336 | down | -0.7091388 | 0.0376589 |
| RBM4 | ENST00000396053 | down | -0.7092287 | 0.0347397 |
| NOP16 | ENST00000618911 | down | -0.7092615 | 0.0217019 |
| TROVE2 | ENST00000367444 | down | -0.7097946 | 0.0071739 |
| BRE | ENST00000379624 | down | -0.7098451 | 0.003886 |
| MARC2 | ENST00000366913 | down | -0.7099069 | 0.0100281 |
| DHRS4 | ENST00000397075 | down | -0.7100527 | 0.0137901 |
| TSEN34 | ENST00000429671 | down | -0.7101982 | 0.0406174 |
| FAM168B | ENST00000409185 | down | -0.7103704 | 0.0088263 |
| MARK3 | ENST00000429436 | down | -0.7107357 | 0.0135726 |
| VRK3 | ENST00000594092 | down | -0.7108472 | 0.0366405 |
| POGK | ENST00000367876 | down | -0.7113829 | 0.021114 |
| TXNL4B | ENST00000268483 | down | -0.711587 | 0.0141426 |
| FKBP3 | ENST00000396062 | down | -0.7118115 | 0.0211991 |
| CHMP3 | ENST00000409727 | down | -0.7121828 | 0.0029261 |
| OS9 | ENST00000551035 | down | -0.7122283 | 0.0222619 |
| UNC93A | ENST00000230256 | down | -0.7124465 | 0.031421 |
| KLHL7 | ENST00000322275 | down | -0.7131379 | 0.0131154 |
| MGARP | ENST00000398955 | down | -0.7131779 | 0.038545 |
| PRPF40B | ENST00000548825 | down | -0.713666 | 0.0232977 |
| HLA-DQB2 | ENST00000411527 | down | -0.7139224 | 0.0183303 |
| COMMD9 | ENST00000532705 | down | -0.7141174 | 0.0048889 |
| MFF | ENST00000349901 | down | -0.7145216 | 0.0254031 |
| ZNF780A | ENST00000414720 | down | -0.7146446 | 0.0417434 |
| CARS | ENST00000278224 | down | -0.7147995 | 0.0335075 |
| LIPC | ENST00000414170 | down | -0.7151296 | 0.0234119 |
| PTPN13 | ENST00000316707 | down | -0.7151986 | 0.0254822 |
| RPL36 | ENST00000579649 | down | -0.7153165 | 0.008393 |
| NGDN | ENST00000397154 | down | -0.7156483 | 0.0022274 |
| APPL1 | ENST00000288266 | down | -0.7160396 | 0.0046372 |
| BIN1 | ENST00000352848 | down | -0.716204 | 0.0110131 |
| FAM185A | ENST00000413034 | down | -0.7164312 | 0.0140819 |
| CPT1B | ENST00000405237 | down | -0.7164531 | 0.0209987 |
| EXOC1 | ENST00000346134 | down | -0.7166858 | 0.0268032 |
| PTPN2 | ENST00000353319 | down | -0.7171371 | 0.0122004 |
| KCMF1 | ENST00000409785 | down | -0.7172024 | 0.0004176 |
| ZNF737 | ENST00000427401 | down | -0.7175698 | 0.0387703 |
| TRAPPC6B | ENST00000347691 | down | -0.7178449 | 0.0397218 |
| PPID | ENST00000307720 | down | -0.7184066 | 0.0135784 |
| CD46 | ENST00000322875 | down | -0.7185501 | 0.0269183 |
| PRDX2 | ENST00000334482 | down | -0.7197784 | 0.0043679 |
| ZFYVE1 | ENST00000318876 | down | -0.7203423 | 0.0352747 |
| INTS9 | ENST00000521022 | down | -0.7210978 | 0.001512 |
| DAP3 | ENST00000343043 | down | -0.7212232 | 0.0229211 |
| ZNF682 | ENST00000358523 | down | -0.7212779 | 0.0267046 |
| ZNF274 | ENST00000345813 | down | -0.7213651 | 0.0466662 |
| TTC12 | NM_001318533 | down | -0.7214012 | 0.0219018 |
| DSCR3 | ENST00000398998 | down | -0.721486 | 0.0134148 |
| ZNF480 | ENST00000334564 | down | -0.7217233 | 0.0059861 |
| TMEM183A | ENST00000367242 | down | -0.7217911 | 0.0305905 |
| TRAPPC11 | ENST00000334690 | down | -0.7218086 | 0.0061468 |
| GRTP1 | ENST00000620217 | down | -0.7219241 | 0.0217245 |
| SMPDL3A | ENST00000539041 | down | -0.7223591 | 0.0132523 |
| THAP3 | ENST00000054650 | down | -0.7224832 | 0.0281235 |
| SP100 | ENST00000409897 | down | -0.7231203 | 0.0192224 |
| SPATC1L | ENST00000330205 | down | -0.7231693 | 0.0438571 |
| HGFAC | ENST00000382774 | down | -0.7232705 | 0.0262465 |
| TMEM106C | ENST00000256686 | down | -0.723736 | 0.0484655 |
| TMEM205 | ENST00000589555 | down | -0.724439 | 0.0481052 |
| SOAT1 | ENST00000367619 | down | -0.7246398 | 0.0008597 |
| ZNF83 | ENST00000594682 | down | -0.7250493 | 0.0042774 |
| PSENEN | ENST00000587708 | down | -0.7251978 | 0.0277153 |
| PSMB8 | uc011dqh.2 | down | -0.7252506 | 0.001122 |
| CLIP1 | ENST00000537178 | down | -0.7256485 | 0.0058572 |
| ACVR1B | ENST00000257963 | down | -0.7256595 | 0.0183063 |
| C20orf196 | ENST00000303142 | down | -0.7258719 | 0.0255842 |
| CD6 | ENST00000352009 | down | -0.7262569 | 0.0380781 |
| ZFY | ENST00000625061 | down | -0.7264039 | 0.0450089 |
| ZZEF1 | ENST00000381638 | down | -0.7264144 | 0.0428857 |
| TGFBR1 | ENST00000374994 | down | -0.7266019 | 0.0409492 |
| MAPKAPK5 | ENST00000550735 | down | -0.7268219 | 0.0251528 |
| C18orf21 | ENST00000618334 | down | -0.7269877 | 0.031384 |
| NBPF12 | ENST00000446760 | down | -0.7272684 | 0.0446178 |
| ALKBH6 | ENST00000378875 | down | -0.7277207 | 0.026236 |
| SART3 | ENST00000228284 | down | -0.728064 | 0.0205232 |
| GPAT3 | ENST00000264409 | down | -0.7282868 | 0.0492874 |
| ZNF573 | ENST00000590414 | down | -0.7283661 | 0.0149632 |
| APPBP2 | ENST00000083182 | down | -0.7286497 | 0.0089875 |
| FASTKD1 | ENST00000453153 | down | -0.7286795 | 0.0249041 |
| EFR3A | ENST00000519656 | down | -0.7288576 | 0.0338304 |
| HAGH | ENST00000455446 | down | -0.7289329 | 0.0070472 |
| E2F4 | ENST00000379378 | down | -0.729063 | 0.0026557 |
| CD40 | NM_001322421 | down | -0.7290686 | 0.0298479 |
| CATG00000072683.1 | MICT00000017894 | down | -0.7295757 | 0.0411345 |
| COA7 | ENST00000371538 | down | -0.7303406 | 0.0423666 |
| CNIH4 | ENST00000366857 | down | -0.7304452 | 0.0411998 |
| LRRC6 | ENST00000618342 | down | -0.7305946 | 0.0464037 |
| ZNF431 | ENST00000600692 | down | -0.7306459 | 0.0043623 |
| METTL2B | ENST00000262432 | down | -0.7311653 | 0.0235223 |
| TMEM114 | ENST00000620492 | down | -0.7312765 | 0.0358451 |
| CXorf40A | ENST00000393985 | down | -0.731323 | 0.0130088 |
| HEMK1 | ENST00000232854 | down | -0.7313496 | 0.0341397 |
| GAPVD1 | ENST00000394083 | down | -0.7316001 | 0.002712 |
| PDHA1 | ENST00000422285 | down | -0.7316799 | 0.0045038 |
| RASGEF1B | ENST00000436139 | down | -0.7320517 | 0.0158844 |
| PAPOLG | ENST00000412217 | down | -0.7322748 | 0.0476575 |
| C8orf44 | ENST00000519561 | down | -0.732778 | 0.0111305 |
| ZNF813 | ENST00000396403 | down | -0.7329409 | 0.0496469 |
| GAPVD1 | ENST00000495955 | down | -0.7329857 | 0.0488852 |
| NAXE | uc001fpk.3 | down | -0.7330882 | 0.009409 |
| TRIM52 | ENST00000611618 | down | -0.7334716 | 0.0272938 |
| LGALS12 | ENST00000255684 | down | -0.7336417 | 0.0361642 |
| RAB1A | uc010yqe.2 | down | -0.7339726 | 2.094E-05 |
| ARMC10 | ENST00000425331 | down | -0.7341499 | 0.0267457 |
| C1orf27 | ENST00000367470 | down | -0.7341982 | 0.028675 |
| FDPS | ENST00000368356 | down | -0.7344908 | 0.0035763 |
| TSN | ENST00000536142 | down | -0.7348546 | 0.0488394 |
| PPP4R2 | ENST00000495566 | down | -0.734927 | 0.0458153 |
| ALAS1 | ENST00000469224 | down | -0.7349781 | 0.0166167 |
| CHD1 | ENST00000284049 | down | -0.7350169 | 0.0311516 |
| CREBL2 | ENST00000228865 | down | -0.7350721 | 0.0192984 |
| AAED1 | ENST00000375234 | down | -0.7352263 | 0.0464707 |
| USP46 | ENST00000451218 | down | -0.7362236 | 0.027183 |
| ALG1L | ENST00000611639 | down | -0.7365789 | 0.0107343 |
| PXK | NM_001349528 | down | -0.7366429 | 0.0379395 |
| OS9 | ENST00000435406 | down | -0.7375182 | 0.0051888 |
| C1orf56 | ENST00000368926 | down | -0.7376494 | 0.0329733 |
| PSMD10 | ENST00000361815 | down | -0.7380049 | 0.0015455 |
| BMP2K | ENST00000335016 | down | -0.7382579 | 0.0235834 |
| RPS14 | ENST00000407193 | down | -0.7384718 | 0.0038594 |
| EXOSC1 | ENST00000370902 | down | -0.7387026 | 0.006681 |
| MYEF2 | ENST00000267836 | down | -0.7389333 | 0.0032997 |
| HSF4 | NM_001040667 | down | -0.7389683 | 0.0118669 |
| NCOA7 | ENST00000229634 | down | -0.7393144 | 0.0108083 |
| TMEM136 | ENST00000529187 | down | -0.739676 | 0.0291564 |
| RAB7A | ENST00000265062 | down | -0.7399364 | 0.0112499 |
| CCDC191 | NM_001353766 | down | -0.7400942 | 0.0242882 |
| RAP1GDS1 | ENST00000453712 | down | -0.740229 | 0.0467923 |
| ADAMTS15 | ENST00000299164 | down | -0.7409316 | 0.0262698 |
| THRAP3 | ENST00000354618 | down | -0.741068 | 0.0085017 |
| HNRNPK | uc011lsw.2 | down | -0.7410956 | 0.0044112 |
| BRMS1 | ENST00000425825 | down | -0.7414346 | 0.0228561 |
| APOL1 | ENST00000426053 | down | -0.7414488 | 0.0061617 |
| DCTN4 | ENST00000446090 | down | -0.7414494 | 0.0449657 |
| ZNF586 | ENST00000391702 | down | -0.7415362 | 0.04542 |
| ETFB | ENST00000309244 | down | -0.7417388 | 0.0239482 |
| ATP5SL | ENST00000438807 | down | -0.7417738 | 0.0139186 |
| FAAP20 | ENST00000400918 | down | -0.7417986 | 0.0199437 |
| RAP1GAP | ENST00000290101 | down | -0.7418116 | 0.0339343 |
| NUDT2 | ENST00000346365 | down | -0.7419235 | 0.0391427 |
| RNF19B | ENST00000235150 | down | -0.7419289 | 0.0172568 |
| PTS | ENST00000280362 | down | -0.7425533 | 0.0382609 |
| PIK3CB | ENST00000544716 | down | -0.742659 | 0.0301816 |
| MFF | ENST00000409616 | down | -0.7432205 | 0.0067392 |
| SGF29 | ENST00000317058 | down | -0.7434791 | 0.0051741 |
| ECSIT | ENST00000270517 | down | -0.7435325 | 0.0367671 |
| SPRYD7 | ENST00000361840 | down | -0.7435719 | 0.0024899 |
| NIFK | ENST00000285814 | down | -0.7442246 | 0.0220373 |
| RABGAP1L | NM_001330989 | down | -0.7450972 | 0.0060068 |
| C5orf22 | ENST00000325366 | down | -0.7456823 | 0.0470922 |
| FASTKD1 | ENST00000453929 | down | -0.745946 | 0.025325 |
| SNRPA | ENST00000243563 | down | -0.7462789 | 0.0040278 |
| MTHFD1L | NM_001350488 | down | -0.7470075 | 0.0100332 |
| CCDC51 | ENST00000412398 | down | -0.7471196 | 0.0396129 |
| HOXC4 | ENST00000430889 | down | -0.7474495 | 0.0462031 |
| NUDT15 | ENST00000258662 | down | -0.747687 | 0.0343513 |
| HIBADH | uc003szi.3 | down | -0.7481226 | 0.0139697 |
| C16orf87 | ENST00000285697 | down | -0.74817 | 0.0269653 |
| ING5 | ENST00000636051 | down | -0.7482106 | 0.0280353 |
| GPBP1 | ENST00000264779 | down | -0.7485821 | 0.0184987 |
| NRIP1 | ENST00000400202 | down | -0.7489777 | 0.0208104 |
| LITAF | ENST00000622633 | down | -0.7491976 | 0.0150117 |
| LMTK3 | ENST00000270238 | down | -0.7493099 | 0.0414452 |
| MBIP | ENST00000359527 | down | -0.7496943 | 0.0257102 |
| PARP3 | ENST00000398755 | down | -0.749892 | 0.0271161 |
| P2RY4 | ENST00000374519 | down | -0.7502343 | 0.02919 |
| RPL23A | ENST00000422514 | down | -0.750321 | 0.0019902 |
| DUS1L | ENST00000354321 | down | -0.7503956 | 0.0276504 |
| CHCHD3 | ENST00000481152 | down | -0.7504188 | 0.0149046 |
| INTS12 | ENST00000340139 | down | -0.7511446 | 0.0198533 |
| PARD3 | ENST00000374789 | down | -0.7513138 | 0.0337204 |
| ZNF185 | ENST00000318529 | down | -0.7513218 | 0.0243526 |
| MARK3 | ENST00000553942 | down | -0.7518752 | 0.0450711 |
| COL6A3 | ENST00000472056 | down | -0.7520333 | 0.0104257 |
| DPM1 | ENST00000371582 | down | -0.752128 | 0.0077553 |
| NCBP2 | ENST00000447325 | down | -0.7522847 | 0.0081847 |
| IL15 | ENST00000320650 | down | -0.7529755 | 0.0311487 |
| CCDC34 | ENST00000317945 | down | -0.7531117 | 0.0008725 |
| NOC3L | ENST00000371361 | down | -0.7534676 | 0.0366928 |
| FAM120AOS | ENST00000423591 | down | -0.7537346 | 0.0347202 |
| TCF12 | ENST00000537840 | down | -0.7538932 | 0.0365907 |
| NAT1 | ENST00000517492 | down | -0.7540528 | 0.0138113 |
| MAP4K2 | ENST00000377350 | down | -0.7542293 | 0.0485196 |
| APTX | ENST00000309615 | down | -0.75445 | 0.0288706 |
| DTNBP1 | ENST00000344537 | down | -0.7546946 | 0.0152686 |
| PPP1R12B | ENST00000608999 | down | -0.7553832 | 0.0074518 |
| GYG1 | ENST00000484197 | down | -0.7554124 | 0.0085923 |
| ALKBH5 | ENST00000399138 | down | -0.755626 | 0.0396804 |
| CSF1 | ENST00000369801 | down | -0.7556544 | 0.00439 |
| CACNB1 | ENST00000394303 | down | -0.7565392 | 0.0025069 |
| GATB | ENST00000263985 | down | -0.7566513 | 0.0169411 |
| ATRAID | ENST00000405489 | down | -0.7577333 | 0.0462315 |
| GPATCH11 | ENST00000409774 | down | -0.7582522 | 0.0150518 |
| TWNK | ENST00000370228 | down | -0.7585463 | 0.0193568 |
| CDC20 | ENST00000310955 | down | -0.7585737 | 0.0474081 |
| TOM1L1 | ENST00000575882 | down | -0.7586793 | 0.045855 |
| SRSF11 | ENST00000370950 | down | -0.7588086 | 0.0052506 |
| UBE2I | ENST00000325437 | down | -0.7590156 | 0.0080087 |
| TMC6 | ENST00000392467 | down | -0.7593599 | 0.0235605 |
| TLK2 | NM_001284363 | down | -0.7595181 | 0.0268313 |
| ALDH3A1 | ENST00000457500 | down | -0.7595702 | 0.0056814 |
| RPH3A | ENST00000389385 | down | -0.7598445 | 0.0129687 |
| BOLA3 | ENST00000295326 | down | -0.7599588 | 0.0289286 |
| RP11-205M20.3 | ENST00000644531 | down | -0.7603568 | 0.006886 |
| SLAMF1 | ENST00000538290 | down | -0.7604019 | 0.0037817 |
| DUSP3 | ENST00000226004 | down | -0.7604255 | 0.0071646 |
| ZNF714 | ENST00000596143 | down | -0.7604991 | 0.0433747 |
| CHCHD7 | ENST00000518801 | down | -0.7613842 | 0.0014152 |
| NAA50 | ENST00000493900 | down | -0.7621505 | 0.0400497 |
| RANBP3 | ENST00000439268 | down | -0.7622973 | 0.0133056 |
| NT5C3A | ENST00000405342 | down | -0.7623203 | 0.0053096 |
| TAB1 | uc003axr.3 | down | -0.7623319 | 0.0292926 |
| TOLLIP | ENST00000317204 | down | -0.7627889 | 0.0126534 |
| CGGBP1 | ENST00000462901 | down | -0.7641015 | 0.0208283 |
| SBDS | ENST00000246868 | down | -0.7641682 | 0.0415735 |
| SELENBP1 | ENST00000368868 | down | -0.764329 | 0.0346293 |
| DENND1A | NM_001352964 | down | -0.764375 | 0.0267774 |
| TUFM | ENST00000313511 | down | -0.7647026 | 0.0119517 |
| CD22 | ENST00000544992 | down | -0.7651768 | 0.0176409 |
| TFB1M | ENST00000367166 | down | -0.7653354 | 0.0036153 |
| POLR2J | ENST00000292614 | down | -0.765539 | 0.0205154 |
| ERMARD | ENST00000366773 | down | -0.765582 | 0.0167172 |
| MKRN2 | ENST00000170447 | down | -0.7656494 | 0.0377946 |
| NENF | ENST00000366988 | down | -0.7656935 | 0.0248722 |
| H3F3B | ENST00000254810 | down | -0.7661458 | 0.0154389 |
| TMEM209 | ENST00000397622 | down | -0.7661825 | 0.0221477 |
| TMEM101 | ENST00000589334 | down | -0.7663202 | 0.0469328 |
| NUDT2 | ENST00000618590 | down | -0.7668198 | 0.0306432 |
| NCAN | ENST00000252575 | down | -0.767077 | 0.0273322 |
| UXS1 | ENST00000283148 | down | -0.7686432 | 0.002553 |
| MKNK1 | ENST00000371945 | down | -0.7686848 | 0.0369916 |
| TMEM5 | ENST00000261234 | down | -0.7690141 | 0.0413035 |
| METTL15 | NM_152636 | down | -0.7692926 | 0.0215781 |
| TMEM158 | ENST00000503771 | down | -0.7693693 | 0.0246135 |
| KAT8 | ENST00000219797 | down | -0.769464 | 0.0297322 |
| RBM39 | uc010gfn.3 | down | -0.7694792 | 0.0160602 |
| RAP1GDS1 | ENST00000264572 | down | -0.7694961 | 0.0190425 |
| NEDD9 | ENST00000620854 | down | -0.7699852 | 0.028294 |
| MGEA5 | ENST00000439817 | down | -0.7701778 | 0.0093455 |
| MIS12 | ENST00000381165 | down | -0.7707379 | 0.0208569 |
| FETUB | ENST00000265029 | down | -0.7708808 | 0.0137949 |
| MNS1 | ENST00000260453 | down | -0.7710465 | 0.0497504 |
| QTRT1 | ENST00000250237 | down | -0.7711024 | 0.0015325 |
| SUPT7L | ENST00000405491 | down | -0.7713811 | 0.0018306 |
| MRPL33 | ENST00000379666 | down | -0.7716328 | 0.0291976 |
| TSTD2 | ENST00000341170 | down | -0.7717083 | 0.0407053 |
| AC064853.3 | ENST00000642055 | down | -0.7719362 | 0.0160729 |
| WDR31 | ENST00000374193 | down | -0.7721547 | 0.0136029 |
| C21orf59 | ENST00000290155 | down | -0.772259 | 0.0215562 |
| RIOK1 | ENST00000379834 | down | -0.772279 | 0.0133017 |
| RPL35 | ENST00000348462 | down | -0.7725986 | 0.0026214 |
| ABHD17B | ENST00000333421 | down | -0.7728255 | 0.0467263 |
| CANX | ENST00000247461 | down | -0.7730356 | 0.0148922 |
| XXbac-BPG32J3.22 | ENST00000375880 | down | -0.7730877 | 0.0054623 |
| TRMT1 | ENST00000357720 | down | -0.7732636 | 0.0192033 |
| FAM168A | ENST00000356467 | down | -0.7733765 | 0.0329009 |
| ZFAND2A | ENST00000316495 | down | -0.7736141 | 0.0042048 |
| CD46 | ENST00000358170 | down | -0.7740066 | 0.0139436 |
| SRP9 | ENST00000366839 | down | -0.7741933 | 0.0001701 |
| OSBPL3 | ENST00000409069 | down | -0.774272 | 0.030466 |
| NDUFC1 | ENST00000505036 | down | -0.7747076 | 0.0048882 |
| FAM136A | NM_001329758 | down | -0.7749299 | 0.0267804 |
| AGA | NM_001171988 | down | -0.7749659 | 0.0223561 |
| DUX4 | ENST00000556625 | down | -0.7750314 | 0.0048624 |
| ZNF706 | ENST00000521272 | down | -0.7761077 | 0.0408165 |
| MYCL | ENST00000372816 | down | -0.7762787 | 0.0033213 |
| RANGRF | ENST00000226105 | down | -0.7763755 | 0.0009829 |
| DUSP1 | ENST00000239223 | down | -0.7763978 | 0.0489392 |
| PCID2 | NM_001353095 | down | -0.7764402 | 0.0132862 |
| PSMG4 | ENST00000438998 | down | -0.7764556 | 0.0089041 |
| GLMP | ENST00000612353 | down | -0.7766363 | 0.024418 |
| RTN3 | ENST00000354497 | down | -0.7773183 | 0.0016068 |
| MGMT | ENST00000306010 | down | -0.7774369 | 0.0054754 |
| PIPOX | ENST00000323372 | down | -0.7776281 | 0.0020933 |
| KIAA1551 | ENST00000312561 | down | -0.7779777 | 0.0198145 |
| NDRG4 | ENST00000394282 | down | -0.778052 | 0.0312612 |
| PCK2 | ENST00000561286 | down | -0.778369 | 0.0152145 |
| TXN2 | ENST00000216185 | down | -0.7784037 | 0.0123657 |
| TMEM136 | NM_001198674 | down | -0.77857 | 0.015524 |
| STAT5A | ENST00000345506 | down | -0.7792272 | 0.0192904 |
| N4BP2L2 | ENST00000504114 | down | -0.7794677 | 0.0448666 |
| ELMO2 | uc002xrs.1 | down | -0.7798111 | 0.041424 |
| C14orf159 | ENST00000520328 | down | -0.7803109 | 0.003271 |
| C2orf49 | ENST00000258457 | down | -0.7808415 | 0.0134984 |
| ELAC1 | ENST00000269466 | down | -0.7812732 | 0.0216747 |
| BAX | ENST00000539787 | down | -0.7813477 | 0.0373912 |
| CDC14B | ENST00000375241 | down | -0.7819872 | 0.0456508 |
| TMX2 | ENST00000278422 | down | -0.7829508 | 0.0346142 |
| RSBN1 | ENST00000261441 | down | -0.7830465 | 0.040282 |
| ZNF160 | ENST00000601421 | down | -0.7835474 | 0.0116719 |
| POLDIP2 | ENST00000618887 | down | -0.7837548 | 0.019475 |
| CNIH4 | NM_001277198 | down | -0.7840289 | 0.0001317 |
| RRP36 | NM_001329704 | down | -0.78463 | 0.0334285 |
| PLEKHB2 | NM_001100623 | down | -0.7848533 | 0.0459559 |
| HSPD1 | uc010fsm.3 | down | -0.7850196 | 0.0122805 |
| UPF3A | ENST00000351487 | down | -0.7851876 | 0.0045051 |
| C10orf105 | ENST00000398786 | down | -0.7853887 | 0.0048653 |
| ZFAND6 | ENST00000559157 | down | -0.7855753 | 0.0129039 |
| MTMR3 | ENST00000351488 | down | -0.7856777 | 0.0405189 |
| ATP5SL | ENST00000221943 | down | -0.7868313 | 0.0036421 |
| POLR2E | ENST00000585838 | down | -0.7877907 | 0.0395612 |
| POLR3C | ENST00000334163 | down | -0.7881965 | 0.0309221 |
| ZMYM5 | ENST00000382905 | down | -0.7884781 | 0.0258248 |
| EIF2B2 | ENST00000266126 | down | -0.7888976 | 0.0475437 |
| DNAL4 | ENST00000216068 | down | -0.7889157 | 0.009841 |
| EMILIN3 | ENST00000332312 | down | -0.789092 | 0.0125822 |
| HEXB | ENST00000511181 | down | -0.7897442 | 0.0386027 |
| IAH1 | ENST00000497473 | down | -0.7899665 | 0.0441058 |
| JRKL | ENST00000332349 | down | -0.7900135 | 0.0386739 |
| SURF2 | NM_001278928 | down | -0.7906125 | 0.0299009 |
| TRAPPC11 | ENST00000357207 | down | -0.7908456 | 0.0133529 |
| SCP2 | ENST00000407246 | down | -0.7909006 | 0.0185299 |
| PPIL3 | ENST00000392283 | down | -0.7909624 | 0.0142803 |
| BAG1 | ENST00000379704 | down | -0.790973 | 0.0369291 |
| PIGBOS1 | ENST00000436697 | down | -0.7912613 | 0.0267443 |
| GMNN | ENST00000620958 | down | -0.7914834 | 0.0035682 |
| PDE4D | ENST00000317118 | down | -0.7916122 | 0.0133639 |
| SOCS6 | ENST00000397942 | down | -0.7916449 | 0.0222344 |
| MAPKAP1 | ENST00000373498 | down | -0.7919399 | 0.0393695 |
| MAPKAPK5 | ENST00000551404 | down | -0.7924819 | 0.0146975 |
| ZNF567 | ENST00000360729 | down | -0.7930447 | 0.0333858 |
| NQO2 | ENST00000380454 | down | -0.7932112 | 0.0004357 |
| TRMT112 | ENST00000544844 | down | -0.7937819 | 0.0329802 |
| TAF9 | ENST00000328663 | down | -0.7945266 | 0.0157274 |
| MGRN1 | ENST00000399577 | down | -0.7946029 | 0.0358001 |
| MTCH2 | NM_001317232 | down | -0.7947444 | 0.0184243 |
| LAMTOR4 | ENST00000474141 | down | -0.7947523 | 0.0155422 |
| RNF146 | ENST00000610153 | down | -0.7953687 | 0.0016262 |
| SH2B3 | ENST00000341259 | down | -0.7961156 | 0.0391403 |
| EPB41 | ENST00000373800 | down | -0.796282 | 0.0368653 |
| LSM10 | ENST00000315732 | down | -0.7964268 | 0.0121243 |
| UBLCP1 | ENST00000296786 | down | -0.796809 | 0.0103401 |
| RAD23A | ENST00000316856 | down | -0.7969066 | 0.0324902 |
| ZFP64 | ENST00000361387 | down | -0.7969162 | 0.0172844 |
| NEK1 | ENST00000507142 | down | -0.7969699 | 0.0499909 |
| ZFAND6 | ENST00000564367 | down | -0.7970291 | 0.0009378 |
| APOL1 | uc010gwx.3 | down | -0.797194 | 0.0094034 |
| CDV3 | ENST00000515421 | down | -0.7982314 | 0.0146451 |
| SAE1 | ENST00000413379 | down | -0.7983123 | 0.0456987 |
| CAMK2D | ENST00000511664 | down | -0.7983839 | 0.0057139 |
| RP11-92K2.2 | ENST00000647225 | down | -0.7985456 | 0.0164273 |
| TMEM5 | ENST00000537373 | down | -0.7985566 | 0.0314657 |
| IRX2 | ENST00000382611 | down | -0.7992305 | 0.0125073 |
| PGPEP1 | ENST00000269919 | down | -0.7994838 | 0.0123396 |
| PARD3 | ENST00000374773 | down | -0.7995224 | 0.0261514 |
| TNFRSF10D | ENST00000312584 | down | -0.7999334 | 0.0071421 |
| CLTA | NM_001311206 | down | -0.7999977 | 0.0010739 |
| WBSCR22 | ENST00000423497 | down | -0.8000261 | 0.0013636 |
| CCDC90B | ENST00000455220 | down | -0.8000708 | 0.0185391 |
| IMMT | ENST00000442664 | down | -0.8000902 | 0.0232877 |
| ASCL5 | ENST00000449188 | down | -0.8009052 | 0.0489173 |
| ZNF385D | ENST00000281523 | down | -0.8017415 | 0.0159005 |
| BECN1 | ENST00000590099 | down | -0.8021779 | 0.0262367 |
| UCP2 | ENST00000310473 | down | -0.8021806 | 0.0323706 |
| PATZ1 | ENST00000405309 | down | -0.802273 | 0.0216458 |
| CDK5RAP1 | uc002wzc.2 | down | -0.8023078 | 0.0033026 |
| FEZ1 | ENST00000278919 | down | -0.8025132 | 0.0444907 |
| ZNF551 | ENST00000282296 | down | -0.8025529 | 0.0291222 |
| PSMA5 | NM_001199772 | down | -0.8027554 | 0.036647 |
| MARC2 | ENST00000359316 | down | -0.8029138 | 0.0237376 |
| DBT | ENST00000370132 | down | -0.8032517 | 0.0082402 |
| TAS1R3 | ENST00000339381 | down | -0.8032634 | 0.0102148 |
| RNF112 | ENST00000461366 | down | -0.8035218 | 0.0282161 |
| TMEM65 | ENST00000297632 | down | -0.8035634 | 0.0313229 |
| PLCD1 | ENST00000334661 | down | -0.8036584 | 0.0231961 |
| STX17 | ENST00000259400 | down | -0.8037289 | 0.0249559 |
| GLYR1 | ENST00000589389 | down | -0.804182 | 0.0390686 |
| AK3 | ENST00000381809 | down | -0.8041965 | 0.038836 |
| SP100 | ENST00000409341 | down | -0.8043527 | 0.0063038 |
| VPS72 | ENST00000368892 | down | -0.8045317 | 0.0279033 |
| ADNP2 | NM_014913 | down | -0.8049024 | 0.0382568 |
| CDK18 | ENST00000360066 | down | -0.8053594 | 0.0445348 |
| RPS9 | ENST00000391751 | down | -0.8056496 | 0.0041241 |
| RPF1 | ENST00000370654 | down | -0.806266 | 0.0233339 |
| SGSH | ENST00000326317 | down | -0.8068581 | 0.0258126 |
| TMEM92 | ENST00000507382 | down | -0.806897 | 0.0049195 |
| UBE2L6 | ENST00000287156 | down | -0.806964 | 0.0252814 |
| CS | ENST00000351328 | down | -0.8069982 | 0.034673 |
| UGGT2 | ENST00000376747 | down | -0.8070623 | 0.0369929 |
| ZCCHC9 | ENST00000380199 | down | -0.8079746 | 0.0152371 |
| FBXO36 | ENST00000283946 | down | -0.8081023 | 0.0222599 |
| GCLC | ENST00000513939 | down | -0.8083031 | 0.0307287 |
| NRIP1 | ENST00000400199 | down | -0.8084226 | 0.0382573 |
| MLLT10 | NM_001195628 | down | -0.8086784 | 0.0297416 |
| SYK | ENST00000375754 | down | -0.8088357 | 0.0341829 |
| ASPH | ENST00000541428 | down | -0.8088978 | 0.0424503 |
| CLUAP1 | ENST00000572600 | down | -0.8095557 | 0.0486212 |
| FARP1 | ENST00000376581 | down | -0.8095568 | 0.0293583 |
| LETMD1 | ENST00000418425 | down | -0.8099704 | 0.0431914 |
| PTPMT1 | ENST00000426530 | down | -0.8103082 | 0.0394628 |
| MAP3K12 | ENST00000547488 | down | -0.8104988 | 0.0033559 |
| CPEB2 | ENST00000538197 | down | -0.8105777 | 0.0048777 |
| ZNF580 | ENST00000325333 | down | -0.8106815 | 0.0117773 |
| PSMG2 | ENST00000585331 | down | -0.8106924 | 0.008984 |
| MTMR3 | ENST00000333027 | down | -0.8108562 | 0.0204206 |
| PRDX6 | ENST00000340385 | down | -0.8110192 | 0.0358247 |
| TSC22D2 | ENST00000466814 | down | -0.811158 | 0.0492928 |
| CPA1 | ENST00000011292 | down | -0.8118628 | 0.0241622 |
| PRKAR2A | ENST00000265563 | down | -0.8120726 | 0.0237258 |
| SLC7A5 | ENST00000261622 | down | -0.8133307 | 0.0031349 |
| BIN1 | NM_001320640 | down | -0.8139466 | 0.0442213 |
| GRK6 | ENST00000355958 | down | -0.8140782 | 0.0084167 |
| CFDP1 | ENST00000283882 | down | -0.8141246 | 0.0308587 |
| RAP1B | ENST00000450214 | down | -0.814326 | 0.0144783 |
| FLYWCH1 | NM_020912 | down | -0.8143641 | 0.0390133 |
| CEP290 | ENST00000552810 | down | -0.8143807 | 0.0471209 |
| CHPT1 | ENST00000549872 | down | -0.8145725 | 0.003509 |
| XRRA1 | ENST00000527087 | down | -0.8147087 | 0.0185648 |
| C14orf93 | ENST00000341470 | down | -0.8149817 | 0.0125225 |
| CUL5 | ENST00000393094 | down | -0.8150552 | 0.0449904 |
| ALKBH2 | ENST00000343075 | down | -0.8151127 | 0.0282195 |
| HMGXB3 | ENST00000613459 | down | -0.815563 | 0.0245262 |
| TMEM185A | ENST00000600449 | down | -0.8156746 | 0.0210259 |
| GTPBP8 | ENST00000383677 | down | -0.8158612 | 0.0178814 |
| SMNDC1 | ENST00000369603 | down | -0.8159647 | 0.023613 |
| GOLGA4 | ENST00000356847 | down | -0.8161947 | 0.0195872 |
| VPRBP | ENST00000504652 | down | -0.8162581 | 0.0454463 |
| RNF5 | ENST00000375094 | down | -0.8163874 | 0.0467228 |
| GSS | ENST00000643188 | down | -0.8164587 | 0.0271361 |
| ZNF256 | ENST00000282308 | down | -0.8165005 | 0.0224581 |
| SPDYE4 | ENST00000328794 | down | -0.8172159 | 0.0115924 |
| FAM173B | ENST00000510047 | down | -0.8176671 | 0.0018364 |
| KIAA0930 | ENST00000336156 | down | -0.8177912 | 0.0231078 |
| OSGIN2 | ENST00000451899 | down | -0.818045 | 0.0406777 |
| CNGA4 | uc001mcn.3 | down | -0.8183696 | 0.0132588 |
| THRA | ENST00000394121 | down | -0.8184316 | 0.0114762 |
| ZNF595 | ENST00000608255 | down | -0.8189059 | 0.0460509 |
| DHRS4 | ENST00000397074 | down | -0.819055 | 0.0279891 |
| FAM45A | NM_001303112 | down | -0.8190785 | 0.0364515 |
| ATP6V0A1 | ENST00000343619 | down | -0.8190897 | 0.0179712 |
| MOB4 | ENST00000448447 | down | -0.8191281 | 0.0096656 |
| TMEM80 | ENST00000397512 | down | -0.8191441 | 0.0359969 |
| TPRA1 | ENST00000296210 | down | -0.8192993 | 0.0102883 |
| MOCS2 | ENST00000450852 | down | -0.8196779 | 0.0015263 |
| RBBP8 | ENST00000399725 | down | -0.8201889 | 0.0493855 |
| GSR | ENST00000546342 | down | -0.8202578 | 0.0163368 |
| BFSP1 | NM_001278607 | down | -0.820509 | 0.010488 |
| PEX7 | ENST00000318471 | down | -0.8208682 | 0.0373787 |
| RBM26 | ENST00000622611 | down | -0.8210162 | 0.0419799 |
| LARS | ENST00000510191 | down | -0.8214556 | 0.0189418 |
| HSCB | NM_001318314 | down | -0.8215314 | 0.0242893 |
| ALX1 | ENST00000316824 | down | -0.8221328 | 0.0151789 |
| PSMD9 | ENST00000542602 | down | -0.822214 | 0.0180988 |
| MCCC2 | ENST00000340941 | down | -0.8222468 | 0.0422182 |
| CATG00000010761.1 | HBMT00000319848 | down | -0.8222648 | 0.0197947 |
| TMEM230 | ENST00000615008 | down | -0.8233835 | 0.0240883 |
| GLRB | ENST00000541722 | down | -0.8244405 | 0.0279704 |
| BGLAP | ENST00000368272 | down | -0.8253899 | 0.0343699 |
| NBDY | ENST00000374922 | down | -0.8263531 | 0.0181089 |
| MLX | ENST00000435881 | down | -0.8270621 | 0.0134604 |
| WBP2 | ENST00000254806 | down | -0.8278539 | 0.0089903 |
| FNBP4 | ENST00000263773 | down | -0.8281819 | 0.0345488 |
| DCPS | NM_001350236 | down | -0.8283058 | 0.0240233 |
| SRSF11 | NM_001350607 | down | -0.8286892 | 0.0141999 |
| GPATCH11 | NM_001322249 | down | -0.8288808 | 0.0062175 |
| PIEZO2 | ENST00000383408 | down | -0.8289669 | 0.0149408 |
| LEAP2 | ENST00000296877 | down | -0.8289899 | 0.0264141 |
| CSTB | ENST00000291568 | down | -0.8291401 | 0.031656 |
| IL4R | ENST00000543915 | down | -0.8291419 | 0.0238393 |
| SLC22A6 | ENST00000360421 | down | -0.8292741 | 0.034471 |
| XKR7 | ENST00000562532 | down | -0.8293386 | 0.0336471 |
| AC018755.1 | ENST00000301439 | down | -0.8295078 | 0.0350126 |
| BTF3L4 | ENST00000472944 | down | -0.8295441 | 0.0259968 |
| EPHA5 | ENST00000354839 | down | -0.8297019 | 0.0414757 |
| PDCD2 | ENST00000392090 | down | -0.8300858 | 0.0029655 |
| AMD1 | ENST00000368882 | down | -0.8300912 | 0.0469872 |
| COMMD2 | ENST00000473414 | down | -0.8302197 | 0.0236309 |
| ANKRD26 | ENST00000376087 | down | -0.8303421 | 0.0080974 |
| GCAT | ENST00000323205 | down | -0.8307034 | 0.001536 |
| RTN4 | ENST00000394611 | down | -0.8310025 | 0.0334744 |
| CEP57L1 | ENST00000368968 | down | -0.8310436 | 0.0063355 |
| INIP | NM_001329585 | down | -0.8313465 | 0.0347106 |
| EIF3K | ENST00000248342 | down | -0.8316349 | 0.0348097 |
| SLC41A3 | ENST00000315891 | down | -0.83169 | 0.025245 |
| PPIL4 | ENST00000253329 | down | -0.8317577 | 0.024032 |
| RBMX | ENST00000320676 | down | -0.831825 | 0.0190975 |
| HNRNPLL | ENST00000449105 | down | -0.8318368 | 0.0188831 |
| TYSND1 | ENST00000335494 | down | -0.8318762 | 0.0073125 |
| SYNRG | ENST00000614941 | down | -0.8319791 | 0.0161516 |
| PCYT1B | ENST00000379145 | down | -0.8321492 | 0.0396595 |
| DDX50 | ENST00000373585 | down | -0.8322384 | 0.0121466 |
| CEBPZ | ENST00000234170 | down | -0.8326518 | 0.0249212 |
| NDUFB10 | ENST00000268668 | down | -0.8326765 | 0.016528 |
| ZBTB6 | ENST00000373659 | down | -0.8327198 | 0.0041313 |
| BROX | ENST00000537020 | down | -0.8332386 | 0.0050793 |
| CXorf40A | NM_001324276 | down | -0.8334643 | 0.0261583 |
| ARL2BP | ENST00000219204 | down | -0.8338919 | 0.001212 |
| E2F6 | ENST00000381525 | down | -0.834122 | 0.0480884 |
| MEIS3 | ENST00000561293 | down | -0.8343149 | 0.0400478 |
| PHB | ENST00000617874 | down | -0.8346413 | 0.0348157 |
| SWI5 | ENST00000419867 | down | -0.8346851 | 0.0007145 |
| AHSG | ENST00000411641 | down | -0.8350295 | 0.0110368 |
| TOMM5 | ENST00000321301 | down | -0.8353781 | 0.0293652 |
| MRPL52 | ENST00000355151 | down | -0.8359493 | 0.0385002 |
| FAM13A | ENST00000503556 | down | -0.8361547 | 0.0461394 |
| TAX1BP1 | ENST00000396319 | down | -0.8362138 | 0.0109995 |
| FKBP4 | ENST00000001008 | down | -0.836216 | 0.0408737 |
| GALC | ENST00000261304 | down | -0.836296 | 0.0356533 |
| SCAF8 | ENST00000417268 | down | -0.8364231 | 0.0015119 |
| TMEM14B | ENST00000379542 | down | -0.8366867 | 0.0058011 |
| MTFR1 | ENST00000262146 | down | -0.8366871 | 0.0369492 |
| NEK4 | ENST00000535191 | down | -0.8369528 | 0.0361665 |
| CUTA | ENST00000611509 | down | -0.8369864 | 0.0177066 |
| MAST2 | NM_001324321 | down | -0.8369991 | 0.0186418 |
| ZNF784 | ENST00000325351 | down | -0.8371638 | 0.0322964 |
| AP3D1 | ENST00000345016 | down | -0.8371881 | 0.0184677 |
| DR1 | ENST00000370267 | down | -0.8372404 | 0.0006443 |
| NDUFB5 | ENST00000611971 | down | -0.8374664 | 0.0001726 |
| LIAS | ENST00000340169 | down | -0.8377351 | 0.0141768 |
| PRKRIP1 | ENST00000397912 | down | -0.8377935 | 0.0280443 |
| TARM1 | ENST00000432826 | down | -0.8378141 | 0.0239283 |
| APP | ENST00000354192 | down | -0.8378196 | 0.0348864 |
| HEBP1 | ENST00000536942 | down | -0.8379164 | 0.0071867 |
| PSMG1 | ENST00000380900 | down | -0.838124 | 0.0295179 |
| PLEKHB2 | ENST00000438882 | down | -0.8383941 | 0.041953 |
| HTR3A | ENST00000355556 | down | -0.8387057 | 0.0473298 |
| SAMD4B | ENST00000598913 | down | -0.838712 | 0.0407112 |
| THOC1 | ENST00000261600 | down | -0.8390128 | 0.0338904 |
| FSTL4 | ENST00000265342 | down | -0.8390265 | 0.0178097 |
| SPG20 | ENST00000438666 | down | -0.8391674 | 0.0286702 |
| CATG00000075886.1 | ENCT00000344450 | down | -0.8393236 | 0.0208434 |
| FRMPD4 | uc011mij.2 | down | -0.8393673 | 0.0315651 |
| FHOD3 | ENST00000359247 | down | -0.8394091 | 0.0098603 |
| AP2A2 | ENST00000332231 | down | -0.8394904 | 0.0010529 |
| NUP133 | ENST00000261396 | down | -0.8397025 | 0.0399068 |
| MAP3K13 | ENST00000446828 | down | -0.8398284 | 0.0004208 |
| IL4R | ENST00000170630 | down | -0.839841 | 0.0335645 |
| DNPH1 | ENST00000393987 | down | -0.839882 | 0.0361792 |
| SKOR1 | ENST00000380035 | down | -0.8401465 | 0.0354295 |
| SLC35B1 | ENST00000240333 | down | -0.8401682 | 0.0451798 |
| PHYHIP | ENST00000321613 | down | -0.8404831 | 0.0069007 |
| C14orf156 | ENST00000238688 | down | -0.8404836 | 0.0002356 |
| VGLL4 | ENST00000273038 | down | -0.840571 | 0.0013142 |
| PPT1 | ENST00000449045 | down | -0.8408326 | 0.0009511 |
| C12orf73 | ENST00000378090 | down | -0.8411616 | 0.0002508 |
| RAP1B | ENST00000393436 | down | -0.8419876 | 0.0028918 |
| GLRX2 | ENST00000367439 | down | -0.8427198 | 0.0376561 |
| ZNF124 | NM_001297569 | down | -0.8427736 | 0.0085616 |
| DTNBP1 | ENST00000355917 | down | -0.8429824 | 0.0082037 |
| VPS13C | ENST00000249837 | down | -0.8432585 | 0.0251897 |
| CHMP5 | ENST00000223500 | down | -0.8434037 | 0.0005758 |
| PBDC1 | ENST00000373358 | down | -0.843694 | 0.006828 |
| QDPR | ENST00000281243 | down | -0.8438158 | 0.000542 |
| MOCS3 | ENST00000244051 | down | -0.8439966 | 0.0163695 |
| BCLAF1 | ENST00000531224 | down | -0.8441576 | 0.043162 |
| USP6 | ENST00000250066 | down | -0.8445129 | 0.0324205 |
| MRPS33 | ENST00000324787 | down | -0.8445254 | 0.0037104 |
| UNC50 | ENST00000357765 | down | -0.8445476 | 0.0221148 |
| ATP2A2 | ENST00000308664 | down | -0.844561 | 0.0374494 |
| FCF1 | ENST00000534938 | down | -0.8445632 | 0.0028694 |
| CHTOP | ENST00000368686 | down | -0.8446037 | 0.0085734 |
| GPR161 | ENST00000537209 | down | -0.8448214 | 0.0377199 |
| C14orf156 | ENST00000613856 | down | -0.8448799 | 0.0002285 |
| C1orf123 | ENST00000294360 | down | -0.8451244 | 0.0181509 |
| SLC25A46 | ENST00000447245 | down | -0.8455359 | 0.0089991 |
| ABLIM1 | ENST00000392952 | down | -0.8456592 | 0.0127557 |
| TPMT | ENST00000309983 | down | -0.8461063 | 0.036974 |
| PDGFRL | ENST00000541323 | down | -0.846331 | 0.0204337 |
| TMEM68 | ENST00000434581 | down | -0.8465269 | 0.044496 |
| MTIF3 | ENST00000381120 | down | -0.8466121 | 0.0363519 |
| PPP4R2 | NM_001318026 | down | -0.8466124 | 0.0295523 |
| CASK | ENST00000644347 | down | -0.8472121 | 0.0175745 |
| DPH3 | ENST00000488423 | down | -0.8474249 | 0.0333185 |
| REPS2 | ENST00000303843 | down | -0.8475954 | 0.0202602 |
| HINT3 | ENST00000229633 | down | -0.8484012 | 0.0421551 |
| DAP3 | ENST00000471642 | down | -0.8484832 | 0.0154886 |
| CTNS | uc010vrv.2 | down | -0.8494163 | 0.0393991 |
| IL34 | ENST00000429149 | down | -0.8498552 | 0.0313049 |
| ZNF497 | ENST00000311044 | down | -0.8499741 | 0.0124151 |
| WASF1 | ENST00000359451 | down | -0.8501607 | 0.0366347 |
| ZNF3 | ENST00000299667 | down | -0.850541 | 0.0338286 |
| PLEKHB2 | ENST00000628582 | down | -0.8512042 | 0.0169607 |
| PKMYT1 | ENST00000574385 | down | -0.8513916 | 0.0152776 |
| SLC29A2 | ENST00000544554 | down | -0.8514144 | 0.0172185 |
| CCDC51 | NM_024661 | down | -0.8514436 | 0.0106059 |
| PROZ | ENST00000375547 | down | -0.8518843 | 0.0246461 |
| ACOT13 | ENST00000537591 | down | -0.8519846 | 0.0038975 |
| FHIT | ENST00000468189 | down | -0.8525866 | 0.0109855 |
| MARK4 | ENST00000262891 | down | -0.8526619 | 0.0148585 |
| SYT15 | ENST00000503753 | down | -0.8533082 | 0.0067508 |
| GEMIN2 | ENST00000396249 | down | -0.853417 | 0.0001038 |
| ATP2C1 | ENST00000513801 | down | -0.8535192 | 0.0193982 |
| PSMB2 | ENST00000373237 | down | -0.8535327 | 0.0104284 |
| ICAM2 | ENST00000418105 | down | -0.853795 | 0.0093498 |
| HMBS | ENST00000544387 | down | -0.8543628 | 0.015803 |
| HKR1 | NM_001329762 | down | -0.8549029 | 0.0160356 |
| PIAS2 | NM_001354033 | down | -0.8551927 | 0.0317643 |
| HSD11B1L | ENST00000616276 | down | -0.855271 | 0.0328968 |
| SRP68 | ENST00000539137 | down | -0.8558793 | 0.0484947 |
| CDK5 | ENST00000485972 | down | -0.85594 | 0.0139848 |
| TMEM147 | ENST00000222284 | down | -0.8563838 | 0.0073188 |
| RGS19 | ENST00000395042 | down | -0.8564004 | 0.0382759 |
| SENP3 | ENST00000321337 | down | -0.8569353 | 0.0168054 |
| SENP3 | ENST00000321337 | down | -0.8569353 | 0.0168054 |
| MAST1 | ENST00000251472 | down | -0.8574183 | 0.0129758 |
| ARHGAP26 | ENST00000645722 | down | -0.8575761 | 0.026222 |
| ZNF543 | ENST00000321545 | down | -0.8577537 | 0.0250212 |
| SERPINF1 | ENST00000254722 | down | -0.8581839 | 0.010756 |
| TRIT1 | ENST00000316891 | down | -0.8583635 | 0.0419196 |
| ALDH3B2 | ENST00000349015 | down | -0.8584129 | 0.0029929 |
| MLLT10 | ENST00000377059 | down | -0.8584625 | 0.0194636 |
| LCE1A | ENST00000335123 | down | -0.8585904 | 0.0077018 |
| APLP1 | NM_005166 | down | -0.8587474 | 0.0468627 |
| RBM17 | ENST00000379888 | down | -0.8587553 | 0.0231179 |
| PARP9 | ENST00000462315 | down | -0.8588069 | 0.0313958 |
| TTC13 | ENST00000366662 | down | -0.8588542 | 0.0186103 |
| COMMD7 | ENST00000446419 | down | -0.8592696 | 0.0021689 |
| ELP5 | ENST00000574993 | down | -0.8595615 | 0.030875 |
| CNPPD1 | ENST00000409789 | down | -0.8596914 | 0.0445688 |
| C8orf59 | ENST00000614462 | down | -0.8598646 | 0.0257556 |
| LY6G6D | ENST00000375824 | down | -0.8600758 | 0.0052435 |
| IMPDH2 | ENST00000326739 | down | -0.8604085 | 0.0098501 |
| HSDL2 | ENST00000398803 | down | -0.8607322 | 0.0388732 |
| AP3S2 | ENST00000336418 | down | -0.8608398 | 0.0092325 |
| CATG00000031212.1 | HBMT00000597373 | down | -0.8610915 | 0.0123201 |
| CARM1 | ENST00000327064 | down | -0.8616172 | 0.0144519 |
| ZNF32 | NM_001324166 | down | -0.8617144 | 0.0247203 |
| PEPD | ENST00000244137 | down | -0.8618007 | 0.0041295 |
| AMZ2 | ENST00000612294 | down | -0.8618204 | 0.0413769 |
| ANKRD10 | ENST00000267339 | down | -0.8620087 | 0.0179153 |
| EYA4 | ENST00000431403 | down | -0.8622803 | 0.0124138 |
| PRRG1 | ENST00000449135 | down | -0.8623585 | 0.0053866 |
| RUVBL2 | ENST00000601968 | down | -0.8624146 | 0.022281 |
| CEP85L | ENST00000368488 | down | -0.8624582 | 0.0150355 |
| FXYD5 | ENST00000423817 | down | -0.8628208 | 0.0397833 |
| LIAS | ENST00000424936 | down | -0.8630897 | 0.0104892 |
| CDHR4 | ENST00000412678 | down | -0.8635092 | 0.0195375 |
| VEGFA | ENST00000324450 | down | -0.863791 | 0.0315291 |
| ZNF706 | ENST00000520984 | down | -0.8640235 | 0.0035449 |
| SYNE2 | ENST00000344113 | down | -0.8640352 | 0.0216958 |
| NCOA6 | ENST00000612493 | down | -0.8642386 | 0.0308148 |
| ANXA4 | NM_001320698 | down | -0.8645999 | 0.0477338 |
| ZC3H6 | ENST00000409871 | down | -0.8648855 | 0.0162702 |
| CHPT1 | ENST00000229266 | down | -0.8650991 | 0.038567 |
| RASGRP4 | ENST00000293062 | down | -0.8654042 | 0.0205387 |
| ANAPC16 | ENST00000615507 | down | -0.8656564 | 0.0014994 |
| SRSF3 | ENST00000373715 | down | -0.8656921 | 0.0412943 |
| ST13 | ENST00000216218 | down | -0.8660331 | 0.0109094 |
| TAX1BP1 | ENST00000433216 | down | -0.8665635 | 0.0149241 |
| LOC100131107 | NM_001310142 | down | -0.8665903 | 0.048391 |
| EZH1 | ENST00000428826 | down | -0.8666108 | 0.0170202 |
| ERP29 | ENST00000261735 | down | -0.8670165 | 0.0055474 |
| RABL6 | ENST00000311502 | down | -0.8671598 | 0.020189 |
| EXOC1 | ENST00000349598 | down | -0.8675263 | 0.0212768 |
| DTD2 | ENST00000310850 | down | -0.8676359 | 0.0126752 |
| ARMCX6 | ENST00000538627 | down | -0.8678176 | 0.0470786 |
| PHB2 | NM_001267700 | down | -0.8681405 | 0.0125387 |
| CSNK2A1 | ENST00000217244 | down | -0.8682311 | 0.0223708 |
| ZBTB8OS | ENST00000468695 | down | -0.8683166 | 0.0284102 |
| CUL4B | ENST00000336592 | down | -0.8684834 | 0.0386696 |
| CH17-270A2.2 | ENST00000625258 | down | -0.8689207 | 0.0036635 |
| VAV1 | ENST00000602142 | down | -0.8690276 | 0.0026671 |
| KRT40 | ENST00000398486 | down | -0.8697021 | 0.0257136 |
| UHRF1BP1 | ENST00000192788 | down | -0.8697886 | 0.0332189 |
| TCEA3 | NM_003196 | down | -0.8698796 | 0.0175721 |
| CATG00000043113.1 | ENCT00000020445 | down | -0.8703433 | 0.0406668 |
| MRPL52 | ENST00000397505 | down | -0.8704339 | 0.0279811 |
| SRSF10 | NM_001300937 | down | -0.8704401 | 0.0177947 |
| C11orf70 | ENST00000534360 | down | -0.8705589 | 0.0117225 |
| PI4KB | ENST00000368874 | down | -0.8706896 | 0.0351203 |
| MEIS2 | ENST00000397620 | down | -0.8718423 | 0.0404215 |
| PSMB8 | ENST00000374882 | down | -0.8718991 | 0.0041493 |
| TSN | ENST00000389682 | down | -0.8728362 | 0.0377661 |
| ZNF286A | ENST00000464847 | down | -0.8728595 | 0.0349395 |
| MYO9A | ENST00000356056 | down | -0.8733991 | 0.0427313 |
| RBM7 | ENST00000624815 | down | -0.8735337 | 0.0259858 |
| HIST1H3B | ENST00000621411 | down | -0.8735511 | 0.0136832 |
| AK6 | ENST00000380818 | down | -0.8738515 | 0.0011433 |
| DHX36 | ENST00000329463 | down | -0.8739851 | 0.0320362 |
| FAM76A | ENST00000373954 | down | -0.8741483 | 0.0079103 |
| PCDH11X | ENST00000504220 | down | -0.8741647 | 0.0316745 |
| PBDC1 | ENST00000373357 | down | -0.8743216 | 0.0020707 |
| PSMA4 | ENST00000044462 | down | -0.8743686 | 0.0171763 |
| HIVEP1 | ENST00000379388 | down | -0.8743988 | 0.0203919 |
| SNRPD3 | NM_001278656 | down | -0.8747038 | 0.0169893 |
| RAB10 | ENST00000264710 | down | -0.8747595 | 7.634E-06 |
| TSC22D2 | ENST00000361875 | down | -0.8748495 | 0.0214252 |
| MRAS | ENST00000423968 | down | -0.8750218 | 0.0462081 |
| FDX1 | ENST00000260270 | down | -0.87532 | 0.0377668 |
| MTX2 | ENST00000452865 | down | -0.8753687 | 0.0109525 |
| RITA1 | ENST00000552495 | down | -0.8755163 | 0.0250409 |
| CD320 | ENST00000301458 | down | -0.8757234 | 0.0459263 |
| RPL32 | ENST00000396957 | down | -0.8757498 | 0.0366621 |
| NEK9 | ENST00000238616 | down | -0.8758396 | 0.0168046 |
| NUDC | ENST00000321265 | down | -0.8760433 | 0.042639 |
| ACSL3 | ENST00000392066 | down | -0.8761931 | 0.0242656 |
| RBMX2 | ENST00000305536 | down | -0.8772893 | 0.0158722 |
| CHCHD4 | ENST00000396914 | down | -0.8773184 | 0.0233971 |
| CCNT2 | ENST00000295238 | down | -0.87736 | 0.0284294 |
| PPARD | ENST00000360694 | down | -0.8779255 | 0.0420428 |
| SPRYD7 | ENST00000378195 | down | -0.8780247 | 0.0046432 |
| DCUN1D4 | ENST00000334635 | down | -0.8782242 | 0.0057792 |
| SLC31A1 | ENST00000374212 | down | -0.8786251 | 0.0007892 |
| FBF1 | ENST00000636174 | down | -0.8786849 | 0.0293911 |
| CUL2 | ENST00000374748 | down | -0.8787537 | 0.0050714 |
| SLC25A33 | ENST00000302692 | down | -0.8788061 | 0.0173312 |
| NOLC1 | ENST00000405356 | down | -0.8792705 | 0.0056252 |
| SIRT5 | ENST00000359782 | down | -0.8793617 | 0.0265427 |
| CRELD2 | ENST00000407217 | down | -0.8794552 | 0.021048 |
| RGR | ENST00000358110 | down | -0.8795835 | 0.0247302 |
| TRAPPC6B | ENST00000330149 | down | -0.8797669 | 0.0325356 |
| BBS9 | ENST00000355070 | down | -0.8803765 | 0.0429138 |
| NFKBIL1 | ENST00000376145 | down | -0.8803973 | 0.0196043 |
| BIN1 | ENST00000259238 | down | -0.8805471 | 0.0079925 |
| CEP70 | ENST00000464035 | down | -0.880649 | 0.0446782 |
| NMT2 | ENST00000378150 | down | -0.8808546 | 0.0331463 |
| HEXDC | ENST00000327949 | down | -0.8815908 | 0.0029889 |
| IFT22 | ENST00000315322 | down | -0.8817097 | 0.0036861 |
| SPHK1 | ENST00000545180 | down | -0.8817333 | 0.0373542 |
| RBM15 | ENST00000618772 | down | -0.8820097 | 0.0247276 |
| MFSD2B | ENST00000338315 | down | -0.8831892 | 0.0143071 |
| MRPL28 | ENST00000199706 | down | -0.8832103 | 0.0220096 |
| BCCIP | ENST00000368759 | down | -0.8832298 | 0.0303102 |
| PROSC | ENST00000328195 | down | -0.8834537 | 0.0064654 |
| PROSC | ENST00000328195 | down | -0.8834537 | 0.0064654 |
| ZNF195 | ENST00000620374 | down | -0.8838558 | 0.0117658 |
| IL1RAP | ENST00000422940 | down | -0.8838756 | 0.0018311 |
| ANGPTL2 | ENST00000373425 | down | -0.8840935 | 0.0238563 |
| POLR2B | ENST00000639658 | down | -0.8842961 | 0.0416044 |
| RPIA | ENST00000283646 | down | -0.8843588 | 0.0461228 |
| ZSWIM7 | ENST00000399277 | down | -0.8845241 | 0.0017394 |
| NFX1 | ENST00000318524 | down | -0.8845946 | 0.0498762 |
| CEBPZOS | ENST00000402297 | down | -0.8846976 | 0.014521 |
| IGSF9 | ENST00000368094 | down | -0.8850356 | 0.0389071 |
| MLST8 | ENST00000569417 | down | -0.8851225 | 0.0137876 |
| COX19 | ENST00000344111 | down | -0.885447 | 0.0029911 |
| MT1A | ENST00000290705 | down | -0.8855263 | 0.0039787 |
| SSX2IP | ENST00000342203 | down | -0.8855876 | 0.0085871 |
| DDX60 | ENST00000393743 | down | -0.8856025 | 0.0266612 |
| CAMK2D | ENST00000508738 | down | -0.885637 | 0.0259344 |
| MPC2 | NM_015415 | down | -0.885927 | 0.0413821 |
| SOCS7 | ENST00000612932 | down | -0.8862573 | 0.0361616 |
| KAT7 | ENST00000424009 | down | -0.8864644 | 0.0379478 |
| ZDBF2 | NM_001285549 | down | -0.8865149 | 0.0266949 |
| EXOSC1 | ENST00000370885 | down | -0.8867755 | 5.906E-05 |
| ANXA7 | NM_001320880 | down | -0.8869228 | 0.0176763 |
| SUGP2 | ENST00000337018 | down | -0.8870902 | 0.0288949 |
| CCDC22 | ENST00000376227 | down | -0.8871829 | 0.0369772 |
| CEP41 | ENST00000541543 | down | -0.8874855 | 0.0179977 |
| PTRH2 | ENST00000470557 | down | -0.8876005 | 0.025968 |
| PXK | ENST00000479241 | down | -0.8876325 | 0.0003337 |
| FAM162A | ENST00000477892 | down | -0.8877518 | 0.0056176 |
| FAM76A | ENST00000419687 | down | -0.8878651 | 0.039306 |
| C12orf43 | ENST00000538296 | down | -0.8879452 | 0.0070679 |
| TMEM116 | ENST00000550831 | down | -0.8883244 | 0.0180283 |
| ZNF557 | ENST00000414706 | down | -0.8884695 | 0.034039 |
| ANXA7 | ENST00000372919 | down | -0.8886553 | 0.0359806 |
| TSEN2 | ENST00000444864 | down | -0.8889323 | 0.0069496 |
| GTF2H1 | ENST00000265963 | down | -0.8892082 | 0.0366549 |
| TMEM230 | ENST00000379283 | down | -0.8892267 | 0.009361 |
| UBE2K | ENST00000510934 | down | -0.890662 | 0.0005511 |
| RAB21 | ENST00000261263 | down | -0.8907511 | 0.020184 |
| IFT22 | ENST00000437644 | down | -0.890758 | 0.0404149 |
| CANX | ENST00000452673 | down | -0.8910618 | 0.0067259 |
| TSC2 | ENST00000642936 | down | -0.891651 | 0.0371512 |
| NDUFA8 | NM_001318195 | down | -0.8917675 | 0.00401 |
| C2orf69 | ENST00000319974 | down | -0.8923421 | 0.0244095 |
| RUFY1 | ENST00000437570 | down | -0.8926417 | 0.0165037 |
| C2CD5 | ENST00000536386 | down | -0.8926937 | 0.0080872 |
| RANBP6 | ENST00000259569 | down | -0.8927272 | 0.0032207 |
| TM2D2 | NM_001024380 | down | -0.8928124 | 0.0330109 |
| EYA3 | ENST00000373871 | down | -0.8932365 | 0.0442018 |
| ZDHHC4 | ENST00000396713 | down | -0.8932706 | 0.007842 |
| RPL28 | ENST00000560583 | down | -0.89332 | 0.0022582 |
| SET | ENST00000322030 | down | -0.8936819 | 0.0289309 |
| MRPS35 | ENST00000538315 | down | -0.8937514 | 0.0060176 |
| GSR | ENST00000537535 | down | -0.8940413 | 0.0124636 |
| TRAPPC4 | ENST00000434101 | down | -0.8944116 | 0.0428746 |
| CHRDL1 | ENST00000482160 | down | -0.894557 | 0.0268465 |
| PHB2 | ENST00000440277 | down | -0.8946899 | 0.0141278 |
| ATP5J | ENST00000400087 | down | -0.8948666 | 0.0038977 |
| FAM76B | ENST00000358780 | down | -0.8951283 | 0.0440556 |
| CHCHD7 | ENST00000523975 | down | -0.8956001 | 0.0299721 |
| STAU2 | ENST00000521210 | down | -0.8957243 | 0.0183632 |
| CACNA1C | ENST00000399634 | down | -0.8957491 | 0.010429 |
| TCF12 | ENST00000557843 | down | -0.8962776 | 0.0341559 |
| RPL19 | ENST00000579260 | down | -0.8963202 | 0.0276713 |
| TMEM159 | NM_001301773 | down | -0.896598 | 0.0156656 |
| RBAK-RBAKDN | ENST00000407184 | down | -0.896965 | 0.0396981 |
| SFSWAP | ENST00000541286 | down | -0.8970445 | 0.0219592 |
| RBM17 | ENST00000446108 | down | -0.8973723 | 0.002411 |
| ZNF419 | ENST00000415379 | down | -0.8977134 | 0.0209052 |
| NFATC2 | ENST00000396009 | down | -0.8977175 | 0.0456083 |
| AC026740.1 | ENST00000594226 | down | -0.8978099 | 0.0484473 |
| PRELID2 | NM_205846 | down | -0.8982257 | 0.0443347 |
| SPG11 | ENST00000535302 | down | -0.8984457 | 0.0236315 |
| TCF12 | ENST00000333725 | down | -0.8988034 | 0.0409705 |
| TAMM41 | ENST00000455809 | down | -0.8989972 | 0.0339964 |
| ZNF138 | ENST00000440155 | down | -0.8990935 | 0.0471148 |
| ZNF227 | ENST00000589005 | down | -0.8991748 | 0.0432563 |
| PRKCSH | NM_002743 | down | -0.8993061 | 0.0192394 |
| SLC16A1 | ENST00000538576 | down | -0.8995225 | 0.0262797 |
| AP4S1 | ENST00000622409 | down | -0.8997531 | 0.0175726 |
| DDRGK1 | ENST00000354488 | down | -0.8999117 | 0.0138644 |
| LZTS2 | NM_001318101 | down | -0.8999355 | 0.0435769 |
| SEMA6C | ENST00000341697 | down | -0.9000811 | 0.0218856 |
| NOS3 | ENST00000297494 | down | -0.9007739 | 0.0300612 |
| CDC40 | ENST00000307731 | down | -0.9011766 | 0.0354173 |
| H1FNT | ENST00000335017 | down | -0.9019539 | 0.0250618 |
| ZNF524 | ENST00000301073 | down | -0.9022759 | 0.0077969 |
| MRPL21 | ENST00000362034 | down | -0.902576 | 0.0452306 |
| TRIM39-RPP21 | ENST00000623385 | down | -0.9026752 | 0.0118883 |
| NSMAF | ENST00000038176 | down | -0.9030128 | 0.0027518 |
| MTMR12 | ENST00000382142 | down | -0.9036105 | 0.0338582 |
| GPATCH11 | ENST00000281932 | down | -0.9040155 | 0.0063207 |
| GBA | ENST00000427500 | down | -0.9040321 | 0.0107925 |
| PHF20L1 | ENST00000220847 | down | -0.9040497 | 0.0397767 |
| EIF3K | ENST00000592558 | down | -0.904271 | 0.0392607 |
| TIMM23B | NM_001290117 | down | -0.9046597 | 0.0050723 |
| MIF4GD | ENST00000577542 | down | -0.9048168 | 0.0006915 |
| NEURL1 | ENST00000369780 | down | -0.9049294 | 0.0362672 |
| MAPK8IP3 | ENST00000356010 | down | -0.9050506 | 0.0121028 |
| ECHDC1 | ENST00000454859 | down | -0.905265 | 0.008024 |
| RP11-152F13.10 | ENST00000562833 | down | -0.9060283 | 0.0027864 |
| FPR2 | ENST00000340023 | down | -0.9060564 | 0.0234409 |
| RPP38 | ENST00000378203 | down | -0.9061646 | 0.0023595 |
| CNNM2 | ENST00000369875 | down | -0.9062312 | 0.0267844 |
| UBAP1L | ENST00000502113 | down | -0.9064692 | 0.0056571 |
| UROS | ENST00000368797 | down | -0.9064905 | 0.0005974 |
| KLLN | ENST00000445946 | down | -0.9065471 | 0.0460685 |
| CISD3 | ENST00000613478 | down | -0.9065739 | 0.0421952 |
| ZNF208 | ENST00000601773 | down | -0.9070699 | 0.0181646 |
| C18orf25 | ENST00000619301 | down | -0.9071414 | 0.0011466 |
| C2orf49 | ENST00000410049 | down | -0.9075049 | 0.0005964 |
| BCLAF1 | ENST00000530767 | down | -0.9078708 | 0.0179487 |
| ZFAND6 | ENST00000616533 | down | -0.9081842 | 0.0421886 |
| GATAD1 | ENST00000287957 | down | -0.9083193 | 0.0111726 |
| SPTBN4 | ENST00000392023 | down | -0.9085018 | 0.0043548 |
| ZNF32 | ENST00000395797 | down | -0.9085148 | 0.0300091 |
| MYH10 | ENST00000379980 | down | -0.9086027 | 0.0445203 |
| ART5 | ENST00000397068 | down | -0.9088025 | 0.0004934 |
| KDM8 | ENST00000286096 | down | -0.9096746 | 0.0192478 |
| TMEM88 | ENST00000301599 | down | -0.9097621 | 0.0457172 |
| KATNA1 | ENST00000367411 | down | -0.9098068 | 0.0052922 |
| TMEM205 | ENST00000593256 | down | -0.9101383 | 0.0020974 |
| MARK3 | ENST00000216288 | down | -0.9103975 | 0.0253713 |
| MDM2 | ENST00000258148 | down | -0.9106123 | 0.016844 |
| LINC00493 | ENST00000411646 | down | -0.9108861 | 0.0040653 |
| RPL26 | ENST00000293842 | down | -0.9112862 | 0.0076729 |
| LRRC23 | ENST00000323702 | down | -0.9117692 | 0.0233311 |
| SLC25A1 | ENST00000451283 | down | -0.9119586 | 0.0060472 |
| AIFM3 | ENST00000405089 | down | -0.9127481 | 0.0162345 |
| FANCF | ENST00000327470 | down | -0.9137562 | 0.0389189 |
| PRR13 | ENST00000549581 | down | -0.9139041 | 0.035305 |
| AMFR | NM_001323512 | down | -0.9140763 | 0.0085503 |
| LILRB5 | ENST00000345866 | down | -0.9140798 | 0.0250987 |
| SMC5 | ENST00000361138 | down | -0.9141937 | 0.0128834 |
| STXBP1 | ENST00000373302 | down | -0.9141987 | 0.0049471 |
| RPS5 | ENST00000196551 | down | -0.9144598 | 0.0053502 |
| GTPBP6 | ENST00000326153 | down | -0.9148275 | 0.0006287 |
| MED30 | ENST00000297347 | down | -0.9150232 | 0.0106868 |
| MRAS | ENST00000464896 | down | -0.9150512 | 0.0262737 |
| SNRPC | ENST00000244520 | down | -0.915177 | 0.0212432 |
| TMEM147 | ENST00000392204 | down | -0.9153749 | 0.0228121 |
| NOP10 | ENST00000328848 | down | -0.915613 | 0.0067487 |
| CATG00000052127.1 | HBMT00000827657 | down | -0.9157837 | 0.0077885 |
| ILVBL | ENST00000263383 | down | -0.9159027 | 0.0020713 |
| PRPF4B | ENST00000337659 | down | -0.9159221 | 0.0258076 |
| PSIP1 | ENST00000397519 | down | -0.9167762 | 0.0277147 |
| ALDH3B2 | ENST00000530069 | down | -0.9168906 | 0.046894 |
| MAP3K7 | ENST00000369325 | down | -0.9169266 | 0.0418126 |
| C22orf39 | ENST00000542103 | down | -0.9170761 | 0.0219654 |
| CEBPG | ENST00000284000 | down | -0.9172529 | 0.0101478 |
| AARS | ENST00000261772 | down | -0.9174238 | 0.0243363 |
| ARMC7 | ENST00000582136 | down | -0.9174438 | 0.0427303 |
| ULK3 | ENST00000440863 | down | -0.9175 | 0.026464 |
| THUMPD1 | ENST00000381337 | down | -0.9175532 | 0.0182174 |
| PSMB7 | ENST00000259457 | down | -0.9176511 | 0.0007671 |
| CNN2 | ENST00000565096 | down | -0.9176726 | 0.0102556 |
| WSB1 | ENST00000348811 | down | -0.9180778 | 0.0031519 |
| ABCC3 | ENST00000427699 | down | -0.9184859 | 0.0040192 |
| PABPC1L | ENST00000217073 | down | -0.918564 | 0.0360289 |
| NFKBIB | ENST00000313582 | down | -0.9188341 | 0.0175981 |
| RUFY2 | ENST00000399200 | down | -0.9192375 | 0.0080493 |
| ADAMTSL4 | ENST00000369041 | down | -0.9194767 | 0.0100671 |
| UTP18 | ENST00000225298 | down | -0.9199002 | 0.0026904 |
| BIN1 | ENST00000346226 | down | -0.9200974 | 0.0476163 |
| KRIT1 | NM_001350681 | down | -0.9200988 | 0.0063737 |
| DCPS | ENST00000263579 | down | -0.9203677 | 0.0116295 |
| KY | ENST00000423778 | down | -0.9206067 | 0.0297908 |
| CCBL2 | ENST00000260508 | down | -0.9212387 | 0.0032562 |
| SERINC3 | ENST00000255175 | down | -0.9212757 | 0.0233485 |
| TTC37 | ENST00000358746 | down | -0.9214566 | 0.0298839 |
| VAMP2 | ENST00000316509 | down | -0.921602 | 0.0073259 |
| ETV2 | ENST00000379026 | down | -0.9216114 | 0.0136879 |
| AMMECR1L | ENST00000272647 | down | -0.9217532 | 0.0166195 |
| HYAL2 | ENST00000442581 | down | -0.9220305 | 0.008881 |
| SCN1A | ENST00000641575 | down | -0.9223022 | 0.0260517 |
| PSIP1 | ENST00000380716 | down | -0.9225269 | 0.0024523 |
| RPL10A | ENST00000322203 | down | -0.9225966 | 1.311E-05 |
| GTF3C3 | ENST00000409364 | down | -0.9228062 | 0.0225923 |
| MTO1 | ENST00000370300 | down | -0.9228976 | 0.0232895 |
| UBE2M | ENST00000253023 | down | -0.9232678 | 0.0090191 |
| SLC25A25 | ENST00000373064 | down | -0.9238726 | 0.0232458 |
| WASHC2C | ENST00000336378 | down | -0.923978 | 0.0215371 |
| CYB5B | ENST00000307892 | down | -0.9241371 | 0.0035961 |
| POLR2M | ENST00000380557 | down | -0.9241882 | 0.0446184 |
| CHCHD4 | ENST00000295767 | down | -0.9244145 | 0.0166516 |
| BRE | ENST00000379632 | down | -0.9245904 | 0.0041894 |
| TRIM45 | ENST00000256649 | down | -0.9246455 | 0.0432791 |
| AMH | ENST00000221496 | down | -0.9246658 | 0.0250991 |
| WBP4 | ENST00000379487 | down | -0.9247264 | 0.0003498 |
| MTRNR2L1 | ENST00000540040 | down | -0.9253692 | 0.0470243 |
| TRAPPC2B | ENST00000596755 | down | -0.92572 | 0.0125618 |
| CTSL | ENST00000343150 | down | -0.9260097 | 0.0169537 |
| TAF1 | ENST00000276072 | down | -0.9261019 | 0.0180177 |
| RAD50 | ENST00000378823 | down | -0.9263564 | 0.046305 |
| WASF1 | ENST00000392588 | down | -0.9264667 | 0.0307587 |
| TBC1D20 | ENST00000354200 | down | -0.9264814 | 0.0052792 |
| MTHFD1L | ENST00000367307 | down | -0.9265541 | 0.0436012 |
| TFIP11 | ENST00000405938 | down | -0.926966 | 0.0054462 |
| APP | ENST00000348990 | down | -0.9271443 | 0.0373501 |
| ZFAND6 | ENST00000558494 | down | -0.9271678 | 0.004389 |
| PPIL1 | ENST00000373699 | down | -0.9278106 | 0.0106312 |
| COX7B | ENST00000481445 | down | -0.9278158 | 0.0062187 |
| C15orf41 | ENST00000566621 | down | -0.9282636 | 0.0093693 |
| RAB4A | ENST00000366690 | down | -0.9288866 | 0.0019158 |
| C6orf163 | ENST00000608326 | down | -0.928933 | 0.0001474 |
| UBE2D1 | ENST00000373910 | down | -0.928963 | 0.0378145 |
| SIGMAR1 | NM_001282205 | down | -0.9290689 | 0.0149715 |
| RELL2 | ENST00000297164 | down | -0.9291976 | 0.0193741 |
| MLLT10 | ENST00000377100 | down | -0.9294778 | 0.0176371 |
| MON1B | ENST00000545553 | down | -0.9296084 | 0.0129389 |
| RP11-864I4.1 | uc010rlw.1 | down | -0.9301808 | 0.0151885 |
| DLD | ENST00000205402 | down | -0.931327 | 0.0003078 |
| TRIM37 | NM_001353084 | down | -0.9314088 | 0.0424326 |
| SLC25A1 | ENST00000215882 | down | -0.9316334 | 0.0199646 |
| ZNF717 | ENST00000468296 | down | -0.931758 | 0.0417463 |
| ASPH | ENST00000379454 | down | -0.9318466 | 0.0275293 |
| THAP1 | ENST00000254250 | down | -0.9320789 | 0.0230388 |
| KDM3A | ENST00000409556 | down | -0.9329672 | 0.0188966 |
| C1orf54 | ENST00000369102 | down | -0.9331833 | 0.0152768 |
| SRP68 | ENST00000602720 | down | -0.9332105 | 0.0197545 |
| PPIE | ENST00000356511 | down | -0.9332213 | 0.0134557 |
| CYTIP | ENST00000264192 | down | -0.9335893 | 0.0001149 |
| JMJD1C | NM_001318153 | down | -0.9343743 | 0.0223479 |
| FOPNL | ENST00000575073 | down | -0.9350639 | 0.0105782 |
| KRT10 | ENST00000269576 | down | -0.9355257 | 0.0335262 |
| PACRGL | ENST00000503585 | down | -0.9358945 | 0.0369193 |
| PPP2CA | ENST00000481195 | down | -0.9369638 | 0.0475331 |
| COX4I1 | ENST00000562336 | down | -0.9373408 | 0.0244108 |
| MYNN | ENST00000544106 | down | -0.9373427 | 0.0358101 |
| OXR1 | ENST00000449762 | down | -0.9375873 | 0.0344985 |
| SMAD5 | ENST00000545620 | down | -0.9383255 | 0.003502 |
| MED10 | ENST00000255764 | down | -0.9383792 | 0.0262443 |
| MRPS9 | ENST00000258455 | down | -0.9383888 | 0.0366279 |
| AIFM2 | ENST00000613322 | down | -0.9387869 | 0.0148542 |
| PIGC | ENST00000344529 | down | -0.9390346 | 0.0211902 |
| FHOD3 | ENST00000590592 | down | -0.939049 | 0.0106619 |
| RAB4A | ENST00000618010 | down | -0.9393585 | 0.0026808 |
| RUFY3 | ENST00000417478 | down | -0.9393625 | 0.0037833 |
| SCN2A | ENST00000636071 | down | -0.9394969 | 0.0031896 |
| KDM6B | ENST00000254846 | down | -0.9395378 | 0.0172502 |
| FNIP1 | ENST00000307954 | down | -0.9395902 | 0.0428787 |
| DAP3 | ENST00000535183 | down | -0.9404265 | 0.0192996 |
| ACSL3 | NM_001354158 | down | -0.9406263 | 0.0214299 |
| PLSCR1 | ENST00000342435 | down | -0.9407894 | 0.0050748 |
| PCBP2 | ENST00000548933 | down | -0.9416997 | 0.015576 |
| ELOF1 | ENST00000252445 | down | -0.9420654 | 0.0050096 |
| TM2D2 | ENST00000456397 | down | -0.9422941 | 0.0414732 |
| SDHD | ENST00000375549 | down | -0.9432972 | 0.0148832 |
| ADA | ENST00000537820 | down | -0.9437715 | 0.0050878 |
| PPP1R8 | ENST00000236412 | down | -0.9437885 | 0.0214773 |
| SNRPD1 | ENST00000300413 | down | -0.944481 | 0.0158198 |
| LARP1B | ENST00000512292 | down | -0.9446457 | 0.0088297 |
| THOC6 | ENST00000326266 | down | -0.9447059 | 0.0255616 |
| TMED4 | NM_001303062 | down | -0.9449239 | 0.0160127 |
| POLR2B | ENST00000314595 | down | -0.9455627 | 0.0173202 |
| KIF3A | ENST00000403231 | down | -0.9458448 | 0.0045415 |
| PHF1 | ENST00000374512 | down | -0.9461245 | 0.0007978 |
| C11orf73 | ENST00000533986 | down | -0.9461841 | 0.0227943 |
| PRMT5 | ENST00000553897 | down | -0.9463324 | 0.0268672 |
| CTD-2207O23.3 | ENST00000617428 | down | -0.9464396 | 0.0177988 |
| MRPS18C | ENST00000507349 | down | -0.9464524 | 0.0028698 |
| ZBTB20 | NM_001348801 | down | -0.9467349 | 0.0392032 |
| TRIM37 | ENST00000262294 | down | -0.9467772 | 0.0217522 |
| FAM161A | ENST00000405894 | down | -0.9471545 | 0.0117883 |
| ZNF32 | ENST00000374433 | down | -0.9473547 | 0.0048311 |
| ASPH | ENST00000517847 | down | -0.9479175 | 0.0039757 |
| C5orf63 | ENST00000535381 | down | -0.9485492 | 0.0494058 |
| FXYD6 | ENST00000539526 | down | -0.9488623 | 0.0178309 |
| FAM220A | ENST00000313324 | down | -0.9493056 | 0.0048482 |
| LTV1 | uc003qju.1 | down | -0.9493525 | 0.0334601 |
| SHISA8 | ENST00000621082 | down | -0.9495115 | 0.036985 |
| SPG11 | ENST00000261866 | down | -0.9495924 | 0.0166525 |
| GGCT | ENST00000409144 | down | -0.9496346 | 0.0411302 |
| NETO2 | ENST00000562435 | down | -0.9498057 | 0.0298088 |
| OBSCN | ENST00000422127 | down | -0.9499917 | 0.0464245 |
| ULK2 | ENST00000361658 | down | -0.9500944 | 0.0387347 |
| NFE2L2 | NM_001313904 | down | -0.9501413 | 0.0356727 |
| NANS | ENST00000427646 | down | -0.9506304 | 0.0463783 |
| PCDHGB5 | ENST00000617380 | down | -0.9509268 | 0.012764 |
| RPL13 | ENST00000311528 | down | -0.950955 | 0.0274546 |
| ZNF268 | ENST00000541211 | down | -0.9509789 | 0.0079545 |
| ATP1B1 | ENST00000367816 | down | -0.9513403 | 0.0492081 |
| ZNF765 | ENST00000594030 | down | -0.9513951 | 0.0375415 |
| NOP16 | ENST00000614830 | down | -0.9518934 | 0.0004169 |
| SUGCT | ENST00000335693 | down | -0.9521306 | 0.0491735 |
| NAE1 | ENST00000290810 | down | -0.9521915 | 0.0320265 |
| IRF3 | ENST00000599223 | down | -0.9527346 | 0.0363223 |
| RAB40C | ENST00000248139 | down | -0.952781 | 0.0479546 |
| FCER1G | uc001fza.1 | down | -0.9531697 | 0.0078677 |
| PRDM5 | ENST00000264808 | down | -0.9532884 | 0.0373951 |
| NFIL3 | ENST00000297689 | down | -0.9537424 | 0.006368 |
| SLC43A3 | ENST00000352187 | down | -0.9539505 | 0.0288318 |
| C15orf40 | ENST00000451195 | down | -0.9539846 | 0.0176626 |
| ARL8A | ENST00000272217 | down | -0.9539922 | 0.033187 |
| ZCCHC4 | ENST00000302874 | down | -0.9542311 | 0.0128478 |
| FHL2 | ENST00000408995 | down | -0.9546206 | 0.013045 |
| NDUFV3 | ENST00000340344 | down | -0.9546276 | 0.0042333 |
| DPP9 | ENST00000594671 | down | -0.9549878 | 0.0141393 |
| TXNRD1 | ENST00000526691 | down | -0.9554778 | 0.0308224 |
| PIGF | ENST00000306465 | down | -0.9557992 | 9.911E-06 |
| ZKSCAN1 | ENST00000535170 | down | -0.9558044 | 0.0254049 |
| RAB20 | ENST00000267328 | down | -0.9558981 | 0.0225913 |
| TRIM22 | ENST00000379965 | down | -0.9567225 | 0.0447843 |
| SNRPC | ENST00000374018 | down | -0.9571459 | 0.0451905 |
| COMMD10 | ENST00000274458 | down | -0.9572561 | 0.0171891 |
| EOGT | ENST00000383701 | down | -0.9573676 | 0.0063754 |
| SEPHS1 | uc021pnd.1 | down | -0.9578941 | 0.0362825 |
| MLLT10 | ENST00000307729 | down | -0.9584623 | 0.0304897 |
| DIRC2 | ENST00000261038 | down | -0.9585766 | 0.0273457 |
| USF2 | ENST00000222305 | down | -0.958952 | 0.0065448 |
| USF2 | ENST00000222305 | down | -0.958952 | 0.0065448 |
| ANKRD6 | ENST00000339746 | down | -0.9591169 | 0.0338526 |
| PTRH2 | ENST00000393038 | down | -0.959275 | 0.0090992 |
| MLLT10 | NM_001324297 | down | -0.9594117 | 0.014407 |
| TGIF1 | ENST00000330513 | down | -0.9599671 | 0.0237861 |
| DPH2 | ENST00000255108 | down | -0.9600293 | 0.0059229 |
| RP13-122B23.13 | ENST00000645271 | down | -0.9603085 | 0.0353472 |
| FAM173B | ENST00000511437 | down | -0.9604065 | 0.0312574 |
| NUBP1 | ENST00000433392 | down | -0.9606205 | 0.0023185 |
| AL353354.1 | ENST00000321830 | down | -0.9609258 | 0.0033365 |
| UTP23 | ENST00000309822 | down | -0.9611251 | 0.0256194 |
| PPP1R21 | ENST00000449090 | down | -0.9614247 | 0.008075 |
| C18orf8 | ENST00000615148 | down | -0.9615858 | 0.0015988 |
| CATG00000001363.1 | ENCT00000063598 | down | -0.961608 | 0.0084923 |
| JUND | ENST00000252818 | down | -0.9625177 | 0.0267581 |
| SEC31B | uc001kre.1 | down | -0.9627281 | 0.0190013 |
| LCA5 | ENST00000369846 | down | -0.962928 | 0.0045855 |
| ZNF33A | ENST00000374618 | down | -0.9632454 | 0.0050013 |
| CHCHD10 | ENST00000520222 | down | -0.9632932 | 0.0212121 |
| CSTF2 | NM_001306209 | down | -0.9637001 | 0.0367223 |
| RPP25 | ENST00000322177 | down | -0.963793 | 0.013688 |
| VTA1 | ENST00000620996 | down | -0.9638918 | 0.006193 |
| FUCA2 | ENST00000002165 | down | -0.9640557 | 0.0001729 |
| NFE2L2 | ENST00000446151 | down | -0.9641944 | 0.0492659 |
| ECI1 | ENST00000562238 | down | -0.9643255 | 0.0036148 |
| ECI1 | ENST00000562238 | down | -0.9643255 | 0.0036148 |
| NTMT1 | ENST00000482347 | down | -0.9645316 | 0.0013794 |
| SLC30A9 | ENST00000264451 | down | -0.9645917 | 0.0323445 |
| TIPRL | ENST00000367833 | down | -0.9646678 | 0.000179 |
| LCNL1 | ENST00000408973 | down | -0.9652882 | 0.0236367 |
| ZCRB1 | ENST00000266529 | down | -0.9653522 | 0.0015572 |
| TAF12 | ENST00000263974 | down | -0.9658101 | 0.0008643 |
| PPWD1 | ENST00000535264 | down | -0.9667245 | 0.0189329 |
| PTDSS2 | ENST00000308020 | down | -0.9672963 | 0.008713 |
| RPP38 | ENST00000616640 | down | -0.9674459 | 0.025881 |
| AJUBA | ENST00000262713 | down | -0.9676448 | 0.0011552 |
| PCID2 | ENST00000375479 | down | -0.9677544 | 0.0040456 |
| EIF4E | ENST00000504432 | down | -0.9689546 | 0.0122419 |
| GSTZ1 | ENST00000349555 | down | -0.9697463 | 0.0171804 |
| CANX | ENST00000504734 | down | -0.9697765 | 0.0227764 |
| RABIF | ENST00000367262 | down | -0.9697792 | 0.0070075 |
| MFF | NM_020194 | down | -0.9703025 | 0.0273495 |
| VPS13C | ENST00000395898 | down | -0.9705289 | 0.0425862 |
| PBRM1 | ENST00000356770 | down | -0.9705858 | 0.0275785 |
| ACSL3 | ENST00000357430 | down | -0.9718796 | 0.0149436 |
| ACSL3 | ENST00000357430 | down | -0.9718796 | 0.0149436 |
| TENM3 | ENST00000511685 | down | -0.9719654 | 0.0235978 |
| FOXC1 | ENST00000380874 | down | -0.9721579 | 0.0070489 |
| CCDC40 | ENST00000269318 | down | -0.9724483 | 0.0243049 |
| PRPF18 | ENST00000378572 | down | -0.9727624 | 0.0064723 |
| RWDD3 | ENST00000370202 | down | -0.9728431 | 0.0461954 |
| ITFG1 | ENST00000544001 | down | -0.9729509 | 0.0124196 |
| CCNH | ENST00000508855 | down | -0.9731863 | 0.0016984 |
| CTSL | ENST00000340342 | down | -0.9738248 | 0.048796 |
| LARP7 | ENST00000324052 | down | -0.9745497 | 0.0287394 |
| MTERF3 | ENST00000287025 | down | -0.9748319 | 0.0345875 |
| DENR | ENST00000280557 | down | -0.9752378 | 0.0044516 |
| PCID2 | ENST00000246505 | down | -0.9752727 | 0.0103723 |
| RTN4IP1 | ENST00000369063 | down | -0.9754681 | 0.009066 |
| SIRT1 | ENST00000432464 | down | -0.9755676 | 0.031385 |
| SESN3 | ENST00000278499 | down | -0.9762323 | 0.0054599 |
| CEP19 | ENST00000409690 | down | -0.9764366 | 0.0104637 |
| XRN2 | ENST00000377191 | down | -0.97662 | 0.025904 |
| RRP36 | ENST00000244496 | down | -0.9767048 | 0.026262 |
| NUFIP1 | ENST00000379161 | down | -0.976719 | 0.0488263 |
| LIMS4 | ENST00000632897 | down | -0.9768654 | 0.0329719 |
| NPM2 | ENST00000518119 | down | -0.9770143 | 0.0290707 |
| PCCA | NM_001352605 | down | -0.9773072 | 0.0294704 |
| DYM | NM_001353211 | down | -0.9776303 | 0.0474183 |
| RBM39 | ENST00000528062 | down | -0.9777461 | 0.0026683 |
| PCSK2 | ENST00000377899 | down | -0.9778461 | 0.0165285 |
| VEGFA | ENST00000457104 | down | -0.9780504 | 0.0172439 |
| MED30 | ENST00000522839 | down | -0.9780555 | 0.0320185 |
| PNISR | ENST00000369239 | down | -0.978076 | 0.021769 |
| INAFM2 | ENST00000638170 | down | -0.9781512 | 0.0391337 |
| TAF6L | ENST00000294168 | down | -0.9784816 | 0.020035 |
| ISOC1 | ENST00000173527 | down | -0.9792024 | 0.0085379 |
| CREB1 | ENST00000432329 | down | -0.9793121 | 0.0134398 |
| PHYKPL | ENST00000308158 | down | -0.9793446 | 0.0066652 |
| METTL15 | ENST00000406787 | down | -0.979395 | 0.0010563 |
| TRMT10B | ENST00000377753 | down | -0.979879 | 0.0097369 |
| GCLM | ENST00000615724 | down | -0.9801908 | 0.0255724 |
| RSL24D1 | ENST00000260443 | down | -0.9802433 | 0.0035218 |
| GCH1 | ENST00000543643 | down | -0.9803175 | 0.0073548 |
| FAU | ENST00000529259 | down | -0.9805837 | 0.0237052 |
| PPDPF | ENST00000370179 | down | -0.9809086 | 0.0043937 |
| INTS12 | ENST00000394735 | down | -0.9812807 | 0.0446687 |
| FAHD1 | ENST00000382666 | down | -0.9813815 | 0.0263868 |
| ECHDC1 | ENST00000454591 | down | -0.9816241 | 0.0113289 |
| OBSCN | ENST00000570156 | down | -0.9816556 | 0.0273106 |
| CDC40 | ENST00000368932 | down | -0.9817469 | 0.0140935 |
| C11orf74 | ENST00000347206 | down | -0.9825005 | 0.0022295 |
| MDM2 | ENST00000258149 | down | -0.9827612 | 0.0440027 |
| MDM2 | ENST00000258149 | down | -0.9827612 | 0.0440027 |
| TFDP2 | ENST00000495310 | down | -0.9831338 | 0.0076028 |
| RCHY1 | ENST00000513257 | down | -0.9831576 | 0.0178546 |
| CREM | ENST00000337656 | down | -0.9833937 | 0.0025842 |
| C18orf8 | ENST00000590868 | down | -0.9835289 | 0.0429123 |
| ZNF268 | ENST00000539248 | down | -0.9837813 | 0.0389544 |
| EXOSC1 | ENST00000370884 | down | -0.9843011 | 0.0028504 |
| ACSL1 | ENST00000507295 | down | -0.9843838 | 0.0227352 |
| PLGRKT | ENST00000223864 | down | -0.9844654 | 0.0169519 |
| NDUFA10 | ENST00000620965 | down | -0.9851235 | 0.0492422 |
| ACOT13 | ENST00000230048 | down | -0.9851841 | 0.0010394 |
| FGFR1OP2 | ENST00000229395 | down | -0.9852586 | 0.0105965 |
| DNAJB9 | ENST00000249356 | down | -0.9854339 | 0.0085434 |
| TRPV4 | ENST00000261740 | down | -0.9854621 | 0.0474808 |
| UROS | NM_001324038 | down | -0.9854999 | 0.0213099 |
| ACAP2 | ENST00000326793 | down | -0.9855415 | 0.0088827 |
| MRPL37 | ENST00000360840 | down | -0.985751 | 0.0003519 |
| POLR3E | ENST00000299853 | down | -0.9857553 | 0.0464707 |
| RTN4 | ENST00000337526 | down | -0.9861067 | 0.0435361 |
| USP25 | ENST00000400183 | down | -0.9862879 | 0.023594 |
| NUBP1 | ENST00000283027 | down | -0.9867646 | 0.0049524 |
| LIAS | ENST00000381846 | down | -0.9871975 | 0.0299891 |
| SMIM19 | ENST00000438528 | down | -0.9878412 | 0.0467366 |
| TMEM185B | ENST00000426077 | down | -0.9878569 | 0.0368002 |
| BLOC1S2 | NM_001282437 | down | -0.9882759 | 0.0466821 |
| RPL19 | ENST00000225430 | down | -0.9884113 | 0.0055436 |
| PRB1 | ENST00000545626 | down | -0.9886674 | 0.0367794 |
| MANBA | ENST00000644159 | down | -0.9887764 | 0.016147 |
| ZNF195 | ENST00000354599 | down | -0.9887871 | 0.0460741 |
| AGTR1 | ENST00000349243 | down | -0.9896296 | 0.0370186 |
| MAPK13 | ENST00000211287 | down | -0.9900999 | 0.0129982 |
| PCNP | ENST00000469941 | down | -0.9902354 | 0.0390668 |
| MPC2 | ENST00000367846 | down | -0.9911371 | 0.0026528 |
| GTF2IRD1 | ENST00000476977 | down | -0.9911997 | 0.0101868 |
| DNAJC1 | ENST00000376980 | down | -0.9914098 | 0.0309927 |
| MFSD2B | ENST00000406420 | down | -0.9918005 | 0.0200735 |
| TIMM10B | ENST00000254616 | down | -0.9924909 | 0.0041466 |
| RAB22A | ENST00000244040 | down | -0.9926532 | 0.0057105 |
| MYH10 | ENST00000269243 | down | -0.9928043 | 0.0373989 |
| TMEM170A | ENST00000561878 | down | -0.99308 | 0.0418999 |
| C2orf74 | ENST00000464909 | down | -0.9934475 | 0.0071131 |
| PRKRA | ENST00000487082 | down | -0.9937818 | 0.0182395 |
| PEX3 | ENST00000367591 | down | -0.9939374 | 0.0380087 |
| RWDD4 | ENST00000512740 | down | -0.9939434 | 0.0224605 |
| PET117 | ENST00000432901 | down | -0.9941811 | 0.0195612 |
| TMEM205 | ENST00000588560 | down | -0.9942282 | 0.0272538 |
| GTDC1 | ENST00000241391 | down | -0.9944246 | 0.0360539 |
| NPIPB3 | NM_130464 | down | -0.9945029 | 0.020482 |
| SAP130 | ENST00000643581 | down | -0.9945044 | 0.0082104 |
| SCD | ENST00000370355 | down | -0.9947927 | 3.128E-05 |
| TAMM41 | ENST00000273037 | down | -0.9948346 | 0.0038133 |
| CUEDC2 | ENST00000369937 | down | -0.9949866 | 0.0163384 |
| VEGFA | ENST00000520948 | down | -0.9952118 | 0.0079139 |
| ELMOD3 | ENST00000315658 | down | -0.9953434 | 0.0215589 |
| UVSSA | ENST00000511216 | down | -0.9954106 | 0.0374596 |
| ZNF174 | ENST00000571936 | down | -0.9955735 | 0.0347301 |
| LILRA1 | ENST00000251372 | down | -0.9956812 | 0.0122988 |
| CCBL2 | uc001dmr.2 | down | -0.995741 | 0.0127474 |
| OSCP1 | ENST00000354267 | down | -0.9963171 | 0.0174112 |
| TXNRD1 | ENST00000529546 | down | -0.9965141 | 0.0110043 |
| ZNF662 | ENST00000440367 | down | -0.9966327 | 0.008582 |
| DTNBP1 | ENST00000622898 | down | -0.9969885 | 0.0018258 |
| C19orf24 | ENST00000409293 | down | -0.9972915 | 0.002306 |
| ROR2 | ENST00000375708 | down | -0.9974796 | 0.019403 |
| HSD11B1 | ENST00000367027 | down | -0.9981749 | 0.034043 |
| HDAC5 | NM_005474 | down | -0.9983808 | 0.0246397 |
| LCOR | ENST00000286067 | down | -0.9995007 | 0.0094621 |
| SNX27 | ENST00000458013 | down | -1.0002684 | 0.0287204 |
| EMC8 | ENST00000435200 | down | -1.0004676 | 0.0094356 |
| UPF3B | ENST00000276201 | down | -1.0005126 | 0.0030864 |
| PARS2 | ENST00000371279 | down | -1.0007489 | 0.0205459 |
| TMEM126B | ENST00000531274 | down | -1.001771 | 0.0096217 |
| CELF1 | ENST00000532048 | down | -1.0019519 | 0.0108533 |
| ROR2 | NM_001318204 | down | -1.0019971 | 0.0153438 |
| ZNF222 | ENST00000391960 | down | -1.0021868 | 0.0013172 |
| BLOC1S2 | ENST00000370372 | down | -1.002315 | 0.0030943 |
| TBCA | ENST00000306388 | down | -1.0023746 | 0.0452958 |
| SDCCAG3 | ENST00000371725 | down | -1.0033476 | 0.0002204 |
| KLHL7 | ENST00000409689 | down | -1.0037554 | 0.0377713 |
| GGA2 | ENST00000309859 | down | -1.0038082 | 0.0173266 |
| GOLM1 | ENST00000388712 | down | -1.0038827 | 0.0150346 |
| TBL1X | ENST00000217964 | down | -1.0044754 | 0.0291367 |
| SECISBP2 | ENST00000375807 | down | -1.0045622 | 0.0303251 |
| VPS37A | ENST00000521829 | down | -1.0051721 | 0.0098686 |
| BRD4 | ENST00000263377 | down | -1.005354 | 0.0074179 |
| SMG7 | NM_001350220 | down | -1.0055278 | 0.0355521 |
| MBD1 | NM_001323942 | down | -1.0059385 | 0.0203997 |
| PLA2G4D | ENST00000290472 | down | -1.00606 | 0.0467269 |
| ADH5 | ENST00000296412 | down | -1.0067243 | 0.0236388 |
| SUPT7L | ENST00000404798 | down | -1.0069156 | 0.0149458 |
| TMEM116 | ENST00000552374 | down | -1.0072477 | 0.0452976 |
| DCAKD | ENST00000614054 | down | -1.0074969 | 0.0182614 |
| SPAG4 | NM_001317931 | down | -1.0077149 | 0.0055901 |
| GSK3B | ENST00000316626 | down | -1.0080503 | 0.0228068 |
| NABP1 | ENST00000425611 | down | -1.0081796 | 0.0339618 |
| ZNF302 | ENST00000507959 | down | -1.0089066 | 0.0265483 |
| MRPS18A | ENST00000372116 | down | -1.0092211 | 0.0005969 |
| ATP6V1A | ENST00000273398 | down | -1.0094857 | 0.0091849 |
| PCNP | ENST00000265260 | down | -1.0099747 | 0.0075698 |
| IQCB1 | ENST00000310864 | down | -1.0100995 | 0.0122041 |
| PAPOLA | ENST00000555626 | down | -1.0104685 | 0.0082056 |
| MBD1 | ENST00000590208 | down | -1.0105669 | 0.0162121 |
| SYNE4 | ENST00000324444 | down | -1.0108782 | 0.0435709 |
| UBE2J2 | ENST00000400929 | down | -1.0114187 | 0.0042144 |
| ZFP62 | ENST00000502412 | down | -1.0118104 | 0.0361944 |
| BCDIN3D | ENST00000333924 | down | -1.0122361 | 0.0266574 |
| FLCN | ENST00000285071 | down | -1.0125732 | 0.0113896 |
| GHITM | uc010qma.1 | down | -1.0130403 | 0.0106505 |
| C4orf27 | ENST00000393381 | down | -1.0132067 | 0.0057637 |
| HEYL | ENST00000372852 | down | -1.0134752 | 0.027752 |
| RBPJ | ENST00000361572 | down | -1.0136754 | 0.0090076 |
| LRRC23 | ENST00000443597 | down | -1.013704 | 0.021582 |
| SNRPE | ENST00000414487 | down | -1.0137275 | 0.0083522 |
| RP11-872D17.8 | ENST00000529411 | down | -1.014427 | 0.0029871 |
| COX7C | ENST00000247655 | down | -1.0148859 | 0.0083736 |
| RPP40 | ENST00000319533 | down | -1.0152888 | 0.0269109 |
| DCAF4 | ENST00000555042 | down | -1.0153481 | 0.0234285 |
| FAM76B | ENST00000536839 | down | -1.0158179 | 0.028063 |
| ECHDC1 | ENST00000528402 | down | -1.0163597 | 0.0076788 |
| GFRA2 | ENST00000524240 | down | -1.0164419 | 0.033664 |
| TLK1 | ENST00000434911 | down | -1.0170406 | 0.0147368 |
| FAM135A | ENST00000370479 | down | -1.0170768 | 0.009536 |
| ICE2 | ENST00000261520 | down | -1.0171332 | 0.0448572 |
| ZFP41 | ENST00000330701 | down | -1.0173902 | 0.0132811 |
| CCDC174 | ENST00000383794 | down | -1.0175943 | 0.0070065 |
| ZFAND5 | NM_006007 | down | -1.0175954 | 0.0204799 |
| YEATS4 | ENST00000247843 | down | -1.0176476 | 0.0263249 |
| ADCK3 | ENST00000366779 | down | -1.0182197 | 0.0123364 |
| CD209 | ENST00000315599 | down | -1.0186738 | 0.0244319 |
| RPAIN | ENST00000381208 | down | -1.0189644 | 0.0073019 |
| PIH1D2 | ENST00000280350 | down | -1.018983 | 0.0057542 |
| DDR1 | ENST00000508312 | down | -1.01911 | 0.0127206 |
| EIF2S2 | NM_001316363 | down | -1.0195058 | 0.0066188 |
| MPV17 | ENST00000380044 | down | -1.0197264 | 0.0195389 |
| MRPS18C | ENST00000507019 | down | -1.0198314 | 0.0156564 |
| KBTBD3 | ENST00000531837 | down | -1.0198802 | 0.0272238 |
| ATP2C1 | ENST00000508532 | down | -1.0200681 | 0.0010786 |
| ZNF568 | ENST00000415168 | down | -1.0203196 | 0.0074023 |
| KHDRBS1 | ENST00000492989 | down | -1.0203603 | 0.0057813 |
| FAM161A | ENST00000404929 | down | -1.0214445 | 0.0110824 |
| PRR7 | ENST00000502922 | down | -1.0218371 | 0.0124671 |
| MGRN1 | ENST00000588994 | down | -1.021856 | 0.0178596 |
| METTL23 | ENST00000586752 | down | -1.022141 | 0.0169218 |
| OR8A1 | ENST00000641670 | down | -1.0222706 | 0.0198305 |
| KLK11 | ENST00000594768 | down | -1.0228634 | 0.0152577 |
| BMP2K | ENST00000502613 | down | -1.0231423 | 0.0295287 |
| EMC8 | ENST00000253457 | down | -1.0236296 | 0.0026818 |
| PEPD | ENST00000397032 | down | -1.0240654 | 0.0163989 |
| TBATA | NM_001318243 | down | -1.0240676 | 0.0016535 |
| MT-ND2 | ENST00000361453 | down | -1.0246676 | 0.0061863 |
| IQCB1 | ENST00000349820 | down | -1.0246723 | 0.0217575 |
| CREM | ENST00000344351 | down | -1.024979 | 0.0108641 |
| ACAT1 | ENST00000265838 | down | -1.0252192 | 0.0014345 |
| FHOD1 | ENST00000258201 | down | -1.0252856 | 0.0316714 |
| AIFM1 | ENST00000287295 | down | -1.0254676 | 0.0155286 |
| SCMH1 | ENST00000372597 | down | -1.0256268 | 0.0191138 |
| SNRNP40 | uc010oge.2 | down | -1.0258649 | 0.0015448 |
| SEC23A | ENST00000307712 | down | -1.0259455 | 0.0487771 |
| YBEY | ENST00000339195 | down | -1.0264958 | 0.0002521 |
| RAP1B | ENST00000540209 | down | -1.026722 | 0.0018129 |
| RBM41 | ENST00000372479 | down | -1.0274161 | 0.0389856 |
| KITLG | ENST00000347404 | down | -1.0278364 | 0.0132261 |
| MRPS30 | ENST00000507110 | down | -1.0279455 | 3.742E-05 |
| PTER | NM_030664 | down | -1.0281211 | 0.0385026 |
| PTPRE | NM_001323354 | down | -1.028158 | 0.0480987 |
| CHMP2A | ENST00000601220 | down | -1.0283595 | 0.0054475 |
| SETD9 | ENST00000285947 | down | -1.0285369 | 0.0341315 |
| ARMC10 | ENST00000541300 | down | -1.0287897 | 0.0001125 |
| DIMT1 | ENST00000506390 | down | -1.0289861 | 0.0098395 |
| ACBD4 | ENST00000321854 | down | -1.0294819 | 0.0414168 |
| MBD1 | ENST00000269468 | down | -1.0298524 | 0.0037878 |
| RABGGTB | ENST00000319942 | down | -1.0303617 | 0.0256205 |
| FHL2 | ENST00000393353 | down | -1.0307914 | 0.0233794 |
| STAMBP | ENST00000339566 | down | -1.0324853 | 0.005356 |
| CATG00000116292.1 | HBMT00000153253 | down | -1.0330885 | 0.0144243 |
| ERP29 | ENST00000455836 | down | -1.033554 | 0.0315757 |
| B3GALNT2 | ENST00000366600 | down | -1.0343812 | 0.0001769 |
| ME3 | ENST00000393324 | down | -1.0343953 | 0.025411 |
| VEGFA | ENST00000372077 | down | -1.0349145 | 0.0192584 |
| C11orf74 | ENST00000334307 | down | -1.0354303 | 0.0066503 |
| FNIP1 | ENST00000307968 | down | -1.0355251 | 0.0093378 |
| SOS2 | ENST00000216373 | down | -1.0358162 | 0.0044625 |
| GTF2E2 | ENST00000355904 | down | -1.0361725 | 0.0189196 |
| FAM98C | ENST00000252530 | down | -1.0370028 | 0.0463078 |
| ZDHHC22 | ENST00000319374 | down | -1.0378959 | 0.0220339 |
| MIS12 | ENST00000573759 | down | -1.0381347 | 0.0404678 |
| PTP4A3 | ENST00000329397 | down | -1.0387933 | 0.008508 |
| CHCHD8 | ENST00000541455 | down | -1.0390609 | 0.0106881 |
| CYSTM1 | ENST00000261811 | down | -1.0393827 | 0.0015812 |
| RBBP6 | ENST00000348022 | down | -1.0397571 | 0.0482964 |
| ZNF577 | ENST00000301399 | down | -1.0397865 | 0.0249154 |
| C11orf1 | ENST00000530214 | down | -1.0400819 | 0.0140825 |
| TNFRSF14 | ENST00000355716 | down | -1.040093 | 0.0040423 |
| FHL2 | ENST00000322142 | down | -1.0409306 | 0.0175795 |
| RPS18 | ENST00000439602 | down | -1.0416803 | 0.0012689 |
| ERCC8 | NM_001290285 | down | -1.0417068 | 0.0026219 |
| UBE2R2 | ENST00000263228 | down | -1.0419399 | 0.0142137 |
| ARV1 | ENST00000310256 | down | -1.0421004 | 0.0343262 |
| RAD1 | ENST00000382038 | down | -1.0425122 | 0.0199458 |
| SENP6 | ENST00000327284 | down | -1.0425273 | 0.0012959 |
| TOP1MT | ENST00000329245 | down | -1.0426027 | 0.0395587 |
| USP38 | ENST00000510377 | down | -1.0429528 | 0.0146263 |
| UTP20 | ENST00000261637 | down | -1.043215 | 0.020447 |
| EEF1E1 | ENST00000379715 | down | -1.0435723 | 0.0013387 |
| POLR1C | ENST00000304004 | down | -1.0438895 | 0.0018946 |
| TRIM37 | ENST00000393066 | down | -1.0439784 | 0.0332553 |
| COPB1 | ENST00000439561 | down | -1.0451614 | 0.0023316 |
| ADAL | ENST00000428046 | down | -1.0452462 | 0.0207379 |
| ECEL1 | ENST00000304546 | down | -1.0460152 | 0.0130032 |
| CHCHD3 | ENST00000262570 | down | -1.0464251 | 0.0059233 |
| YEATS4 | ENST00000548020 | down | -1.046567 | 0.0145676 |
| SQSTM1 | ENST00000389805 | down | -1.046724 | 0.008967 |
| C1orf43 | ENST00000350592 | down | -1.0471201 | 0.0004241 |
| DNAJC17 | ENST00000220496 | down | -1.0484998 | 0.0196871 |
| ZNF593 | ENST00000374266 | down | -1.048608 | 0.0019463 |
| IARS | ENST00000447699 | down | -1.0488586 | 0.0157178 |
| LSM5 | ENST00000450169 | down | -1.0489085 | 0.0475237 |
| RAP1GDS1 | ENST00000339360 | down | -1.0495367 | 0.0027305 |
| MRPL32 | ENST00000223324 | down | -1.0497986 | 0.0045351 |
| NUPR1 | ENST00000395641 | down | -1.0499043 | 0.0413609 |
| CMTR2 | ENST00000338099 | down | -1.0507876 | 0.017315 |
| OGFOD2 | ENST00000454694 | down | -1.0513581 | 0.0003195 |
| RPL7 | ENST00000352983 | down | -1.0515168 | 0.0154761 |
| PCDH9 | ENST00000456367 | down | -1.0515456 | 0.0403211 |
| NECAB2 | ENST00000565691 | down | -1.0520312 | 0.0067804 |
| ARFGEF2 | ENST00000371917 | down | -1.0522148 | 0.003267 |
| IFI16 | ENST00000359709 | down | -1.0525621 | 0.002134 |
| ZSCAN25 | NM_001350984 | down | -1.0528925 | 0.0437873 |
| DNMT3A | ENST00000321117 | down | -1.0529017 | 0.0086318 |
| ADORA2B | ENST00000304222 | down | -1.0531281 | 0.0346383 |
| HSD17B8 | ENST00000374662 | down | -1.0536669 | 0.0242205 |
| RABEP1 | NM_001083585 | down | -1.0539532 | 0.0433306 |
| GXYLT2 | ENST00000389617 | down | -1.0541337 | 0.0047778 |
| LBP | ENST00000217407 | down | -1.0544006 | 0.0256948 |
| HKR1 | ENST00000392153 | down | -1.0544019 | 0.0090631 |
| ZSCAN16 | ENST00000340487 | down | -1.0544178 | 0.0034804 |
| BRF2 | ENST00000220659 | down | -1.0545732 | 0.0003347 |
| BRF2 | ENST00000220659 | down | -1.0545732 | 0.0003347 |
| C9orf85 | ENST00000334731 | down | -1.054677 | 0.0041292 |
| CCDC115 | ENST00000409127 | down | -1.0547281 | 0.0048975 |
| OFD1 | ENST00000340096 | down | -1.0552559 | 0.0335844 |
| CHMP2B | ENST00000471660 | down | -1.0552979 | 0.0063287 |
| AKIP1 | ENST00000534147 | down | -1.055433 | 0.0123662 |
| SYF2 | ENST00000236273 | down | -1.0556095 | 0.0053223 |
| HCCS | ENST00000321143 | down | -1.0557854 | 0.0027732 |
| PARN | ENST00000420015 | down | -1.0563511 | 0.0104909 |
| CCDC50 | ENST00000392456 | down | -1.0566773 | 0.0111665 |
| RCL1 | ENST00000381750 | down | -1.0568944 | 0.0422979 |
| MRPL49 | ENST00000279242 | down | -1.0569363 | 0.013652 |
| MRPL49 | ENST00000279242 | down | -1.0569363 | 0.013652 |
| RNF138 | NM_001191324 | down | -1.0570578 | 0.002188 |
| NTMT1 | ENST00000372486 | down | -1.0577404 | 0.021926 |
| CDC73 | ENST00000367435 | down | -1.0579196 | 0.0093934 |
| SPNS1 | ENST00000334536 | down | -1.0580741 | 0.0138601 |
| FAM13B | ENST00000033079 | down | -1.0584202 | 0.0357038 |
| TOMM5 | ENST00000544379 | down | -1.0586793 | 0.0099659 |
| CHCHD1 | ENST00000372833 | down | -1.0588099 | 0.0082247 |
| HIST2H2BF | ENST00000369167 | down | -1.0589276 | 0.000119 |
| TUBGCP4 | ENST00000564079 | down | -1.0590074 | 0.0144656 |
| SRSF5 | ENST00000394366 | down | -1.0593596 | 0.0245222 |
| CHMP2B | ENST00000263780 | down | -1.0594541 | 0.0046689 |
| MMACHC | ENST00000616135 | down | -1.0598077 | 0.0008786 |
| FIG4 | ENST00000230124 | down | -1.0598716 | 0.0038173 |
| PRB1 | ENST00000500254 | down | -1.0600319 | 0.0269733 |
| MUM1 | NM_032853 | down | -1.0604182 | 0.0046919 |
| CTSA | ENST00000354880 | down | -1.0609594 | 0.0037585 |
| EZH1 | ENST00000415827 | down | -1.0613003 | 0.0195692 |
| CWC25 | ENST00000614790 | down | -1.0615051 | 0.0307649 |
| POLR3G | ENST00000399107 | down | -1.0619086 | 0.0126904 |
| NPM1 | ENST00000351986 | down | -1.0619339 | 0.0113813 |
| EIF5A2 | ENST00000295822 | down | -1.0619551 | 0.0056624 |
| AMPD2 | ENST00000342115 | down | -1.0624311 | 0.0001439 |
| CREM | ENST00000348787 | down | -1.0625107 | 0.0291446 |
| NETO2 | ENST00000303155 | down | -1.0625822 | 0.0247294 |
| FUCA2 | uc003qjn.3 | down | -1.0626771 | 0.0369049 |
| MRPL24 | ENST00000361531 | down | -1.0628514 | 0.0089071 |
| NDRG2 | ENST00000554104 | down | -1.0629741 | 0.033479 |
| PARP11 | ENST00000228820 | down | -1.0630222 | 0.0251663 |
| SNX2 | ENST00000514949 | down | -1.0631871 | 0.0042226 |
| HEG1 | ENST00000311127 | down | -1.0633798 | 0.0242568 |
| CCDC113 | ENST00000219299 | down | -1.0634801 | 0.0063158 |
| PRDM5 | ENST00000515109 | down | -1.0641205 | 0.0008059 |
| ZNF787 | ENST00000610935 | down | -1.064227 | 0.0096033 |
| RPS19 | ENST00000593863 | down | -1.0642888 | 0.000654 |
| DOCK11 | ENST00000276202 | down | -1.0642904 | 0.0111531 |
| RNF14 | ENST00000356143 | down | -1.0643844 | 0.012157 |
| WDR4 | ENST00000330317 | down | -1.0650237 | 0.0164917 |
| ALG14 | ENST00000370205 | down | -1.0652341 | 0.009106 |
| KDSR | ENST00000645214 | down | -1.0659751 | 0.0021198 |
| ATP9B | ENST00000307671 | down | -1.0661105 | 0.0299749 |
| THNSL2 | ENST00000377254 | down | -1.0661214 | 0.0036961 |
| TOP3B | NM_001349852 | down | -1.0661694 | 0.0164297 |
| NKAP | ENST00000371410 | down | -1.0669488 | 0.0060133 |
| MDM1 | ENST00000430606 | down | -1.0670753 | 0.0085405 |
| ZNF568 | ENST00000333987 | down | -1.068029 | 0.006321 |
| CCNL1 | ENST00000295926 | down | -1.0685286 | 0.0119692 |
| FBXO22 | ENST00000308275 | down | -1.0685309 | 0.0075454 |
| G0S2 | ENST00000367029 | down | -1.0685566 | 0.0063602 |
| NOL8 | ENST00000535387 | down | -1.0690382 | 0.0362082 |
| ULBP1 | ENST00000229708 | down | -1.069209 | 0.0035879 |
| NCK2 | ENST00000451463 | down | -1.0696539 | 0.0056615 |
| MRPL52 | ENST00000397496 | down | -1.0696741 | 0.0135554 |
| PSMC1 | ENST00000543772 | down | -1.0703553 | 0.0370469 |
| PQBP1 | ENST00000447146 | down | -1.0708898 | 0.0292498 |
| LMO4 | ENST00000370544 | down | -1.0711643 | 0.0124851 |
| ZBTB44 | ENST00000530205 | down | -1.0713632 | 0.0077916 |
| RNH1 | ENST00000533410 | down | -1.0713711 | 0.0152175 |
| GTPBP2 | ENST00000307126 | down | -1.0715728 | 0.0050111 |
| NDUFAF4 | ENST00000316149 | down | -1.0716447 | 0.0415945 |
| METTL23 | ENST00000590964 | down | -1.0718593 | 0.0261956 |
| DNAJB5 | ENST00000453597 | down | -1.072047 | 0.0330243 |
| ANTXR2 | ENST00000307333 | down | -1.0720815 | 0.0382702 |
| FOPNL | ENST00000573396 | down | -1.0725891 | 0.0181456 |
| PRAME | ENST00000398743 | down | -1.0728615 | 0.0260678 |
| ALKBH3 | ENST00000302708 | down | -1.0736348 | 0.0002723 |
| SRPK3 | ENST00000370101 | down | -1.0737023 | 0.0260093 |
| NMRK1 | ENST00000376808 | down | -1.0737209 | 0.0176034 |
| MIEN1 | ENST00000577810 | down | -1.0737523 | 0.0407193 |
| SHMT1 | ENST00000316694 | down | -1.0742727 | 0.0073306 |
| CLN6 | ENST00000249806 | down | -1.0746613 | 0.0498183 |
| GALE | ENST00000374497 | down | -1.0747098 | 0.0029431 |
| TTC1 | ENST00000522793 | down | -1.0747532 | 0.0025712 |
| NCK1 | ENST00000469404 | down | -1.0747784 | 0.0493484 |
| DNAJC24 | uc009yjm.3 | down | -1.0757449 | 0.0179044 |
| SRRM5 | ENST00000417606 | down | -1.0758485 | 0.0242574 |
| ADSL | ENST00000342312 | down | -1.0758832 | 0.0010062 |
| SDHD | ENST00000525291 | down | -1.0759145 | 0.0202693 |
| RAB27A | ENST00000396307 | down | -1.0761334 | 0.0326295 |
| TLK1 | ENST00000431350 | down | -1.076224 | 0.0263632 |
| ADA | ENST00000372874 | down | -1.0764875 | 0.0054935 |
| VWA8 | ENST00000281496 | down | -1.0765118 | 0.0454495 |
| CMSS1 | ENST00000421999 | down | -1.0767217 | 9.256E-05 |
| ANAPC16 | ENST00000621663 | down | -1.0772865 | 0.0008359 |
| P2RX2 | ENST00000348800 | down | -1.077649 | 0.0194549 |
| ATRN | ENST00000446916 | down | -1.0777167 | 0.0031361 |
| TATDN1 | ENST00000276692 | down | -1.078268 | 0.0017427 |
| TMEM168 | ENST00000454074 | down | -1.0794015 | 0.022783 |
| APP | ENST00000346798 | down | -1.0794886 | 0.0089595 |
| C14orf159 | ENST00000428926 | down | -1.080772 | 0.0113382 |
| SLC25A38 | ENST00000273158 | down | -1.0808096 | 0.0299608 |
| TBL3 | ENST00000568546 | down | -1.0808114 | 0.041069 |
| SDHD | ENST00000528048 | down | -1.080856 | 0.0304699 |
| PAPD5 | NM_001040285 | down | -1.0808707 | 0.0306193 |
| APP | ENST00000357903 | down | -1.0809559 | 0.0205899 |
| PAPPA | ENST00000328252 | down | -1.0813208 | 0.0075808 |
| EIF3J | ENST00000424492 | down | -1.0814197 | 0.0094739 |
| VPS26A | NM_001318944 | down | -1.0818867 | 0.0024508 |
| PRDM5 | ENST00000428209 | down | -1.0819157 | 0.0106795 |
| EXTL2 | ENST00000370113 | down | -1.0819902 | 0.0215002 |
| ANAPC13 | ENST00000354910 | down | -1.0820277 | 0.0050746 |
| MBD1 | ENST00000398495 | down | -1.0826202 | 0.0072524 |
| SVBP | ENST00000372521 | down | -1.0826352 | 0.0018314 |
| GMPR2 | ENST00000456667 | down | -1.0828258 | 0.0158964 |
| ESCO1 | ENST00000269214 | down | -1.0828346 | 0.0172625 |
| TMEM55B | ENST00000250489 | down | -1.0830497 | 0.0158308 |
| AAMP | ENST00000444053 | down | -1.0833572 | 0.0034983 |
| ZNF534 | ENST00000617900 | down | -1.0835892 | 0.0050637 |
| POLR1D | ENST00000647448 | down | -1.0836193 | 0.0097142 |
| ZNF404 | ENST00000587539 | down | -1.0838666 | 0.0477713 |
| CDK17 | ENST00000261211 | down | -1.0839141 | 0.0051917 |
| TXNRD1 | ENST00000388854 | down | -1.0839252 | 0.0096424 |
| MVD | ENST00000301012 | down | -1.0840712 | 0.004818 |
| GLB1 | ENST00000307377 | down | -1.0845431 | 0.0081878 |
| TTC17 | ENST00000039989 | down | -1.0845785 | 0.036403 |
| CXCL16 | ENST00000574412 | down | -1.0846608 | 0.0162563 |
| CCSER2 | ENST00000493409 | down | -1.0848012 | 0.017042 |
| REPIN1 | ENST00000425389 | down | -1.0852006 | 0.0289803 |
| EFEMP1 | ENST00000394555 | down | -1.0862503 | 0.0041176 |
| CNTLN | ENST00000380647 | down | -1.0869758 | 0.044156 |
| PCYT2 | NM_001256434 | down | -1.0871253 | 0.0032952 |
| DSCR3 | ENST00000309117 | down | -1.0871346 | 0.0086762 |
| FIBIN | ENST00000318627 | down | -1.0874135 | 0.0464179 |
| TSEN15 | ENST00000645668 | down | -1.087476 | 0.0210718 |
| PTPN13 | ENST00000427191 | down | -1.0878498 | 0.0061354 |
| SIRT2 | uc002ojv.2 | down | -1.0879017 | 0.0093694 |
| PPP1R15A | ENST00000200453 | down | -1.0879032 | 0.0373138 |
| ZNF585A | ENST00000588354 | down | -1.0882449 | 0.0152398 |
| ZNF26 | ENST00000328654 | down | -1.088365 | 0.0124337 |
| FAM206A | ENST00000322940 | down | -1.0883719 | 0.0186095 |
| CTSC | ENST00000529974 | down | -1.0884392 | 0.0114285 |
| THYN1 | ENST00000392595 | down | -1.0887563 | 0.0038665 |
| CRELD1 | ENST00000383811 | down | -1.0889032 | 0.0012392 |
| RPS3 | ENST00000278572 | down | -1.0892247 | 0.0273839 |
| DOCK11 | ENST00000276204 | down | -1.0893374 | 0.0056031 |
| C7orf49 | ENST00000483029 | down | -1.0894752 | 0.0131732 |
| CPEB4 | ENST00000265085 | down | -1.0895162 | 0.0460756 |
| CALCR | ENST00000426151 | down | -1.08982 | 0.0050248 |
| ZCCHC4 | ENST00000612982 | down | -1.0901607 | 0.0014034 |
| GBAS | ENST00000446778 | down | -1.0907062 | 0.0093304 |
| ORMDL1 | ENST00000325795 | down | -1.0911752 | 0.0266104 |
| PSAP | ENST00000394936 | down | -1.0912443 | 0.0069684 |
| TRIT1 | NM_001312691 | down | -1.0915143 | 0.022587 |
| BNIP1 | ENST00000352523 | down | -1.0916786 | 0.0004603 |
| ERAP1 | ENST00000296754 | down | -1.0939288 | 0.00553 |
| ANKRD10 | ENST00000310847 | down | -1.0945475 | 0.0108532 |
| ZNF566 | ENST00000392170 | down | -1.0946776 | 0.021209 |
| NKAPL | ENST00000343684 | down | -1.0949976 | 0.0267598 |
| AC090186.1 | ENST00000415643 | down | -1.0950433 | 0.0021025 |
| SLC33A1 | ENST00000359479 | down | -1.095301 | 0.0098674 |
| VEPH1 | ENST00000468233 | down | -1.0958821 | 0.0156136 |
| NSRP1 | ENST00000247026 | down | -1.0961325 | 0.0225031 |
| CSNK2B | ENST00000375882 | down | -1.0963669 | 0.0003204 |
| CCDC91 | ENST00000539107 | down | -1.0964805 | 0.0141444 |
| AASDH | ENST00000502617 | down | -1.0972169 | 0.0461756 |
| CATG00000031631.1 | ENCT00000176029 | down | -1.0974028 | 0.0171867 |
| MED6 | ENST00000256379 | down | -1.097821 | 0.0018859 |
| UPF3A | ENST00000375299 | down | -1.0979293 | 0.0058386 |
| DPP4 | ENST00000360534 | down | -1.0984208 | 0.0303941 |
| PROC | ENST00000234071 | down | -1.0985492 | 0.0449032 |
| EIF4B | ENST00000420463 | down | -1.0985577 | 0.0424743 |
| LGALS8 | ENST00000527974 | down | -1.0989521 | 0.0024674 |
| TINCR | ENST00000448587 | down | -1.0991589 | 0.0155814 |
| RNF41 | ENST00000345093 | down | -1.0993218 | 0.0082888 |
| CH507-9B2.9 | ENST00000623998 | down | -1.0993777 | 0.005995 |
| C14orf2 | ENST00000286953 | down | -1.0995733 | 0.0269697 |
| ZNF79 | ENST00000612342 | down | -1.1010899 | 0.0210019 |
| CSTB | ENST00000640406 | down | -1.1012296 | 0.0124111 |
| GTF3C3 | ENST00000263956 | down | -1.1014558 | 0.0020653 |
| ACSL1 | ENST00000504342 | down | -1.1016413 | 0.011888 |
| CCDC125 | ENST00000396496 | down | -1.101652 | 0.0370063 |
| GATA2 | ENST00000341105 | down | -1.1020776 | 0.0001105 |
| OSMR | NM_001323506 | down | -1.1023194 | 0.0107149 |
| C9orf9 | ENST00000350499 | down | -1.1034596 | 0.0062074 |
| FAM200B | ENST00000422728 | down | -1.1036024 | 0.0189018 |
| RABEP1 | ENST00000341923 | down | -1.1040454 | 0.0191776 |
| RNASEH2B | ENST00000336617 | down | -1.1040935 | 0.0024152 |
| RPS19 | ENST00000600467 | down | -1.1048227 | 0.0028769 |
| PRPF38A | ENST00000257181 | down | -1.1048708 | 0.003059 |
| CTSC | ENST00000524463 | down | -1.1050228 | 0.0096074 |
| PLA2G12A | ENST00000243501 | down | -1.1052409 | 0.0447365 |
| ATP6V0A1 | ENST00000393829 | down | -1.1054028 | 0.0180077 |
| NFX1 | NM_001318758 | down | -1.1055596 | 0.0136181 |
| GTF2IRD1 | ENST00000424337 | down | -1.1057989 | 0.0033311 |
| GTF2IRD1 | ENST00000424337 | down | -1.1057989 | 0.0033311 |
| C14orf179 | ENST00000238628 | down | -1.1060521 | 7.374E-05 |
| MTX3 | ENST00000509852 | down | -1.1068708 | 0.0207433 |
| LZIC | ENST00000488540 | down | -1.1075882 | 0.002833 |
| FBXW11 | ENST00000265094 | down | -1.1075892 | 0.0027683 |
| ACSL1 | ENST00000513317 | down | -1.1077931 | 0.0204898 |
| EYA4 | ENST00000531901 | down | -1.1078405 | 0.0194312 |
| PSMA3 | ENST00000557508 | down | -1.1085249 | 0.0092008 |
| TCF12 | ENST00000438423 | down | -1.1085473 | 0.0471512 |
| ZNF568 | ENST00000617745 | down | -1.1089899 | 0.0318103 |
| GJC2 | ENST00000366714 | down | -1.1090498 | 0.0405673 |
| KDM5C | ENST00000452825 | down | -1.1090902 | 0.0200237 |
| AIMP2 | ENST00000223029 | down | -1.1091463 | 0.003158 |
| RP4-548D19.5 | ENST00000222388 | down | -1.1097373 | 0.0016627 |
| CEACAM6 | ENST00000199764 | down | -1.1098447 | 0.01947 |
| BTF3L4 | ENST00000313334 | down | -1.1108365 | 0.0044305 |
| FAM204A | ENST00000369172 | down | -1.1109451 | 0.0012557 |
| SRGAP1 | ENST00000631006 | down | -1.1110772 | 0.0104286 |
| CIITA | ENST00000381835 | down | -1.1120871 | 0.0073058 |
| PCM1 | ENST00000325083 | down | -1.1122716 | 0.0212103 |
| DDX21 | ENST00000620315 | down | -1.1122856 | 0.0186265 |
| OFD1 | ENST00000380550 | down | -1.1123833 | 0.0301443 |
| ZFP92 | ENST00000338647 | down | -1.1124681 | 0.0299368 |
| UBE3B | ENST00000340074 | down | -1.1126146 | 0.0083184 |
| CEP164 | ENST00000278935 | down | -1.1126737 | 0.025317 |
| CTNS | ENST00000046640 | down | -1.1130674 | 0.0164843 |
| LAGE3 | ENST00000357360 | down | -1.1132592 | 0.0065159 |
| VEGFA | ENST00000482630 | down | -1.1146576 | 0.019785 |
| NQO2 | ENST00000380441 | down | -1.114746 | 0.0016277 |
| C14orf159 | ENST00000521077 | down | -1.115386 | 0.0114109 |
| DTNA | ENST00000598334 | down | -1.1155411 | 0.0030094 |
| RAD51C | ENST00000337432 | down | -1.1155469 | 0.0195686 |
| PAIP1 | ENST00000306846 | down | -1.1155971 | 0.0401549 |
| DES | ENST00000373960 | down | -1.1156639 | 0.0324437 |
| PDCL3 | ENST00000264254 | down | -1.1159106 | 0.013045 |
| PNRC1 | ENST00000336032 | down | -1.1162448 | 0.005119 |
| MPV17 | ENST00000233545 | down | -1.1164404 | 0.0260964 |
| NQO1 | ENST00000439109 | down | -1.1165755 | 1.486E-05 |
| OLFML3 | ENST00000320334 | down | -1.1170202 | 0.0338895 |
| MFSD11 | ENST00000336509 | down | -1.117077 | 0.0089954 |
| PPIE | NM_001319293 | down | -1.1171624 | 0.0062234 |
| EIF2S2 | ENST00000374980 | down | -1.1174859 | 0.0134022 |
| KANSL3 | ENST00000431828 | down | -1.117758 | 0.0062585 |
| ARV1 | NM_001346992 | down | -1.1178225 | 0.0387671 |
| SNTB2 | ENST00000336278 | down | -1.1178752 | 0.0308343 |
| ZDHHC4 | ENST00000396707 | down | -1.1179585 | 0.0025575 |
| ENOPH1 | ENST00000509635 | down | -1.1180762 | 0.0191522 |
| DMKN | ENST00000408915 | down | -1.1186461 | 0.0026026 |
| VPS26A | ENST00000263559 | down | -1.1191343 | 0.0046292 |
| AMDHD2 | ENST00000302956 | down | -1.1192587 | 0.0329631 |
| COX15 | NM_001320975 | down | -1.1199358 | 0.0099659 |
| CEBPG | ENST00000585933 | down | -1.119938 | 0.0027569 |
| CEP63 | ENST00000383229 | down | -1.1199803 | 0.0160772 |
| MTHFD2 | ENST00000394053 | down | -1.1203628 | 0.0062964 |
| PRDX5 | ENST00000347941 | down | -1.1205666 | 0.0353106 |
| NPM1 | NM_001355007 | down | -1.1209577 | 0.0031946 |
| PHF7 | ENST00000347025 | down | -1.1215344 | 0.0172428 |
| CEMP1 | ENST00000567119 | down | -1.1217098 | 0.0092147 |
| TRIM47 | ENST00000254816 | down | -1.1217434 | 0.0340954 |
| ENTPD8 | ENST00000344119 | down | -1.1217596 | 0.0083211 |
| CC2D1B | ENST00000371586 | down | -1.122023 | 0.0040079 |
| NOL9 | ENST00000377705 | down | -1.122137 | 0.0047722 |
| LTV1 | ENST00000367576 | down | -1.1222258 | 0.0128977 |
| ARL13B | ENST00000303097 | down | -1.1224438 | 0.0154769 |
| MSRB2 | ENST00000376510 | down | -1.1227984 | 0.0091754 |
| ERAL1 | NM_001317986 | down | -1.1233462 | 0.0330594 |
| ICT1 | NM_001303265 | down | -1.123443 | 0.0004521 |
| CKM | ENST00000221476 | down | -1.1235178 | 0.0384608 |
| GPX2 | ENST00000389614 | down | -1.1236521 | 0.0170596 |
| KIAA0753 | NM_001351225 | down | -1.1241188 | 0.0055314 |
| COX4I1 | ENST00000561569 | down | -1.1255422 | 0.0050315 |
| RAB3IL1 | ENST00000301773 | down | -1.1256232 | 0.0484441 |
| CLASRP | ENST00000221455 | down | -1.125704 | 0.0416868 |
| RPP21 | ENST00000433076 | down | -1.1260203 | 0.0173818 |
| RBFOX2 | ENST00000405409 | down | -1.1262316 | 0.0070882 |
| PER3 | ENST00000361923 | down | -1.1268561 | 0.0102482 |
| MIEF1 | ENST00000325301 | down | -1.1268907 | 0.0040602 |
| PTER | ENST00000423462 | down | -1.1270193 | 0.00276 |
| VEGFA | ENST00000425836 | down | -1.127253 | 0.0305712 |
| ACTR6 | ENST00000546902 | down | -1.1274707 | 0.0318587 |
| TRAPPC2L | ENST00000568583 | down | -1.1274976 | 0.0107986 |
| ATXN1 | ENST00000436367 | down | -1.1276423 | 0.0020306 |
| C1GALT1C1L | ENST00000475092 | down | -1.1276459 | 0.0277028 |
| ASAH1 | ENST00000262097 | down | -1.1279913 | 0.0238092 |
| C15orf57 | ENST00000561011 | down | -1.1281841 | 0.0090336 |
| TACO1 | ENST00000258975 | down | -1.1283142 | 0.020117 |
| BLOC1S2 | ENST00000618916 | down | -1.1285663 | 0.0118722 |
| PRELID3B | ENST00000355937 | down | -1.1294168 | 0.0269464 |
| PCK2 | ENST00000559250 | down | -1.1296719 | 0.0004908 |
| NCBP2 | ENST00000452404 | down | -1.1300654 | 0.0041794 |
| RNF216 | ENST00000389902 | down | -1.1301497 | 0.0183472 |
| CNIH4 | ENST00000366856 | down | -1.1302212 | 0.047558 |
| CHD2 | ENST00000420239 | down | -1.1307124 | 0.0082102 |
| ARHGEF7 | ENST00000646102 | down | -1.1315734 | 0.0032349 |
| ING3 | ENST00000315870 | down | -1.1315771 | 0.0113344 |
| MAPK8IP3 | ENST00000610761 | down | -1.1316572 | 0.0138039 |
| SNAPIN | ENST00000368685 | down | -1.13185 | 0.0024549 |
| MAN2C1 | ENST00000565683 | down | -1.1323361 | 0.0118423 |
| TAF11 | ENST00000420584 | down | -1.1323863 | 0.0036422 |
| JOSD1 | NM_014876 | down | -1.1328683 | 0.0318473 |
| ZNF22 | ENST00000298299 | down | -1.1332482 | 0.0231637 |
| GGPS1 | ENST00000282841 | down | -1.1333888 | 0.044317 |
| RRM2B | ENST00000395912 | down | -1.1335408 | 0.0223584 |
| AL353354.2 | ENST00000596102 | down | -1.134119 | 0.007974 |
| SRFBP1 | ENST00000339397 | down | -1.135018 | 0.0025872 |
| MON1A | ENST00000455683 | down | -1.1350899 | 0.0233888 |
| MCTP1 | ENST00000515393 | down | -1.1356289 | 0.0379001 |
| FMO4 | ENST00000367749 | down | -1.1362201 | 0.0157324 |
| PNPO | ENST00000642017 | down | -1.1362271 | 0.0087532 |
| SF3B6 | ENST00000233468 | down | -1.1370084 | 0.0023748 |
| ZNF410 | ENST00000540593 | down | -1.137089 | 0.031683 |
| TMEM91 | ENST00000436170 | down | -1.138208 | 0.0285323 |
| NFKBIB | ENST00000392079 | down | -1.1392353 | 0.00872 |
| SIPA1L2 | ENST00000366630 | down | -1.1393436 | 0.0072014 |
| SNRPC | ENST00000374017 | down | -1.1395058 | 0.046892 |
| SUPT7L | ENST00000337768 | down | -1.139581 | 0.0142621 |
| ATP6V1C1 | ENST00000395862 | down | -1.1400283 | 0.0166077 |
| ZNF138 | ENST00000307355 | down | -1.1404259 | 0.0035786 |
| LCMT1 | ENST00000399069 | down | -1.1416548 | 0.0036563 |
| ARMCX6 | ENST00000361910 | down | -1.1417973 | 0.0095221 |
| OLA1 | ENST00000284719 | down | -1.1422528 | 0.0280438 |
| PPIE | ENST00000372830 | down | -1.1424533 | 0.0125334 |
| PIGL | ENST00000395844 | down | -1.1428721 | 0.0037863 |
| PPP1R12B | ENST00000480184 | down | -1.1429777 | 0.0292903 |
| TMEM126A | ENST00000304511 | down | -1.1430445 | 0.0228232 |
| BEX2 | ENST00000372674 | down | -1.1435806 | 0.0012735 |
| DHODH | ENST00000572887 | down | -1.1438665 | 0.0317907 |
| ACY1 | ENST00000476854 | down | -1.1439026 | 0.0001695 |
| TMEM42 | ENST00000302392 | down | -1.1439068 | 0.0130906 |
| LARP6 | ENST00000299213 | down | -1.1440789 | 0.0166424 |
| FXN | ENST00000396366 | down | -1.1441115 | 0.002523 |
| ARL4C | ENST00000390645 | down | -1.1444007 | 0.0204432 |
| WWOX | ENST00000355860 | down | -1.1446594 | 0.0222781 |
| RBM23 | ENST00000399922 | down | -1.1446668 | 0.0044723 |
| LIPE | ENST00000244289 | down | -1.1449 | 0.0027002 |
| HPS4 | ENST00000398145 | down | -1.1450702 | 0.0003868 |
| PTPMT1 | ENST00000326674 | down | -1.1451564 | 0.0057529 |
| SNX1 | ENST00000261889 | down | -1.1454301 | 0.0384975 |
| NFIL3 | NM_001290000 | down | -1.1455262 | 0.0219489 |
| SLC1A5 | ENST00000542575 | down | -1.1460422 | 0.0276015 |
| SCP2 | ENST00000371509 | down | -1.1472388 | 0.0252975 |
| PRB4 | ENST00000445719 | down | -1.147578 | 0.0015184 |
| HDAC9 | ENST00000524023 | down | -1.1479213 | 0.0031342 |
| ARL14EP | ENST00000282032 | down | -1.1482171 | 0.0141202 |
| VEGFA | ENST00000519767 | down | -1.1485503 | 0.0069461 |
| C1orf43 | NM_001297718 | down | -1.1486547 | 0.0015692 |
| DTNB | ENST00000406818 | down | -1.1487101 | 0.0499145 |
| DCAF4 | NM_001352448 | down | -1.1492087 | 0.02535 |
| TFAM | ENST00000487519 | down | -1.1493093 | 0.0096734 |
| SRGAP3 | ENST00000360413 | down | -1.1497689 | 0.0413998 |
| WDR74 | ENST00000311713 | down | -1.1500453 | 0.0039954 |
| MIB2 | ENST00000505820 | down | -1.1502584 | 0.008344 |
| NDRG2 | ENST00000360463 | down | -1.1503326 | 0.0057927 |
| TMEM186 | ENST00000333050 | down | -1.1505277 | 0.0318143 |
| KAT8 | ENST00000448516 | down | -1.1509082 | 0.0487808 |
| NFX1 | ENST00000379540 | down | -1.1509486 | 0.0090393 |
| RWDD4 | NM_001307922 | down | -1.1510903 | 0.0187823 |
| PELP1 | ENST00000301396 | down | -1.1513602 | 0.0162178 |
| PELP1 | ENST00000301396 | down | -1.1513602 | 0.0162178 |
| ELP4 | ENST00000379163 | down | -1.1529077 | 0.0082641 |
| STOM | ENST00000347359 | down | -1.1534691 | 0.0164949 |
| SDR39U1 | ENST00000538105 | down | -1.1537934 | 0.0007495 |
| SNW1 | ENST00000555761 | down | -1.1538537 | 0.0224169 |
| IFT27 | ENST00000340630 | down | -1.154168 | 0.0017867 |
| ZNF277 | ENST00000421043 | down | -1.1542109 | 0.023114 |
| ASPSCR1 | ENST00000306729 | down | -1.1548023 | 0.0138583 |
| MRPS35 | ENST00000081029 | down | -1.1548474 | 0.0077521 |
| IMMP2L | ENST00000405709 | down | -1.155302 | 0.0228974 |
| FAM98C | ENST00000343358 | down | -1.155537 | 0.0178139 |
| RPS3 | ENST00000422465 | down | -1.1556062 | 0.0019408 |
| ERN2 | ENST00000457008 | down | -1.1557773 | 0.033173 |
| UBE3B | ENST00000536398 | down | -1.1566097 | 0.0188278 |
| TAF7 | ENST00000313368 | down | -1.1573248 | 0.016764 |
| GM2A | ENST00000357164 | down | -1.1574307 | 0.0022431 |
| GBA2 | ENST00000378103 | down | -1.1577828 | 0.045104 |
| EMC2 | ENST00000220853 | down | -1.1577944 | 0.0120693 |
| NQO1 | ENST00000379046 | down | -1.1580555 | 0.0160774 |
| IFT22 | ENST00000621899 | down | -1.1581339 | 0.0106907 |
| ZFAND1 | ENST00000519523 | down | -1.1585334 | 0.0031765 |
| CREM | ENST00000354759 | down | -1.1588898 | 0.0056188 |
| ERMARD | ENST00000418781 | down | -1.158997 | 0.0010018 |
| IP6K2 | ENST00000340879 | down | -1.1591286 | 0.0013729 |
| TBCA | ENST00000520361 | down | -1.159352 | 0.0004241 |
| ZMYM5 | ENST00000382907 | down | -1.1593968 | 0.0338129 |
| RNF20 | ENST00000389120 | down | -1.1594767 | 0.036987 |
| DNPH1 | ENST00000509253 | down | -1.159529 | 0.0214473 |
| C19orf73 | ENST00000408991 | down | -1.1598824 | 0.0035154 |
| HDAC9 | ENST00000456174 | down | -1.1599793 | 0.0186323 |
| MRPL21 | ENST00000450904 | down | -1.16057 | 3.047E-05 |
| FAM86C1 | ENST00000346333 | down | -1.1608023 | 0.0012975 |
| MRPL52 | ENST00000432849 | down | -1.1612305 | 0.0003076 |
| PMS1 | ENST00000409823 | down | -1.1612988 | 0.0077456 |
| NFE2L2 | ENST00000397063 | down | -1.1616475 | 0.0307267 |
| IMPA1 | ENST00000256108 | down | -1.1618906 | 0.0098162 |
| PMS1 | ENST00000409985 | down | -1.1621346 | 0.0117286 |
| KRT8 | ENST00000619952 | down | -1.1628782 | 0.0357458 |
| RP11-429J17.8 | ENST00000533004 | down | -1.1629328 | 0.0479787 |
| CSRP2BP | ENST00000435364 | down | -1.1642784 | 0.0076799 |
| RSL1D1 | ENST00000571133 | down | -1.1646084 | 0.0413292 |
| DGUOK | ENST00000629438 | down | -1.1646104 | 0.0060137 |
| OSBP2 | ENST00000535268 | down | -1.1649803 | 0.0025368 |
| SEC11C | ENST00000509791 | down | -1.1655669 | 0.0185541 |
| AP1AR | ENST00000309703 | down | -1.1658977 | 0.0006682 |
| VEGFA | ENST00000523125 | down | -1.1659058 | 0.006818 |
| VDAC2 | ENST00000313132 | down | -1.1663949 | 0.0112915 |
| RAB29 | ENST00000437324 | down | -1.167165 | 0.0105746 |
| SUPT7L | NM_001282729 | down | -1.167379 | 0.0075008 |
| BLOC1S2 | ENST00000614731 | down | -1.167893 | 0.014222 |
| SLC18B1 | ENST00000275227 | down | -1.1688481 | 0.0017698 |
| CCDC51 | ENST00000442740 | down | -1.1691363 | 0.0001472 |
| CATG00000002513.1 | FTMT24400002846 | down | -1.169141 | 0.0450288 |
| PMS1 | ENST00000624204 | down | -1.1691499 | 0.0331876 |
| MCUR1 | ENST00000379170 | down | -1.1695736 | 0.0162882 |
| TRIM37 | ENST00000393065 | down | -1.1697535 | 0.0069099 |
| C8orf59 | ENST00000612977 | down | -1.1698092 | 0.0058696 |
| MIB2 | ENST00000355826 | down | -1.1698856 | 0.0190181 |
| AMN1 | ENST00000536761 | down | -1.1715092 | 0.0001606 |
| TRMT10B | ENST00000377754 | down | -1.171647 | 0.0173805 |
| NAE1 | ENST00000379463 | down | -1.1724605 | 0.0173972 |
| FAM228B | ENST00000613899 | down | -1.1737165 | 0.0111109 |
| ING3 | ENST00000339121 | down | -1.1739028 | 0.0026558 |
| C1orf43 | ENST00000640799 | down | -1.1744385 | 0.0132145 |
| BRE | ENST00000361704 | down | -1.175475 | 0.0052595 |
| NOL10 | ENST00000381685 | down | -1.1755297 | 0.006641 |
| SLC2A11 | ENST00000345044 | down | -1.1756336 | 0.0223416 |
| PRKG1 | ENST00000646535 | down | -1.1756664 | 0.0184176 |
| PAK1IP1 | ENST00000379568 | down | -1.1760635 | 0.0078226 |
| ATXN2L | ENST00000340394 | down | -1.1771895 | 0.033563 |
| ZNF552 | ENST00000391701 | down | -1.1773326 | 0.0158774 |
| FLNB | ENST00000490882 | down | -1.1774345 | 0.007456 |
| MBD1 | ENST00000269471 | down | -1.1775226 | 0.039978 |
| NAMPT | ENST00000222553 | down | -1.17773 | 0.0110485 |
| CCDC91 | ENST00000381259 | down | -1.1784989 | 0.0086492 |
| CLTA | ENST00000540080 | down | -1.178565 | 0.0069413 |
| TMEM192 | ENST00000306480 | down | -1.1788236 | 0.0118729 |
| PDE4DIP | ENST00000369347 | down | -1.1796118 | 0.0039878 |
| MPC2 | ENST00000271373 | down | -1.1801643 | 0.0010099 |
| FAM96A | ENST00000380290 | down | -1.1803146 | 0.002071 |
| SP100 | ENST00000340126 | down | -1.1806391 | 0.0234421 |
| FZD3 | ENST00000537916 | down | -1.1808073 | 0.0053954 |
| NPM1 | NM_001355009 | down | -1.1812653 | 0.0063139 |
| ICT1 | ENST00000301585 | down | -1.1814616 | 0.0084461 |
| PTPRS | NM_130854 | down | -1.1815516 | 0.0033033 |
| NMRK1 | ENST00000361092 | down | -1.1819268 | 0.0104572 |
| CDK14 | ENST00000406263 | down | -1.1820573 | 0.0109721 |
| CHIC1 | ENST00000373502 | down | -1.1824029 | 0.0343535 |
| ZNF717 | NM_001324027 | down | -1.1827006 | 0.0118728 |
| RPS15A | ENST00000322989 | down | -1.1834526 | 0.0251424 |
| UBE3D | ENST00000369747 | down | -1.1835199 | 0.0324328 |
| APTX | ENST00000397172 | down | -1.1842178 | 0.0020606 |
| PCM1 | ENST00000519253 | down | -1.1845164 | 0.0186144 |
| THPO | NM_001290028 | down | -1.1856028 | 0.0447891 |
| TASP1 | ENST00000337743 | down | -1.1864967 | 0.0215214 |
| AGBL5 | ENST00000323064 | down | -1.1866118 | 0.0202197 |
| G6PD | ENST00000621232 | down | -1.1866451 | 0.0036361 |
| KMT5B | ENST00000401547 | down | -1.1869252 | 0.0124504 |
| CCDC41 | ENST00000339839 | down | -1.1874058 | 0.011182 |
| ATP1A2 | ENST00000361216 | down | -1.1876599 | 0.0476001 |
| ASPH | ENST00000517856 | down | -1.1878701 | 0.0173634 |
| FAM103A1 | ENST00000304191 | down | -1.1890384 | 0.0333088 |
| LRRC38 | ENST00000376085 | down | -1.1891626 | 0.0097755 |
| PDCD2 | ENST00000541970 | down | -1.1891634 | 0.0027675 |
| LYNX1 | ENST00000614491 | down | -1.1895481 | 0.0199189 |
| KHDRBS1 | ENST00000327300 | down | -1.1897969 | 0.028126 |
| PTP4A1 | ENST00000370651 | down | -1.1902529 | 0.0099968 |
| VPS26A | ENST00000395098 | down | -1.1904211 | 0.003607 |
| ADPRM | ENST00000379774 | down | -1.1908505 | 0.0034832 |
| PRNP | ENST00000379440 | down | -1.1908759 | 0.0249835 |
| ARHGAP5 | NM_001173 | down | -1.1911406 | 0.0081928 |
| ANKRD29 | ENST00000322980 | down | -1.1919162 | 0.0050147 |
| BRMS1 | ENST00000359957 | down | -1.1929249 | 0.040611 |
| LSM8 | ENST00000424702 | down | -1.1931208 | 0.0049224 |
| RAB3A | ENST00000222256 | down | -1.1932867 | 0.0059594 |
| BOD1 | ENST00000285908 | down | -1.1942462 | 0.0237056 |
| ERMARD | ENST00000588451 | down | -1.1943021 | 0.0399941 |
| RNF41 | ENST00000552656 | down | -1.1943658 | 0.0122725 |
| REC8 | ENST00000611366 | down | -1.1947894 | 0.0391127 |
| GPATCH8 | ENST00000591680 | down | -1.195036 | 0.0041272 |
| CNOT10 | ENST00000328834 | down | -1.1952066 | 0.0122862 |
| ASCC3 | ENST00000522650 | down | -1.19551 | 0.0185374 |
| PWWP2B | ENST00000631148 | down | -1.1956158 | 0.0135151 |
| ZZZ3 | ENST00000370801 | down | -1.1956519 | 0.0069348 |
| C2CD3 | ENST00000313663 | down | -1.1960254 | 0.017519 |
| TTC1 | ENST00000231238 | down | -1.1960492 | 0.0001296 |
| GM2A | NM_001167607 | down | -1.1962557 | 0.0011351 |
| TAF1A | ENST00000352967 | down | -1.1963451 | 0.0269952 |
| MLXIPL | ENST00000313375 | down | -1.1964869 | 0.0025185 |
| RFESD | ENST00000380005 | down | -1.1966341 | 0.0052657 |
| CREG1 | ENST00000370509 | down | -1.1968898 | 0.0116677 |
| ZNF773 | ENST00000593916 | down | -1.1971827 | 0.0050095 |
| PM20D2 | ENST00000275072 | down | -1.1972449 | 0.0420292 |
| ZNF593 | ENST00000270812 | down | -1.1974059 | 0.0001683 |
| CHMP4A | ENST00000347519 | down | -1.1995252 | 0.0051933 |
| NME7 | ENST00000367811 | down | -1.2005221 | 0.0069789 |
| RPS2 | ENST00000343262 | down | -1.2007731 | 0.0032548 |
| KCTD15 | ENST00000589786 | down | -1.2010005 | 0.0155255 |
| MRPL9 | ENST00000368829 | down | -1.201348 | 0.0297984 |
| TIMM8A | ENST00000647359 | down | -1.2014849 | 0.0076844 |
| SDCBP | NM_001348341 | down | -1.2015207 | 0.0329303 |
| PRH2 | ENST00000396400 | down | -1.2017368 | 0.0133762 |
| SMIM4 | ENST00000477703 | down | -1.2022986 | 0.0011077 |
| CSF1 | ENST00000369802 | down | -1.2023996 | 0.0038563 |
| TLR4 | ENST00000645071 | down | -1.2037755 | 0.0113423 |
| TFPT | ENST00000391759 | down | -1.203827 | 0.0054251 |
| LSM7 | ENST00000252622 | down | -1.2047923 | 3.294E-05 |
| CFAP53 | ENST00000398545 | down | -1.2048766 | 0.0097152 |
| RNF14 | ENST00000394514 | down | -1.2056881 | 0.0044578 |
| GEM | ENST00000297596 | down | -1.2057579 | 0.0239521 |
| CLTA | ENST00000345519 | down | -1.2065328 | 0.0001582 |
| DTNB | ENST00000288642 | down | -1.2066688 | 0.0259143 |
| CTD-2537I9.19 | ENST00000635964 | down | -1.2072833 | 0.041832 |
| SLC39A11 | ENST00000542342 | down | -1.2075824 | 0.0354927 |
| NEK4 | ENST00000233027 | down | -1.2076591 | 0.027512 |
| RNH1 | ENST00000438658 | down | -1.2081962 | 0.0054051 |
| ALDH3B1 | ENST00000342456 | down | -1.2090592 | 0.0123321 |
| ZNF276 | ENST00000289816 | down | -1.2093358 | 0.0098968 |
| MRS2 | ENST00000378386 | down | -1.2093508 | 0.0013243 |
| COX20 | ENST00000366528 | down | -1.2096283 | 0.0317884 |
| EIF2D | ENST00000271764 | down | -1.2099005 | 0.0049174 |
| PSIP1 | ENST00000380738 | down | -1.2103158 | 0.0036302 |
| PPFIBP2 | NM_001351856 | down | -1.2104958 | 0.0147526 |
| EEF1D | ENST00000395119 | down | -1.211185 | 0.0076712 |
| ZXDC | ENST00000389709 | down | -1.2111853 | 0.0087815 |
| SENP6 | ENST00000447266 | down | -1.2120087 | 0.0291666 |
| LDHB | ENST00000350669 | down | -1.2123073 | 0.0015921 |
| ZNF493 | ENST00000392288 | down | -1.2124802 | 0.0406417 |
| CYB5R4 | ENST00000369681 | down | -1.2127254 | 0.0055018 |
| EDEM1 | ENST00000256497 | down | -1.2128595 | 0.0093888 |
| DDX51 | ENST00000397333 | down | -1.2131543 | 0.0126128 |
| PRDX3 | ENST00000298510 | down | -1.2137648 | 0.0008092 |
| GPR83 | ENST00000539203 | down | -1.2142093 | 0.0493534 |
| HARBI1 | ENST00000326737 | down | -1.2144541 | 0.008616 |
| ACSL1 | ENST00000281455 | down | -1.214483 | 0.0129385 |
| ATXN2L | ENST00000325215 | down | -1.21461 | 0.0211069 |
| LIN52 | ENST00000555028 | down | -1.2159234 | 0.0277656 |
| PAPD5 | ENST00000436909 | down | -1.2159408 | 0.0213408 |
| RTN4 | ENST00000357732 | down | -1.2160199 | 0.0319205 |
| DRD4 | ENST00000176183 | down | -1.2165753 | 0.0134887 |
| ME1 | uc011dzb.2 | down | -1.2166234 | 0.035376 |
| VEGFA | ENST00000518689 | down | -1.2166494 | 0.0251456 |
| IFI16 | ENST00000295809 | down | -1.2167745 | 0.0271038 |
| AMZ2 | ENST00000359783 | down | -1.2171345 | 0.0366742 |
| WRNIP1 | ENST00000380773 | down | -1.2176863 | 0.0191433 |
| PUS3 | ENST00000530811 | down | -1.2177067 | 0.0045662 |
| RAB27A | ENST00000336787 | down | -1.217751 | 0.0281185 |
| PTPRU | ENST00000428026 | down | -1.217827 | 0.0255829 |
| VEGFA | ENST00000417285 | down | -1.2180037 | 0.0077953 |
| AASDH | ENST00000205214 | down | -1.2190895 | 0.0364343 |
| FAN1 | ENST00000561607 | down | -1.2201043 | 0.0099915 |
| EIF1AX | ENST00000379607 | down | -1.2207563 | 0.00462 |
| SIGMAR1 | ENST00000477726 | down | -1.220946 | 0.0058691 |
| ZBTB8OS | ENST00000436661 | down | -1.2217504 | 0.0083094 |
| SMIM20 | ENST00000506197 | down | -1.221908 | 0.0088971 |
| FAM91A1 | ENST00000521166 | down | -1.2231428 | 0.0033345 |
| TMEM8B | ENST00000377988 | down | -1.2234474 | 0.0041884 |
| CATG00000053459.1 | HBMT00000891055 | down | -1.223503 | 0.0023806 |
| CERS3 | ENST00000394113 | down | -1.2243029 | 0.0073485 |
| TMEFF2 | ENST00000272771 | down | -1.224734 | 0.0237268 |
| VEGFA | ENST00000523950 | down | -1.2250187 | 0.0041136 |
| TRMT12 | ENST00000328599 | down | -1.2259014 | 0.0234033 |
| SPG20 | ENST00000494062 | down | -1.2260043 | 0.0208937 |
| ASAH2 | ENST00000395526 | down | -1.2261978 | 0.0382635 |
| CEACAM3 | ENST00000630848 | down | -1.2263915 | 0.047703 |
| POU5F1 | ENST00000513407 | down | -1.2280793 | 0.0017411 |
| PRPSAP2 | ENST00000542013 | down | -1.2284964 | 0.0022753 |
| CATG00000051919.1 | MICT00000209856 | down | -1.229047 | 0.0374026 |
| RPS7 | ENST00000304921 | down | -1.2294158 | 0.0305566 |
| CLTA | NM_001311205 | down | -1.2294681 | 0.0002613 |
| DFNA5 | ENST00000419307 | down | -1.229822 | 0.0119036 |
| MGA | ENST00000566586 | down | -1.2298257 | 0.027535 |
| FRA10AC1 | uc001kjb.1 | down | -1.2301869 | 0.0336525 |
| FOXE1 | ENST00000375123 | down | -1.2304346 | 0.0108809 |
| N4BP2L1 | NM_001286460 | down | -1.2322779 | 0.0140789 |
| TMEM209 | ENST00000473456 | down | -1.2326375 | 0.0149769 |
| TFPT | ENST00000391758 | down | -1.2330561 | 0.0149724 |
| PNISR | ENST00000438806 | down | -1.2331402 | 0.0149678 |
| FGFR1OP2 | ENST00000327214 | down | -1.2342558 | 0.0149633 |
| FLNB | ENST00000358537 | down | -1.2343227 | 0.0149588 |
| MTX3 | ENST00000512528 | down | -1.2346388 | 0.0149542 |
| FAM13B | ENST00000420893 | down | -1.2348315 | 0.0149497 |
| RPE | ENST00000452025 | down | -1.2351847 | 0.0149451 |
| JMJD6 | ENST00000397625 | down | -1.2354893 | 0.0149406 |
| PIP4K2A | ENST00000545335 | down | -1.2357201 | 0.0149361 |
| RPLP1 | ENST00000260379 | down | -1.2362281 | 0.0149315 |
| JMJD6 | ENST00000445478 | down | -1.2370887 | 0.014927 |
| ZNF354A | ENST00000335815 | down | -1.2371461 | 0.0149225 |
| PMS1 | ENST00000441310 | down | -1.2380438 | 0.0149179 |
| HMGCR | ENST00000287936 | down | -1.2380515 | 0.0149134 |
| TMEM55A | ENST00000285419 | down | -1.2389567 | 0.0149089 |
| ETNK1 | ENST00000335148 | down | -1.240584 | 0.0149043 |
| DNMT3A | NM_001320892 | down | -1.2407742 | 0.0148998 |
| TP53INP1 | ENST00000342697 | down | -1.2408395 | 0.0148952 |
| YBEY | ENST00000397694 | down | -1.2413335 | 0.0148907 |
| TCAIM | ENST00000396078 | down | -1.2413961 | 0.0148862 |
| GOLGA8B | ENST00000342314 | down | -1.2415668 | 0.0148816 |
| ZNF677 | ENST00000598513 | down | -1.2415865 | 0.0148771 |
| LIFR | ENST00000263409 | down | -1.2416135 | 0.0148726 |
| APP | ENST00000358918 | down | -1.2425388 | 0.014868 |
| MBD4 | ENST00000393278 | down | -1.2425566 | 0.0148635 |
| SAYSD1 | NM_001304793 | down | -1.2427759 | 0.014859 |
| VEGFA | ENST00000413642 | down | -1.2428876 | 0.0148544 |
| LZTFL1 | ENST00000539217 | down | -1.2429685 | 0.0148499 |
| RBM23 | ENST00000346528 | down | -1.2432678 | 0.0148454 |
| ATP6V0B | ENST00000472174 | down | -1.2435717 | 0.0148408 |
| ABHD14B | ENST00000361143 | down | -1.2436295 | 0.0148363 |
| AKAP8L | ENST00000595465 | down | -1.2436964 | 0.0148317 |
| FNDC4 | ENST00000264703 | down | -1.243927 | 0.0148272 |
| AKAP8L | ENST00000397410 | down | -1.2440154 | 0.0148227 |
| PCNP | NM_001320399 | down | -1.2445718 | 0.0148181 |
| GTF2A2 | ENST00000396063 | down | -1.2462243 | 0.0148136 |
| BOD1 | ENST00000311086 | down | -1.2462538 | 0.0148091 |
| ADCY3 | ENST00000405392 | down | -1.2463881 | 0.0148045 |
| SYNGR1 | ENST00000328933 | down | -1.2465951 | 0.0148 |
| MEGF6 | ENST00000356575 | down | -1.2468203 | 0.0147955 |
| CREM | ENST00000494479 | down | -1.2470521 | 0.0147909 |
| WDR24 | ENST00000293883 | down | -1.24797 | 0.0147864 |
| TRA2B | ENST00000382191 | down | -1.2481216 | 0.0147818 |
| GOLGA1 | ENST00000373555 | down | -1.2495974 | 0.0147773 |
| TNFSF8 | ENST00000618336 | down | -1.2499225 | 0.0147728 |
| C7orf50 | ENST00000357429 | down | -1.2503843 | 0.0147682 |
| TRMT11 | ENST00000334379 | down | -1.2504773 | 0.0147637 |
| PSMA4 | ENST00000413382 | down | -1.2505226 | 0.0147592 |
| DCLRE1A | ENST00000361384 | down | -1.2506119 | 0.0147546 |
| COMMD8 | NM_001329668 | down | -1.2507192 | 0.0147501 |
| TRIM16L | NM_001353220 | down | -1.2512463 | 0.0147456 |
| SDAD1 | ENST00000356260 | down | -1.2514717 | 0.014741 |
| KIF27 | ENST00000297814 | down | -1.2517732 | 0.0147365 |
| MYH14 | ENST00000642316 | down | -1.2519872 | 0.0147319 |
| MARCH9 | ENST00000266643 | down | -1.252071 | 0.0147274 |
| SENP7 | ENST00000394091 | down | -1.2523738 | 0.0147229 |
| CPSF4 | ENST00000436336 | down | -1.253365 | 0.0147183 |
| DNAJA3 | ENST00000431375 | down | -1.2537214 | 0.0147138 |
| GSAP | NM_001350898 | down | -1.254371 | 0.0147093 |
| FRA10AC1 | ENST00000359204 | down | -1.2554426 | 0.0147047 |
| C1orf43 | ENST00000368521 | down | -1.2562913 | 0.0147002 |
| ZBTB24 | ENST00000230122 | down | -1.2564061 | 0.0146957 |
| NPM1 | NM_001355010 | down | -1.2564823 | 0.0146911 |
| HEBP1 | ENST00000014930 | down | -1.2566736 | 0.0146866 |
| CLCN6 | ENST00000346436 | down | -1.2572029 | 0.014682 |
| ZNF91 | ENST00000397082 | down | -1.2576879 | 0.0146775 |
| AMZ2 | ENST00000392720 | down | -1.2578688 | 0.014673 |
| ANKRD29 | ENST00000592179 | down | -1.2578964 | 0.0146684 |
| APP | NM_001204303 | down | -1.2584032 | 0.0146639 |
| XPOT | ENST00000332707 | down | -1.2594838 | 0.0146594 |
| VEGFA | ENST00000230480 | down | -1.2596071 | 0.0146548 |
| METAP1D | NM_001322278 | down | -1.2598234 | 0.0146503 |
| PTRH2 | ENST00000409433 | down | -1.2603323 | 0.0146458 |
| MTCH2 | ENST00000302503 | down | -1.2616324 | 0.0146412 |
| PQLC2 | ENST00000375155 | down | -1.2619394 | 0.0146367 |
| VEGFA | ENST00000518824 | down | -1.2620216 | 0.0146321 |
| MRPL9 | ENST00000368830 | down | -1.262746 | 0.0146276 |
| ADAM17 | ENST00000310823 | down | -1.262803 | 0.0146231 |
| ALDH6A1 | ENST00000350259 | down | -1.262868 | 0.0146185 |
| BRWD1 | ENST00000333229 | down | -1.2631318 | 0.014614 |
| KHNYN | ENST00000251343 | down | -1.2632187 | 0.0146095 |
| TTC13 | ENST00000366661 | down | -1.2636331 | 0.0146049 |
| NBPF3 | ENST00000318249 | down | -1.2643421 | 0.0146004 |
| HSBP1L1 | ENST00000451882 | down | -1.2643873 | 0.0145959 |
| FBXO32 | ENST00000443022 | down | -1.2650733 | 0.0145913 |
| ZNF227 | ENST00000313040 | down | -1.2658341 | 0.0145868 |
| GYPC | ENST00000356887 | down | -1.2663783 | 0.0145823 |
| COMMD8 | ENST00000381571 | down | -1.2670284 | 0.0145777 |
| RAB11FIP1 | ENST00000330843 | down | -1.2671639 | 0.0145732 |
| FAHD2A | ENST00000447036 | down | -1.2673541 | 0.0145686 |
| STYXL1 | ENST00000340062 | down | -1.267359 | 0.0145641 |
| RMND1 | ENST00000622845 | down | -1.2677903 | 0.0145596 |
| HEXB | ENST00000261416 | down | -1.2685385 | 0.014555 |
| ASTE1 | ENST00000514044 | down | -1.2688009 | 0.0145505 |
| PSMD6 | ENST00000482510 | down | -1.2688271 | 0.014546 |
| SQSTM1 | NM_001142298 | down | -1.2691173 | 0.0145414 |
| STOM | ENST00000286713 | down | -1.2691395 | 0.0145369 |
| GZF1 | ENST00000377051 | down | -1.2697647 | 0.0145324 |
| HINT2 | ENST00000259667 | down | -1.2702328 | 0.0145278 |
| FDX1L | ENST00000393708 | down | -1.270418 | 0.0145233 |
| MED1 | ENST00000300651 | down | -1.2711028 | 0.0145187 |
| PABPC1L | ENST00000372819 | down | -1.2713923 | 0.0145142 |
| RPAIN | ENST00000381209 | down | -1.2715411 | 0.0145097 |
| FAM91A1 | ENST00000334705 | down | -1.271831 | 0.0145051 |
| HAP1 | ENST00000393939 | down | -1.2718785 | 0.0145006 |
| C1orf43 | ENST00000362076 | down | -1.2725586 | 0.0144961 |
| TCN2 | ENST00000407817 | down | -1.2735629 | 0.0144915 |
| INTS3 | uc001fcv.3 | down | -1.2735829 | 0.014487 |
| LCMT1 | ENST00000380966 | down | -1.2736124 | 0.0144825 |
| PCDHGB1 | ENST00000523390 | down | -1.2745847 | 0.0144779 |
| NOX3 | ENST00000159060 | down | -1.2747282 | 0.0144734 |
| C8orf59 | ENST00000612809 | down | -1.2748034 | 0.0144688 |
| CMBL | ENST00000296658 | down | -1.2748823 | 0.0144643 |
| CTB-43P18.3 | ENST00000509990 | down | -1.2749289 | 0.0144598 |
| THYN1 | ENST00000341541 | down | -1.2755264 | 0.0144552 |
| NRBF2 | ENST00000435510 | down | -1.275633 | 0.0144507 |
| GSK3B | ENST00000264235 | down | -1.2759485 | 0.0144462 |
| MBD4 | ENST00000503197 | down | -1.2766711 | 0.0144416 |
| FAM182B | ENST00000376403 | down | -1.2766802 | 0.0144371 |
| DNAJC18 | ENST00000302060 | down | -1.2766883 | 0.0144326 |
| CWC27 | NM_001318000 | down | -1.2769515 | 0.014428 |
| CHAC2 | ENST00000295304 | down | -1.2771259 | 0.0144235 |
| HMBOX1 | ENST00000287701 | down | -1.2774949 | 0.0144189 |
| SYNRG | ENST00000619541 | down | -1.2777143 | 0.0144144 |
| CCDC91 | ENST00000545336 | down | -1.2784824 | 0.0144099 |
| CHCHD10 | ENST00000401675 | down | -1.278757 | 0.0144053 |
| TP53INP1 | ENST00000448464 | down | -1.2788157 | 0.0144008 |
| PLBD2 | ENST00000545182 | down | -1.2800096 | 0.0143963 |
| ADNP | ENST00000621696 | down | -1.2801233 | 0.0143917 |
| TAF2 | ENST00000378164 | down | -1.2803868 | 0.0143872 |
| ICA1L | ENST00000392237 | down | -1.2804012 | 0.0143827 |
| STOM | NM_001270526 | down | -1.2807819 | 0.0143781 |
| GCAT | ENST00000248924 | down | -1.2812321 | 0.0143736 |
| ME2 | ENST00000382927 | down | -1.2812434 | 0.014369 |
| ETNK1 | ENST00000266517 | down | -1.2812941 | 0.0143645 |
| APOBEC3G | ENST00000407997 | down | -1.2814248 | 0.01436 |
| C9orf64 | ENST00000376344 | down | -1.2817258 | 0.0143554 |
| GLTPD2 | ENST00000331264 | down | -1.2830032 | 0.0143509 |
| GCC1 | ENST00000321407 | down | -1.283025 | 0.0143464 |
| RHOBTB3 | ENST00000379982 | down | -1.28389 | 0.0143418 |
| CD1D | uc009wss.3 | down | -1.2841506 | 0.0143373 |
| CEP70 | ENST00000264982 | down | -1.2845717 | 0.0143328 |
| FAM217A | ENST00000274673 | down | -1.2856697 | 0.0143282 |
| MDM4 | ENST00000454264 | down | -1.287232 | 0.0143237 |
| RHOQ | ENST00000238738 | down | -1.2873291 | 0.0143192 |
| HECTD2 | ENST00000371681 | down | -1.2873797 | 0.0143146 |
| STYXL1 | NM_001317788 | down | -1.2889768 | 0.0143101 |
| RRP15 | ENST00000366932 | down | -1.2892703 | 0.0143055 |
| KIZ | ENST00000620891 | down | -1.2893575 | 0.014301 |
| PBRM1 | ENST00000394830 | down | -1.2898299 | 0.0142965 |
| CMTR2 | ENST00000434935 | down | -1.2900388 | 0.0142919 |
| BLOC1S3 | ENST00000433642 | down | -1.2900575 | 0.0142874 |
| CSNK2A2 | ENST00000563307 | down | -1.2905971 | 0.0142829 |
| CATG00000032576.1 | ENCT00000178861 | down | -1.2907544 | 0.0142783 |
| TATDN1 | ENST00000519548 | down | -1.2913725 | 0.0142738 |
| SESN3 | ENST00000536441 | down | -1.2916386 | 0.0142693 |
| NDUFB9 | ENST00000276689 | down | -1.2917457 | 0.0142647 |
| NDUFB9 | ENST00000276689 | down | -1.2917457 | 0.0142602 |
| NUBPL | ENST00000281081 | down | -1.2917595 | 0.0142556 |
| NEK3 | ENST00000611833 | down | -1.291962 | 0.0142511 |
| UTP15 | ENST00000508491 | down | -1.2922513 | 0.0142466 |
| ARL13B | ENST00000535334 | down | -1.2922854 | 0.014242 |
| SRGAP1 | ENST00000355086 | down | -1.2928336 | 0.0142375 |
| TRMT10B | NM_001286953 | down | -1.2932536 | 0.014233 |
| ESRRG | ENST00000493748 | down | -1.2935817 | 0.0142284 |
| GMPR2 | ENST00000355299 | down | -1.2935983 | 0.0142239 |
| GALK2 | ENST00000327171 | down | -1.2939277 | 0.0142194 |
| THYN1 | ENST00000352327 | down | -1.2945313 | 0.0142148 |
| ADNP | ENST00000396029 | down | -1.2947056 | 0.0142103 |
| SAYSD1 | ENST00000373249 | down | -1.2953685 | 0.0142057 |
| ZMAT3 | ENST00000432729 | down | -1.2956179 | 0.0142012 |
| ATPAF1 | ENST00000329231 | down | -1.29587 | 0.0141967 |
| SEMA5B | ENST00000616742 | down | -1.2966285 | 0.0141921 |
| ACAD10 | ENST00000313698 | down | -1.2968004 | 0.0141876 |
| MSANTD4 | ENST00000301919 | down | -1.2980728 | 0.0141831 |
| CXorf23 | ENST00000379687 | down | -1.298654 | 0.0141785 |
| ZNF138 | ENST00000437743 | down | -1.2988378 | 0.014174 |
| AK2 | NM_001319139 | down | -1.2990296 | 0.0141695 |
| PUS3 | ENST00000613398 | down | -1.2990617 | 0.0141649 |
| PUS3 | ENST00000613398 | down | -1.2990617 | 0.0141604 |
| DOCK3 | ENST00000266037 | down | -1.2992602 | 0.0141558 |
| B3GAT3 | ENST00000534026 | down | -1.2992825 | 0.0141513 |
| AIG1 | ENST00000357847 | down | -1.3002377 | 0.0141468 |
| FCGBP | ENST00000616721 | down | -1.3002595 | 0.0141422 |
| PDCD7 | ENST00000204549 | down | -1.3008684 | 0.0141377 |
| TNFRSF10B | ENST00000347739 | down | -1.3023429 | 0.0141332 |
| WISP2 | ENST00000372865 | down | -1.3028098 | 0.0141286 |
| SYF2 | ENST00000354361 | down | -1.3032462 | 0.0141241 |
| ARL13B | ENST00000471138 | down | -1.3033851 | 0.0141196 |
| ME1 | ENST00000369705 | down | -1.3036191 | 0.014115 |
| VEGFA | ENST00000372067 | down | -1.304048 | 0.0141105 |
| CATG00000104415.1 | ENCT00000441226 | down | -1.3041697 | 0.0141059 |
| MNAT1 | ENST00000539616 | down | -1.3054215 | 0.0141014 |
| SPAG1 | ENST00000251809 | down | -1.306149 | 0.0140969 |
| NDUFB9 | ENST00000517367 | down | -1.3063283 | 0.0140923 |
| SP100 | ENST00000409112 | down | -1.306371 | 0.0140878 |
| CWC27 | ENST00000381070 | down | -1.3065003 | 0.0140833 |
| RALA | ENST00000005257 | down | -1.3065697 | 0.0140787 |
| IQCB1 | NM_001319107 | down | -1.3073216 | 0.0140742 |
| ZNF396 | ENST00000589332 | down | -1.3076485 | 0.0140697 |
| PAPOLA | ENST00000557320 | down | -1.3086596 | 0.0140651 |
| RMND1 | ENST00000444024 | down | -1.3094804 | 0.0140606 |
| PSIP1 | ENST00000380733 | down | -1.3101255 | 0.0140561 |
| LIN7B | ENST00000221459 | down | -1.3102914 | 0.0140515 |
| FRG1 | ENST00000226798 | down | -1.3111303 | 0.014047 |
| XPA | ENST00000375128 | down | -1.3111387 | 0.0140424 |
| SSX2IP | ENST00000605755 | down | -1.3116358 | 0.0140379 |
| CCDC25 | ENST00000522915 | down | -1.3122728 | 0.0140334 |
| MTRNR2L11 | ENST00000604646 | down | -1.3124568 | 0.0140288 |
| RAB29 | ENST00000446390 | down | -1.3129389 | 0.0140243 |
| WWP2 | ENST00000569174 | down | -1.3129795 | 0.0140198 |
| EPHA2 | ENST00000358432 | down | -1.3133101 | 0.0140152 |
| MDK | ENST00000395569 | down | -1.3138336 | 0.0140107 |
| TDP2 | ENST00000341060 | down | -1.3151877 | 0.0140062 |
| EEF1D | NM_001130053 | down | -1.3152463 | 0.0140016 |
| HMCES | ENST00000383463 | down | -1.3162775 | 0.0139971 |
| ENOSF1 | ENST00000251101 | down | -1.317305 | 0.0139925 |
| TAF1C | ENST00000567759 | down | -1.3174808 | 0.013988 |
| NBAS | ENST00000281513 | down | -1.317735 | 0.0139835 |
| SNX16 | ENST00000396330 | down | -1.3180471 | 0.0139789 |
| LRRC28 | ENST00000447360 | down | -1.3185433 | 0.0139744 |
| GUCA2A | ENST00000357001 | down | -1.3190765 | 0.0139699 |
| B9D1 | ENST00000461069 | down | -1.3191738 | 0.0139653 |
| ACP6 | NM_001323625 | down | -1.3204512 | 0.0139608 |
| RFK | ENST00000376736 | down | -1.320762 | 0.0139563 |
| MUT | ENST00000274813 | down | -1.3211593 | 0.0139517 |
| AP2S1 | ENST00000601498 | down | -1.3224799 | 0.0139472 |
| ACO2 | ENST00000216254 | down | -1.3226263 | 0.0139426 |
| LACE1 | NM_001323005 | down | -1.322893 | 0.0139381 |
| ELOVL5 | ENST00000542638 | down | -1.3238938 | 0.0139336 |
| ISCU | ENST00000431221 | down | -1.3239437 | 0.013929 |
| EIF2S2 | NM_001316364 | down | -1.3243012 | 0.0139245 |
| SLC38A1 | ENST00000439706 | down | -1.3247865 | 0.01392 |
| RBCK1 | ENST00000382181 | down | -1.3249363 | 0.0139154 |
| MMP17 | ENST00000360564 | down | -1.3252269 | 0.0139109 |
| ZNF717 | ENST00000478296 | down | -1.3255724 | 0.0139064 |
| OMA1 | ENST00000371226 | down | -1.327195 | 0.0139018 |
| ZNF793 | ENST00000445217 | down | -1.3284224 | 0.0138973 |
| IFT27 | uc003apw.3 | down | -1.3287103 | 0.0138927 |
| CASP5 | ENST00000418434 | down | -1.3289816 | 0.0138882 |
| SETD9 | ENST00000628593 | down | -1.3293018 | 0.0138837 |
| ATP6V1E1 | ENST00000399798 | down | -1.3297614 | 0.0138791 |
| HMGCL | ENST00000436439 | down | -1.3298457 | 0.0138746 |
| TXLNB | NM_153235 | down | -1.3308881 | 0.0138701 |
| AP2S1 | ENST00000352203 | down | -1.3309801 | 0.0138655 |
| MUM1 | ENST00000591806 | down | -1.3313017 | 0.013861 |
| MLH3 | ENST00000380968 | down | -1.3313731 | 0.0138565 |
| METTL17 | ENST00000382985 | down | -1.3325204 | 0.0138519 |
| MLF1 | ENST00000619577 | down | -1.3336234 | 0.0138474 |
| NCOA7 | NM_001199620 | down | -1.3337139 | 0.0138428 |
| KRTAP19-8 | ENST00000382822 | down | -1.3359701 | 0.0138383 |
| MBD4 | ENST00000429544 | down | -1.3368435 | 0.0138338 |
| MIA2 | ENST00000396158 | down | -1.3376075 | 0.0138292 |
| ZNF415 | ENST00000421033 | down | -1.3376276 | 0.0138247 |
| PTPDC1 | ENST00000288976 | down | -1.3377651 | 0.0138202 |
| HADHB | ENST00000537713 | down | -1.3379077 | 0.0138156 |
| ZNF420 | ENST00000337995 | down | -1.3383232 | 0.0138111 |
| MRPS36 | ENST00000256441 | down | -1.3383747 | 0.0138066 |
| LRRC23 | ENST00000007969 | down | -1.3384894 | 0.013802 |
| ECHDC2 | ENST00000371522 | down | -1.3386758 | 0.0137975 |
| HEG1 | uc011bke.2 | down | -1.3389383 | 0.0137929 |
| FRA10AC1 | NM_001347714 | down | -1.3399201 | 0.0137884 |
| PTER | ENST00000535784 | down | -1.3402233 | 0.0137839 |
| SERPINB1 | ENST00000380739 | down | -1.3417359 | 0.0137793 |
| RPL38 | ENST00000311111 | down | -1.3432198 | 0.0137748 |
| PDCD2L | NM_001353433 | down | -1.3434479 | 0.0137703 |
| SEL1L | ENST00000336735 | down | -1.3436473 | 0.0137657 |
| APOE | ENST00000434152 | down | -1.3440193 | 0.0137612 |
| SIK1 | ENST00000270162 | down | -1.344443 | 0.0137567 |
| CES2 | ENST00000317091 | down | -1.3452971 | 0.0137521 |
| CEP70 | ENST00000474781 | down | -1.3458756 | 0.0137476 |
| OTUD3 | ENST00000375120 | down | -1.3466323 | 0.0137431 |
| PIP4K2A | ENST00000376573 | down | -1.3466644 | 0.0137385 |
| UNC50 | NM_001330353 | down | -1.3466824 | 0.013734 |
| GPR65 | ENST00000267549 | down | -1.3473083 | 0.0137294 |
| MIA2 | ENST00000556148 | down | -1.3497943 | 0.0137249 |
| HSD17B6 | ENST00000322165 | down | -1.3499095 | 0.0137204 |
| EIF4EBP1 | ENST00000338825 | down | -1.3510164 | 0.0137158 |
| GYPC | ENST00000409836 | down | -1.3514464 | 0.0137113 |
| CYP4F12 | ENST00000550308 | down | -1.3516306 | 0.0137068 |
| NSMCE2 | ENST00000287437 | down | -1.3525572 | 0.0137022 |
| GUF1 | ENST00000281543 | down | -1.353941 | 0.0136977 |
| ZNF572 | ENST00000319286 | down | -1.3546674 | 0.0136932 |
| DUSP12 | ENST00000367943 | down | -1.3550543 | 0.0136886 |
| DLG3 | ENST00000194900 | down | -1.3556215 | 0.0136841 |
| C7orf49 | ENST00000617987 | down | -1.3573749 | 0.0136795 |
| AMZ2 | ENST00000580753 | down | -1.3578636 | 0.013675 |
| DUSP23 | ENST00000368109 | down | -1.3581565 | 0.0136705 |
| HAS1 | ENST00000222115 | down | -1.3587397 | 0.0136659 |
| CTH | ENST00000370938 | down | -1.3588756 | 0.0136614 |
| EFR3B | ENST00000402191 | down | -1.3589414 | 0.0136569 |
| IFT27 | ENST00000433985 | down | -1.3589948 | 0.0136523 |
| TMEM115 | ENST00000266025 | down | -1.3590762 | 0.0136478 |
| PCDHGA12 | ENST00000252085 | down | -1.3591056 | 0.0136433 |
| LARP6 | ENST00000344870 | down | -1.3598272 | 0.0136387 |
| ABR | ENST00000536794 | down | -1.36035 | 0.0136342 |
| CCT6B | ENST00000421975 | down | -1.3606284 | 0.0136296 |
| FGF2 | ENST00000608478 | down | -1.3606653 | 0.0136251 |
| LAMTOR3 | ENST00000226522 | down | -1.3609674 | 0.0136206 |
| MSMO1 | ENST00000261507 | down | -1.3610994 | 0.013616 |
| DVL2 | ENST00000005340 | down | -1.361663 | 0.0136115 |
| STIM1 | ENST00000300737 | down | -1.3619104 | 0.013607 |
| MRPL27 | ENST00000442592 | down | -1.3623991 | 0.0136024 |
| CREM | ENST00000474931 | down | -1.3635491 | 0.0135979 |
| LHFPL2 | ENST00000380345 | down | -1.3641815 | 0.0135934 |
| TXNIP | ENST00000425134 | down | -1.3651426 | 0.0135888 |
| TMEM116 | ENST00000549537 | down | -1.3653267 | 0.0135843 |
| PCDHGB6 | ENST00000616430 | down | -1.3664875 | 0.0135797 |
| ADAL | ENST00000562188 | down | -1.3668557 | 0.0135752 |
| ASCC3 | ENST00000369143 | down | -1.367034 | 0.0135707 |
| ZNF232 | ENST00000575898 | down | -1.3682266 | 0.0135661 |
| C21orf91 | ENST00000284881 | down | -1.3692056 | 0.0135616 |
| RUFY3 | ENST00000381006 | down | -1.3694257 | 0.0135571 |
| CATG00000107360.1 | MICT00000369011 | down | -1.3698295 | 0.0135525 |
| PIGL | ENST00000225609 | down | -1.3705438 | 0.013548 |
| CLTA | ENST00000396603 | down | -1.3727628 | 0.0135435 |
| INSR | ENST00000341500 | down | -1.3729904 | 0.0135389 |
| ANKS3 | ENST00000614075 | down | -1.3731507 | 0.0135344 |
| ZNF518A | ENST00000614149 | down | -1.3740753 | 0.0135298 |
| RNF24 | ENST00000358395 | down | -1.374108 | 0.0135253 |
| MRPL47 | ENST00000476781 | down | -1.374728 | 0.0135208 |
| LRPPRC | ENST00000260665 | down | -1.3747326 | 0.0135162 |
| MTHFS | ENST00000258874 | down | -1.3758989 | 0.0135117 |
| SLC2A8 | ENST00000610552 | down | -1.3761314 | 0.0135072 |
| HOMER2 | ENST00000304231 | down | -1.3761333 | 0.0135026 |
| CCDC58 | ENST00000291458 | down | -1.3764573 | 0.0134981 |
| PSAT1 | ENST00000347159 | down | -1.3769137 | 0.0134936 |
| UQCRC2 | ENST00000268379 | down | -1.3773083 | 0.013489 |
| VEGFB | ENST00000426086 | down | -1.3788467 | 0.0134845 |
| RPL3 | NM_001033853 | down | -1.3791227 | 0.01348 |
| C5orf51 | ENST00000381647 | down | -1.3796883 | 0.0134754 |
| CISD1 | ENST00000333926 | down | -1.3806805 | 0.0134709 |
| TOLLIP | ENST00000525159 | down | -1.3814239 | 0.0134663 |
| IKZF1 | ENST00000492782 | down | -1.381507 | 0.0134618 |
| MOSPD1 | ENST00000370779 | down | -1.3819187 | 0.0134573 |
| NIT1 | ENST00000368008 | down | -1.3821352 | 0.0134527 |
| PIGH | ENST00000216452 | down | -1.3824444 | 0.0134482 |
| APTX | ENST00000476858 | down | -1.382711 | 0.0134437 |
| CEP63 | ENST00000354446 | down | -1.3829323 | 0.0134391 |
| SRP19 | ENST00000391338 | down | -1.3831485 | 0.0134346 |
| RPS16 | ENST00000339471 | down | -1.3839636 | 0.0134301 |
| PSAT1 | ENST00000376588 | down | -1.3844024 | 0.0134255 |
| NUDT2 | ENST00000379158 | down | -1.3850856 | 0.013421 |
| ZNF512 | ENST00000413371 | down | -1.3855845 | 0.0134164 |
| ZC2HC1A | ENST00000263849 | down | -1.3856938 | 0.0134119 |
| CCDC113 | ENST00000443128 | down | -1.3859385 | 0.0134074 |
| N4BP2L1 | ENST00000495479 | down | -1.3861201 | 0.0134028 |
| DPF2 | ENST00000252268 | down | -1.3862243 | 0.0133983 |
| ISCA2 | ENST00000556816 | down | -1.386355 | 0.0133938 |
| SPAG1 | ENST00000388798 | down | -1.386402 | 0.0133892 |
| MLF1 | ENST00000359117 | down | -1.388479 | 0.0133847 |
| PCM1 | ENST00000327578 | down | -1.3888372 | 0.0133802 |
| FAM120C | ENST00000477084 | down | -1.3890837 | 0.0133756 |
| LRIF1 | ENST00000369763 | down | -1.3899023 | 0.0133711 |
| MTO1 | ENST00000498286 | down | -1.3899394 | 0.0133665 |
| CC2D2A | ENST00000503292 | down | -1.3905382 | 0.013362 |
| AVL9 | ENST00000318709 | down | -1.3910461 | 0.0133575 |
| SRSF5 | ENST00000557154 | down | -1.3912902 | 0.0133529 |
| BDH1 | ENST00000392378 | down | -1.3914846 | 0.0133484 |
| ZBTB41 | ENST00000367405 | down | -1.3915407 | 0.0133439 |
| GBAS | ENST00000322090 | down | -1.3922455 | 0.0133393 |
| MGA | ENST00000219905 | down | -1.3923672 | 0.0133348 |
| BEX2 | ENST00000536889 | down | -1.3929194 | 0.0133303 |
| CHAC1 | ENST00000617768 | down | -1.3931035 | 0.0133257 |
| SDAD1 | ENST00000395711 | down | -1.3933368 | 0.0133212 |
| BLOC1S2 | NM_001282439 | down | -1.3949118 | 0.0133166 |
| C17orf75 | ENST00000577809 | down | -1.3949143 | 0.0133121 |
| MAN2A1 | ENST00000261483 | down | -1.3951279 | 0.0133076 |
| SIRT5 | ENST00000379262 | down | -1.395439 | 0.013303 |
| MTHFS | ENST00000559722 | down | -1.3960695 | 0.0132985 |
| IRF2BP2 | ENST00000366609 | down | -1.3970621 | 0.013294 |
| DCXR | ENST00000306869 | down | -1.397359 | 0.0132894 |
| FUCA1 | ENST00000374479 | down | -1.3974054 | 0.0132849 |
| CWC27 | ENST00000508024 | down | -1.397965 | 0.0132804 |
| DPH5 | ENST00000342173 | down | -1.3984816 | 0.0132758 |
| PLCB1 | ENST00000338037 | down | -1.3994332 | 0.0132713 |
| SRL | ENST00000399609 | down | -1.3994567 | 0.0132667 |
| CCDC25 | ENST00000640944 | down | -1.4010881 | 0.0132622 |
| TUBE1 | ENST00000368662 | down | -1.4012739 | 0.0132577 |
| GCOM1 | NM_001018091 | down | -1.4014198 | 0.0132531 |
| ZNF28 | ENST00000457749 | down | -1.4019158 | 0.0132486 |
| C19orf18 | ENST00000314391 | down | -1.4022247 | 0.0132441 |
| ENOPH1 | ENST00000505846 | down | -1.4035512 | 0.0132395 |
| RIPOR2 | ENST00000643898 | down | -1.4046098 | 0.013235 |
| PPIP5K1 | ENST00000396923 | down | -1.4047222 | 0.0132305 |
| MTIF2 | NM_001321004 | down | -1.4050403 | 0.0132259 |
| GPX2 | ENST00000612794 | down | -1.4059931 | 0.0132214 |
| DCAF4 | ENST00000509153 | down | -1.4065802 | 0.0132169 |
| NCOA1 | ENST00000348332 | down | -1.4070499 | 0.0132123 |
| TRA2A | ENST00000297071 | down | -1.4075431 | 0.0132078 |
| EIF3L | ENST00000381683 | down | -1.4077027 | 0.0132032 |
| MTIF2 | ENST00000263629 | down | -1.4077275 | 0.0131987 |
| SCYL1 | ENST00000524944 | down | -1.4085866 | 0.0131942 |
| TWISTNB | ENST00000222567 | down | -1.4087319 | 0.0131896 |
| DEGS2 | ENST00000305631 | down | -1.4090826 | 0.0131851 |
| TNFRSF14 | NM_001297605 | down | -1.4110094 | 0.0131806 |
| ATP2C1 | ENST00000507488 | down | -1.4115191 | 0.013176 |
| TIGD2 | ENST00000317005 | down | -1.4127866 | 0.0131715 |
| MSANTD4 | NM_001318748 | down | -1.4128154 | 0.013167 |
| TARS | ENST00000455217 | down | -1.412973 | 0.0131624 |
| UQCC2 | ENST00000607484 | down | -1.4141821 | 0.0131579 |
| C14orf39 | ENST00000321731 | down | -1.4148409 | 0.0131533 |
| EMC4 | ENST00000267750 | down | -1.4153787 | 0.0131488 |
| KATNAL2 | ENST00000245121 | down | -1.4157489 | 0.0131443 |
| CEP63 | ENST00000337090 | down | -1.4157531 | 0.0131397 |
| RPP21 | ENST00000436442 | down | -1.4159029 | 0.0131352 |
| DNAJA4 | ENST00000343789 | down | -1.4163678 | 0.0131307 |
| TRA2A | ENST00000538367 | down | -1.4164887 | 0.0131261 |
| TTLL7 | NM_001350214 | down | -1.4166001 | 0.0131216 |
| MME | ENST00000462745 | down | -1.4172867 | 0.0131171 |
| RAB11FIP1 | ENST00000287263 | down | -1.4175431 | 0.0131125 |
| PLIN2 | ENST00000276914 | down | -1.4182121 | 0.013108 |
| SMC6 | ENST00000448223 | down | -1.4185751 | 0.0131034 |
| RPL36A | ENST00000614077 | down | -1.4195951 | 0.0130989 |
| RUFY3 | ENST00000226328 | down | -1.4201419 | 0.0130944 |
| MRPL41 | ENST00000371443 | down | -1.4201535 | 0.0130898 |
| RPL18 | ENST00000552588 | down | -1.4205924 | 0.0130853 |
| CATG00000033939.1 | ENCT00000185214 | down | -1.4208476 | 0.0130808 |
| GOLGA8B | uc001zip.3 | down | -1.4229556 | 0.0130762 |
| TBC1D7 | NM_001318809 | down | -1.4230681 | 0.0130717 |
| ATP2C1 | ENST00000533801 | down | -1.4233521 | 0.0130672 |
| C2CD3 | ENST00000334126 | down | -1.4235582 | 0.0130626 |
| HHATL | ENST00000441594 | down | -1.4243647 | 0.0130581 |
| NVL | ENST00000391875 | down | -1.4245641 | 0.0130535 |
| ENOSF1 | ENST00000383578 | down | -1.4246164 | 0.013049 |
| RHBDD1 | ENST00000341329 | down | -1.4255871 | 0.0130445 |
| RPE | ENST00000354506 | down | -1.426154 | 0.0130399 |
| RAB11FIP2 | ENST00000355624 | down | -1.4267436 | 0.0130354 |
| MRPS15 | ENST00000373116 | down | -1.4267711 | 0.0130309 |
| ZNF213 | ENST00000416391 | down | -1.4282945 | 0.0130263 |
| TATDN1 | ENST00000630259 | down | -1.4313629 | 0.0130218 |
| PRSS23 | ENST00000533902 | down | -1.4318064 | 0.0130173 |
| PCM1 | ENST00000524226 | down | -1.4318368 | 0.0130127 |
| SLC35A4 | ENST00000514199 | down | -1.4320818 | 0.0130082 |
| CNOT10 | ENST00000331889 | down | -1.4326714 | 0.0130036 |
| SPAG16 | ENST00000432529 | down | -1.4342779 | 0.0129991 |
| ZNF521 | ENST00000361524 | down | -1.4348981 | 0.0129946 |
| NFE2L2 | NM_001313902 | down | -1.4357245 | 0.01299 |
| EPDR1 | ENST00000423717 | down | -1.4359968 | 0.0129855 |
| ATP6V1E1 | ENST00000399796 | down | -1.4362786 | 0.012981 |
| ETV7 | ENST00000627426 | down | -1.4364872 | 0.0129764 |
| PCYT2 | ENST00000571105 | down | -1.4378002 | 0.0129719 |
| ARL13B | ENST00000394222 | down | -1.4389183 | 0.0129674 |
| L3MBTL2 | ENST00000216237 | down | -1.4391037 | 0.0129628 |
| CCPG1 | ENST00000442196 | down | -1.4392035 | 0.0129583 |
| BCAP29 | ENST00000445771 | down | -1.4404675 | 0.0129538 |
| AP2S1 | ENST00000601649 | down | -1.4405707 | 0.0129492 |
| DHODH | ENST00000219240 | down | -1.4407708 | 0.0129447 |
| TXNIP | ENST00000582401 | down | -1.441524 | 0.0129401 |
| TXNIP | ENST00000582401 | down | -1.441524 | 0.0129356 |
| CADM1 | ENST00000542447 | down | -1.4423559 | 0.0129311 |
| GUCA1B | ENST00000230361 | down | -1.4428433 | 0.0129265 |
| ASCC3 | ENST00000369162 | down | -1.4435234 | 0.012922 |
| MAFG | ENST00000357736 | down | -1.443575 | 0.0129175 |
| PHF20L1 | ENST00000337920 | down | -1.4438052 | 0.0129129 |
| MITD1 | ENST00000409107 | down | -1.4440995 | 0.0129084 |
| HOMER2 | ENST00000450735 | down | -1.4452319 | 0.0129039 |
| KIZ | ENST00000616848 | down | -1.4452754 | 0.0128993 |
| TMEM67 | ENST00000453321 | down | -1.4453347 | 0.0128948 |
| CASP1 | ENST00000533400 | down | -1.4472665 | 0.0128902 |
| HMBOX1 | ENST00000524238 | down | -1.447763 | 0.0128857 |
| SLC25A51 | ENST00000242275 | down | -1.4482278 | 0.0128812 |
| ZFYVE28 | ENST00000515169 | down | -1.4483097 | 0.0128766 |
| IL31RA | NM_001297572 | down | -1.4483418 | 0.0128721 |
| PTPRU | ENST00000373779 | down | -1.4496803 | 0.0128676 |
| ASB7 | ENST00000332783 | down | -1.4498948 | 0.012863 |
| POLB | ENST00000265421 | down | -1.4502998 | 0.0128585 |
| LARS | ENST00000394434 | down | -1.451051 | 0.012854 |
| ZNF770 | ENST00000356321 | down | -1.4516756 | 0.0128494 |
| TMEM161B | ENST00000296595 | down | -1.4516955 | 0.0128449 |
| TMUB2 | ENST00000357984 | down | -1.4529516 | 0.0128403 |
| SRPX | ENST00000538295 | down | -1.4541179 | 0.0128358 |
| RANGRF | ENST00000580434 | down | -1.4546669 | 0.0128313 |
| IRF2BP2 | ENST00000366610 | down | -1.4547771 | 0.0128267 |
| LYRM9 | ENST00000379103 | down | -1.4551502 | 0.0128222 |
| ZNF701 | ENST00000540331 | down | -1.4594871 | 0.0128177 |
| NCOA7 | ENST00000368357 | down | -1.4595369 | 0.0128131 |
| SMUG1 | ENST00000337581 | down | -1.4597083 | 0.0128086 |
| ZFAND1 | ENST00000523096 | down | -1.4600593 | 0.0128041 |
| ISCU | ENST00000311893 | down | -1.4615121 | 0.0127995 |
| BBIP1 | ENST00000448814 | down | -1.4621887 | 0.012795 |
| CEP41 | ENST00000343969 | down | -1.4623179 | 0.0127904 |
| FAM24B | ENST00000368898 | down | -1.4627429 | 0.0127859 |
| STARD4 | ENST00000296632 | down | -1.4648866 | 0.0127814 |
| TGFB2 | ENST00000366929 | down | -1.4655286 | 0.0127768 |
| CXCL16 | ENST00000293778 | down | -1.4680812 | 0.0127723 |
| CASP1 | ENST00000526568 | down | -1.4684989 | 0.0127678 |
| EPAS1 | ENST00000263734 | down | -1.4686851 | 0.0127632 |
| NCAM1 | NM_000615 | down | -1.4694309 | 0.0127587 |
| LRP8 | ENST00000354412 | down | -1.4698505 | 0.0127542 |
| RPE | ENST00000411934 | down | -1.4701301 | 0.0127496 |
| EPDR1 | ENST00000199448 | down | -1.470218 | 0.0127451 |
| TBCK | ENST00000432496 | down | -1.4734777 | 0.0127405 |
| SLC16A4 | ENST00000369779 | down | -1.4767798 | 0.012736 |
| ADM2 | ENST00000395738 | down | -1.4767914 | 0.0127315 |
| ADCK2 | ENST00000072869 | down | -1.4775273 | 0.0127269 |
| FOXRED2 | ENST00000397224 | down | -1.4778656 | 0.0127224 |
| RPS6KA1 | ENST00000374168 | down | -1.4778976 | 0.0127179 |
| C7orf50 | NM_001134395 | down | -1.4782525 | 0.0127133 |
| MAGI1 | ENST00000402939 | down | -1.4788334 | 0.0127088 |
| GTF2H3 | ENST00000543341 | down | -1.479281 | 0.0127043 |
| MBD4 | ENST00000249910 | down | -1.4797334 | 0.0126997 |
| NCOA1 | ENST00000288599 | down | -1.4802408 | 0.0126952 |
| RP11-923I11.8 | ENST00000642069 | down | -1.4810423 | 0.0126906 |
| GAPVD1 | ENST00000394105 | down | -1.4814438 | 0.0126861 |
| PMS1 | NM_001321044 | down | -1.4816087 | 0.0126816 |
| MAMDC2 | ENST00000377182 | down | -1.4845823 | 0.012677 |
| PHLDA3 | ENST00000367311 | down | -1.4851404 | 0.0126725 |
| MRPL24 | ENST00000368211 | down | -1.4852553 | 0.012668 |
| ATP2B1 | ENST00000428670 | down | -1.4856269 | 0.0126634 |
| ARL8B | ENST00000256496 | down | -1.486529 | 0.0126589 |
| HTRA2 | ENST00000258080 | down | -1.4868791 | 0.0126544 |
| ZC3H8 | ENST00000409573 | down | -1.4868916 | 0.0126498 |
| ZFAND1 | ENST00000522520 | down | -1.4877617 | 0.0126453 |
| CABYR | ENST00000415309 | down | -1.4880455 | 0.0126408 |
| NEK1 | ENST00000511633 | down | -1.4886635 | 0.0126362 |
| DNAJA3 | ENST00000262375 | down | -1.4897984 | 0.0126317 |
| TEC | ENST00000381501 | down | -1.4899121 | 0.0126271 |
| C15orf61 | ENST00000342683 | down | -1.4899681 | 0.0126226 |
| FAM228B | ENST00000615575 | down | -1.4903225 | 0.0126181 |
| PEPD | ENST00000436370 | down | -1.4907868 | 0.0126135 |
| ORMDL1 | ENST00000392349 | down | -1.4911299 | 0.012609 |
| LAMA4 | ENST00000453937 | down | -1.4911394 | 0.0126045 |
| MCTP1 | ENST00000312216 | down | -1.4913933 | 0.0125999 |
| GUF1 | NM_001345867 | down | -1.4928216 | 0.0125954 |
| FABP3 | ENST00000373713 | down | -1.4929026 | 0.0125909 |
| ZNF621 | ENST00000339296 | down | -1.4960054 | 0.0125863 |
| CEP63 | ENST00000620544 | down | -1.496746 | 0.0125818 |
| ZFP82 | ENST00000392161 | down | -1.4969111 | 0.0125772 |
| SRPX | ENST00000378533 | down | -1.4979786 | 0.0125727 |
| STK26 | ENST00000496850 | down | -1.497995 | 0.0125682 |
| METTL18 | ENST00000310392 | down | -1.4995275 | 0.0125636 |
| ECD | ENST00000454759 | down | -1.5003984 | 0.0125591 |
| SFXN4 | ENST00000355697 | down | -1.5008841 | 0.0125546 |
| RRM2B | ENST00000522368 | down | -1.5013655 | 0.01255 |
| NDFIP2 | ENST00000218652 | down | -1.501378 | 0.0125455 |
| ADGRE3 | ENST00000344373 | down | -1.5015776 | 0.012541 |
| FTCDNL1 | ENST00000416668 | down | -1.5016789 | 0.0125364 |
| CASP1 | ENST00000531166 | down | -1.5022909 | 0.0125319 |
| TMEM205 | ENST00000354882 | down | -1.5034215 | 0.0125273 |
| CLCN7 | ENST00000448525 | down | -1.5035616 | 0.0125228 |
| ATP5L | ENST00000300688 | down | -1.5036352 | 0.0125183 |
| DYNC2LI1 | ENST00000260605 | down | -1.5037375 | 0.0125137 |
| FAIM | ENST00000393034 | down | -1.504145 | 0.0125092 |
| TRIAP1 | ENST00000546954 | down | -1.5046498 | 0.0125047 |
| ADAMTS9 | ENST00000498707 | down | -1.5048423 | 0.0125001 |
| ESYT1 | ENST00000394048 | down | -1.5066557 | 0.0124956 |
| ZFP3 | ENST00000318833 | down | -1.5067883 | 0.0124911 |
| FLCN | ENST00000389169 | down | -1.5067957 | 0.0124865 |
| ZNF419 | ENST00000347466 | down | -1.5071602 | 0.012482 |
| MLF1 | ENST00000469452 | down | -1.5093882 | 0.0124774 |
| NMRK1 | NM_001330679 | down | -1.5098397 | 0.0124729 |
| ENOSF1 | ENST00000580982 | down | -1.5104281 | 0.0124684 |
| MRPL47 | ENST00000392659 | down | -1.5110639 | 0.0124638 |
| LIMS1 | ENST00000544547 | down | -1.5129845 | 0.0124593 |
| CATG00000088194.1 | HBMT00001252151 | down | -1.5135001 | 0.0124548 |
| CLEC16A | ENST00000409790 | down | -1.5136582 | 0.0124502 |
| STOM | ENST00000538954 | down | -1.5137147 | 0.0124457 |
| TGFB2 | uc001hll.3 | down | -1.5137807 | 0.0124412 |
| WDR63 | ENST00000294664 | down | -1.5158083 | 0.0124366 |
| HMGB2 | ENST00000438704 | down | -1.5159673 | 0.0124321 |
| FDXR | ENST00000582944 | down | -1.5183112 | 0.0124275 |
| MAPK8IP3 | ENST00000250894 | down | -1.5186736 | 0.012423 |
| RP11-437B10.1 | ENST00000629685 | down | -1.5196429 | 0.0124185 |
| OTUD7A | NM_130901 | down | -1.519792 | 0.0124139 |
| HABP4 | ENST00000375249 | down | -1.5217433 | 0.0124094 |
| OR2A7 | ENST00000641841 | down | -1.5226426 | 0.0124049 |
| KIZ | ENST00000619574 | down | -1.5242694 | 0.0124003 |
| PRDM12 | ENST00000253008 | down | -1.5244235 | 0.0123958 |
| LSM5 | ENST00000409952 | down | -1.5251458 | 0.0123913 |
| DLC1 | ENST00000512044 | down | -1.5252 | 0.0123867 |
| PHC1 | ENST00000543824 | down | -1.525849 | 0.0123822 |
| IMMP1L | ENST00000532287 | down | -1.5265537 | 0.0123777 |
| IMMP2L | ENST00000447215 | down | -1.5286682 | 0.0123731 |
| PFDN2 | ENST00000368010 | down | -1.5290747 | 0.0123686 |
| RBBP4 | ENST00000373493 | down | -1.5306383 | 0.012364 |
| VGLL4 | ENST00000424529 | down | -1.5310731 | 0.0123595 |
| C2CD5 | ENST00000333957 | down | -1.5327309 | 0.012355 |
| HAGHL | ENST00000389703 | down | -1.534102 | 0.0123504 |
| NUPR1 | ENST00000324873 | down | -1.5344017 | 0.0123459 |
| TRAPPC2L | ENST00000564365 | down | -1.5344705 | 0.0123414 |
| ARHGAP42 | ENST00000298815 | down | -1.5347273 | 0.0123368 |
| C11orf71 | ENST00000623205 | down | -1.5355496 | 0.0123323 |
| BCKDHB | ENST00000356489 | down | -1.5362472 | 0.0123278 |
| SLC1A5 | ENST00000412532 | down | -1.5377339 | 0.0123232 |
| PCM1 | NM_001352650 | down | -1.5380028 | 0.0123187 |
| CARD8 | ENST00000519332 | down | -1.5399298 | 0.0123141 |
| EPDR1 | ENST00000425345 | down | -1.5424349 | 0.0123096 |
| ARHGAP42 | ENST00000524892 | down | -1.5460636 | 0.0123051 |
| FOPNL | ENST00000255759 | down | -1.5461359 | 0.0123005 |
| NDUFAF6 | ENST00000396111 | down | -1.5463028 | 0.012296 |
| ING3 | uc003vjp.3 | down | -1.5465553 | 0.0122915 |
| TGFBI | ENST00000442011 | down | -1.5469194 | 0.0122869 |
| NCOA1 | ENST00000395856 | down | -1.5479846 | 0.0122824 |
| TLR4 | ENST00000355622 | down | -1.5481254 | 0.0122779 |
| RPS27A | ENST00000272317 | down | -1.5495961 | 0.0122733 |
| ZNF330 | ENST00000262990 | down | -1.5510713 | 0.0122688 |
| CAAP1 | ENST00000625311 | down | -1.5516081 | 0.0122642 |
| SARS | ENST00000369923 | down | -1.5552743 | 0.0122597 |
| INSR | ENST00000302850 | down | -1.5557493 | 0.0122552 |
| TCERG1L | ENST00000368642 | down | -1.5564995 | 0.0122506 |
| RAD51D | ENST00000345365 | down | -1.5568144 | 0.0122461 |
| RMND5B | ENST00000515098 | down | -1.5579975 | 0.0122416 |
| ISCU | ENST00000392807 | down | -1.5600567 | 0.012237 |
| RPRD1A | ENST00000399022 | down | -1.5605734 | 0.0122325 |
| SIKE1 | ENST00000060969 | down | -1.5607349 | 0.012228 |
| KATNAL2 | NM_001353899 | down | -1.5607827 | 0.0122234 |
| ADH1C | ENST00000515683 | down | -1.562089 | 0.0122189 |
| ADH1B | ENST00000305046 | down | -1.5640298 | 0.0122143 |
| VPS13C | ENST00000644861 | down | -1.5658614 | 0.0122098 |
| CEP63 | ENST00000513612 | down | -1.5667378 | 0.0122053 |
| RNASET2 | ENST00000508775 | down | -1.56764 | 0.0122007 |
| DFNA5 | ENST00000409970 | down | -1.5683128 | 0.0121962 |
| IFRD1 | ENST00000535603 | down | -1.5685019 | 0.0121917 |
| UMAD1 | ENST00000636849 | down | -1.5692363 | 0.0121871 |
| PDE12 | ENST00000311180 | down | -1.5692843 | 0.0121826 |
| DRC7 | ENST00000336825 | down | -1.5696149 | 0.0121781 |
| ITGB3 | ENST00000559488 | down | -1.5706162 | 0.0121735 |
| TMEM68 | ENST00000521229 | down | -1.5714305 | 0.012169 |
| NARS | ENST00000256854 | down | -1.5718398 | 0.0121644 |
| TMEM97 | ENST00000226230 | down | -1.5725581 | 0.0121599 |
| LONP1 | ENST00000593119 | down | -1.573005 | 0.0121554 |
| PTPDC1 | NM_001253830 | down | -1.5730369 | 0.0121508 |
| CHKA | ENST00000265689 | down | -1.5736965 | 0.0121463 |
| CCDC159 | ENST00000588790 | down | -1.5757183 | 0.0121418 |
| GLDN | ENST00000335449 | down | -1.5766978 | 0.0121372 |
| TMIE | ENST00000326431 | down | -1.5772747 | 0.0121327 |
| STARD10 | ENST00000543304 | down | -1.5793801 | 0.0121282 |
| SDCCAG8 | NM_001350249 | down | -1.5795207 | 0.0121236 |
| PMAIP1 | ENST00000316660 | down | -1.5804544 | 0.0121191 |
| SERINC5 | ENST00000507668 | down | -1.5809883 | 0.0121146 |
| RPAIN | ENST00000536255 | down | -1.5819365 | 0.01211 |
| KRT18 | ENST00000550600 | down | -1.5831501 | 0.0121055 |
| SIKE1 | ENST00000369528 | down | -1.5842226 | 0.0121009 |
| AIG1 | ENST00000629020 | down | -1.5852074 | 0.0120964 |
| SVBP | ENST00000372522 | down | -1.5903903 | 0.0120919 |
| RPL18 | ENST00000550973 | down | -1.591212 | 0.0120873 |
| ARL8B | ENST00000611208 | down | -1.5941408 | 0.0120828 |
| ADH1A | ENST00000209668 | down | -1.5956044 | 0.0120783 |
| BBIP1 | ENST00000423273 | down | -1.5957439 | 0.0120737 |
| PPIL2 | ENST00000335025 | down | -1.5977923 | 0.0120692 |
| HSPA13 | ENST00000285667 | down | -1.5991741 | 0.0120647 |
| SPIRE1 | ENST00000410092 | down | -1.5998933 | 0.0120601 |
| RPE | ENST00000435437 | down | -1.6021279 | 0.0120556 |
| TSEN15 | ENST00000533373 | down | -1.6112657 | 0.012051 |
| CIRBP | ENST00000320936 | down | -1.6117708 | 0.0120465 |
| METTL6 | ENST00000453819 | down | -1.612167 | 0.012042 |
| MLH3 | ENST00000556740 | down | -1.6125233 | 0.0120374 |
| RRM2B | ENST00000251810 | down | -1.6126111 | 0.0120329 |
| TRAPPC6A | ENST00000592647 | down | -1.6129255 | 0.0120284 |
| FAM13C | ENST00000611933 | down | -1.6134362 | 0.0120238 |
| ECHDC1 | ENST00000368291 | down | -1.6179138 | 0.0120193 |
| TMEM68 | ENST00000523073 | down | -1.6201614 | 0.0120148 |
| TCEA1 | ENST00000396401 | down | -1.6217001 | 0.0120102 |
| CACNA2D3 | ENST00000474759 | down | -1.6220502 | 0.0120057 |
| DNAJC27 | ENST00000534855 | down | -1.6222732 | 0.0120011 |
| TMEM205 | ENST00000586956 | down | -1.6239792 | 0.0119966 |
| TMEM205 | ENST00000586956 | down | -1.6239792 | 0.0119921 |
| SPATA17 | ENST00000366933 | down | -1.6266762 | 0.0119875 |
| PPP1R3B | ENST00000310455 | down | -1.6269318 | 0.011983 |
| AZI2 | ENST00000334100 | down | -1.6285288 | 0.0119785 |
| C12orf66 | ENST00000398055 | down | -1.6293265 | 0.0119739 |
| ETV4 | ENST00000545954 | down | -1.6296103 | 0.0119694 |
| RPAIN | ENST00000327154 | down | -1.6305615 | 0.0119649 |
| ZNF577 | ENST00000639636 | down | -1.6318145 | 0.0119603 |
| TFAM | ENST00000373895 | down | -1.6319474 | 0.0119558 |
| TP53RK | ENST00000372114 | down | -1.6323149 | 0.0119512 |
| SNAPC1 | ENST00000216294 | down | -1.6323996 | 0.0119467 |
| ZNF468 | ENST00000595646 | down | -1.6332901 | 0.0119422 |
| MSC | ENST00000325509 | down | -1.6334963 | 0.0119376 |
| RRAGB | ENST00000262850 | down | -1.6373693 | 0.0119331 |
| VTI1A | ENST00000393077 | down | -1.6381296 | 0.0119286 |
| ELOVL4 | ENST00000369816 | down | -1.6385399 | 0.011924 |
| MAP3K8 | ENST00000375321 | down | -1.6388646 | 0.0119195 |
| RRAGC | ENST00000373001 | down | -1.6401909 | 0.011915 |
| GTF2H5 | ENST00000607778 | down | -1.6409817 | 0.0119104 |
| CERS6 | ENST00000305747 | down | -1.6415218 | 0.0119059 |
| EYA4 | ENST00000367895 | down | -1.6419179 | 0.0119013 |
| MVK | ENST00000228510 | down | -1.6422249 | 0.0118968 |
| CCDC152 | ENST00000361970 | down | -1.6430674 | 0.0118923 |
| ZNF577 | ENST00000451628 | down | -1.6437821 | 0.0118877 |
| HDDC2 | ENST00000398153 | down | -1.6462726 | 0.0118832 |
| DYNC2H1 | ENST00000375735 | down | -1.6463657 | 0.0118787 |
| CASP1 | ENST00000525825 | down | -1.6482501 | 0.0118741 |
| ZNF397 | ENST00000330501 | down | -1.6495465 | 0.0118696 |
| SRPX | ENST00000432886 | down | -1.6510855 | 0.0118651 |
| ZNF599 | ENST00000587354 | down | -1.6520777 | 0.0118605 |
| PMS1 | ENST00000432292 | down | -1.6529678 | 0.011856 |
| LAMA4 | ENST00000230538 | down | -1.6543657 | 0.0118515 |
| VAC14 | ENST00000261776 | down | -1.6565652 | 0.0118469 |
| SMYD3 | ENST00000630181 | down | -1.6568909 | 0.0118424 |
| YBEY | ENST00000397692 | down | -1.6575198 | 0.0118378 |
| STOML2 | ENST00000452248 | down | -1.6579106 | 0.0118333 |
| DNAH14 | ENST00000366849 | down | -1.657944 | 0.0118288 |
| ASTN2 | ENST00000341734 | down | -1.6589691 | 0.0118242 |
| WARS | ENST00000392882 | down | -1.6606636 | 0.0118197 |
| DHRS3 | ENST00000616661 | down | -1.6625715 | 0.0118152 |
| TRIM55 | ENST00000315962 | down | -1.6637794 | 0.0118106 |
| SLC38A1 | ENST00000398637 | down | -1.6640874 | 0.0118061 |
| ARHGAP4 | ENST00000350060 | down | -1.665313 | 0.0118016 |
| OSTM1 | ENST00000193322 | down | -1.6656368 | 0.011797 |
| FAN1 | ENST00000561594 | down | -1.6661461 | 0.0117925 |
| HSPA9 | ENST00000297185 | down | -1.6674599 | 0.0117879 |
| LYRM1 | ENST00000412082 | down | -1.6691702 | 0.0117834 |
| SCML1 | ENST00000380041 | down | -1.6704038 | 0.0117789 |
| TRAPPC6A | ENST00000585934 | down | -1.6724996 | 0.0117743 |
| ALPL | ENST00000539907 | down | -1.6726263 | 0.0117698 |
| STX3 | ENST00000529177 | down | -1.6727418 | 0.0117653 |
| GLRB | ENST00000264428 | down | -1.6734834 | 0.0117607 |
| MAP1A | ENST00000382031 | down | -1.673738 | 0.0117562 |
| GNL3 | ENST00000418458 | down | -1.6750563 | 0.0117517 |
| VTI1A | NM_001318203 | down | -1.677208 | 0.0117471 |
| KIAA0586 | ENST00000423743 | down | -1.6781378 | 0.0117426 |
| OSBPL1A | ENST00000357041 | down | -1.6801531 | 0.011738 |
| ATP2C1 | ENST00000422190 | down | -1.6810466 | 0.0117335 |
| SH3BGRL2 | ENST00000369838 | down | -1.6812964 | 0.011729 |
| ENOSF1 | ENST00000340116 | down | -1.6819393 | 0.0117244 |
| IDNK | ENST00000376419 | down | -1.6838615 | 0.0117199 |
| TIGD2 | ENST00000603357 | down | -1.6852649 | 0.0117154 |
| CASP1 | ENST00000353247 | down | -1.6861858 | 0.0117108 |
| RCAN1 | ENST00000313806 | down | -1.6877082 | 0.0117063 |
| CIRBP | ENST00000589235 | down | -1.6893848 | 0.0117018 |
| CASP1 | NM_001223 | down | -1.6895768 | 0.0116972 |
| PHGDH | ENST00000641023 | down | -1.6896344 | 0.0116927 |
| FAM46A | ENST00000369754 | down | -1.6900208 | 0.0116881 |
| PDZRN3 | ENST00000466780 | down | -1.6907648 | 0.0116836 |
| AIFM2 | ENST00000307864 | down | -1.6917122 | 0.0116791 |
| CSTA | ENST00000264474 | down | -1.694982 | 0.0116745 |
| PLD6 | ENST00000321560 | down | -1.6977292 | 0.01167 |
| ABCC6 | ENST00000205557 | down | -1.6978132 | 0.0116655 |
| SAT2 | ENST00000573566 | down | -1.7002185 | 0.0116609 |
| FBXO22 | ENST00000453211 | down | -1.7003796 | 0.0116564 |
| SEC11C | ENST00000588875 | down | -1.7016407 | 0.0116519 |
| COA6 | ENST00000366612 | down | -1.7018752 | 0.0116473 |
| TMEM178A | ENST00000281961 | down | -1.7036117 | 0.0116428 |
| KLHL17 | ENST00000338591 | down | -1.7046516 | 0.0116382 |
| C12orf66 | ENST00000311915 | down | -1.7048862 | 0.0116337 |
| RP11-27I1.4 | ENST00000464488 | down | -1.7056434 | 0.0116292 |
| PHF14 | ENST00000403050 | down | -1.7061827 | 0.0116246 |
| HS1BP3 | ENST00000304031 | down | -1.7071941 | 0.0116201 |
| LONP1 | ENST00000585374 | down | -1.7082319 | 0.0116156 |
| NOL8 | ENST00000545558 | down | -1.7088609 | 0.011611 |
| FAM89A | ENST00000366654 | down | -1.7091141 | 0.0116065 |
| NMRK1 | ENST00000376811 | down | -1.7097775 | 0.011602 |
| TLR4 | ENST00000472304 | down | -1.7100118 | 0.0115974 |
| FDFT1 | ENST00000443614 | down | -1.711166 | 0.0115929 |
| RPS16 | ENST00000601655 | down | -1.7114732 | 0.0115883 |
| TSEN15 | ENST00000361641 | down | -1.7114905 | 0.0115838 |
| GLIPR1L2 | ENST00000320460 | down | -1.7143071 | 0.0115793 |
| SP100 | ENST00000264052 | down | -1.7147701 | 0.0115747 |
| TFDP2 | ENST00000499676 | down | -1.7228732 | 0.0115702 |
| CAPS | ENST00000588776 | down | -1.7240646 | 0.0115657 |
| SPATA18 | ENST00000419395 | down | -1.7263855 | 0.0115611 |
| SAT2 | ENST00000269298 | down | -1.7270961 | 0.0115566 |
| DYRK3 | ENST00000367109 | down | -1.7273345 | 0.0115521 |
| PATL1 | ENST00000300146 | down | -1.7277633 | 0.0115475 |
| CPSF4 | ENST00000452047 | down | -1.7285343 | 0.011543 |
| MFHAS1 | ENST00000276282 | down | -1.7296253 | 0.0115385 |
| DYNC2H1 | ENST00000398093 | down | -1.7320049 | 0.0115339 |
| CCDC136 | ENST00000297788 | down | -1.732111 | 0.0115294 |
| CRY1 | ENST00000008527 | down | -1.7338496 | 0.0115248 |
| TNFSF4 | ENST00000367718 | down | -1.7345105 | 0.0115203 |
| FAM83G | ENST00000388995 | down | -1.734986 | 0.0115158 |
| RPS3 | ENST00000527446 | down | -1.7361127 | 0.0115112 |
| SETDB2 | NM_001160308 | down | -1.7381784 | 0.0115067 |
| RP11-467J12.4 | ENST00000619363 | down | -1.7388342 | 0.0115022 |
| RFXANK | ENST00000392324 | down | -1.7396733 | 0.0114976 |
| SGTB | ENST00000381007 | down | -1.7409332 | 0.0114931 |
| VPS13C | ENST00000261517 | down | -1.7416612 | 0.0114886 |
| LSM5 | ENST00000409909 | down | -1.7417208 | 0.011484 |
| COA6 | ENST00000366613 | down | -1.7419913 | 0.0114795 |
| TTC25 | NM_031421 | down | -1.7420553 | 0.0114749 |
| ECHDC3 | ENST00000379215 | down | -1.7424988 | 0.0114704 |
| DUS4L | ENST00000265720 | down | -1.7434619 | 0.0114659 |
| CCPG1 | ENST00000310958 | down | -1.7446365 | 0.0114613 |
| CTH | ENST00000346806 | down | -1.7456096 | 0.0114568 |
| SEZ6L2 | ENST00000617533 | down | -1.7463757 | 0.0114523 |
| KLF5 | ENST00000539231 | down | -1.7485907 | 0.0114477 |
| NSMCE2 | ENST00000522563 | down | -1.7497057 | 0.0114432 |
| TBC1D29 | ENST00000579181 | down | -1.7502103 | 0.0114387 |
| ZBTB8OS | ENST00000373506 | down | -1.7502641 | 0.0114341 |
| LRIF1 | ENST00000494675 | down | -1.7505312 | 0.0114296 |
| RCAN1 | ENST00000481448 | down | -1.7512337 | 0.011425 |
| THYN1 | ENST00000392594 | down | -1.7538229 | 0.0114205 |
| RCAN1 | ENST00000399272 | down | -1.7572458 | 0.011416 |
| CADM1 | ENST00000537058 | down | -1.7575751 | 0.0114114 |
| DNPEP | ENST00000523282 | down | -1.7582235 | 0.0114069 |
| SNAPC5 | ENST00000395589 | down | -1.7588227 | 0.0114024 |
| HARS | ENST00000504156 | down | -1.7590327 | 0.0113978 |
| KRR1 | ENST00000229214 | down | -1.7594215 | 0.0113933 |
| CASP5 | ENST00000531367 | down | -1.762305 | 0.0113888 |
| OXER1 | ENST00000378661 | down | -1.7638231 | 0.0113842 |
| ATP6V1B2 | ENST00000276390 | down | -1.7688587 | 0.0113797 |
| DYNC2LI1 | ENST00000605786 | down | -1.7711197 | 0.0113751 |
| CDKN1A | ENST00000405375 | down | -1.7756694 | 0.0113706 |
| RPL22L1 | ENST00000463836 | down | -1.779248 | 0.0113661 |
| MDM1 | ENST00000303145 | down | -1.7801098 | 0.0113615 |
| SETDB2 | ENST00000317257 | down | -1.7812025 | 0.011357 |
| ISCU | ENST00000547005 | down | -1.7853731 | 0.0113525 |
| SNX24 | ENST00000261369 | down | -1.7860493 | 0.0113479 |
| LAMA3 | ENST00000313654 | down | -1.7866994 | 0.0113434 |
| SPAG16 | ENST00000413312 | down | -1.7901407 | 0.0113389 |
| VPS13C | ENST00000395896 | down | -1.7912204 | 0.0113343 |
| HIST3H2A | ENST00000366695 | down | -1.7933432 | 0.0113298 |
| ZCCHC17 | ENST00000627541 | down | -1.7952133 | 0.0113252 |
| SHMT2 | ENST00000557487 | down | -1.7960649 | 0.0113207 |
| TIGD6 | ENST00000515406 | down | -1.7976867 | 0.0113162 |
| TMEM205 | ENST00000447337 | down | -1.7978778 | 0.0113116 |
| FAM47E | ENST00000424749 | down | -1.7990709 | 0.0113071 |
| CHCHD3 | ENST00000448878 | down | -1.7990756 | 0.0113026 |
| RRP9 | ENST00000232888 | down | -1.8001653 | 0.011298 |
| KRTAP10-5 | ENST00000400372 | down | -1.8010257 | 0.0112935 |
| SLC35F6 | ENST00000344420 | down | -1.8011198 | 0.011289 |
| ZNF717 | ENST00000477374 | down | -1.8016483 | 0.0112844 |
| DYRK3 | ENST00000367108 | down | -1.8031238 | 0.0112799 |
| BTF3L4 | ENST00000489308 | down | -1.8034864 | 0.0112754 |
| THAP6 | ENST00000380837 | down | -1.8037169 | 0.0112708 |
| OCIAD2 | ENST00000620187 | down | -1.8041589 | 0.0112663 |
| ARHGAP5 | ENST00000345122 | down | -1.805331 | 0.0112617 |
| ATP2B2 | ENST00000645850 | down | -1.8095639 | 0.0112572 |
| CPEB4 | ENST00000520867 | down | -1.8106573 | 0.0112527 |
| SLC25A4 | ENST00000281456 | down | -1.8112767 | 0.0112481 |
| C12orf29 | ENST00000356891 | down | -1.8113444 | 0.0112436 |
| TBC1D7 | ENST00000379307 | down | -1.8115998 | 0.0112391 |
| MOV10L1 | ENST00000540615 | down | -1.8183692 | 0.0112345 |
| NEK1 | ENST00000439128 | down | -1.8209364 | 0.01123 |
| IDNK | ENST00000376417 | down | -1.821632 | 0.0112255 |
| MLF1 | ENST00000392822 | down | -1.8233539 | 0.0112209 |
| FAM213A | ENST00000606162 | down | -1.8234911 | 0.0112164 |
| LYPLAL1 | ENST00000366928 | down | -1.8271712 | 0.0112118 |
| GCH1 | ENST00000395514 | down | -1.8280581 | 0.0112073 |
| CALCOCO1 | ENST00000262059 | down | -1.8283812 | 0.0112028 |
| MCTP1 | ENST00000429576 | down | -1.8308064 | 0.0111982 |
| EYA4 | ENST00000355167 | down | -1.834213 | 0.0111937 |
| CSTA | ENST00000479204 | down | -1.8352137 | 0.0111892 |
| LRIF1 | ENST00000485275 | down | -1.837053 | 0.0111846 |
| TOP1MT | ENST00000519148 | down | -1.8383305 | 0.0111801 |
| GHITM | ENST00000372134 | down | -1.8398525 | 0.0111756 |
| ALAS1 | ENST00000394965 | down | -1.8417242 | 0.011171 |
| PLEKHF1 | ENST00000436066 | down | -1.8440678 | 0.0111665 |
| RRAGB | ENST00000374941 | down | -1.8489014 | 0.0111619 |
| CETN3 | ENST00000283122 | down | -1.8518661 | 0.0111574 |
| NRIP3 | ENST00000309166 | down | -1.8533203 | 0.0111529 |
| C2CD5 | ENST00000446597 | down | -1.8539108 | 0.0111483 |
| MOCOS | ENST00000261326 | down | -1.8597045 | 0.0111438 |
| CYP11A1 | ENST00000268053 | down | -1.8621454 | 0.0111393 |
| SLC2A9 | ENST00000506583 | down | -1.8624418 | 0.0111347 |
| RPL37 | ENST00000274242 | down | -1.866264 | 0.0111302 |
| BAIAP2 | ENST00000428708 | down | -1.8686173 | 0.0111257 |
| CROT | ENST00000412227 | down | -1.8718194 | 0.0111211 |
| STARD10 | ENST00000334805 | down | -1.8742619 | 0.0111166 |
| PQLC2L | ENST00000468043 | down | -1.8780489 | 0.011112 |
| HEXA | ENST00000566304 | down | -1.8811548 | 0.0111075 |
| HYOU1 | ENST00000617285 | down | -1.8845653 | 0.011103 |
| KMO | ENST00000366558 | down | -1.8852569 | 0.0110984 |
| ZBTB8OS | ENST00000373501 | down | -1.8869533 | 0.0110939 |
| TMEM254 | ENST00000613758 | down | -1.8895811 | 0.0110894 |
| AGPHD1 | ENST00000563233 | down | -1.8900425 | 0.0110848 |
| MYCL | ENST00000397332 | down | -1.8910407 | 0.0110803 |
| CPSF4 | NM_001318160 | down | -1.8912947 | 0.0110758 |
| RASAL1 | ENST00000261729 | down | -1.8914057 | 0.0110712 |
| LURAP1L | ENST00000319264 | down | -1.8914418 | 0.0110667 |
| KCNS3 | ENST00000304101 | down | -1.8922375 | 0.0110621 |
| NPRL3 | ENST00000620134 | down | -1.8955081 | 0.0110576 |
| TRPV3 | ENST00000301365 | down | -1.9075889 | 0.0110531 |
| GOT1 | ENST00000370508 | down | -1.9081399 | 0.0110485 |
| BRIX1 | ENST00000336767 | down | -1.9143086 | 0.011044 |
| ZNF701 | ENST00000391785 | down | -1.9146617 | 0.0110395 |
| MARC1 | ENST00000366910 | down | -1.9153091 | 0.0110349 |
| RPL22L1 | ENST00000295830 | down | -1.9153647 | 0.0110304 |
| ISCA2 | ENST00000554924 | down | -1.9166798 | 0.0110259 |
| RNLS | ENST00000331772 | down | -1.9207934 | 0.0110213 |
| IGF2R | ENST00000356956 | down | -1.9233729 | 0.0110168 |
| ACACA | ENST00000617649 | down | -1.9312676 | 0.0110123 |
| MPZ | ENST00000533357 | down | -1.9314854 | 0.0110077 |
| TCEA1 | ENST00000522635 | down | -1.9318238 | 0.0110032 |
| SEPT8 | ENST00000378719 | down | -1.9346634 | 0.0109986 |
| WARS | ENST00000344102 | down | -1.9360725 | 0.0109941 |
| ZNF512 | ENST00000355467 | down | -1.9397126 | 0.0109896 |
| FAM154B | NM_001348700 | down | -1.9407693 | 0.010985 |
| ETFA | ENST00000433983 | down | -1.9430696 | 0.0109805 |
| ZNF518A | ENST00000624776 | down | -1.9499752 | 0.010976 |
| CCDC30 | ENST00000340612 | down | -1.955245 | 0.0109714 |
| CSNK2A2 | ENST00000262506 | down | -1.9593409 | 0.0109669 |
| MXD1 | NM_001202513 | down | -1.9702792 | 0.0109624 |
| GLIPR1L2 | ENST00000550916 | down | -1.9750093 | 0.0109578 |
| CLEC2D | ENST00000290855 | down | -1.975453 | 0.0109533 |
| SNAPC5 | ENST00000563480 | down | -1.975758 | 0.0109487 |
| ABHD4 | ENST00000428304 | down | -1.9775769 | 0.0109442 |
| CPSF4 | ENST00000292476 | down | -1.9825888 | 0.0109397 |
| DHTKD1 | ENST00000263035 | down | -1.9889235 | 0.0109351 |
| CA13 | ENST00000321764 | down | -1.9899712 | 0.0109306 |
| SOX9 | ENST00000245479 | down | -1.9945499 | 0.0109261 |
| DOK1 | ENST00000409429 | down | -1.9950722 | 0.0109215 |
| TIMM9 | ENST00000555404 | down | -2.0031839 | 0.010917 |
| PARK2 | ENST00000366898 | down | -2.009013 | 0.0109125 |
| ATP2B1 | ENST00000261173 | down | -2.0104251 | 0.0109079 |
| PQLC2L | ENST00000312275 | down | -2.013517 | 0.0109034 |
| MLF1 | ENST00000355893 | down | -2.0145916 | 0.0108988 |
| RCAN1 | ENST00000620920 | down | -2.0153629 | 0.0108943 |
| MFGE8 | NM_001310321 | down | -2.0178467 | 0.0108898 |
| MAGEA8 | ENST00000535454 | down | -2.0229828 | 0.0108852 |
| UCN | ENST00000296099 | down | -2.0260928 | 0.0108807 |
| LRRC28 | NM_001321676 | down | -2.0262011 | 0.0108762 |
| VEPH1 | ENST00000537559 | down | -2.035559 | 0.0108716 |
| CCDC169 | ENST00000503173 | down | -2.0368077 | 0.0108671 |
| PQLC2L | ENST00000449199 | down | -2.0368244 | 0.0108626 |
| CNTRL | ENST00000238341 | down | -2.0373907 | 0.010858 |
| HEXA | ENST00000268097 | down | -2.0387623 | 0.0108535 |
| TBC1D31 | ENST00000287380 | down | -2.0400764 | 0.0108489 |
| EFCAB1 | ENST00000433756 | down | -2.0411587 | 0.0108444 |
| TBC1D7 | ENST00000343141 | down | -2.0433293 | 0.0108399 |
| SCML1 | ENST00000380043 | down | -2.0434769 | 0.0108353 |
| PLEKHH2 | ENST00000282406 | down | -2.0450893 | 0.0108308 |
| NEURL2 | ENST00000372518 | down | -2.0468124 | 0.0108263 |
| LAMA3 | ENST00000399516 | down | -2.0501079 | 0.0108217 |
| KLF15 | ENST00000296233 | down | -2.0537497 | 0.0108172 |
| LYPLAL1 | ENST00000366927 | down | -2.0538479 | 0.0108127 |
| DLC1 | ENST00000358919 | down | -2.0550564 | 0.0108081 |
| HOGA1 | ENST00000370647 | down | -2.0582542 | 0.0108036 |
| STRIP2 | ENST00000249344 | down | -2.0583093 | 0.010799 |
| LDLR | ENST00000545707 | down | -2.0616013 | 0.0107945 |
| ATP2B2 | ENST00000360273 | down | -2.0635321 | 0.01079 |
| SLFN5 | ENST00000299977 | down | -2.0658711 | 0.0107854 |
| GPNMB | ENST00000381990 | down | -2.0676204 | 0.0107809 |
| SGCE | ENST00000445866 | down | -2.0692265 | 0.0107764 |
| RCBTB1 | NM_001352506 | down | -2.0794738 | 0.0107718 |
| EPHX2 | ENST00000380476 | down | -2.0802634 | 0.0107673 |
| BCAM | ENST00000270233 | down | -2.0813613 | 0.0107628 |
| ALDH6A1 | ENST00000555126 | down | -2.0863514 | 0.0107582 |
| MXD1 | ENST00000264444 | down | -2.0886487 | 0.0107537 |
| DMKN | ENST00000418261 | down | -2.0950001 | 0.0107492 |
| NEURL2 | ENST00000545238 | down | -2.1017646 | 0.0107446 |
| CDKL3 | ENST00000523054 | down | -2.1054792 | 0.0107401 |
| TIMM9 | ENST00000556367 | down | -2.1077632 | 0.0107355 |
| COA6 | ENST00000366615 | down | -2.1089365 | 0.010731 |
| DCLK2 | ENST00000296550 | down | -2.1113356 | 0.0107265 |
| TXNDC12 | ENST00000371626 | down | -2.116509 | 0.0107219 |
| BLVRB | ENST00000643519 | down | -2.1188099 | 0.0107174 |
| CDKL3 | ENST00000265334 | down | -2.1234053 | 0.0107129 |
| PRKG1 | ENST00000401604 | down | -2.127192 | 0.0107083 |
| OSBPL6 | ENST00000409045 | down | -2.127477 | 0.0107038 |
| MLXIPL | ENST00000414749 | down | -2.1296775 | 0.0106993 |
| BTG2 | ENST00000290551 | down | -2.1304648 | 0.0106947 |
| RBX1 | ENST00000216225 | down | -2.132056 | 0.0106902 |
| TBC1D7 | ENST00000356436 | down | -2.1396689 | 0.0106856 |
| IP6K3 | ENST00000293756 | down | -2.1403244 | 0.0106811 |
| OLAH | ENST00000378217 | down | -2.1446801 | 0.0106766 |
| PQLC2L | ENST00000426338 | down | -2.1463846 | 0.010672 |
| TBC1D31 | ENST00000522420 | down | -2.1500295 | 0.0106675 |
| EI24 | ENST00000615917 | down | -2.1504831 | 0.010663 |
| SLC7A11 | ENST00000280612 | down | -2.1576276 | 0.0106584 |
| BRINP3 | NM_001317188 | down | -2.1597677 | 0.0106539 |
| CLEC2D | ENST00000545918 | down | -2.1697065 | 0.0106494 |
| BNC1 | ENST00000345382 | down | -2.1706653 | 0.0106448 |
| SLC7A11 | NM_014331 | down | -2.1744075 | 0.0106403 |
| TRIB3 | NM_001301188 | down | -2.1790909 | 0.0106357 |
| CDCP1 | ENST00000296129 | down | -2.1802436 | 0.0106312 |
| GALM | ENST00000272252 | down | -2.1899786 | 0.0106267 |
| NTRK3 | ENST00000355254 | down | -2.1932224 | 0.0106221 |
| ITPR1 | ENST00000456211 | down | -2.1979055 | 0.0106176 |
| DYNC2LI1 | ENST00000406852 | down | -2.198065 | 0.0106131 |
| ALPL | ENST00000374840 | down | -2.2035824 | 0.0106085 |
| CHODL | ENST00000400127 | down | -2.2112035 | 0.010604 |
| ADHFE1 | ENST00000396623 | down | -2.2130709 | 0.0105995 |
| M6PR | NM_001207024 | down | -2.2138318 | 0.0105949 |
| C7orf31 | ENST00000409280 | down | -2.2150019 | 0.0105904 |
| SNN | ENST00000329565 | down | -2.2160742 | 0.0105858 |
| CAPS | NM_080590 | down | -2.2171016 | 0.0105813 |
| GNPDA1 | ENST00000311337 | down | -2.2191674 | 0.0105768 |
| KIF26B | ENST00000366518 | down | -2.2209758 | 0.0105722 |
| C6orf48 | ENST00000375640 | down | -2.2226312 | 0.0105677 |
| GNPDA1 | ENST00000513454 | down | -2.2282794 | 0.0105632 |
| GPC2 | ENST00000292377 | down | -2.2312801 | 0.0105586 |
| ANKMY1 | ENST00000406958 | down | -2.2330713 | 0.0105541 |
| MESP1 | ENST00000300057 | down | -2.234802 | 0.0105496 |
| PPIL6 | ENST00000521072 | down | -2.2410098 | 0.010545 |
| SLC16A4 | ENST00000369781 | down | -2.2459954 | 0.0105405 |
| TIMM9 | ENST00000556007 | down | -2.2512023 | 0.0105359 |
| CLEC2D | ENST00000261340 | down | -2.263131 | 0.0105314 |
| SSX2IP | ENST00000437941 | down | -2.2640658 | 0.0105269 |
| EPHX2 | NM_001256484 | down | -2.2691279 | 0.0105223 |
| CYGB | ENST00000293230 | down | -2.2704707 | 0.0105178 |
| CDKN1A | ENST00000244741 | down | -2.27468 | 0.0105133 |
| CCNB1IP1 | ENST00000437553 | down | -2.2777771 | 0.0105087 |
| CHODL | ENST00000400135 | down | -2.278001 | 0.0105042 |
| WARS | ENST00000358655 | down | -2.2817527 | 0.0104997 |
| MAGI1 | ENST00000330909 | down | -2.2909342 | 0.0104951 |
| TBC1D31 | ENST00000327098 | down | -2.299465 | 0.0104906 |
| RNH1 | ENST00000397615 | down | -2.3040727 | 0.010486 |
| KIF26B | ENST00000407071 | down | -2.3053579 | 0.0104815 |
| LYPLAL1 | NM_001350629 | down | -2.3136044 | 0.010477 |
| ABCC9 | ENST00000261200 | down | -2.3140712 | 0.0104724 |
| CASP1 | ENST00000446369 | down | -2.3145211 | 0.0104679 |
| COL21A1 | ENST00000370819 | down | -2.3263456 | 0.0104634 |
| GULP1 | ENST00000409843 | down | -2.3275203 | 0.0104588 |
| EVI2A | ENST00000462804 | down | -2.3306271 | 0.0104543 |
| NABP1 | ENST00000410026 | down | -2.3309411 | 0.0104498 |
| DACT1 | ENST00000395153 | down | -2.3345959 | 0.0104452 |
| TLR4 | ENST00000394487 | down | -2.3407492 | 0.0104407 |
| OPRK1 | ENST00000265572 | down | -2.3463768 | 0.0104362 |
| EVI2A | ENST00000247270 | down | -2.3465623 | 0.0104316 |
| BHLHE40 | ENST00000256495 | down | -2.3469782 | 0.0104271 |
| CCDC181 | ENST00000367805 | down | -2.3483127 | 0.0104225 |
| SETDB2 | NM_001320699 | down | -2.3488656 | 0.010418 |
| RNF157 | ENST00000269391 | down | -2.3497109 | 0.0104135 |
| NUTM2D | ENST00000412718 | down | -2.3512047 | 0.0104089 |
| SPATA24 | ENST00000450845 | down | -2.352005 | 0.0104044 |
| GDAP1 | ENST00000220822 | down | -2.3538181 | 0.0103999 |
| RPL13 | NM_001243130 | down | -2.3542223 | 0.0103953 |
| KIF27 | ENST00000334204 | down | -2.3552678 | 0.0103908 |
| TMEM104 | ENST00000582330 | down | -2.3563485 | 0.0103863 |
| BAIAP2 | ENST00000321300 | down | -2.3584995 | 0.0103817 |
| NEU1 | ENST00000375631 | down | -2.3598231 | 0.0103772 |
| PQLC2L | ENST00000459838 | down | -2.360997 | 0.0103726 |
| MDK | ENST00000395566 | down | -2.3636788 | 0.0103681 |
| SLC39A9 | ENST00000556605 | down | -2.3710228 | 0.0103636 |
| ITPR1 | ENST00000354582 | down | -2.372601 | 0.010359 |
| TTLL2 | ENST00000239587 | down | -2.3846998 | 0.0103545 |
| MAP2 | NM_031847 | down | -2.3915715 | 0.01035 |
| EIF1B | ENST00000232905 | down | -2.3926951 | 0.0103454 |
| IMMP2L | ENST00000331762 | down | -2.3934928 | 0.0103409 |
| DNA2 | NM_001080449 | down | -2.3978758 | 0.0103364 |
| MSMO1 | ENST00000393766 | down | -2.4002651 | 0.0103318 |
| ATF3 | ENST00000613104 | down | -2.4004664 | 0.0103273 |
| BBIP1 | ENST00000454061 | down | -2.4008328 | 0.0103227 |
| C8orf88 | ENST00000517562 | down | -2.4027916 | 0.0103182 |
| GULP1 | ENST00000451191 | down | -2.404997 | 0.0103137 |
| PPIL6 | ENST00000520723 | down | -2.4147797 | 0.0103091 |
| CDCP1 | ENST00000425231 | down | -2.4182321 | 0.0103046 |
| MITD1 | NM_001320417 | down | -2.4194571 | 0.0103001 |
| INPP4B | ENST00000509777 | down | -2.4221364 | 0.0102955 |
| MTSS1 | ENST00000325064 | down | -2.42612 | 0.010291 |
| UPK1A | ENST00000222275 | down | -2.4292137 | 0.0102865 |
| AVPI1 | ENST00000370626 | down | -2.4308296 | 0.0102819 |
| FAM46A | ENST00000320172 | down | -2.4316106 | 0.0102774 |
| FLRT3 | ENST00000378053 | down | -2.4325553 | 0.0102728 |
| CASP1 | ENST00000534497 | down | -2.4331795 | 0.0102683 |
| DPH6 | ENST00000256538 | down | -2.4371843 | 0.0102638 |
| DFNB59 | ENST00000644580 | down | -2.4385488 | 0.0102592 |
| HMGCR | ENST00000343975 | down | -2.4449188 | 0.0102547 |
| C17orf64 | ENST00000269127 | down | -2.449104 | 0.0102502 |
| VPS36 | ENST00000611132 | down | -2.4523564 | 0.0102456 |
| HSPA5 | ENST00000324460 | down | -2.4552957 | 0.0102411 |
| RNF157 | ENST00000319945 | down | -2.4572628 | 0.0102366 |
| SOHLH2 | ENST00000379881 | down | -2.4646663 | 0.010232 |
| CLEC2D | ENST00000261339 | down | -2.4670055 | 0.0102275 |
| LSM8 | ENST00000249299 | down | -2.4741993 | 0.0102229 |
| DNAJC12 | ENST00000225171 | down | -2.4758774 | 0.0102184 |
| LACTB2 | ENST00000276590 | down | -2.4778435 | 0.0102139 |
| BEND7 | ENST00000440282 | down | -2.4865772 | 0.0102093 |
| ZNF396 | NM_001322290 | down | -2.4866969 | 0.0102048 |
| CD48 | ENST00000368046 | down | -2.4871443 | 0.0102003 |
| MOV10L1 | ENST00000262794 | down | -2.488355 | 0.0101957 |
| KCNK15 | ENST00000372861 | down | -2.5078513 | 0.0101912 |
| TOP1MT | ENST00000523676 | down | -2.5161879 | 0.0101867 |
| CLYBL | ENST00000339105 | down | -2.5169539 | 0.0101821 |
| TRAPPC6A | ENST00000006275 | down | -2.5187081 | 0.0101776 |
| KIAA0586 | ENST00000619416 | down | -2.5273573 | 0.0101731 |
| NMNAT3 | NM_001320511 | down | -2.532178 | 0.0101685 |
| CENPV | ENST00000299736 | down | -2.5388941 | 0.010164 |
| TMEM128 | ENST00000254742 | down | -2.5484563 | 0.0101594 |
| RHPN2 | ENST00000254260 | down | -2.556694 | 0.0101549 |
| ASPHD2 | ENST00000215906 | down | -2.5754473 | 0.0101504 |
| TMEM169 | ENST00000454545 | down | -2.59004 | 0.0101458 |
| FHIT | ENST00000476844 | down | -2.597631 | 0.0101413 |
| PRKCE | ENST00000306156 | down | -2.5979572 | 0.0101368 |
| SPATA24 | ENST00000302091 | down | -2.6036516 | 0.0101322 |
| DMKN | ENST00000451297 | down | -2.60815 | 0.0101277 |
| EFCAB1 | ENST00000523092 | down | -2.6108806 | 0.0101232 |
| GPR1 | ENST00000612892 | down | -2.6137393 | 0.0101186 |
| LCA5L | ENST00000288350 | down | -2.6171417 | 0.0101141 |
| CNNM4 | ENST00000377075 | down | -2.6201897 | 0.0101095 |
| STRBP | ENST00000360998 | down | -2.6228025 | 0.010105 |
| DHCR7 | ENST00000355527 | down | -2.6269678 | 0.0101005 |
| SULF2 | ENST00000359930 | down | -2.6295434 | 0.0100959 |
| MKX | ENST00000419761 | down | -2.6517932 | 0.0100914 |
| SLC3A2 | ENST00000377889 | down | -2.652551 | 0.0100869 |
| CCT4 | ENST00000544079 | down | -2.6617323 | 0.0100823 |
| FBXO16 | ENST00000518734 | down | -2.6716248 | 0.0100778 |
| TIGD3 | ENST00000309880 | down | -2.6802828 | 0.0100733 |
| NGF | ENST00000369512 | down | -2.7075395 | 0.0100687 |
| ITPR1 | ENST00000357086 | down | -2.7281268 | 0.0100642 |
| FAM47E | ENST00000502320 | down | -2.7532611 | 0.0100596 |
| LDLR | ENST00000455727 | down | -2.7689534 | 0.0100551 |
| GLDN | ENST00000396399 | down | -2.7741416 | 0.0100506 |
| OSBPL6 | ENST00000392505 | down | -2.7828791 | 0.010046 |
| PRPF38B | NM_001349767 | down | -2.7856152 | 0.0100415 |
| MKNK2 | ENST00000309340 | down | -2.7865761 | 0.010037 |
| SYBU | ENST00000276646 | down | -2.7957925 | 0.0100324 |
| TMEM170B | ENST00000379426 | down | -2.8014153 | 0.0100279 |
| DEFB124 | ENST00000317676 | down | -2.8030558 | 0.0100234 |
| MAP2 | ENST00000392194 | down | -2.8084176 | 0.0100188 |
| CH507-396I9.3 | ENST00000646133 | down | -2.8109041 | 0.0100143 |
| BAIAP2 | ENST00000435091 | down | -2.8134399 | 0.0100097 |
| GULP1 | ENST00000409805 | down | -2.8323031 | 0.0100052 |
| COLEC12 | ENST00000400256 | down | -2.8344232 | 0.0100007 |
| STRBP | uc004bnv.3 | down | -2.8542353 | 0.0099961 |
| APOE | NM_001302690 | down | -2.8630292 | 0.0099916 |
| FOXA1 | ENST00000250448 | down | -2.8684643 | 0.0099871 |
| ABR | ENST00000544583 | down | -2.8884928 | 0.0099825 |
| FAM129A | ENST00000367511 | down | -2.8896558 | 0.009978 |
| CD74 | ENST00000377795 | down | -2.9012747 | 0.0099735 |
| RAB38 | ENST00000243662 | down | -2.9133555 | 0.0099689 |
| SDCBP | ENST00000424270 | down | -2.9179647 | 0.0099644 |
| TMEM27 | ENST00000380342 | down | -2.928356 | 0.0099598 |
| RAVER2 | ENST00000371072 | down | -2.9453772 | 0.0099553 |
| RCAN1 | ENST00000482533 | down | -2.9568723 | 0.0099508 |
| BCL11A | ENST00000356842 | down | -2.9655758 | 0.0099462 |
| CPA3 | ENST00000296046 | down | -2.9687572 | 0.0099417 |
| DOK5 | ENST00000262593 | down | -2.9747502 | 0.0099372 |
| BAALC | ENST00000438105 | down | -2.9804014 | 0.0099326 |
| RAB3IP | ENST00000247833 | down | -3.002875 | 0.0099281 |
| UNC5B | ENST00000373192 | down | -3.0056711 | 0.0099236 |
| LDLR | ENST00000558013 | down | -3.00952 | 0.009919 |
| BCL2 | ENST00000398117 | down | -3.0106036 | 0.0099145 |
| TDRKH | ENST00000368827 | down | -3.0284135 | 0.00991 |
| MKNK2 | ENST00000591601 | down | -3.0444389 | 0.0099054 |
| SH3BGR | ENST00000380637 | down | -3.0611663 | 0.0099009 |
| CCDC171 | ENST00000380701 | down | -3.0788038 | 0.0098963 |
| TRIM69 | ENST00000558329 | down | -3.0821858 | 0.0098918 |
| RSPO3 | ENST00000356698 | down | -3.0919785 | 0.0098873 |
| HLA-DMA | ENST00000374843 | down | -3.1047016 | 0.0098827 |
| TBP | ENST00000540980 | down | -3.1187495 | 0.0098782 |
| HESX1 | ENST00000295934 | down | -3.1336395 | 0.0098737 |
| HLA-DMB | ENST00000418107 | down | -3.1430696 | 0.0098691 |
| C11orf21 | ENST00000381153 | down | -3.1442509 | 0.0098646 |
| CFAP57 | ENST00000372492 | down | -3.1554305 | 0.0098601 |
| UBL4B | ENST00000334179 | down | -3.161295 | 0.0098555 |
| RBP7 | ENST00000294435 | down | -3.1619514 | 0.009851 |
| OSBPL1A | ENST00000319481 | down | -3.1628361 | 0.0098464 |
| ITPR1 | ENST00000443694 | down | -3.175039 | 0.0098419 |
| FLRT3 | ENST00000341420 | down | -3.1880775 | 0.0098374 |
| PTGDS | ENST00000371625 | down | -3.1899614 | 0.0098328 |
| SMKR1 | ENST00000462322 | down | -3.1933091 | 0.0098283 |
| MKNK2 | ENST00000250896 | down | -3.1953112 | 0.0098238 |
| HSPA4L | ENST00000505726 | down | -3.200063 | 0.0098192 |
| GADD45A | ENST00000617962 | down | -3.2020255 | 0.0098147 |
| GSTO2 | ENST00000338595 | down | -3.2150361 | 0.0098102 |
| CATG00000029933.1 | ENCT00000168705 | down | -3.2172631 | 0.0098056 |
| GADD45A | ENST00000370985 | down | -3.2413581 | 0.0098011 |
| SLC22A15 | ENST00000369503 | down | -3.2700103 | 0.0097965 |
| FAM134B | ENST00000306320 | down | -3.2780946 | 0.009792 |
| C10orf35 | ENST00000373279 | down | -3.3008253 | 0.0097875 |
| ZNF396 | ENST00000306346 | down | -3.3072114 | 0.0097829 |
| FLRT1 | ENST00000246841 | down | -3.311532 | 0.0097784 |
| IDH1 | ENST00000415913 | down | -3.3147944 | 0.0097739 |
| NBPF6 | ENST00000370040 | down | -3.3157353 | 0.0097693 |
| S100P | ENST00000296370 | down | -3.320817 | 0.0097648 |
| ATP2A3 | ENST00000397035 | down | -3.3277698 | 0.0097603 |
| FHIT | ENST00000492590 | down | -3.3287866 | 0.0097557 |
| EVI2B | ENST00000330927 | down | -3.3518961 | 0.0097512 |
| S1PR1 | ENST00000305352 | down | -3.3527271 | 0.0097466 |
| CD1D | NM_001319145 | down | -3.3557702 | 0.0097421 |
| ATP6V0D2 | ENST00000285393 | down | -3.3830043 | 0.0097376 |
| HSPA4L | ENST00000296464 | down | -3.3832817 | 0.009733 |
| CCDC69 | ENST00000355417 | down | -3.3883275 | 0.0097285 |
| TAC1 | ENST00000350485 | down | -3.4292921 | 0.009724 |
| SULF2 | ENST00000467815 | down | -3.4311703 | 0.0097194 |
| GKAP1 | ENST00000376365 | down | -3.4313266 | 0.0097149 |
| GPR1 | ENST00000621141 | down | -3.4340984 | 0.0097104 |
| TMEM56 | ENST00000370203 | down | -3.4643777 | 0.0097058 |
| NPC1 | ENST00000269228 | down | -3.4683787 | 0.0097013 |
| PDE4D | ENST00000502484 | down | -3.5085388 | 0.0096967 |
| PTP4A3 | ENST00000521578 | down | -3.5155194 | 0.0096922 |
| SYBU | ENST00000533895 | down | -3.5161877 | 0.0096877 |
| ITPR1 | ENST00000302640 | down | -3.5483539 | 0.0096831 |
| BORCS7-ASMT | uc009xxh.3 | down | -3.5487352 | 0.0096786 |
| DDIT3 | NM_001195055 | down | -3.5655909 | 0.0096741 |
| PECR | ENST00000265322 | down | -3.5865833 | 0.0096695 |
| DDIT3 | ENST00000547303 | down | -3.6131864 | 0.009665 |
| GKAP1 | ENST00000376371 | down | -3.6566914 | 0.0096605 |
| UNC5B | ENST00000335350 | down | -3.6663107 | 0.0096559 |
| HOXD9 | ENST00000249499 | down | -3.6790217 | 0.0096514 |
| STON2 | ENST00000267540 | down | -3.691502 | 0.0096469 |
| ATP2A3 | ENST00000397043 | down | -3.7130194 | 0.0096423 |
| CASP5 | ENST00000444749 | down | -3.7204697 | 0.0096378 |
| TRIB3 | ENST00000422053 | down | -3.7405317 | 0.0096332 |
| CPEB1 | ENST00000615198 | down | -3.7415112 | 0.0096287 |
| DDIT3 | ENST00000346473 | down | -3.7877683 | 0.0096242 |
| CTD-2370N5.3 | ENST00000578584 | down | -3.8863348 | 0.0096196 |
| TNFSF18 | ENST00000404377 | down | -3.927795 | 0.0096151 |
| HLA-DRB1 | ENST00000360004 | down | -3.9472685 | 0.0096106 |
| CIITA | ENST00000324288 | down | -3.9700225 | 0.009606 |
| ANKRD22 | ENST00000371930 | down | -3.9742417 | 0.0096015 |
| DDIT3 | ENST00000551116 | down | -3.9776738 | 0.009597 |
| SLC6A15 | ENST00000266682 | down | -4.0120715 | 0.0095924 |
| STAC | ENST00000457375 | down | -4.0538623 | 0.0095879 |
| UBXN2A | ENST00000404924 | down | -4.0544226 | 0.0095833 |
| BEX4 | ENST00000372695 | down | -4.0683231 | 0.0095788 |
| ALPK1 | ENST00000177648 | down | -4.0763514 | 0.0095743 |
| ATP2A3 | ENST00000397041 | down | -4.1460844 | 0.0095697 |
| HSPBAP1 | ENST00000306103 | down | -4.1685982 | 0.0095652 |
| STAC | ENST00000273183 | down | -4.205395 | 0.0095607 |
| CPB1 | ENST00000282957 | down | -4.2610145 | 0.0095561 |
| LRRC49 | ENST00000544974 | down | -4.3295457 | 0.0095516 |
| UNC5D | ENST00000287272 | down | -4.333352 | 0.0095471 |
| GATA3 | ENST00000346208 | down | -4.3398388 | 0.0095425 |
| MOV10L1 | ENST00000395858 | down | -4.3801106 | 0.009538 |
| HLA-DRA | ENST00000395388 | down | -4.4546679 | 0.0095334 |
| LAMP3 | ENST00000265598 | down | -4.4663111 | 0.0095289 |
| TAC1 | ENST00000346867 | down | -4.4839642 | 0.0095244 |
| ERICH2 | NM_001290030 | down | -4.4860465 | 0.0095198 |
| SCG2 | ENST00000305409 | down | -4.51054 | 0.0095153 |
| DMGDH | ENST00000255189 | down | -4.5250153 | 0.0095108 |
| STON2 | NM_033104 | down | -4.5525952 | 0.0095062 |
| ASPA | ENST00000263080 | down | -4.5679094 | 0.0095017 |
| TAC1 | NM_013998 | down | -4.6048342 | 0.0094972 |
| ELMOD1 | ENST00000265840 | down | -4.7390621 | 0.0094926 |
| SEC14L6 | ENST00000402034 | down | -4.8047398 | 0.0094881 |
| PCDH17 | ENST00000377918 | down | -4.9490782 | 0.0094835 |
| RAB33A | ENST00000257017 | down | -4.9545264 | 0.009479 |
| CASP5 | ENST00000260315 | down | -4.98285 | 0.0094745 |
| KCNE1 | ENST00000399289 | down | -5.6033832 | 0.0094699 |
| LRRN4CL | ENST00000317449 | down | -5.8838367 | 0.0094654 |
| SPX | ENST00000256969 | down | -6.273743 | 0.0094609 |
| RRAGD | ENST00000369415 | down | -6.2909281 | 0.0094563 |
| RAB39B | ENST00000369454 | down | -6.5104549 | 0.0094518 |
| PRDM16 | ENST00000270722 | down | -6.6579584 | 0.0094473 |
