# Supplementary material for: METTL7A-mediated m6A modification of corin reverses bisphosphonates-impaired osteogenic differentiation of orofacial BMSCs
Source: Int J Oral Sci. 2024 May 23;16:42. doi: 10.1038/s41368-024-00303-1 (PMC11116408; doi:10.1038/s41368-024-00303-1)
Supplement: Supplementary file 2 — Supplementary Table 2 [file 41368_2024_303_MOESM2_ESM.docx]

**Supplementary Table 2. m6A-epitranscriptomic microarray results illustrated genes with differentially m6A methylation level**

| **GeneSymbol** | **Transcript ID** | **Regulation** | **Fold change**  **（log2）** | **Pvalue** |
| --- | --- | --- | --- | --- |
| SLC7A4 | ENST00000382932 | up | 1.2092518 | 0.0471712 |
| PIP | ENST00000291009 | up | 1.1947537 | 0.0044627 |
| SKA1 | ENST00000285116 | up | 1.0982735 | 0.0015793 |
| HECW2 | ENST00000409111 | up | 1.0824311 | 0.0038404 |
| OR2T12 | ENST00000317996 | up | 1.0186615 | 0.0223583 |
| CATG00000040062.1 | MICT00000004709 | up | 0.9678516 | 0.0054666 |
| NKD2 | ENST00000296849 | up | 0.9610564 | 0.0026654 |
| CCNH | ENST00000504878 | up | 0.9597824 | 0.0340838 |
| XXbacBPG116M5.17 | ENST00000456570 | up | 0.9482129 | 0.0041199 |
| PPFIA2 | NM_001220475 | up | 0.9460274 | 0.0004564 |
| CATG00000030168.1 | ENCT00000169304 | up | 0.9403683 | 0.0128377 |
| PFKP | ENST00000381075 | up | 0.9316327 | 0.021051 |
| NTRK2 | ENST00000376214 | up | 0.9170421 | 0.0079173 |
| CLEC3B | ENST00000428034 | up | 0.911524 | 0.0093257 |
| MAGEC3 | ENST00000298296 | up | 0.888287 | 0.0232828 |
| HMMR | ENST00000358715 | up | 0.8868642 | 0.0233911 |
| PPFIA2 | ENST00000552948 | up | 0.8834444 | 0.0150465 |
| CYP7B1 | NM_001324112 | up | 0.8722231 | 0.0016028 |
| SMYD3 | ENST00000630181 | up | 0.8569092 | 0.0014332 |
| P2RX2 | ENST00000449132 | up | 0.8562075 | 0.0150215 |
| COL5A3 | ENST00000264828 | up | 0.8234556 | 0.0037091 |
| HFE | ENST00000336625 | up | 0.8227553 | 0.0313446 |
| SNX12 | ENST00000622277 | up | 0.8198777 | 0.0438379 |
| TLR2 | NM_001318795 | up | 0.8179401 | 0.0063431 |
| SETD3 | ENST00000329331 | up | 0.8131713 | 0.0471022 |
| PTGIS | ENST00000244043 | up | 0.8097467 | 0.0106676 |
| TK1 | ENST00000301634 | up | 0.8013235 | 0.0005999 |
| MYOM1 | ENST00000261606 | up | 0.7861475 | 0.0309053 |
| EVPLL | ENST00000399134 | up | 0.7831938 | 0.0276379 |
| CCDC88A | ENST00000436346 | up | 0.7737692 | 0.0098003 |
| OLFM2 | ENST00000264833 | up | 0.7598301 | 0.0285718 |
| LAYN | ENST00000530962 | up | 0.7583228 | 0.0206766 |
| TMEM130 | ENST00000339375 | up | 0.7462726 | 0.0067068 |
| PLCB2 | ENST00000260402 | up | 0.7438221 | 0.0394335 |
| CACNB2 | ENST00000645287 | up | 0.7411528 | 0.0009132 |
| RAD51AP1 | ENST00000352618 | up | 0.7404176 | 0.0434933 |
| VASH2 | ENST00000366966 | up | 0.7365536 | 0.0048602 |
| ARHGEF40 | ENST00000298694 | up | 0.7279556 | 0.0217453 |
| CENPW | ENST00000368325 | up | 0.7198362 | 0.0410734 |
| NCAPG | ENST00000251496 | up | 0.7191039 | 0.0047349 |
| THAP1 | ENST00000345117 | up | 0.7143986 | 0.0277864 |
| ANLN | ENST00000396068 | up | 0.7044465 | 0.0301191 |
| WISP1 | ENST00000250160 | up | 0.6980605 | 0.0004892 |
| C18orf54 | ENST00000382911 | up | 0.6976756 | 0.0275867 |
| LMO1 | ENST00000428101 | up | 0.6948378 | 0.0110619 |
| ASPM | ENST00000294732 | up | 0.6937663 | 0.0008574 |
| FAM111B | ENST00000529618 | up | 0.6819415 | 0.0006881 |
| IGF2 | ENST00000381395 | up | 0.6817859 | 0.003213 |
| FLI1 | ENST00000527786 | up | 0.6743118 | 0.0059027 |
| RIPPLY1 | ENST00000411805 | up | 0.6620923 | 0.0095074 |
| DAB2 | ENST00000545653 | up | 0.6615749 | 0.0077397 |
| NDP | ENST00000642620 | up | 0.660663 | 0.0385554 |
| SPRY4 | ENST00000434127 | up | 0.6590565 | 0.0167557 |
| MPZL1 | ENST00000359523 | up | 0.6576728 | 0.0269008 |
| ARRB1 | ENST00000360025 | up | 0.6576034 | 0.0234544 |
| E2F8 | ENST00000620009 | up | 0.6532103 | 0.0005437 |
| CORIN | ENST00000273857 | up | 0.6526252 | 0.0003164 |
| MYOCD | ENST00000425538 | up | 0.6520254 | 1.489E-05 |
| ZFYVE16 | NM_001349434 | up | 0.6502501 | 0.0035387 |
| EXTL2 | ENST00000370113 | up | 0.6480564 | 0.0219015 |
| KIFC1 | ENST00000428849 | up | 0.6478964 | 0.008832 |
| TIMD4 | ENST00000274532 | up | 0.6471028 | 0.0104168 |
| SHBG | ENST00000441599 | up | 0.6417932 | 0.0212077 |
| NFATC1 | ENST00000318065 | up | 0.6415745 | 0.00141 |
| PHLPP2 | NM_001289003 | up | 0.6407737 | 0.043084 |
| TP53I3 | ENST00000238721 | up | 0.633672 | 0.0490397 |
| TK1 | ENST00000588734 | up | 0.6242074 | 0.0038064 |
| MCF2L2 | ENST00000328913 | up | 0.6230113 | 0.0172669 |
| FAIM | ENST00000393034 | up | 0.6204578 | 0.0033974 |
| WDYHV1 | ENST00000287387 | up | 0.619323 | 0.0457306 |
| APAF1 | ENST00000359972 | up | 0.6176744 | 0.0328744 |
| RASA3 | ENST00000334062 | up | 0.6103525 | 0.0018799 |
| P2RX1 | ENST00000225538 | up | 0.6094244 | 0.046748 |
| DDX19A | ENST00000302243 | up | 0.6087397 | 0.0344776 |
| LGALS12 | ENST00000415491 | up | 0.608421 | 0.0241418 |
| LGI3 | ENST00000306317 | up | 0.6062863 | 0.0132794 |
| ZNF160 | ENST00000429604 | up | 0.6060241 | 0.0098762 |
| FAM47A | ENST00000346193 | up | 0.6033024 | 0.0170691 |
| ACKR4 | ENST00000249887 | up | 0.6006942 | 0.0365668 |
| ITGA8 | ENST00000378076 | up | 0.600424 | 0.0026981 |
| ABCA2 | NM_212533 | up | 0.6003369 | 0.0321444 |
| ZNF74 | ENST00000403682 | up | 0.5985178 | 0.0261416 |
| ZKSCAN3 | ENST00000341464 | up | 0.5947436 | 0.0197775 |
| CALB2 | ENST00000302628 | up | 0.5944557 | 0.0380947 |
| USP10 | ENST00000570191 | up | 0.5915183 | 0.0017537 |
| RRM2 | ENST00000304567 | up | 0.5914179 | 0.0117977 |
| SLC4A11 | ENST00000642402 | up | 0.5886051 | 0.0219738 |
| MRPL52 | ENST00000397505 | down | -0.5883785 | 0.015696 |
| CH507-396I9.3 | ENST00000646133 | down | -0.5925152 | 0.0038142 |
| CDK1 | ENST00000373809 | down | -0.6009063 | 0.015495 |
| TXNDC12 | ENST00000371626 | down | -0.6029297 | 0.0271635 |
| S100A4 | ENST00000354332 | down | -0.6198152 | 0.0125884 |
| CASP5 | ENST00000526056 | down | -0.6257071 | 0.0179565 |
| HIST1H2BB | ENST00000615966 | down | -0.6283981 | 0.0026758 |
| SCG2 | ENST00000305409 | down | -0.6300798 | 0.0035933 |
| BBIP1 | ENST00000454061 | down | -0.6408674 | 0.0155169 |
| UNC5B | ENST00000335350 | down | -0.6446805 | 0.011437 |
| LYSMD2 | ENST00000454181 | down | -0.6558054 | 0.0186476 |
| CCDC69 | ENST00000355417 | down | -0.6732972 | 0.0368241 |
| VDAC2 | ENST00000313132 | down | -0.6825297 | 0.0384778 |
| NTRK3 | ENST00000355254 | down | -0.6986385 | 0.0255218 |
| MGP | ENST00000260210 | down | -0.7034153 | 0.0161645 |
| CATG00000029933.1 | ENST00000539261 | down | -0.7088658 | 0.0017802 |
| UBE2C | ENCT00000168705 | down | -0.7461943 | 0.0133455 |
| MGP | ENST00000243893 | down | -0.7479679 | 0.0004498 |
| MMP12 | ENST00000571244 | down | -0.7605107 | 0.0394858 |
| TRPV3 | ENST00000301365 | down | -0.7861016 | 0.0273312 |
| SPX | ENST00000256969 | down | -0.8013638 | 0.0033876 |
| TMEM128 | ENST00000254742 | down | -0.8877334 | 0.0440856 |
| CPA3 | ENST00000296046 | down | -1.054761 | 0.0094773 |
