# Supplementary material for: METTL7A-mediated m6A modification of corin reverses bisphosphonates-impaired osteogenic differentiation of orofacial BMSCs
Source: Int J Oral Sci. 2024 May 23;16:42. doi: 10.1038/s41368-024-00303-1 (PMC11116408; doi:10.1038/s41368-024-00303-1)
Supplement: Supplementary file 3 — Supplementary Table 3 [file 41368_2024_303_MOESM3_ESM.docx]

**Supplementary Table 3. Primers used in RT-PCR and MeRIP-qPCR**

| **Genes** | **Primers 5’- 3’** |
| --- | --- |
| Corin | **F:** CCTCCTCCGGTTCCTATTGC |
|  | R: CCAAAGGTTCACTCCCATTTGA |
| GAPDH | F: TCGGAGTCAACGGATTTGGT |
|  | R: TTCCCGTTCTCAGCCTTGAC |
| METTL3 | F: AGATGGGGTAGAAAGCCTCCT |
|  | R: TGGTCAGCATAGGTTACAAGAGT |
| METTL14 | F: GAACACAGAGCTTAAATCCCCA |
|  | R: TGTCAGCTAAACCTACATCCCTG |
| METTL7A | F: CCTTCTGAGCAATGGAGCTT |
|  | R: GGTGGCTGACGTCTGTAATCA |
| MYOCD | F: CCACCTATGGACTCAGCCTAC |
|  | R: CTCAGTGGCGTTGAAGAAGAG |
| ANLN | F: ATGTCTTCGTGGCCGATTTGA |
|  | R: CTCTGACAGTGAGTTTCCTGTTT |
| Corin (MeRIP) | F: TGGACAGAAATGCTCAATCGTG |
|  | R: AGGCCTGGCAAAAGGACAA |
| MYOCD (MeRIP) | **F:** TCTGTGGGACCTTGTGGAGT |
|  | R: GGGTACATCACACAGCCACG |
| ANLN (MeRIP) | F: TGCAGAAAAAGGCGGAAACTC |
|  | R: TCATTTCGCATTCAGTGAAACCT |
