# Supplementary figures and images for: METTL7A-mediated m6A modification of corin reverses bisphosphonates-impaired osteogenic differentiation of orofacial BMSCs
Source: Int J Oral Sci. 2024 May 23;16:42. doi: 10.1038/s41368-024-00303-1 (PMC11116408; doi:10.1038/s41368-024-00303-1)

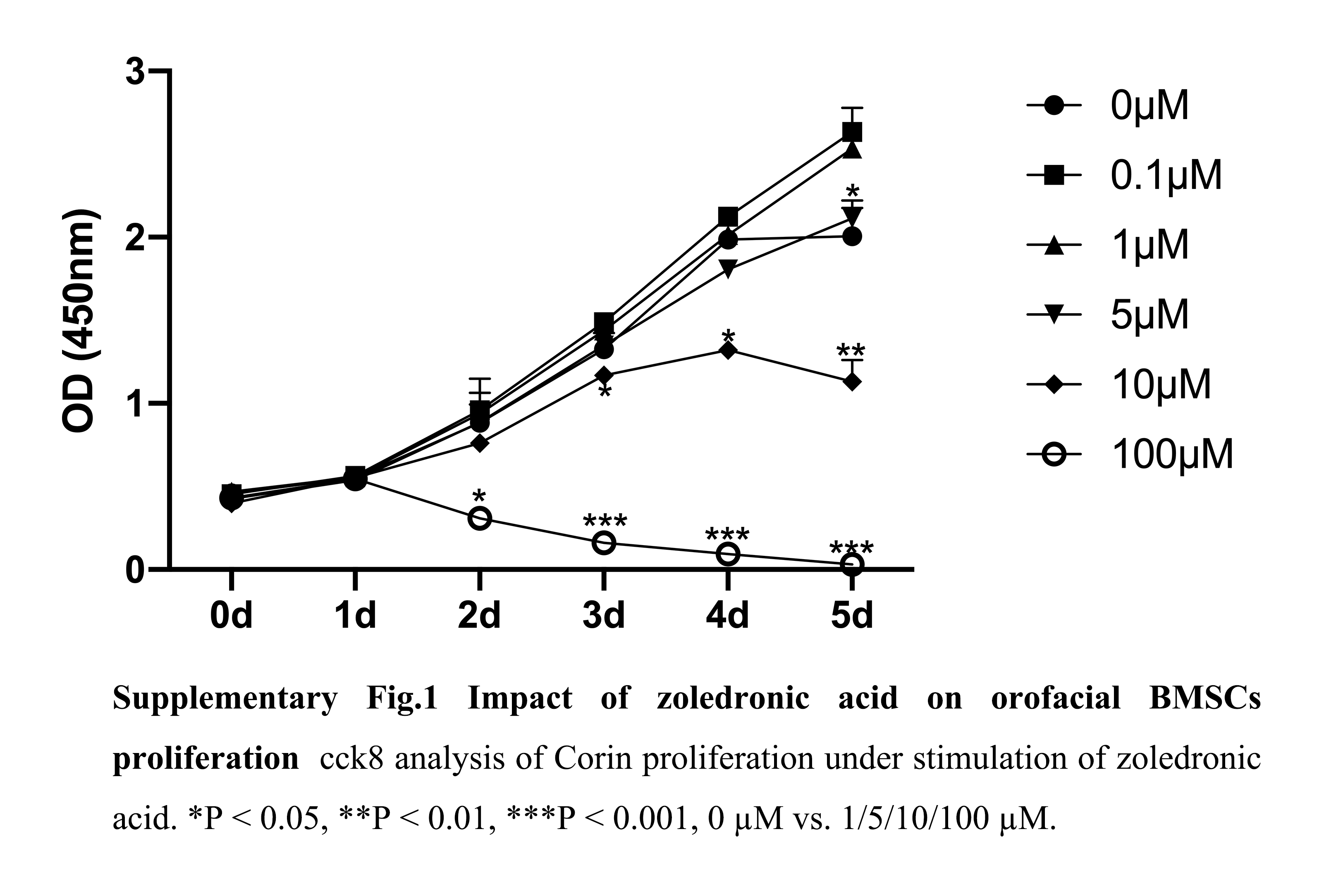

Supplement: Supplementary file 4 — Supplementary Figure 1 [file 41368_2024_303_MOESM4_ESM.tif]

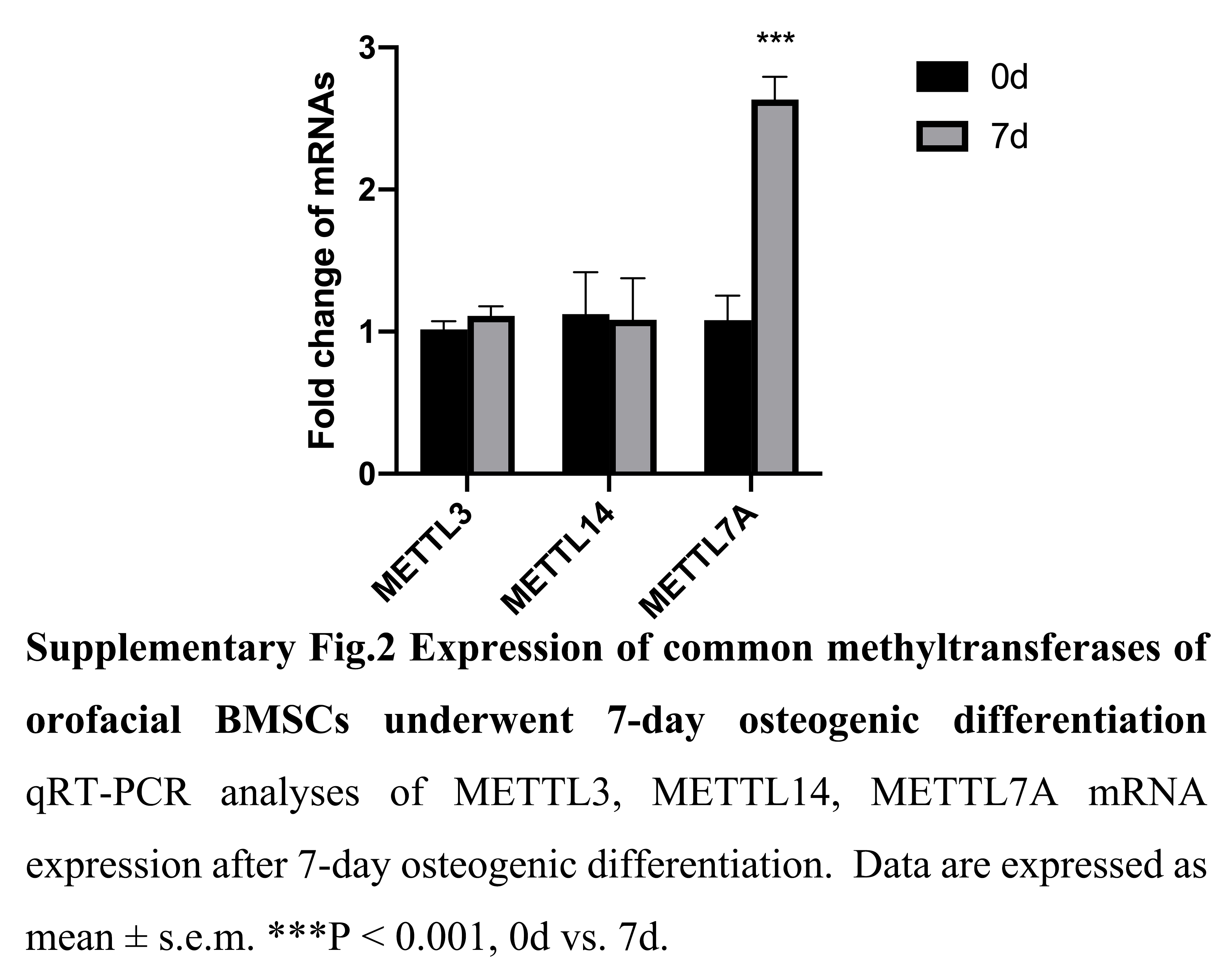

Supplement: Supplementary file 5 — Supplementary Figure 2 [file 41368_2024_303_MOESM5_ESM.tif]
